# Unravelling ring chromosome structures and formation mechanisms by short-read and long-read genomic sequencing

**DOI:** 10.1016/j.gimo.2025.103475

**Published:** 2025-11-19

**Authors:** Mei Ling Chong, Bruna Burssed, Chen Zhao, Jiadi Wen, Evelyn Ng, Bryan Szewczyk, Guilin Wang, Khi Pin Chua, Thomas Liehr, Ying Zou, Jaclyn B. Murry, Frenny Sheth, Peining Li, Maria Isabel Melaragno

**Affiliations:** 1 Department of Genetics, Yale School of Medicine, New Haven, CT; 2 Genetics Division, Universidade Federal de São Paulo, São Paulo, SP, Brazil; 3 Yale Center for Genome Analysis, Yale University, New Haven, CT; 4 Pacific Biosciences of California, Menlo Park, CA; 5 Jena University Hospital, Friedrich Schiller University, Institute of Human Genetics, Jena, Germany; 6 Cytogenomics Laboratory, Division of Molecular Pathology, Department of Pathology, School of Medicine, The Johns Hopkins University, Baltimore, MD; 7 Cytogenetics Laboratory, Greenwood Genetic Center, Greenwood, SC; 8 Department of Cytogenetics and Molecular Cytogenetics, FRIGE Institute of Human Genetics, Ahmedabad, India

**Keywords:** Breakage-fusion sequence, Ring chromosome (RC), Ring formation mechanisms, Short-real and long-read genomic sequencing (srGS/lrGS), Telomere-to-telomere (T2T) reference genome

## Abstract

**Purpose:**

Ring chromosomes (RCs) are rare cytogenetic abnormalities involving copy-number variants and chromosome instability. Identifying the breakage-fusion sequences of RCs at nucleotide-level resolution can elucidate the cytogenomic rearrangements and ring formation mechanisms.

**Methods:**

This study used short-read genomic sequencing (srGS) and long-read genomic sequencing (lrGS) alongside the telomere-to-telomere reference genome to characterize the breakage-fusion events of 17 RC cases.

**Results:**

Complete RCs without loss of euchromatin by a fusion of subtelomeric or telomeric regions were noted in a RC14 and a RC20. Incomplete RCs with intrachromosomal copy-number variants were noted in 15 cases, including a RC3, a RC4, 4 RC13s, a RC14, 3 RC18s, a RC21, 3 RC22s, and an RCY. srGS defined breakage-fusion sequences in single-copy sequences, and lrGS mapped subtelomeric and pericentric repetitive sequences using the telomere-to-telomere reference genome. The breakage-fusion sequences revealed ring formation mechanisms by intrastrand nonhomology end joining in 5 RCs, microhomology-mediated end joining in 8 RCs, and microhomology-mediated break-induced replication in 4 RCs.

**Conclusion:**

This study demonstrated the analytic validity and diagnostic utility of srGS and lrGS in delineating the genomic rearrangements in RCs for better interpreting clinico-cytogenomic correlations and further analysis of RC behavior in cell cycles.

## Introduction

Constitutional ring chromosomes (RCs) are a rare chromosomal structural abnormality with an estimated newborn incidence of 1 in 50,000.^1^^,^^2^ RCs have been reported in all human chromosomes, but with higher relative frequencies for all acrocentric chromosomes (13, 14, 15, 21, and 22) and chromosomes 18 and 20.^3^^,^^4^ Patients with a RC display a spectrum of clinical phenotypes ranging from relatively normal to variable developmental and intellectual disabilities and multiple congenital anomalies. Severe growth retardation, variable intellectual disability, and reproduction failure have been suggested as “ring syndrome” for all RCs resulting from a small proportion of cellular loss due to RC instability through cell cycles.^5^, ^6^, ^7^ Other clinical findings include chromosome-specific deletion and duplication syndromes, gene-related organ and tissue defects, and predisposition to several types of cancers.^8^, ^9^, ^10^, ^11^ A recent report and compendium by the International Consortium for Human Ring Chromosomes (ICHRC) have summarized the cytogenomic approaches in analyzing RCs and possible clinico-cytogenomic correlations for RC patients reported in the literature.^2^^,^^12^

In clinical cytogenetic laboratories, karyotyping and fluorescence in situ hybridization (FISH) are routinely performed to analyze RCs and related dynamic mosaicism in patients and to detect de novo or inherited RCs in recommended follow-up parental study.^10^^,^^13^ Chromosome microarray analysis (CMA) has been used in the past decade to determine breakpoints and genomic imbalances in the RCs.^14^, ^15^, ^16^, ^17^, ^18^ Approximately 95% to 99% of the RCs are de novo and form during meiosis or early postzygotic mitosis.^13^ Cytogenetic mechanisms for RC formation have been described, including a telomeric fusion without loss of euchromatin for a complete RC and breakpoint fusions of either 1 or both short (p) and long (q) arms with loss of euchromatin for an incomplete RC. Depending on the loci of DNA double-strand breaks and the mechanisms of DNA repair, incomplete RCs with a distal deletion in only one arm, distal deletions in both arms, interstitial inverted duplication followed by a terminal deletion (inv-dup-del), and other complex rearrangements have also been described.^15^^,^^19^, ^20^, ^21^

Current cytogenomic analysis has limitations in mapping the breakage-fusion events, characterizing genomic rearrangements, and inferring ring formation mechanisms at a nucleotide-level resolution. Sanger sequencing on a long-range PCR product flanking the junction of a RC17 inferred a microhomology-mediated nonhomologous end joining (NHEJ) mechanism in its RC formation.^11^ Several studies have used short-read genomic sequencing (srGS) to characterize breakage-fusion sequences in 3 cases of RC18 and 1 case of RC9.^22^, ^23^, ^24^ However, identifying breakpoints within subtelomeric and pericentric regions and short arms of acrocentric chromosomes poses significant challenges due to the largely unresolved nucleotide sequences in the GRCh37/hg19 and GRCh38/hg38 genome assemblies. Recently, the telomere-to-telomere (T2T) consortium has generated the first complete human genome assembly, including the acrocentric chromosomes.^25^ Long-read genomic sequencing (lrGS) has been used for detecting structural variants in ring chromosomes, Robertsonian translocations, and complex structural variants.^26^ The ICHRC has organized a collaborative study to use both srGS and lrGS in 17 cases of RCs. This study aims to evaluate the analytical validity and diagnostic utility in delineating breakage-fusion sequences and genomic rearrangements and inferring ring formation mechanisms and functional implications.

## Materials And Methods

### Collected cases of RCs

Archived DNA samples from a total of 17 deidentified patients carrying a RC were collected, including 1 case each for RC3, RC4, RC20, RC21, and RCY, 2 cases for RC14, 3 instances each for RC18 and RC22, and 4 cases for RC13.

Karyotyping, FISH, and CMA were carried out according to the standard cytogenomic procedures by participating laboratories, and all instances were previously published^15^, ^16^, ^17^, ^18^ except for 1 case each of RC13, RC20, and RCY. The genome coordinates of GRCh37/hg19 from the available CMA data were converted to T2T-CHM13v2.0 using the LiftOver tool in the UCSC Genome Browser. Both srGS and lrGS were performed at Yale Center for Genome Analysis (YCGA).

### srGS and data analysis

For each case, 500 to 1500 ng of DNA was used for library preparation using the xGen DNA library EZ prep kit (Integrated DNA Technologies, Inc) following the manufacturer’s protocol. Libraries were sequenced at YCGA on the Illumina NovaSeq platform using a 2 × 150 bp paired-end read configuration (Illumina, Inc). Reads from srGS were aligned to the reference genome T2T-CHM13v2.0 using BWA-MEM. Copy-number variants (CNVs) and structural variants (SVs) analysis were performed using Dragen Germline (v4.2.4) with Manta used for SV calling.

### lrGS and data analysis

For each case, 1 to 5 μg of DNA was sheared to 15 to 20 kb using the Megarupter, followed by DNA repair ligation of PacBio adaptors and nuclease treatment using SMRTbell prep kit 3.0 following the manufacturer’s protocol. The PacBio SMRT libraries were sequenced at YCGA on a PacBio Revio System, utilizing Revio 24M SMRT Cells with their increased density of 25 million zero-mode waveguides (PacBio, Inc). Sequencing reads from lrGS were mapped to the T2T-CHM13v2.0 genome using pbmm2 (SMRT link v12.0.0). Mosdepth was used to calculate the coverage of the aligned BAM files.^27^ SVs were called using pbsv, and CNVs were detected using HiFiCNV. HiPhase was used to jointly phase small variants and SVs that were generated from DeepVariant and pbsv.^28^

### Mapping breakpoints and fusion sequences and inferring ring formation mechanisms

CNVs identified from the srGS and lrGS were cross-examined with CMA data. Breakage-fusion sequences were manually inspected using Integrative Genomics Viewer within a ±1 kb region upstream and downstream of the starting chromosome position of the deleted CNV. For cases showing distal and segmental deletion and duplication, the beginning and end of the duplication and the start of the deletion were manually inspected. Subsequently, soft-clipped reads at the junction region were subjected to BLAT against the T2T-CHM13 reference genome to identify the chromosomal rearrangement(s) in the RC. The features of breakage-fusion sequences were further annotated by the UCSC Repeat Browser, including RepeatMasker, SEDEF Segmental Duplications, Simple Repeats, and WM+SDust. The description of sequencing results follows the ISCN 2024 (An International System for Human Cytogenomic Nomenclature 2024).^29^

Ring formation mechanisms were inferred from breakage-fusion sequences and genomic rearrangements in the RCs, indicative of molecular mechanisms of DNA repair.^21^^,^^30^^,^^31^ The srGS and lrGS provided a nucleotide-level resolution of the breakage-fusion sequences and precise orientation of CNVs, allowing the recognition of blunt ends, microhomology sequences, and insertions between the breakpoints to guide the inference of ring formation mechanisms.

## Results

A genomic sequencing (GS) number linked with the specific RC was designated for the 17 collected cases. srGS was performed in 14 cases, and lrGS was performed in all 17 cases. Brief clinical features, karyotyping results, and related references are summarized in Table 1. The results of CNVs from CMA, srGS, and lrGS, features of breakage-fusion sequences, and repairing mechanisms are summarized in Supplemental Table 1. Considering each technique’s resolution, acceptable concordance was observed in detecting CNVs in these RCs by CMA, srGS, and lrGS. One limitation from the current CMA was unable to define short-arm CNVs for acrocentric chromosomes.

**Table 1:** Brief clinical summary, karyotyping results, and references for the 17 RC cases

Case No.	Age	Gender	Brief Clinical Summary	Karyotyping Results	Refs.
GS1-RC3	10 y	Male	IUGR, SS, facial dysmorphisms, crossed renal ectopia, moderate DD/ID.	46,XY,r(3)(p26.1q29)	^15^
GS2-RC4	11 y	Female	IUGR, SS, microcephaly, facial dysmorphism, neuro-psychomotor delay, and ID.	46,XX,r(4)(p16.3q35.2)	^15^
GS3-RC13	3 y	Male	Preterm delivery, IUGR, microcephaly, facial dysmorphism, peno-scrotal inversion, scrotal hypoplasia, prominent and large halluces, renal ectopia, hypotonia, and severe neuro-psychomotor delay.	46,XY,r(13)(p11.2q33.1)	^15^
GS4-RC13	1 y	Male	IUGR, microsomia, microcephaly, facial dysmorphism, thoraco-lombar scoliosis, right feet pos-axial polydactyly, hypotonia, and neuro-psychomotor delay.	46,XY,r(13)(p13q34)	^15^
GS5-RC13	3 y	Male	Microcephaly, brachycephaly, hypotonia, strabismus, hypertelorism, epicanthus, prominent nose, otitis, leukopenia, cerebellar hypoplasia, speech delay, and neuro-psychomotor delay.	46,XY,r(13)(p13q34)	N.R
GS6-RC14	23 y	Male	Facial dysmorphism, upper anus implantation and decreased subcutaneous tissue in gluteal region, hypotrophy of the lower limbs, club feet, protrusion of the calcaneus, and mild ID.	46,XY,r(14)(p13q32.33)	^15^
GS7-RC18	11 y	Female	SS, facial dysmorphism, hepatomegaly/splenomegaly, IgA immunodeficiency, chronic hepatitis, renal tubular acidosis, neuro-psychomotor delay and ID.	46,XX,r(18)(p11.1q23)	^15^
GS8-RC18	5 y	Female	SS, facial dysmorphism, clinodactyly of 5th fingers, gastro-esophageal reflux and atrial/tricuspid cardiac defects corrected by surgery.	46,XX,r(18)(p11.32q21.33)	^15^
GS9-RC22	24 y	Male	Hypotonia, facial dysmorphism, 2 café-au-lait spots, chest asymmetry, dorso-lombar scoliosis, C2-C3 vertebral fusion, neuro-psychomotor delay and ID.	46,XY,r(22)(p11.2q13.2)	^15^
GS10-RC22	2 y	Female	Hypotonia, mild motor DD. irregular teeth, large nose, small mouth, a small supernumerary nipple at left, proximal implantation of halluces.	46,XX,r(22)(p12q13.2)	^15^
GS11-RC18	7 y	Female	Severe DD, gross facial dysmorphism.	mos 46,XX,r(18)(q11.1q22.3)[30]dn/46,XX,psu idic(18)(p11.2)[25]	^17^
GS12-RC13	1 m	Female	IUGR, mild dysmorphic features, and TOF/PA/VSD.	46,XX,r(13)(p11q33.2)[88]/45,XX,-13[7]/46,XX,dic r(13)[5]	^16^
GS13-RC20	17 y	Female	Follow-up from age 17y to 28y for Intractable refractory epilepsy.	mos 46,XX,r(20)(p13q13.33)[41]/46,XX[59]	N.R
GS14-RCY	11 y	Male	SS.	46,X,r(Y)(p11.2q11.221)[16]/45,X[4]	N.R
GS15-RC14	6 y	Male	Profound ID, language deficiency, severe hyperactivity, mild orthopedic deformity, facial dysmorphic features, and seizure disorders.	46,XY,r(14)(p11.2q32.33)[25]	^18^
GS16-RC21	3 y	Male	High palate, down-slanted eyes, a shield-shaped chest, strabismus, and a bulbous nose.	46,XY,r(21)(p13q22.2)[21]	^18^
GS17-RC22	4.5 y	Female	Mild dysmorphic features, such as wide spaced eyes, flat nasal bridge, brachydactyly in 5th finger and toes, DD with major speech delay, and motor clumsiness.	mos 46,XX,r(22)(q11.21q13.32)inv(22)(p13q11.2)[26]/45,XX,-22[3]	^18^

*DD*, developmental delay; *HT*, hypotonia; *ID*, intellectual deficiency; *IUGR*, intrauterine growth retardation; *m*, month; *NA*, not applied; *N.R.*, not reported; *PA*, pulmonary atresia; *SS*, short stature; *TOF*, tetralogy of Fallot; *VSD*, ventricular septal defect; *y*, year.

As summarized in Table 2, the srGS had a sequencing depth ranged from 26 to 42, with an average coverage of approximately 32; the HiFi lrGS had a read length ranged from 8.3 to 11.9 kb with an average of 9.9 kb, and the sequencing depth ranged from 6 to 25 with an average of approximately 17. Among the 14 cases submitted to srGS, soft-clipped sequence reads implied the breakage-fusion sequences at the single-nucleotide level in 5 instances, GS2-RC4, GS4-RC13, GS5-RC13, GS7-RC18, and GS8-RC18; and then low-coverage lrGS confirmed the sequences. The lrGS defined the breakage-fusion sequences in the remaining 12 RCs. The results from the lrGS data and the nomenclature following ISCN2024 for these 17 cases of RCs are shown in Supplemental Files 1-4 and Supplemental Table 1 and presented based on the types of genomic rearrangements as follows:

**Table 2:** Sequencing depth by srGS and lrGS and median length by lrGS

Case No.	srGS Read Depth	lrGS HiFi Read Length (Median, bp)	lrGS Read Depth
GS1-RC3	30.19	11,718	21.08
GS2-RC4	32.61	10,548	6.62
GS3-RC13	26.67	8,316	25.60
GS4-RC13	34.33	11,950	12.37
GS5-RC13	31.90	11,950	12.11
GS6-RC14	27.96	12,125	19.72
GS7-RC18	34.18	10,548	7.44
GS8-RC18	30.85	10,800	6.78
GS9-RC22	32.68	11,921	24.84
GS10-RC22	31.99	10,958	25.31
GS11-RC18	42.42	5,910	18.21
GS12-RC13	39.36	9,362	18.32
GS13-RC20	26.53	8,797	15.67
GS14-RCY	27.62	10,462	20.95
GS15-RC14	NA	6,917	6.19
GS16-RC21	NA	7,255	19.63
GS17-RC22	NA	8,919	24.65
Average:	32.09	9,909	16.79

*NA*, not applied.

### Complete RCs by subtelomeric or telomeric fusion

**Figure 1:**
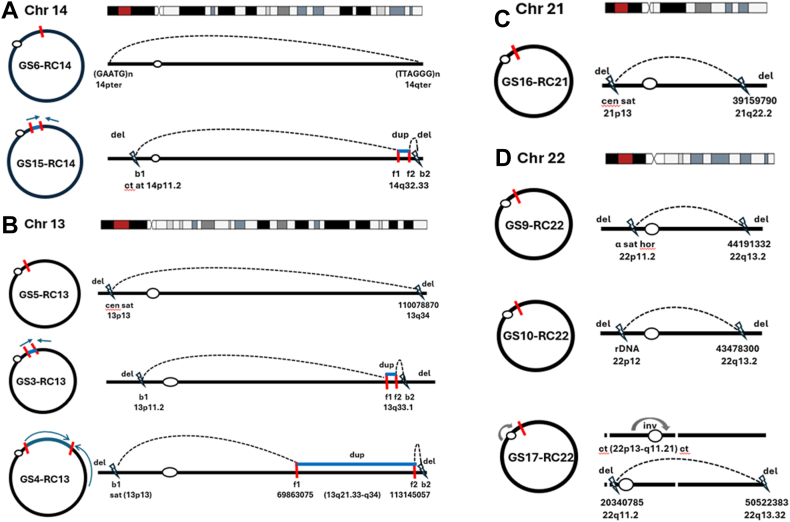
**Sequence results on acrocentric ring chromosomes.** A. Two cases of RC14 showing a complete RC14 by a fusion of subtelomeric repeats at 14pter and telomeric repeats at 14qter in GS6-RC14 and an interstitial inverted duplication and distal deletions in GS15-RC14. B. Three cases of RC13 showing distal deletions were detected in GS5-RC13, and interstitial inverted duplications and distal deletions were detected in GS3-RC13 and GS4-RC13. C. Distal deletions in GS16-RC21. D. Three cases of RC22 showing distal deletions in GS9-RC22, concurrent RC22 and a deletion at 22q11.2 in GS10-RC22, and a pericentric inversion and distal deletions in GS17-RC22. α sat hor, α-satellite higher-order repeat; b1, breakpoint 1; b2, breakpoint 2; ct, centromeric transition sequences; cen sat, centromeric satellite; del, deletion; dup, duplication; f1, fusion 1; f2, fusion 2; hum sat, human satellite; rDNA, ribosomal DNA.

CMA detected a normal pattern for GS6-RC14 and GS13-RC20; neither srGS nor lrGS detected CNVs. For GS6-RC14, lrGS read into the telomeric region (TTAGGG)n repeats at 14q32.33 and extended into the terminal region (GAATG)n repeats at 14p13. The proximal sequence (TTAGGG)n is typical human telomere repeat, but the (GAATG)n repeats along with the distal sequence are mapped to the short-arm subtelomeric region at 14p13, as well as other acrocentric chromosomes (Figure 1A, Supplemental File 1A). After the ISCN2024, this RC14 was designated as: r(14)(p13q32.33). For GS13-RC20, the sequencing reads extended to the terminal regions at 20p13 and 20q13.33 (Supplemental File 1B); this RC20 was designated as: r(20)(p13q13.33). It is anticipated that breakage-fusion could have occurred as an end-joining event within the subtelomeric or telomeric regions to form these complete RC14 and RC20. lrGS could clarify the repetitive nature of the subtelomeric and telomeric sequences for complete RCs.

### Incomplete RCs by 1-arm breakage followed by telomeric or centromeric fusion

Three cases showed a distal deletion in 1 arm and a direct fusion with telomeric variant repeats (TVRs) or subtelomeric sequences at the opposite arm to form an incomplete RC.

**Figure 2:**
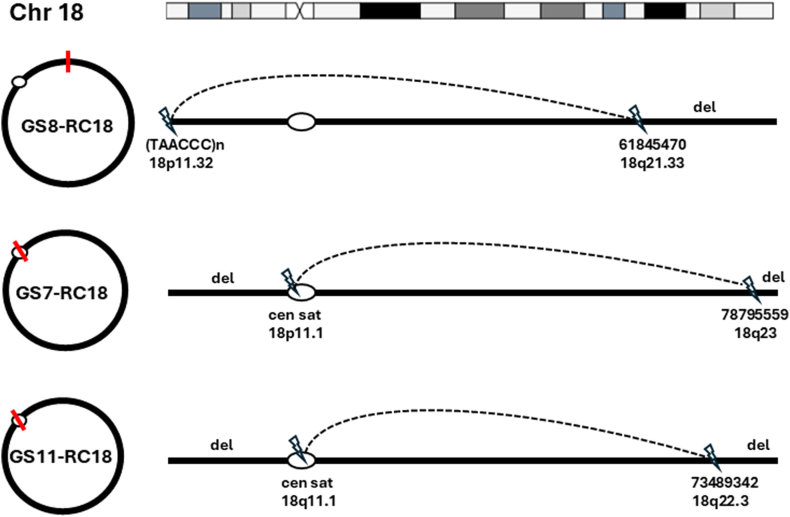
**Sequence results in 3 cases of RC18.** A blunt-end fusion of short-arm telomeric variant repeats with a breakpoint at 18q21.22 is shown in GS8-RC18, microhomology-mediated fusions of centromeric satellite sequences (cen sat) at 18p11.1 or 18q11.1 with a breakpoint at 18q23 and 18q22.3 are shown in GS7-RC18 and GS11-RC18, respectively. cen sat, centromeric satellite; del, deletion; dup, duplication.

For GS1-RC3, lrGS detected a 6.01 megabase (Mb) distal deletion of 3pter-p26.1 with the breakpoint at 3p26.1 joining the TVR (TCAGGG)n at approximately 3.6 kilobase (kb) from the distal end of 3q29 mediated by a microhomology sequence “TCA”; the breakpoint at 3p26.1 is in intron 2 of the *AC087857* gene for a long noncoding RNA (lncRNA) (Supplemental File 2A). For GS2-RC4, lrGS detected a 1.86 Mb distal deletion of 4pter-p16.3 with a breakpoint at 4p16.3 joining a subtelomeric sequence at approximately 24.3 kb from the distal end of 4q35.2 mediated by a microhomology sequence “CTGC”; the breakpoint at 4q35.2 is in intron 1 of the *DUX4-1* gene (Supplemental File 2B). For GS8-RC18, lrGS detected an 18.7 Mb distal deletion of 18q21.33-qter with a breakpoint at 18q21.33 joining the TVR (TAACCC)n at approximately 55.6 kb from the 18pter most likely by a direct end-joining event; the breakpoint at 18q21.33 is in intron 3 of the *AC105094-2* gene, a lncRNA and contains LIME5 repeats (LINE class L1 subclass) (Figure 2, Supplemental File 2C).

Fusions of centromeric active α-satellite sequence with a distal deletion breakpoint in the long arm were detected in 2 cases of RC18. In GS7-RC18, lrGS detected a 17.85-Mb distal deletion of 18pter-p11.1 and a 1.8-Mb distal deletion of 18q23-qter with the breakpoint at 18q23 joining short-arm centromeric satellite sequence (S2C18H1L) at 18p11.1 by a microhomology sequence “CTTTT” (Figure 2, Supplemental File 2D). In GS11-RC18, lrGS detected an 18.5-Mb distal deletion of 18pter-q11.1 and a 7.1-Mb distal deletion of 18q22.3-qter with the breakpoint at 18q22.3 joining the long arm centromeric satellite sequence (S2C18H1L) at 18q11.1 by a microhomology sequence “CACAC” (Figure 2, Supplemental File 2E). These 2 RC18s showed a loss of 18p, predicting clinical manifestations of 18p deletion syndrome (OMIM 146390).

### Incomplete RCs by direct breakage-fusion at 2 arms

Four RCs presented breaks and terminal deletions in both chromosome arms.

In GS5-RC13, lrGS detected a 5.35-Mb distal deletion of 13pter-p13 and a 3.49-Mb distal deletion of 13q34-qter with a fusion of the breakpoints at 13p13 and 13q34 mediated by a microhomology sequence “CT.” The breakpoint sequence at 13p13 belongs to the other centromeric satellite sequence (censat_13_7) with numerous matches in the short arms of other acrocentric chromosomes (14, 15, 21, and 22) (Figure 1B, Supplemental File 3A).

In GS16-RC21, lrGS detected a 2.71-Mb distal deletion of 21pter-21p13 and a 5.93-Mb distal deletion of 21q22.2-qter with a fusion of the breakpoints at 21p13 and 21q22.2 mediated by a microhomology sequence “C.” The breakpoint at 21p13 belongs to the other centromeric satellite sequence (censat_21_16) with numerous matches for the short arms of acrocentric chromosomes 22, 14, 15, and 13, and the distal breakpoint at 21q22.2 is in intron 1 of the *DSCAM* gene (Figure 1C, Supplemental File 3B).

In GS9-RC22, lrGS detected a 10.07-Mb distal deletion of 22pter-p11.2 and a 7.13-Mb distal deletion of 22q13.2-qter with a fusion of the breakpoints at 22p11.2 and 22q13.2 mediated by a microhomology sequence “AAA.” The breakpoint sequence at 22p11.2 is in the centromeric inactive α-satellite higher-order repeat (hor_22_6, S6C22H2-B) with multiple copies at 22p, and the breakpoint at 22q13.2 is in intron 8 of the *EFCAB6* gene (Figure 1D, Supplemental File 3C).

In GS14-RCY, lrGS detected an 8.46 kb distal deletion of Ypter-Yp11.2 and a 45.38-Mb distal deletion of Yq11.221-qter. The breakage-fusion sequence of Yp11.2 and Yq11.221 showed end joining with an insertion of 30 bp. These 2 breakpoints are LTR repeats (ERV1 subclass), and the breakpoint at Yp11.2 is in intron 1 of the *AL672277* gene (Supplemental File 3D).

### Incomplete RCs with complex rearrangements

Six incomplete RCs presented greater complexity in their rearrangements.

In GS3-RC13, lrGS detected a 10.41 Mb distal deletion of 13pter-p11.2, a 1.461-Mb duplication at 13q33.1, and a 10.79-Mb distal deletion of 13q33.1-qter. This RC13 was formed via a 2-break 2-fusion process to present 2 terminal deletions and an inverted duplication with a spacer. To form this RC13, the proximal breakpoint 1 (b1) at 13p11.2 joined with a proximal locus at 13q33.1 for a fusion 1 (f1) by a microhomology sequence “GTTT,” whereas the distal breakpoint 2 (b2) folded to a distal locus at 13q33.1 for a fusion 2 (f2) by a microhomology sequence “TGGGA,” which forms the inverted duplication with a 6.9 kb spacer in between (Figure 1B, Supplemental File 4A). The breakpoint 1 at 13p11.2 is in a LIM1 repeat (LINE class and L1 subclass), the breakpoint 2 at 13q33.1 is in a Tigger2 repeat (TcMar-Tigger subclass), the proximal locus at 13q33.1 is in intron 1 of the *FGF14* gene, and the distal locus at 13q33.1 is in a MLT1E2 repeat (LTR class and ERVL-MaLR subclass). The *FGF14* gene at 13q33.1 encodes fibroblast growth factor-14, which is highly expressed in brain tissue. Dysfunction of *FGF14* has been associated with autosomal dominant Spinocerebellar Ataxia 27A (OMIM 193003) and late-onset Spinocerebellar Ataxia 27B (OMIM 620174). The b1-f1 junction might potentially disrupt the *FGF14* function and explain the severe neurological and psychomotor delay manifestation in this patient.

In GS4-RC13, lrGS detected a 4.75-Mb distal deletion of 13pter-p13, a 43.28-Mb duplication of 13q21.33q34, and a 417-kb distal deletion at 13q34. This RC13 was formed using the 2-break 2-fusion process with a fusion 1 (f1) joining the proximal breakpoint 1 (b1) at 13p13 with a proximal locus at 13q21.33 via a microhomology sequence “TAG” and a fusion 2 (f2) joining the distal breakpoint 2 (b2) at 13q34 with a distal locus at 13q34 via a blunt end (Figure 1B, Supplemental File 4B). The proximal breakpoint at 13p13 is at human satellite 1A repeat (hsat1A-13_1) with multiple copies at 13p13, and the distal locus at 13q34 is in a LIMD1 repeat (LINE class and L1 subclass).

In GS15-RC14, lrGS detected a 5.79-Mb distal deletion of 14pter-p11.2, a 1.75-Mb duplication at 14q32.33, and a 1-Mb distal deletion of 14q32.33-qter. This RC14 was formed using the 2-break 2-fusion process with a fusion 1 (f1) joining the proximal breakpoint 1 (b1) at 14p11.2 with a proximal locus at 14q32.33 by a 3-bp insertion and a fusion 2 (f2) joining the distal breakpoint 2 (b2) at 14q32.33 with a distal locus at 14q32.33 mediated by a homology sequence “CTT” (Figure 1A, Supplemental File 4C). The proximal breakpoint 1 is in a centromeric transition region (ct_14_35) and the proximal locus at 14q32.33 is in a HAL1 repeat (LINE class and LI subclass), the distal locus at 14q32.33 is in exon 1 of the *IGHD* gene, and the distal breakpoint 2 at 14q32.33 is in a MLT1C repeat (LTR class and ERVL-MaLR subclass). The breakpoint 2 at 14q32.33 could potentially disrupt the *IGHD* gene function (OMIM ∗147170).

In GS10-RC22, lrGS detected a 1.44 Mb interstitial deletion at 22q11.2, a 5.63-Mb deletion at 22pter-p12, and a 7.85-Mb distal deletion at 22q13.2-qter. The deletion at 22q11.2, which includes *HIRA* and *TBX1,* is a known microdeletion caused by low-copy repeat-induced nonallelic homologous recombination associated with DiGeorge syndrome (OMIM#188400). The fusion for RC22 occurred by joining the rDNA sequence (rDNA_22_1) at 22p12 with a breakpoint within intron 1 of the *PACSIN2* gene at 22q13.2 by a 7-bp insertion (Figure 1D, Supplemental File 4D).

In GS17-RC22, CNV analysis noted an 802-kb distal deletion of 22q13.32-qter. Within the same phase block, lrGS detected a pericentric inversion and a fusion of distal regions for a ring formation. The inversion between 22p13 (chr22:53014) and 22q11.21 (chr22:20358565 at intron 5 of the *ARVCF* gene) involves a centromeric transition region (ct_22_1) at 22p13 and a centromeric transition region at 22q11.21 (ct_22_103). Then a breakage-fusion event occurred between 22q11.2 (chr22:20340785 at intron 3 of the *COMT* gene, approximately 17.8 kb away from chr22:20358564 but within the same centromeric transition region, ct_22_103) and 22q13.32 (chr22:50522383 at intron 12 of the *TTLL8* gene) mediated by a microhomology sequence “GG” (Figure 1D, Supplemental File 4E).

**Figure 3:**
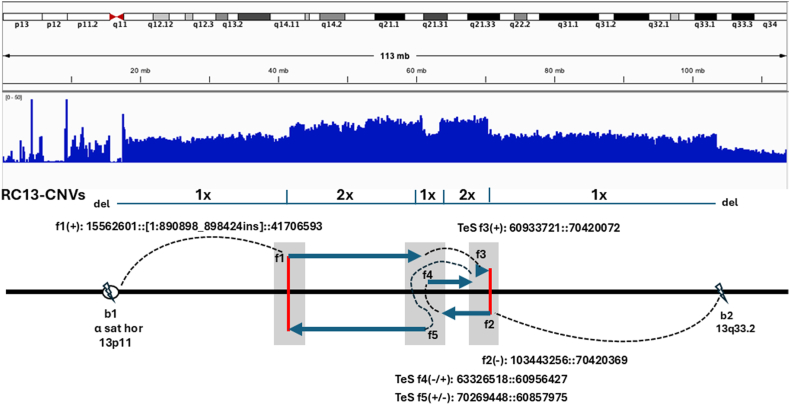
**Sequencing results on an RC13.** The distal breakpoints fold back to intrastrand loci for microhomology-mediated break-induced replication (MMBIR) with template switch (TeS) during replication. Shade areas for replication start points at f1 and f2 and TeS at f3, f4, and f5. The observed interstitial 2 duplications and an intervening spacer are likely caused by a fork-stall and template switch during replication repair. b1, breakpoint 1; b2, breakpoint 2; del, deletion; dup, duplication; f1, fusion 1; f2, fusion 2; f3, fusion 3; f4, fusion 4; f5, fusion 5; TeS, template switch.

In GS12-RC13, results from CMA, srGS, and lrGS identified 2 interstitial duplications with a spacer at 13q14.11-q21.33 and distal deletions at 13p11 and 13q33.2. As depicted in Figure 3 and Supplemental File 4F, the lrGS and srGS results showed 2 breaks and 5 fusion sequences with template switches (TeS) mediated by microhomology sequences for a complex rearrangement. The proximal breakpoint 1 (b1) at 13p11.1 (chr13:15562601, active α-satellite higher-order repeat, hor_13_3, S2C13/21H1L) inserted a 474-bp sequence from 1p36.33 (chr1:890898-890424, including intron 6-exon 7-intron 7 of the *ATAD3C* gene) then joining at 13q14.11 (chr13:41706593, L1ME4a, LINE/L1) as fusion 1 (f1) in the forward strand (+). The distal breakpoint 2 (b2) at 13q33.2 (chr13:103443256, Arthur 1, hAT-Tip100) fused to 13q21.33 (chr13:70420369, MLT1G1, LTR/ERVL-MaLR) as fusion 2 (f2) in the reverse strand (-). One replication TeS was noted as fusion 3 (f3) for the forward strand, and 2 TeS were noted as fusion 4 and 5 (f4, f5) for the reverse strand. The TeS resulted in 2 duplication segments with a spacer in 13q14.11-13q21.3 in the RC13. Phasing analysis confirmed that the rearrangements within the region were in the same haplotype block, indicating an intra-chromosome rearrangement with the RC13.

### Breakage-fusion sequences and ring formation mechanisms

Sequencing results showed that 14 out of 17 cases (82%) contained certain breakage-fusion sequences uniquely available in the T2T-CHM13 v2.0 assembly compared with the GRCh38/hg38 and GRCh37/hg19 reference assemblies. The lrGS results and the T2T reference genome were required to dissect the breakage-fusion in repetitive sequences of the telomeric/subtelomeric and pericentric regions, especially for acrocentric RCs involving the pericentric satellite high-order repeats (hor), rDNA sequences, centromeric transition (ct) sequences (Figure 1).

Other interspersed repeat sequences were identified in the breakage-fusion loci in 9 out of 17 RC cases (53%). The SINE elements of AluSc8 and AluY, the LINE elements with several subclasses of LIM1, LIME5, L1MD1, LIME4a, and HAL1, the LTR elements with particularly those subclasses of the ERVL-MaLR (MLT1E2, MLT1G1, and MLT1C) and ERV1 (LTR9 and LTR30), and the DNA transposons of Tigger2 and Authur1 were observed at the breakage-fusion loci, suggesting a diverse array of repetitive sequences contributing to the structural variations (Supplemental Table 1). Breakage-fusion sequences in gene introns and an exon were noted in 10 RC cases and one RC case, respectively, suggesting that gene disruption and potential functional implications should be considered (Supplemental Table 1).

Several mechanisms of ring formation were inferred from breakage-fusion sequences. As shown in Supplemental Table 1, RC formation by nonhomologous end joining was indicated in five RC cases (29%); microhomology-mediated end joining was indicated in eight RC cases (47%), and microhomology-mediated break-induced replication (MMBIR) with terminal deletions and an interstitial duplication was noted in 4 RC cases (24%). The complex rearrangements GS12-RC13 most likely resulted from fork stalling and template switching (FoSTeS) during MMBIR.

## Discussion

The results from this case series, including rare and recurrent RCs, identified considerable cytogenomic heterogeneity and provided new insights into the breakage-fusion processes and formation mechanisms of RCs. Breakpoint analysis suggested that chromosome regions enriched in repetitive elements, such as the telomeric/subtelomeric and pericentric regions, could play a significant role in chromosome instability and structural variation. Repetitive elements pose a challenge in alignment using srGS, as the short repeat sequences can map to multiple genomic loci, resulting in their misclassification and misinterpretation.^32^ An exemplar case illustrated in this study is GS1-RC3, whose 3q subtelomeric region at the junction with the breakpoint at 3p could not be located on chromosome 3 by srGS, but it was successfully mapped to the 3q subtelomeric region using lrGS. In addition, the lrGS analysis revealed that the short-arm sequences of acrocentric chromosomes could be mapped to multiple loci within the targeted chromosome and to different acrocentric chromosomes with similar sequence identity in the T2T-CHM13 reference genome. This observation is likely due to pseudo-homologous regions, which implicate recombination between non-homologous sequences on these acrocentric chromosomes, as suggested by a recent study.^33^ Although the T2T-CHM13 reference genome provides a comprehensive resolution of the genomic structure of the acrocentric chromosomes and confirms their strong similarity, it does not provide information on how the sequence of these short arms varies among individuals in the human population. General considerations on mapping lrGS results to the T2T-CHM13 telomeric/subtelomeric and pericentric regions of acrocentric chromosomes in RCs include: (1) select the matched region with the highest identity on the targeted chromosome and document other matched regions on the same or other acrocentric chromosomes; (2) map a short sequence of 0.2 to 1 kb near the breakpoint to a higher identity to anchor the breakpoint; (3) arbitrarily determine the most proximal mapped sequence as the breakpoint. This approach has been used in the mapping of the TVRs, centromeric satellite sequence (cen sat), active centromeric α satellite high-order repeats (α sat hor), rDNA sequences, and centromeric transition (ct) sequences from the 10 cases of acrocentric RCs (Figures 1 and 3).

Current cytogenomic analysis of RCs relies on routine karyotyping and FISH for cell-based assessment of RC structure and dynamic mosaicism and CMA for CNVs; clinico-cytogenomic correlations for RC patients could be explained based on detected RC behavior and deletions and duplications of dosage-sensitive regions.^2^^,^^34^ In this case series of RCs, GS10-RC22 may have a RC22 and an interstitial deletion at 22q11.2 likely in its homolog, whereas GS17-RC22 could have undergone a pericentric inversion before ring formation. These complex structural details, discernible only through lrGS, underscore the necessity of using lrGS to perform phasing analysis and accurately resolve complex chromosomal architectures. Furthermore, FISH or fiber-FISH analysis using targeted probes could visualize these compound or complex rearrangements in chromosomal or chromatin level.^35^

As demonstrated in this study, lrGS enabled higher resolution and more accurate delineation of complex structural rearrangements within the RCs by mapping breakage-fusion sequences and CNV patterns in telomeric/subtelomeric and pericentric regions with the T2T reference genome. For example, the GS7-RC18 and GS11-RC18 showed breakpoints in the proximal 18p11.1 and distal 18q11.1 centromeric satellite sequences, respectively (Figure 2), and chromosome mosaicism was noted in GS11-RC18. The integrity of the centromeric components may affect the stability of these RCs through cell cycles and ultimately cause dynamic mosaicism and contribute to clinical heterogeneity. As shown in GS3-RC13, GS4-RC13, GS15-RC14, and GS12-RC13 (Figures 1 and 3), the 2-break 2-fusion process has been proved to be a recurrent intrastrand repair mechanism by MMBIR to generate interstitial inverted duplications with a spacer and distal deletions (inv-dup-del) in the RCs.^24^ Unlike simple deletions, the inv-dup-del can have different effects on gene function depending on the duplication’s breakpoints and orientation and their impact on the reading frame and transcription. Inverted duplications can potentially disrupt genes at breakpoint junctions without preserving an intact copy. Thus, determining the orientation and location of duplication CNVs is essential to interpret their effects on genes and correlate the phenotypes.^36^ Identifying the inv-dup at the intronic region of the *FGF14* gene in GS3-RC13 and the exonic region of the *IGHD* gene in GS15-RC14 is crucial because it may provide valuable insights into the genetic basis of clinical manifestations in affected individuals. This finding highlights the importance of detecting and characterizing SVs, such as inversions, to explain and predict patient phenotypes in future research. Similar to findings from GS on balanced chromosomal rearrangements and complex insertions,^37^^,^^38^ our lrGS analysis showed that RCs can form through several DNA repair mechanisms including NHEJ, microhomology-mediated end joining, and MMBIR (FoSTeS). The proportion of these mechanisms in our cases was comparable to that reported for inversions and translocations.^37^ However, lrGS has the advantage to resolve additional complex features, such as inverted duplications and a pericentric inversion at the breakpoints and RC formation mediated by higher-order repeat sequences.

A recent online database of RCs included approximately 1900 reported cases of constitutional RCs globally^4^; the extremely low incidence of constitutional RCs has resulted in almost all being single-case reports and very few systematic analyses of this unique chromosomal abnormality in a case series. This series of 17 cases of RCs of chromosomes 3, 4, 13, 14, 18, 20, 21, 22, and Y was considered a systematic effort from the ICHRC to validate and utilize GS for RCs. Further collaborative efforts will be pursued to include more RC cases for full coverage of all autosomes and sex chromosomes. One limitation of this study is the inability to interrogate the role of dynamic mosaicism and secondary events for RC variants by this DNA-based approach on archived DNA specimens. Integrated cytogenetic and genomic analyses should be recommended for all cases of RCs.

The results from this case series of RCs highlighted the importance of GS in resolving chromosomal structural rearrangements and ruling out other genomic variants. The analysis of srGS results in the current diagnostic pipeline detected no other pathogenic gene variants, indicating the RC abnormality as the sole cause for these patients’ phenotypes. For chromosomal rearrangements involving nonrepetitive sequences, srGS can define the CNVs and breakage-fusion sequences. For chromosomal rearrangements involving telomeric/subtelomeric and pericentromeric regions, lrGS should be the method to determine the CNVs, the breakage-fusion rearrangements, and potentially the aberrant methylation patterns. Furthermore, the recently updated ISCN2024 is straightforward on the description of sequencing results of simple RCs but could be a challenge for complex RCs involving multiple breakage-fusion events and CNVs in different orientations.

In conclusion, the lrGS analysis and the T2T reference genome enabled the detection of breakage-fusion sequences to resolve genomic rearrangements and infer formation mechanisms in the RCs. Identifying gene content in the deleted and duplicated regions and structural rearrangements for possible gene disruption can be essential to explain or predict phenotypes resulting from these RCs. A future collaborative study on a large case series of RCs will be pursued to understand various mechanisms of RC formation and their functional implications on RC stability and behavior. Development and updating of cytogenomic variations in RC databases are also needed to aid interpretation of this unique chromosomal abnormality. Expert recommendations and guidelines should be developed to adopt GS into cytogenomic practice for RCs and other chromosomal structural abnormalities.

## Conflict of Interest

All authors declare no conflicts of interests.
