# Supplementary material for: Unravelling ring chromosome structures and formation mechanisms by short-read and long-read genomic sequencing
Source: Genet Med Open. 2025 Nov 19;4:103475. doi: 10.1016/j.gimo.2025.103475 (PMC13207348; doi:10.1016/j.gimo.2025.103475)

**Supplementary File 1. Complete RCs by subtelomeric or telomeric fusion.**

**A. lrGS for GS6-RC14.**

**B. lrGS for GS13-RC20.**

**Supplementary File 1A. GS6-RC14**

IGV view of the sequence reads on chromosome 14 for GS6-RC14. **A.** CNV analysis revealed no consistent soft-clipped regions at 14p13 or 14q32.33. **B.** A closer examination of the 14q32.33 region revealed several soft-clipped reads that did not align to the same chromosomal location at 14q32.33. To further investigate, de novo assembly was performed to obtain the sequence at 14q32.33. As illustrated in the following de novo assembled sequence, the proximal sequence along with the (TTAGGG)n repeats are specific for the terminal region at 14q32.33 (chr14: 101159020-101161492, 98.2% identity), and the (GAATG)n repeats along with distal sequence could be mapped to the subtelomeric region at 14p13 (chr14:972727-1244018, 95.1% identity) but also mapped to the short arm subtelomeric regions of chromosomes 13, 15, 21, and 22 (92%-99.2% identity).


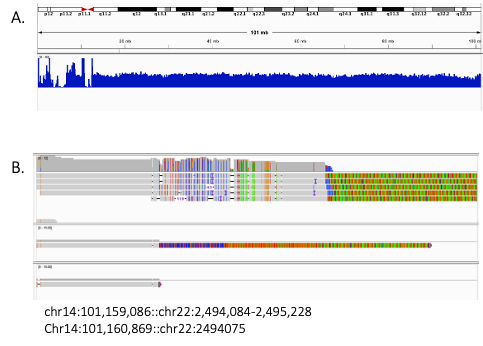


Below sequence is assembled by HiFIasm and BLAT Search Results showed: 1691-bp best match to chr14:101159020-101161492 at 14q32.33 (98.2% identity)::77-bp with 16 matches to chr14:972994-1097461 at 14p13 (91.5%-94.3% identity)::1137-bp best match to chr14:972727-1244018 at 14p13 (95.1% identity, also to 22p, 13p, 21p, and 15p with 92.4%-99.3% identity)

**ISCN:** seq[T2T] r(14)(p13q32.33) g.(pter)_~972727del::~101161492_(qter)del

CCCCATCTCTGCGCCTGCGCGGCCGCGCCTCCCCATCTCTGCGCCTGCGCGGCCGCGCCGCGCCTCTCTGCGCCTGCGCGGCCGCGCCTCCCCATCTCTGCGCCTGCGCGGCCGCGCCTCCCCATCTCTGCGCCTGCGCGGCCGCGCCGCGCCTCTCTGCGCCTGCGCGGCCGCGCCGCGCCTCTCTGCGCCTGCGCGGCCGCGCCTCCCCATCTCTGCGCCTGCGCGGCCGCGCCGCGCCTCTCTGCGCCTGCGCGGCCGCGCCTCCCCATCTCTGCGCCTGCGCGGCCGCGCCTCCCCATCTCTGCGCCTGCGCGGCCGCGCCGCGCCTCTCTGCGCCTGCGCGGCCGCGCCTCCCCATCTCTGCGCCTGCGCGGCCGCGCCTCCCCATCTCTGCGCCTGCGCGGCCGCGCCGCGCCTCTCTGCGCCTGCGCGGCCGCGCCTCCCCATCTCTGCGCCTGCGCGGCCGCGCCGCGCCTCTCTGCGCCTGCGCGGCCGCGACTCCCCATCTCTGCGCCTGCGCGGCCGCGACGCGCCTCTCTGCGCCTGCGCGGCCGCGCCGCGCCGCGCCTCTCTGCGCCTGCGCGGCCGCGCCGCGCCTCTCTGCGCCTGCGCGGCCGCGCCGCGCCTCTCTGCGCCTGCGCGGCCGCGCCGCGCCTCTCTGCGCCTGCGCGGCCGCGCCGCGCCTCTCTGCGCCTGCGCGGCCGCGCCGCCTTTGCGAGGGTGGAGTTGCCTTAGGGTTAGGGTTAGGGTTAGGGTTAGGGTTAGGGTTAGGGTTAGGGGCGTTAGGGTTAGGGGCGTTAGGGTTAGGGTTAGGGTTAGGGTTAGGGTTAGGGTTAGGGTTAGGGTTAGGGTTAGGGTTAGGGTAGGGTTAGGGTTAGGGTTAGGGTTAGGGTTAGGGTTAGGGTTAGGGTTAGGGTTAGGGTTAGGGTTAGGGTAGGGTTAGGGTTAGGGTTAGGGTTAGGGTTAGGGTTAGGGGTTAGGGTTAGGGTTAGGGTTAGGGTTAGGGTTAGGGTTAGGGTTAGGGTTAGGGGTTAGGGTTAGGGTTAGGGTTAGGGTTGGGGTTGGGGTTGGGGTTGGGGTTGGGGTTGGGGTTGGGGTTGGGGTTGGGGTTAGGGGTTAGGGTTAGGGTTAGGGTTAGGGTTAGGGTTAGGGTTAGGGTTAGGGTTAGGGTTAGGGTTAGAGGGTTAGGGTTAGGGTTAGGGTTAGGGTTAGGGTTAGGGTTAGGGTTAGGGTTAGGGTTAGGGTTAGGGTTAGGGTTAGGGTTAGGGTTAGGGTTAGGGTTAGGGTTAGGGTTAGGGTTAGGGTTAGGGTAGGGTTAGGGTAGGGTTAGGGTAGGGTTAGGGTTAGGGTTAGGGTTAGGGTTAGGGTTAGGGTTAGGGTTAGGGTTAGGGTTAGGGTTAGGGTTAGGGTTAGGGTTAAGGGTTAGGGTTAAGGGTTAGGGTTAGGGTTAGGGTTAGGGTTAGGGTTAGGGTTAGGGTTAGGGGGTTAGGGTTAGGGTTAGGGTTAGGGTTAGGGTTAGGGTTAGGGTTAGGGTTAGGGTTAGGGTTAGGGTTAGGGTTAGGGTAGGGTTAGGGTTAGGGTTGGTTAGGGTTGGGTTAGGGTTAGGGTTAGGGGTTAGGGTTAGGGGGTTAGGGGTTAGGGGTAGGGTTAGGGTTAGGGTTAGGGTAGGGTTAGGGGTAGGGTTAGGGTAGGGGTAGGGTGGGTCAGGGCGGGATGGATGGAATGAAATCTATTGGATCAGAATGGAATCCAATGGAATCAACTGGAATGGAATGGAATGGAATGGAATGGAATGGAATGAAAAGGAAAGGAACAAAATGGAATCAACACGAGTGGAATGGAATGGAACGGAATGGAATTAACCCGAGTGGAATGAAATGGAATGTAATGCAATGGGATGGAGTGGAATCAACCCCAGTGGAATGGAATGGAATTGAATGGAATGGAATGGAATGGAATGGAATGGAACGGAATGGAATGGAATGGAATGGAATGGAACGGAATGGAATGGAATGGAATGAAATGGAATGGATCGGAATGGAATCCAATGGAATCAATTGGAATGAAATGGAATGGAATGGAAAGGAATGGGATGGAATGGAATAAATCCGGGTGGAGTGGAATGGAATGTAATGGAGTGTAATGTAATGGAATTTAGTGGAATGGAATGGAATCGAATGGAATGAAATGGAATGGAATGGAATGGAAAAAATCCGGGTGGAGTGGAATGGAATGTAATGGAGTGGAATGTAATGGAATTTAGTGGAATGGAATGGAATGGAATGGAATGAAATGGATTGGATCGGAAAGGAATCCAATGGAATCAAATGGAATGCAATAGAATGGAATGGAATGGAAAGGAACGAATGGAATGGAATGGTGTGGAATCAACACGAGAGGAATGGAATGGAATGGAATGGAACAGAACGGAATGGAAAGGAGTGGAATGGAATGAACCCGAATGGAATGGAATGGATTGGAATTTAATGGAATGGAATCAACCTGAGCGGAAAGGAATGGAATGGAATGGAGTGGAATGGAATGTAACGGAATGGAATGGAATGGAATGGAATCATCACGAATGGAATGGAACGGAATGTAATGGGATGGGATGGAATGGAAGAGAATGGATGTTAATCAACACGAATGGAATGGAATGGAATGGAATAGAATGGAATGGAATGGAATGGATCGGAATGGAATCCAATGGAATCAACTGGAATGGAATGCAATGTAATGGAATGGAATGGAATGGAATGGATTGGAATGCAATCGAATGGAATGGAATGGAATAAATCCGGGTGGAGTGGAATGGAATGTAATGGAGTGGAATGTAATGGAATTTAGTGGAATGGAATGGAATGGAATGGAATGGAATGAAATCTATTGGATCAGAATGGAA

**Supplementary File 1B.** **GS13-RC20**

IGV view of the sequence reads on chromosome 20 for GS13-RC20. **a.** CNV analysis revealed no consistent soft-clipped regions at 20p13 or 20q13.33. **b**. At the end of 20p13, with the first aligned read at chr20:6. **c**. At the end of 20q13.33, with read coverage extending to chr20:66,202,178.

**ISCN:** seq[T2T] r(20)(p13q13.33) g.(pter)_6del::66202178_(qter)del

**a**.


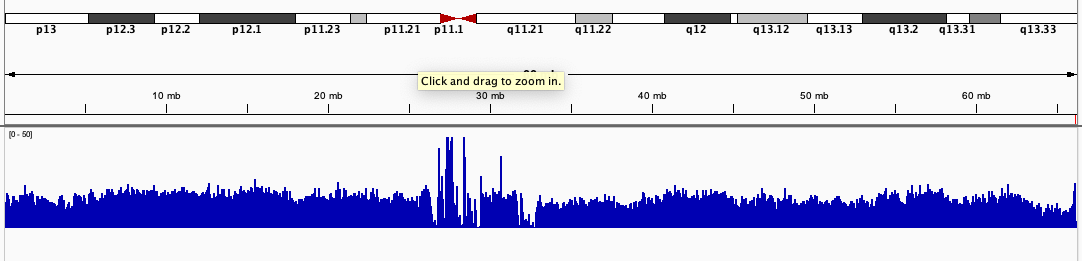


**b**.


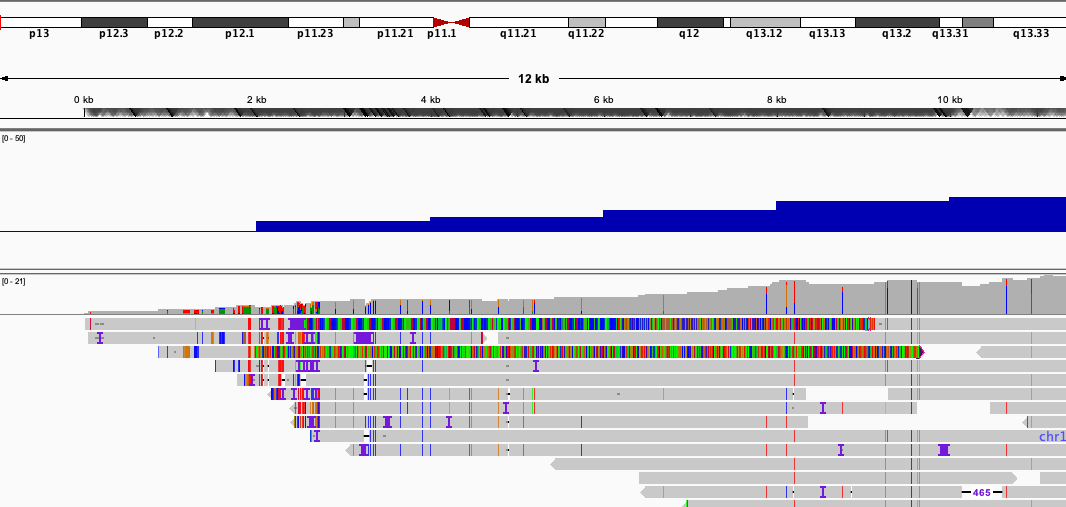


**c**.


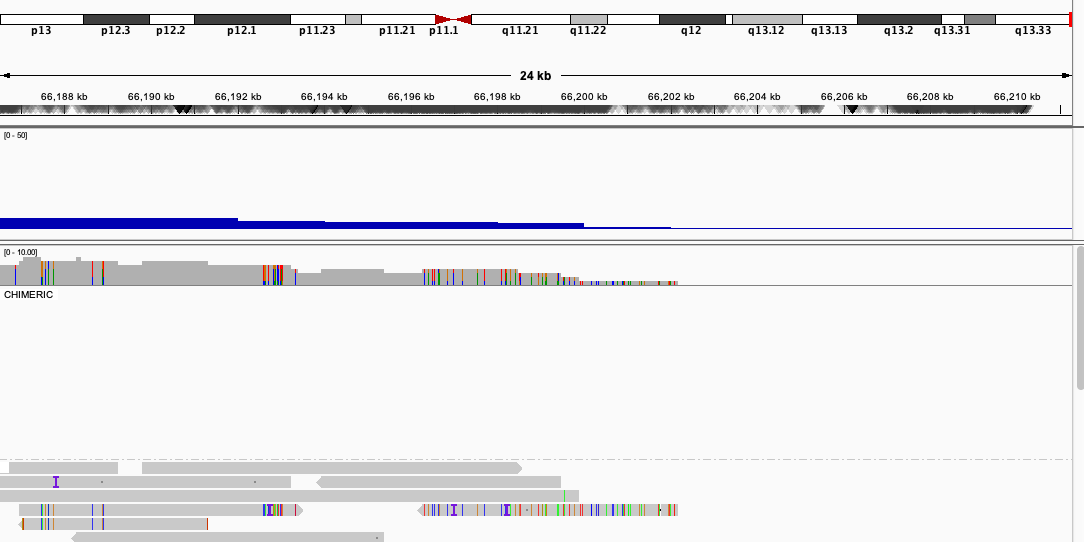

Supplement: Supplementary File 1 [file mmc2.docx]
