# Supplementary material for: Unravelling ring chromosome structures and formation mechanisms by short-read and long-read genomic sequencing
Source: Genet Med Open. 2025 Nov 19;4:103475. doi: 10.1016/j.gimo.2025.103475 (PMC13207348; doi:10.1016/j.gimo.2025.103475)
Supplement: Supplementary File 2 [file mmc3.docx]

**Supplementary File 2. Incomplete RCs by one arm breakage followed by telomeric or centromeric fusion.**

**A. Breakage-fusion sequence for GS1-RC3.**

**B. Breakage-fusion sequence for GS2-RC4.**

**C. Breakage-fusion sequence for GS8-RC18.**

**D. Breakage-fusion sequence for GS7-RC18.**

**E. Breakage-fusion sequence for GS11-RC18.**

**Supplementary File 2A. GS1-RC3**

**Breakage-fusion sequence for GS1-RC3 (microhomology)**

**3p26.1(+)** 6010280-TTAAACCAGCTTGAAA**TCA**CTTCAGTTCTTTGTAGCCACAT-6010320

**3q29(+)** 20110379-TCAGGGTCAGGGGTCAGGGGTCAAGGGGTCAAGGG**TCA**GG-201103828

[Telomeric variant repeat (TCAGGG)n likely derived from (TGAGGG)n]

**Fusion sequence:** TCAGGGGTCAAGGGGTCAAGGG**TCA**CTTCAGTTCTTTGTAGCCACAT

**ISCN:** seq[T2T] r(3)(p26.1q29) g.(pter)_6010295del::201103823-(qter)del

1. **CNV analysis showing a 6.01 Mb distal deletion of 3pter-p26.1**

**
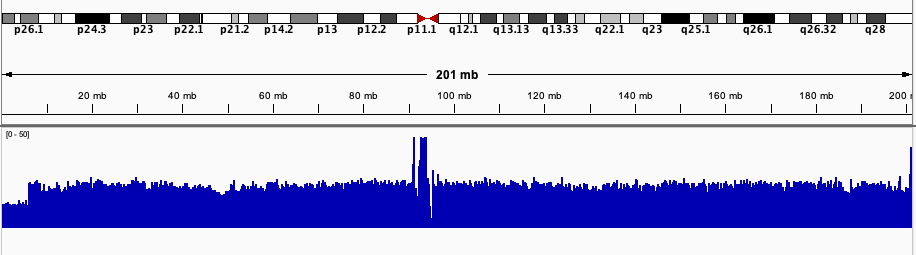
**

1. **A closer examination of the 3p26.1 region revealed the soft-clipped reads.**

chr3:6,010,296 (left-clipped sequence)

**
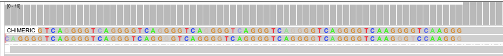
**

**c. BLAT search results to T2T assembly**

The 13 Kb lrGS sequence matching to chr3:201088525-201103828 (99.6% identity), showing below the distal 1.822 Kb from exact match to dispersed matches by (GGGTTA)n and telomeric variant repeat (TVR) (TCAGGG)n (highlighted blue) at the fusion junction to chr3:6010296 by a microhomology sequence **TCA** (highlighted yellow):

[chr3:201100315-201101332-(GGGTTA)n-(TCAGGG)n-201103823] CAGCAGGGGGGCGC

CCTGGCACAGCACCGTGAGCAAGCGGGTCCTGTAGTGCCCGGCTGCAAGCAAGGGGCTGTCGATCCCGGCGTTTCGGATTACTGAGGTTCCACCCGTCTCTGCGCCGCGCCGCCGTGACGTGAGTTTCTGCGCGTGCACGGCGCCACCCCCCCCCCCGCCCCCAGCCCGGCGCCGTGCGACTTTGCTCCTGTAACACACGCCCCCCCAACCCCCGCCCGTAGGCGTGCGTCTCTGCGCCTGCGCCACGCCTCCACCCCGGACGCGCTAGCATGTGTCTCTGCGCCTGCGCCGGCGCGGCGCGCCTCTCTGCGCCTGCGCCACGCCTCCTCCCCTGGGCGCGCTAGCATGTGTCTCTGCGCCTGCGCCACGCCTCCTCCCCTGGACGCGCTAGCATGTGTCTCTGCGCCTGCGCCGGCGCGGCGCGCCTCTCTGCGCCTGCGCCGGCGCGGCGCGCCTCTCTGCGCCTGCGCCGGGCGCGGCGCGCCTCTCTGCGCCTGCGCCGGCGCGGCGCGCCTCTCTGCGCCTGCGCCGGCGCGGCGCGCCTCTCTGCGCCTGCGCCGGCGCGGCGCGCCTCTCTGCGCCTGCGCCGGCGCGGCGCGCCTCTCTGCGCCTGCGCCGGCGCGGCGCGCCTCTCTGCGCCTGCGCCGGCGCGGCGCGCCTCTCTGCGCCTGCGCCGGCGCGGCGCGCCTCTCTGCGCCTGCGCCGGCGCGGCGCGCCTCTCTGCGCCTGCGCCGGCGGAGTTGCGTTCTCTTCAGCACAGACCCGGAGAGCACCGCGAGGGCGGAGCTGCGTTGTCCTCTGCACAGATTTCGGTGGTACTGCGAAGGCGGAGCAGAGTTCTCCTCAGGTCAGACCCGGGCGGGCGGGCCGGCTGAGGGTACCGCCAGGGTTAGGGTTAGGGTTAGGGTTAGGGTTAGGGTTAGGGTTAGGGTTAGGGTTAGGGTTAGGGTTAGGGTTAGGGTTAGAGGGTTAGGGTTAGGGTTAGGGTGAGGGTTAGGGTGAGGGTTAGGGTGAGGGGTTAGGGTTAGAGGGTTAGGGTTAGGGTTAGGGTTAGGGTTAGGGTTAGGTTTTAGGGTTAGGGTTAGGGTTAGGGTTAGGGTTAGGGTAGGGTTAGGGTTAGGGTAGGGTTAGGTTAGGGTTAGGGTTAGGGGTTAGGGTTAGGGTTAGGGTTAGGGTTAGGGGTTAGGGTTAGGGTTAGGGTTAGGGTTAGGATGGGTTAGGGTTAGGGTTAGGGTTAGGGTTAGGGTAGGGTTAGGGTTAGGGTTAGGGTAGGGTTAGGGTTAGGGTTAGGGTTAGGGTTAGGGTTAGGGTTTAGGGTTAGGGTTAGGGTTAGGGTTAGGGTTAGGGGTAGGGTTAGGGTTAGGGTTAGGGTTAGGGTTAGGGTTAGGGTTAGGGTTAGGGTTAGGGTTAGGGTTGGGTTAGGGTTAGGGTTAGGGTTAGGGTTAGGGTTAGGGTAGGGTTAGGGTTAGGGTTAGGGTTAGGGTTAGGGTTAGGGTTGGGTTAGGGTTAGGTTGGGTTAGGGTTAGGTTAGGGTTAGGGTTAGGGTTAGGGTAGGGTTAGGGTTAGGGTTAGGGTTAGGGTTAGGGTTAGGGTTTGGGTTAGGGTTTGGTAGGGTTAGGGTTAGGGGGTGGGTTTGGGTTAGGGTTAGGGTTAGGGTTAGGGTCAGGGTCAGGGTGGTGGTCGGTCAGGGTCAGGGTTCAGGGGTCAGGGTCAGGGTCAGGGGTCAGGGGTCAGGGGTCAGGGGTCAGGGGTCAGGGGTCAGGGGTCAGGGGTCAGGGTCAGGGGTCAGGGGTCAAGGGGTCAAGGG::[chr3:6,010,296]**TCA**CTTCAGTTCTTTGTAGCCACAT

**Supplementary File 2B. GS2-RC4**

**Breakage-fusion sequence for GS2-RC4 (microhomology)**

**4p16.3(+)** 1860970-GTCCCTGTGCAGAACGGGGG**CTGC**GGGGGCGGCAGGAGGAA-1861010

**4q35.2(+)** 193550595-AGCAGCTTCTCCTCTATGTTCTTCA**CTGC**CTCATACTGTTGTTGA-193550639

**Fusion sequence:** AGCAGCTTCTCCTCTATGTTCTTCA**CTGC**GGGGGCGGCAGGAGGAA

**ISCN:** seq[T2T] r(4)(p16.3q35.2) g.(pter)_1860889del::193550619_(qter)del

**a. CNV analysis showing a 1.86 Mb distal deletion of 4pter-p16.3**


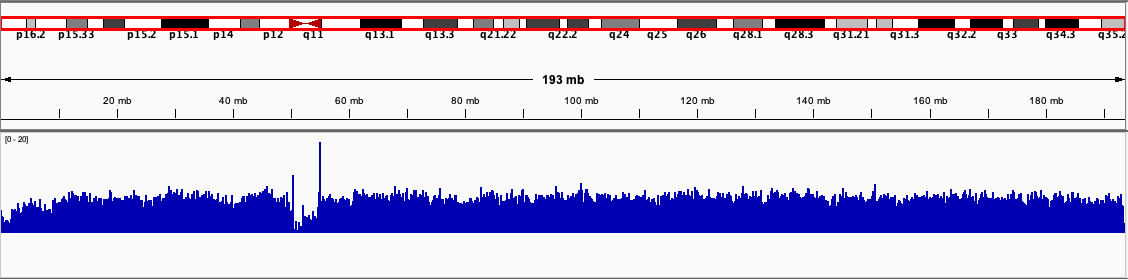


**b. A closer examination of the 4p16.3 region revealed the soft-clipped reads.**

chr4:1860994
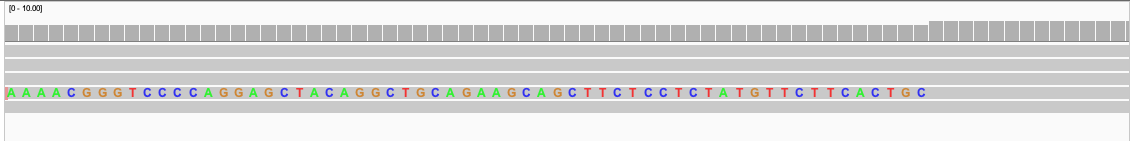


1. **IGV view of the chimeric alignments in split screen**

chr14:1860994---------------------------------------------chr4:193550623


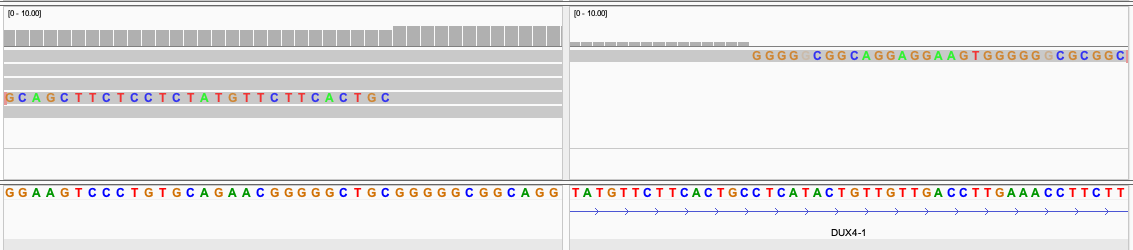


**d. BLAT search results to T2T assembly**

The 2.002 Kb lrGS result matching chr4:193548622-193550619 (highlighted blue, identity 99.8%) at the fusion junction to chr3:1860990 by a microhomology sequence **CTGC** (highlighted yellow):

GAGCCTAGACAAATGTTCCATCGCCTGGGTGATCAGTGCAGAGATATGTGACAAGGCCCCTTTAAGCAGAGCCTAGACAATAGTTACATCACCTGAGTGATCAATGCAGTGATATGCCACTACGCCCCAGTAGGCAGAGCCTAGTCAAGCGTTACATCACCTGAGTGATCAGTGCAGAGATATGTCACAAAGCCCCCATACACAGAGCCTAGACAACAGTCCCATCCCCTGGGTGATCAGTGCAGAAATATGTCGCAATGCCCCCATAGGCAGATCCAACACAAGAGTTACATCACCTGGGTGATCAGTGTAGAGATATGTCACAATGCCCCCATAGGCAGAGCGTAGACAAAAGTCCCATCACCAAGGTGATCAGTGCAGAGATATGTCACAAAGCCCCCATAGGCAGAGCCTAGACAAGAGTTACATCACTTGGTTGATCAGTTCAGAGATGTGTCACAATGCCCATGTAGGCAGAGCCTACACCAGTGTTACATCACTTAGGTGATCAGTGCAGAGCTATGTCACAATACCCCCGTAAGCAGAGCCTAGACAAGAGTTACATCACCTGGTTGATCAGTGCAGAGATATCTCACAATGTCCCTGCAGGCAGAGTATAGACAAGAGTTACATCACCTAGATGATCAGTGCAGAGATATTTCACAATGCCCCCTGTAGGCAGAGCCTAGATAAGAATTATATTACCTGGATGATCAGTACGGTGATATGTCACTATGTCCCCTGTGGGCGGAGCCTAGACAAGAGTTACATCACCTGGGTCATAAGGGCAGAGATATGTCACAATGCTCCAGTAGGCAGAGCCTAGACAAGAGTCCTATCACCTGGGTGATCAGTGCAGAAATATGTCACAATGCTCCCAGTAGACAGAGCCTAGACAAGAGTTACATCACCTGGGTGATCAGTGCAGAAATATGTTGCAATGCCCCCATAGGCAGATCCAACACAAGAGTTACATCACCTGGGTGATCAGTGCAGAGATATGTAACAATGCCCCCAGTAGGCAGAGCCTAGAGGAGAGTTACATCATCTGGGTGATCTTTGCAGAGATATGTCACAATCCCCCAAGTAAGCAGAGCCTAGACAAAAGTTACATCATCTGGGCGATCAGTGCAGAGAGAAGTCACAAAACCCACATAGGAAAAGACTAGACAAGAGTTACATCATCTGGGTCATCAGTGCAGACATATGTCAAAGCTGCCGTAGACAGAGTGTAGACAATTATTACATCACTTGGGTGATCAGTGCAGAGATCTATCACAGTGCCCCCATAGGCAGAGCCTAGACAAGAGTTCCATCACCTGTGTGATCAGTGTAGAGATATGTCACAATGCCTCCTGTAGGCAGAGGCTAAACAAGAGTTACATCACCTGGATTTTGTTTCCTGCAATATGTCACAATGGCGAGGGTGAGGGTTAGGGTGAGGGTGAGGGTTAGGGTGAGGGTCAGGGTGAGGGTGAGGGTTAGGGTTAGGGTGAGCATTAGGTTTAGGGTTAGGGTTAGGGTTAGGGGTTAGGCTTAGGCTTAGGGTTAGGCTTAGGCTTAGGGTAAGGCTTAGGGTTCAGGTTCAAGTTTGGATTCGGGTTCAGGTTAAGAGTTAGGGTTAGGGTTACTGGTTAGGGTTAGGGGTTAGGGTTAGGGGTTAGGGCTGGGTTAGGTTTAGGGTTAGGGTTAGGGTTATGGGTTACAGTTAGGGTTAGGGTTAGGTTTTAGGGTTAAGGTTAGGGTTAGGATTGGGGTTAGGTTTAGGGTTAGGGTAGTGTAAATAATTTCACATTATTACTAATAATAAATTATTATTTATATTACACTATTACTTAATATATAGGCTATTAAGACATGTTTGTCTTCAAAGAATGGCCTTGGTTTCTGTGGACAGTTTCTCCTCATGGAAAGGTAGTGTGTTCCTGCTAAATCATGGACAAAACGGGTCCCCAGGAGCTACAGGCTGCAGAAGCAGCTTCTCCTCTATGTTCTTCA-**CTGC**GGGGGCGGCAGGAGGAA

**Supplementary File 2C. GS8-RC18**

**Breakage-fusion sequence for GS8-RC18 (blunt ends)**

**18p11.32(+)**  55565-TAACCCTAACCCTAACCCTAACCCTAACCC-55594

**18q21.33 (+)** 61845450-TAGACGCTGAATTGCTGGGACATTACATTTTTCATTTAATC-61845490

**Fusion sequence:** TAGACGCTGAATTGCTGGGACTAACCCTAACCCTAACCC

**ISCN:** seq[T2T] r(18)(p11.32q21.33) g.(pter)_55654del::61845470_(qter)del

**a.** **CNV analysis showing 18.7 Mb distal deletion of 18q21.33-qter**


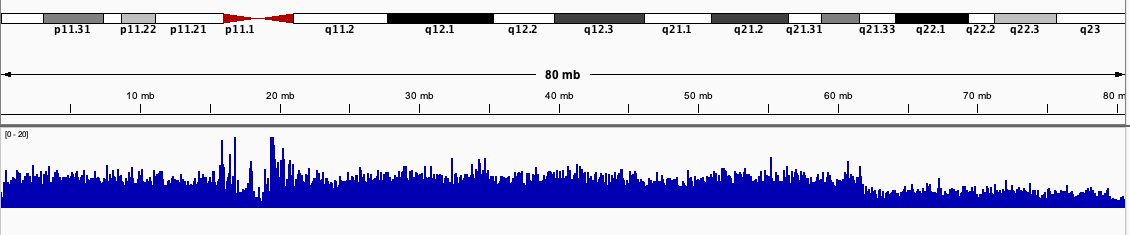


**b.** **A closer examination of the 18q21.33 region revealed the soft-clipped reads.**

Chr18:61845470 (right-clipped sequences)


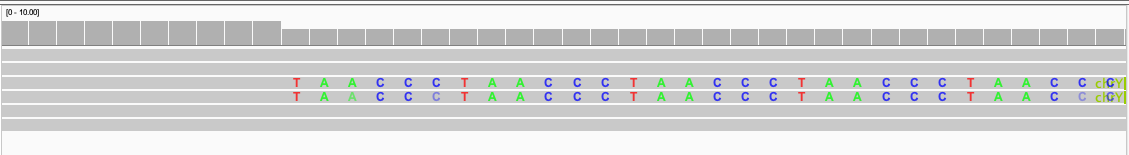


**c. BLAT search results: Chr18:61845470 link to (TAACCC)n at 18p11.32**

A 9.717 kb of lrGS sequence show 20 blocks of (TAACCC)n from chr18:1-217986 (identity 98%). Distal 103 bp (wave underlined) matches to chr18:2006-2085; 56082-56161, 110080-110159, 164119-164198 (identity 94.9%). Based on the size of (TAACCC)n repeat, the breakpoint at 18p11.32 was estimated at ~55565 with (TAACCC)n anchored by the 103 bp sequence at 56082-56161.

-(TAACCC)n-TAACCCCTAACCCCTAACCCCTAACCCCTAACCCAACCCTAACCCTAACCC

TAACCCTAACCCTAACCCTAACCCTACCCTAACCCTAACCCTAACCCTAACCCAAACCCTAACCCTAACCCTAACCCTAACCCTAACCCTAACCCTAACCCTAACCCTACCCTAACCCTACCCTAACCCTAACCCTAACCCTAACCCTAACCCTAACCCTAACCTAACCCTAACCCTAACCCTAACCCCTAACCCTAACCCTAACCCAACCCCAACCCCAACCCTAACCCCAAACCTAACCCCTAACCCTAACCCTAACCCTAACCCTAACCCTCTAACCCTAACCCTCTAACCCTAACCCTAACCCTAACCCTAACCCTACCCCTACCCCTACCCTAACCCTACCCTAACCCTAACCCTAACCCTAACCCTAACCCTAACCCTAACCCCTAACCCTAACCCTAACCCTAACCCTAACCCCTAACCCCTAACCCCGACCCCGACCCCGACCCCGACCCTAACCCTAACTGGACTCTGACCCTGATTGTTCAGGGCAGCAAAGAGGAAGAATTTTATTTACCGTCGCTTTGG

**Supplementary File 2D. GS7-RC18**

**Breakage-fusion sequence for GS7-RC18 (microhomology)**

**18p11.1(+)** 17848791-CTTGAAACACCC**CTTTT**GTAGTATCTGGAACTGGACTTTT-17848830

**18q23(+)** 78795540-TTGTTTTCTAATATGGAAAA**CTTTT**AAATTTCCTAAGAATG-7879580

**Fusion sequence:** TTGTTTTCTAATATGGAAAA**CTTTT**GTAGTATCTGGAACTGGACTTTT

**ISCN:** seq[T2T] r(18)(p11.1q23) g.(pter)_17848802del::78795559_(qter)del

**a.**  **CNV analysis showing a 17.85 Mb distal deletion of 18pter-p11.1 and a 1.8 Mb distal deletion of 18q23-qter**


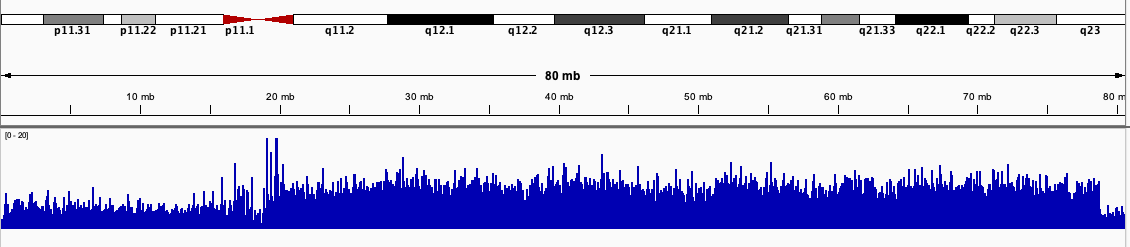


**b. A closer examination of the 18q23 region revealed the soft-clipped reads.**

Chr18:78795560—right-clipped sequences


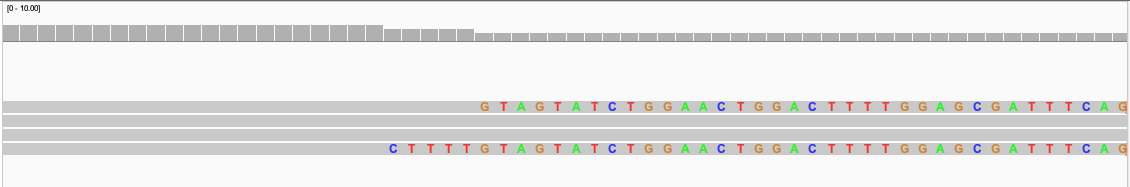


**c. BLAT search results to T2T**

The 9.448 kb lrGS sequence matches 16 segments of centromeric satellite sequences (S2C18H1L) in an 873.2 Kb region (chr18:18537471-194106810, identity 99.7%-99.8%) at 18q11.1. BLAT the proximal 1.031 kb (wave lined) matches 16 segments in a 2.15 Mb region (chr18:17,255,448-19,408,368, identity 99.8-99.9%) at 18p11.1-q11.1. The fusion sequence was arbitrary assigned to the most proximal identical match (chr18:17,848,803-17,849,833, identity 100%) at 18p11.1. Microhomology sequence **CTTTT** present at the fusion sequence.

CTTTTGTAGTATCTGGAACTGGACTTTTGGAGCGATTTCAGGGCTAAGGTGAAAAAGGAAATATCTTCCCATAAAAACTGGACAGAAGCATTCTCAGAAACTTGTTTATGCTGTATCTACTCAACTAACAAAGTTGAACCTTTCTTTTGATAGAGCAGTTTTGAAATGGTCTTTTTGTGGAATCTGCAAGTGGATATTTGGCTAGTTTTGAGGATTTCGTTGGAAGCGGGAATTCATACAAATTGCAGACTGCAGCGTTCTGAGAAACATCTTTGTGATGTTTGTATTCAGGACACAGAGTTGAACATTCCCTATCATAGAGCAGGTTGGAATCACTCCTTTTGTAGTATCTGGAAGTGGACATTTGGAGCGCTTTCAGGCCTATTTTGGAAAGGGAAATATCTTCCCGTAACAACTATGCAGAAGCATTCTCAGAAACTTGTTTGTGATGTGTGCCCTCTACTGACAGAGTTGAACCTTTCTTTTCATAGAGCAGTTTTGAAACACTCTTTTTGTAGAATCTGCAAGAGGATATTTGCATAGCTTTGAGGATTTCGTGGGAAACGGGATTGTCTTCAGGTAAAATCTAGACAGAAGCATTCTCAGAAACTTCTTTGGGATGTTTGCATTCAAGTCACAGAGTAGAACATTCCCTTTGGTAGAGCAGGTTTGAAACACTCTTTTTGTAGTATCTGGAAGTGGACATTTGGAGCGCTTTCAGGCCCATGTTGGAAAGGGAAATATCTTCCCGTAACAACTAGGCAGAAGCATTCTCAGAAACTTATTTGAGATGTGTGTACTCAACTAAGAGAATTGAACCACCGTTTTGAAGGAGCAGTTTTGAAACACTCTTTTTCTGGAATCTGCAAGAGTATATTTGCCTAGCCTTGAGGATTTCGTTGGAAACGGGATTGTCTTCAGAGAAAATCTAGACAGAAGCATTCTCAGAAACCTCTTTGGGATGTTTGCATTCAAGTCACAGAGTAGAACATTCCCTTTGGTAGAGCAGGTTTGAAACACTCTTTTTTTAGTATATGGAAGTGGACATTTGGAGCGCTTTCAGGCCTACGTTGGAAAAGGAAATATCTTCCCATAACAACTAGACAGAAGCATTCTCAGAAACTAGTTTCTGATGTGTGTCCTCAACTAACACAGTTGAACTTTTCTTTAGACAGAACAGTTTTGAAACACTCTTTTTGTGGAATCTGCAAGTGGATATTTGGCTAGATTTGAGGATTTCGTTGGAAACGGGATTACATATTAAAAGCAGACAGCAGCATTCTCAGAAAGTTCTTTGTGATGATTGCATTCAAGTCACAGAATTGAACATTCCCTTTCACAGAGCAGGTTTGAAACACTCTTTTTGTAGTGTGTGTAAGTGGACATTTGGAGCACTTACCGGCCTAAGGTGAAAAAGGAAATATCTTCCCATAAAAACTAGACAGAAGCATTCTCAGAAACTTACTCGTGATGTGTGTCCTCAACTAAAGGAGTAGAACCTTTCTTTTCATAGAGAAGTTTTGAAACGCTCTTTTTGTGGAATCTGCAAGTGGATATTTGGCTAGTTTTGAGGATTTCGTTGGAAGCGGGAATTCATACAAATTGCAGACTGCAGCGTTCTGAGAAACATCTTTGTGATGTTTGTATTCAGGACACAGAGTTGAACATTCCCTATCATAGAGCAGGTTTGAATCACTCCTTTTGTAGTATCTGGAAGTGGACATTTGGAGTGCTTTCAGGCCTATGTTGGAAAAGGAAATATCTTCCCATAACAACTAGACAGAAGCATTCTCAGAAACTTATTTGAGATGTGTGTACTCAACTAAGAGAATTGAACCACCGTTTTGAAGGAGCAGTTTTGAAACACTCTTTTTCTGGAATCTGCAAGTGGATATTTGGCTAGCTTTGGGGATTTCGCTGGAAGCGGGAATACATATAAAAAGCACACAGCAGCGTTCTGAGAAACTGCTTTCTGATGTTTGCATTCAAGTCAAAAGTTGAACACTCCCTTTCATAGAGCAGTCTTGAAACACCCCTTTTGTAGTATCTGGAACTGGACTTTTGGAGCGATTTCAGGGCTAAGGTGAAAAAGGAAATATCTTCCCATAAAAACTGGACAGAAGCATTCTCAGAAACTTGTTTATGCTGTATCTACTCAACTAACAAAGTTGAACCTTTCTTTTGATAGAGCAGTTTTGAAATGGTCTTTTTGTGGAATCTGCAAGTGGATATTTGGCTAGTTTTGAGGATTTCGTTGGAAGCGGGAATTCATACAAATTGCAGACTGCAGCGTTCTGAGAAACATCTTTGTGATGTTTGTATTCAGGACACAGAGTTGAACATTCCCTATCATAGAGCAGGTTGGAATCACTCCTTTTGTAGTATCTGGAAGTGGACATTTGGAGCGCTTTCAGGCCTATTTTGGAAAGGGAAATATCTTCCCGTAACAACTATGCAGAAGCATTCTCAGAAACTTGTTTGTGATGTGTGCCCTCTACTGACAGAGTTGAACCTTTCTTTTCATAGAGCAGTTTTGAAACACTCTTTTTGTAGAATCTGCAAGAGGATATTTGCATAGCTTTGAGGATTTCGTGGGAAACGGGATTGTCTTCAGGTAAAATCTAGACAGAAGCATTCTCAGAAACTTCTTTGGGATGTTTGCATTCAAGTCACAGAGTAGAACATTCCCTTTGGTAGAGCAGGTTTGAAACACTCTTTTTGTAGTATCTGGAAGTGGACATTTGGAGCGCTTTCAGGCCCATGTTGGAAAGGGAAATATCTTCCCGTAACAACTAGGCAGAAGCATTCTCAGAAACTTATTTGAGATGTGTGTACTCAACTAAGAGAATTGAACCACCGTTTTGAAGGAGCAGTTTTGAAACACTCTTTTTCTGGAATCTGCAAGAGTATATTTGCCTAGCCTTGAGGATTTCGTTGGAAACGGGATTGTCTTCAGAGAAAATCTAGACAGAAGCATTCTCAGAAACTTCTTTGGGATGTTTGCATTCAAGTCACAGAGTAGAACATTCCCTTTGGTAGAGCAGGTTTGAAACACTCTTTTTTTAGTATATGGAAGTGGACATTTTGATCGCTTTCAGGCCTACGTTGGAAAAGGAAATATCTTCCCATAACAACTAGACAGAAGCATTCTCAGAAACTAGTTTCTGATGTGTGTCCTCAACTAACACAGTTGAACATTTCTTTAGACAGAACAGTTTTGAAACACTCTTTTTGTGGAATCTGCAAGTGGCTATTTGGCTAGATTTGAGGATTTCGTTGGAAACGGGATTACATATAAAAAGCAGTCAGCAGCATTCTCAGAAAGTTCTTTGTGATGATTGCATTCAAGTCACAGAATTGAACATTCCCTTTCACAGAGCAGGTTTGAAACACTCTTTTTGTAGTGTGTGTAAGTGGACATTTGGAGCACTTACCGGCCTAAGGTGAAAAAGGAAATATCTTCCCATAAAAACTAGACAGAAGCATTCTCAGAAACTTACTCGTGATGTGTGTCCTCAACTAAAGGAGTAGAACCTTTCTTTTCATAGAGAAGTTTTGAAACGCTCTTTTTGTGGAATCTGCAAGTGGATATTTGGCTAGTTTTGAGGATTTCGTTGGAAGCGGGAATTCATACAAATTGCAGACTGCAGCGTTCTGAGAAACATCTTTGTGATGTTTGTATTCAGGACACAGAGTTGAACATTCCCTATCATAGAGCAGGTTTGAATCACTCCTTTTGTAGTATCTGGAAGTGGACATTTGGAGTGCTTTCAGGCCTATGTTGGAAAAGGAAATATCTTCCCATAACAACTAGACAGAAGCATTCTCAGAAACTTATTTGAGATGTGTGTACTCAACTAAGAGAATTGAACCACCGTTTGAAGGAGCAGTTTTGAAACACTCTTTTTCTGGAATCTGCAAGTGGATATTTGGCTAGCTTTGGGGATTTCGCTGGAAGCGGGAATACATATAAAAAGCACACAGCAGCGTTCTGAGAAACTGCTTTCTGATGTTTGCATTCAAGTCAAAAGTTGAACACTCCCTTTCATAGAGCAGTCTTGAAACACCCCTTTTGTAGTATCTGGAACTGGACTTTTGGAGCGATTTCAGGGCTAAGGTGAAAAAGGAAATATCTTCCCATAAAAACTGGACAGAAGCATTCTCAGAAACTTGTTTATGCTGTATCTACTCAACTAACAAAGTTGAACCTTTCTTTTGATAGAGCAGTTTTGAAATGGTCTTTTTGTGGAATCTGCAAGTGGATATTTGGCTAGTTTTGAGGATTTCGTTGGAAGCGGGAATTCATACAAATTGCAGACTGCAGCGTTCTGAGAAACATCTTTGTGATGTTTGTATTCAGGACACAGAGTTGAACATTCCCTATCATAGAGCAGGTTGGAATCACTCCTTTTGTAGTATCTGGAAGTGGACATTTGGAGCGCTTTCAGGCCTATTTTGGAAAGGGAAATATCTTCCCGTAACAACTATGCAGAAGCATTCTCAGAAACTTGTTTGTGATGTGTGCCCTCTACTGACAGAGTTGAACCTTTCTTTTCATAGAGCAGTTTTGAAACACTCTTTTTGTAGAATCTGCAAGAGGATATTTGCATAGCTTTGAGGATTTCGTGGGAAACGGGATTGTCTTCAGGTAAAATCTAGACAGAAGCATTCTCAGAAACTTCTTTGGGATGTTTGCATTCAAGTCACAGAGTAGAACATTCCCTTTGGTAGAGCAGGTTTGAAACACTCTTTTTGTAGTATCTGGAAGTGGACATTTGGAGCGCTTTCAGGCCCATGTTGGAAAGGGAAATATCTTCCCGTAACAACTAGGCAGAAGCATTCTCAGAAACTTATTTGAGATGTGTGTACTCAACTAAGAGAATTGAACCACCGTTTTTGAAGGAGCAGTTTTGAAACACTCTTTTTCTGGAATCTGCAAGAGTATATTTGCCTAGCCTTGAGGATTTCGTTGGAAACGGGATTGTCTTCAGAGAAAATCTAGACAGAAGCATTCTCAGAAACCTCTTTGGGATGTTTGCATTCAAGTCACAGAGTAGAACATTCCCTTTGGTAGAGCAGGTTTGAAACACTCTTTTTTTAGTATATGGAAGTGGACATTTTGATCGCTTTCAGGCCTACGTTGGAAAAGGAAATATCTTCCCATAACAACTAGACAGAAGCATTCTCAGAAACTAGTTTCTGATGTGTGTCCTCAACTAACACAGTTGAACTTTTCTTTAGACAGAACAGTTTTGAAACACTCTTTTTGTGGAATCTGCAAGTGGATATTTGGCTAGATTTGAGGATTTCGTTGGAAACGGATTACATATAAAAAGCAGACAGCAGCATTCTCAGAAAGTTCTTTGTGATGATTGCATTCAAGTCACAGAATTGAACATTCCCTTTCACAGAGCAGGTTTGAAACACTCTTTTTGTAGTGTGTGTAAGTGGACATTTGGAGCACTTACCGGCCTAAGGTGAAAAAGGAAATATCTTCCCATAAAAACTAGACAGAAGCATTCTCAGAAACTTACTCGTGATGTGTGTCCTCAGCTAAAGGAGTAGAACATTTCTTTTCATAGAGAAGTTTTGAAACGCTCTTTTTGTGGAATCTGCAAGTGGATATTTGGCTAGTTTTGAGGATTTCGTTGGAAGCGGGAATTCATACAAATTGCAGACTGCAGCGTTCTGAGAAACATCTTTGTGATGTTTGTATTCAGGACACAGAGTTGAACATTCCCTATCATAGAGCAGGTTTGAATCACTCCTTTTGTAGTATCTGGAAGTGGACATTTGGAGTGCTTTCAGGCCTATGTTGGAAAAGGAAATATCTTCCCATAACAACTAGACAGAAGCATTCTCAGAAACTTATTTGAGATGTGTGTACTCAACTAAGAGAATTGAACCACCGTTTTGAAGGAGCAGTTTGGAAACACTCTTTTTCTGGAATCTGCAAGTGGATATTTGGCTAGCTTTGGGGATTTCGCTGGAAGCGGGAATACATATAAAAAGCACACAGCAGCGTTCTGAGAAACTGCTTTCTGATGTTTGCATTCAAGTCAAAAGTTGAACACTCCCTTTCATAGAGCAGTCTTGAAACACCCCTTTTGTAGTATCTGGAACTGGACTTTTGGAGCGATTTCAGGGCTAAGGTGAAAAAGGAAATATCTTCCCATAAAAACTGGACAGAAGCATTCTCAGAAACTTGTTTATGCTGTATCTACTCAACTAACAAAGTTGAACCTTTCTTTTGATAGAGCAGTTTTGAAATGGTCTTTTTGTGGAATCTGCAAGTGGATATTTGGCTAGTTTTGAGGATTTCGTTGGAAGCGGGAATTCATACAAATTGCAGACTGCAGCGTTCTGAGAAACATCTTTGTGATGTTTGTATTCAGGACACAGAGTTGAACATTCCCTATCATAGAGCAGGTTGGAATCACTCCTTTTGTAGTATCTGGAAGTGGACATTTGGAGCGCTTTCAGGCCTATTTTGGAAAGGGAAATATCTTCCCGTAACAACTATGCAGAAGCATTCTCAGAAACTTGTTTGTGATGTGTGCCCTCTACTGACAGAGTTGAACCTTTCTTTTCATAGAGCAGTTTTGAAACACTCTTTTTGTAGAATCTGCAAGAGGATATTTGCATAGCTTTGAGGATTTCGTGGGAAACGGGATTGTCTTCAGGTAAAATCTAGACAGAAGCATTCTCAGAAACTTCTTTGGGATGTTTGCATTCAAGTCACAGAGTAGAACATTCCCTTTGGTAGAGCAGGTTTGAAACACTCTTTTTGTAGTATCTGGAAGTGGACATTTGGAGCGCTTTCAGGCCCATGTTGGAAAGGGAAATATCTTCCCGTAACAACTAGGCAGAAGCATTCTCAGAAACTTATTTGAGATGTGTGTACTCAACTAAGAGAATTGAACCACCGTTTTGAAGGAGCAGTTTTGAAACACTCTTTTTCTGGAATCTGCAAGAGTATATTTGCCTAGCCTTGAGGATTTCGTTGGAAACGGGATTGTCTTCAGAGAAAATCTAGACAGAAGCATTCTCAGAAACCTCTTTGGGATGTTTGCATTCAAGTCACAGAGTAGAACATTCCCTTTGGTAGAGCAGGTTTGAAACACTCTTTTTTTAGTATATGGAAGTGGACATTTTGATCGCTTTCAGGCCTACGTTGGAAAAGGAAATATCTTCCCATAACAACTAGACAGAAGCATTCTCAGAAACTAGTTTCTGATGTGTGTCCTCAACTAACACAGTTGAACATTTCTTTAGACAGAACAGTTTTGAAACACTCTTTTTGTGGAATCTGCAAGTGGCTATTTGGCTAGATTTGAGGATTTCGTTGGAAACGGGATTACATATAAAAAGCAGTCAGCAGCATTCTCAGAAAGTTCTTTGTGATGATTGCATTCAAGTCACAGAATTGAACATTCCCTTTCACAGAGCAGGTTTGAAACACTCTTTTTGTAGTGTGTGTAAGTGGACATTTGGAGCACTTACCGGCCTAAGGTGAAAAAGGAAATATCTTCCCATAAAAACTAGACAGAAGCATTCTCAGAAACTTACTCGTGATGTGTGTCCTCAACTAAAGGAGTAGAACCTTTCTTTTCATAGAGAAGTTTTGAAACGCTCTTTTTGTGGAATCTGCAAGTGGATATTTGGCTAGTTTTGAGGATTTCGTTGGAAGCGGGAATTCATACAAATTGCAGACTGCAGCGTTCTGAGAAACATCTTTGTGATGTTTGTATTCAGGACACAGAGTTGAACATTCCCTATCATAGAGCAGGTTTGAATCACTCCTTTTGTAGTATCTGGAAGTGGACATTTGGAGTGCTTTCAGGCCTATGTTGGAAAAGGAAATATCTTCCCATAACAACTAGACAGAAGCATTCTCAGAAACTTATTTGAGATGTGTGTACTCAACTAAGAGAATTGAACCACCGTTTTGAAGGAGCAGTTTTGAAACACTCTTTTTCTGGAATCTGCAAGTGGATATTTGGCTAGCTTTGGGGATTTCGCTGGAAGCGGGAATACATATAAAAAGCACACAGCAGCGTTCTGAGAAACTGCTTTCTGATGTTTGCATTCAAGTCAAAAGTTGAACACTCCCTTTCATAGAGCAGTCTTGAAACACCCCTTTTGTAGTATCTGGAACTGGACTTTTGGAGCGATTTCAGGGCTAAGGTGAAAAAGGAAATATCTTCCCATAAAAACTGGACAGAAGCATTCTCAGAAACTTGGTTATGCTGTATCTACTCAACTAACAAAGTTGAACCTTTCTTTTGATAGAGCAGTTTTGAAATGGTCTTTTTGTGGAATCTGCAAGTGGATATTTGGCTAGTTTTGAGGATTTCGTTGGAAGCGGGAATTCATACAAATTGCAGACTGCAGCGTTCTGAGAAACATCTTTGTGATGTTTGTATTCAGGACACAGAGTTGAACATTCCCTATCATAGAGCAGGTTGGAATCACTCCTTTTGTAGTATCTGGAAGTGGACATTTGGAGCGCTTTCAGGCCTATTTTGGAAAGGGAAATATCTTCCCGTAACAACTATGCAGAAGCATTCTCAGAAACTTGTTTGTGATGTGTGCCCTCTACTGACAGAGTTGAACCTTTCTTTTCATAGAGCAGTTTTGAAACACTCTTTTTGTAGAATCTGCAAGAGGATATTTGCATAGCTTTGAGGATTTCGTGGGAAACGGGATTGTCTTCAGGTAAAATCTAGACAGAAGCATTCTCAGAAACTTCTTTGGGATGTTTGCATTCAAGTCACAGAGTAGAACATTCCCTTTGGTAGAGCAGGTTTGAAACACTCTTTTTGTAGTATCTGGAAGTGGACATTTGGAGCGCTTTCAGGCCCATGTTGGAAAGGGAAATATCTTCCCGTAACAACTAGGCAGAAGCATTCTCAGAAACTTATTTGAGATGTGTGTACTCAACTAAGAGAATTGAACCACCGTTTTGAAGGAGCAGTTTTGAAACACTCTTTTTCTGGAATCTGCAAGAGTATATTTGCCTAGCCTTGAGGATTTCGTTGGAAACGGGATTGTCTTCAGAGAAAATCTAGACAGAAGCATTCTCAGAAACCTCTTTGGGATGTTTGCATTCAAGTCACAGAGTAGAACATTCCCTTTGGTAGAGCAGGTTTGAAACACTCTTTTTTTAGTATATGGAAGTGGACATTTGGAGCGCTTTCAGGCCTACGTTGGAAAAGGAAATATCTTCCCATAACAACTAGACAGAAGCATTCTCAGAAACTAGTTTCTGATGTGTGTCCTCAACTAACACAGTTGAACTTTTCTTTAGACAGAACAGTTTTGAAACACTCTTTTTGTGGAATCTGCAAGTGGCTATTTGGCTAGATTTGAGGATTTCGTTGGAAACGGGATTACATATAAAAAGCAGACAGCAGCATTCTCAGAAAGTTCTTTGTGATGATTGCATTCAAG

**Supplementary File 2E. GS11-RC18**

**Breakage fusion sequence for GS11-RC18 (microhomology)**

**18q11.1 (+)**18543849-TGTTTGTATTCAGGA**CACAC**AGTTGAACATTCCCTATCATA-18543889

**18q22.3 (+)** 73489330-TTTGTTATATATC**CACAC**CTGCTACTAGTGAAGTACATGAG-73489370

**Fusion sequence:** TTTGTTATATATC**CACAC**AGTTGAACATTCCCTAT

**ISCN:** seq[T2T] r(18)(q11.1q22.3) g.(pter)_18543863del::73489342_(qter)del

**a.**  **CNV analysis showing an 18.5 Mb distal deletion of 18pter-q11.1 and a 7.1 Mb distal deletion of 18q22.3-qter**


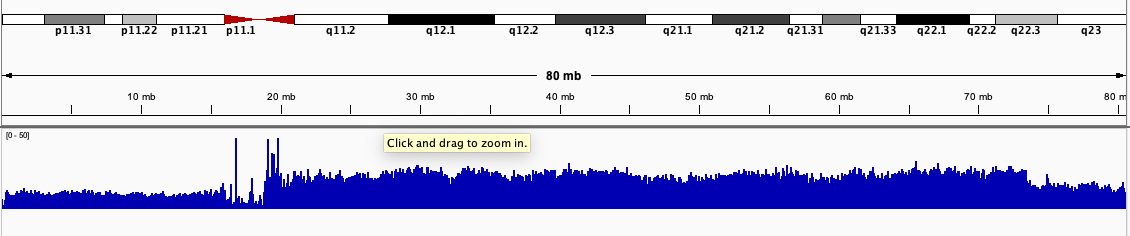


**b.** **A closer examination of the 18q22.3 region revealed the soft-clipped reads**

chr18:73489342-right-clipped sequences


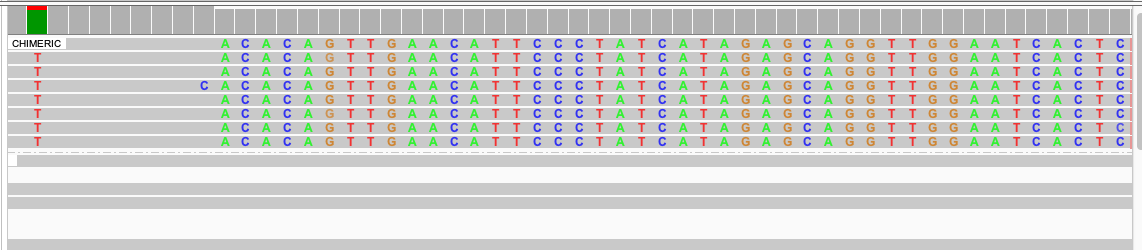


**c. BLAT search results to T2T**

The 2.658 kb lrGS sequence matches 16 segments of centromeric satellite sequences (S2C18H1L) in a 1.01 Mb region (chr18:17,812,467-18,826,322, identity 99.9%-100%) at 18p11.1-q11.1. The junction sequence was arbitrary assigned to the most proximal identical match (chr18:18,543,865-18,546,521, identity 100%) at 18q11.1. Microhomology sequence CACAC present at the fusion sequence.

ACACAGTTGAACATTCCCTATCATAGAGCAGGTTGGAATCACTCCTTTTGTAGTATCTGGAAGTGGACATTTGGAGCGCTTTCAGGCCTATTTTGGAAAGGGAAATATCTTCCCGTAACAACTATGCAGAAGCATTCTCAGAAACTTGTTTGTGATGTGTGCCCTCTACTGACAGAGTTGAACCTTTCTTTTCATAGAGCAGTTTTGAAACACTCTTTTTGTAGAATCTGCAAGAGGATATTTGCATAGCTTTGAGGATTTCGTGGGAAACGGGATTGTCTTCAGGTAAAATCTAGACAGAAGCATTCTCAGAAACTTCTTTGGGATGTTTGCATTCAAGTCACAGAGTAGAACATTCCCTTTGGTAGAGCAGGTTTGAAACACTCTTTTTGTAGTATCTGGAAGTGGACATTTGGAGCGCTTTCAGGCCCATGTTGGAAAGGGAAATATCTTCCCGTAACAACTAGGCAGAAGCATTCTCAGAAACTTATTTGAGATGTGTGTACTCAACTAAGAGAATTGAACCACCGTTTTGAAGGAGCAGTTTTGAAACACTCTTTTTCTGGAATCTGCAAGAGTATATTTGCCTAGCCTTGAGGATTTCGTTGGAAACGGGATTGTCTTCAGAGAAAATCTAGACAGAAGCATTCTCAGAAACTTCTTTGGGATGTTTGCATTCAAGTCACAGAGTAGAACATTCCCTTTGGTAGAGCAGGTTTGAAACAGTCTTTTTTTAGTATATGGAAGTGGACATTTGGAGCGCTTTCAGGCCTACGTTGGAAAAGGAAATATCTTCCCATAACAACTAGACAGAAGCATTCTCAGAAACTAGTTTCTGATGTGTGTCCTCAACTAACACAGTTGAACATTTCTTTAGACAGAACAGTTTTGAAACACTCTTTTTGTGGAATCTGCAAGTGGCTATTTGGCTAGATTTGAGGATTTCGTTGGAAACGGGATTACATATAAAAAGCAGTCAGCAGCATTCTCAGAAAGTTCTTTGTGATGATTGCATTCAAGTCACAGAATTGAACATTCCCTTTCACAGAGCAGGTTTGAAACACTCTTTTTGTAGTGTGTGTAAGTGGACATTTGGAGCACTTTCCGGCCTAAGGTGAAAAAGGAAATATCTTCCCATAAAAACTAGACAGAAGCATTCTCAGAAACTTACTCGTGATGTGTGTCCTCAACTAAAGGAGTAGAACCTTTCTTTTCATAGAGAAGTTTTGAAACGCTCTTTTTGTGGAATCTGCAAGTGGATATTTGGCTAGTTTTGAGGATTTCGTTGGAAGCGGGAATTCATACAAATTGCAGACTGCAGCGTTCTGAGAAACATCTTTGTGATGTTTGTATTCAGGACACAGAGTTGAACATTCCCTATCATAGAGCAGGTTTGAATCACTCCTTTTGTAGTATCTGGAAGTGGACATTTGGAGCGCTTTCAGGCCTATGTTGGAAAAGGAAATATCTTCCCATAACAACTAGACAGAAGCATTCTCAGAAACTTATTTGAGATGTGTGTACTCAACTAAGAGAATTGAACCACCGTTTTGAAGGAGCAGTTTTGAAACTCTCTTTTTCTGGAATCTGCAAGTGGATATTTGGCTAGCTTTGGGGATTTCGCTGGAAGCGGGAATACATATAAAAAGCACACAGCAGCGTTCTGAGAAACTGCTTTCTGATGTTTGCATTCAAGTCAAAAGTTGAACACTCCCTTTCATAGAGCAGTCTTGAAACACCCCTTTTGTAGTATCTGGAACTGGACTTTTGGAGCGATTTCAGGGCTAAGGTGAAAAAGGAAATATCTTCCCATAAAAACTGGACAGAAGCATTCTCAGAAACTTGTTTATGCTGTATCTACTCAACTAACAAAGTTGAACCTTTCTTTTGATAGAGCAGTTTTGAAATGGTCTTTTTGTGGAATCTGCAAGTGGATATTTGGCTAGTTTTGAGGATTTCGTTGGAAGCGGGAATTCATACAAATTGCAGACTGCAGCGTTCTGAGAAACATCTTTGTGATGTTTGTATTCAGGACACAGAGTTGAACATTCCCTATCATAGAGCAGGTTGGAATCACTCCTTTTGTAGTATCTGGAAGTGGACATTTGGAGCGCTTTCAGGCCTATTTTGGAAAGGGAAATATCTTCCCGTAACAACTATGCAGAAGCATTCTCAGAAACTTGTTTGTGATGTGTGCCCTCTACTGACAGAGTTGAACCTTTCTTTTCATAGAGCAGTTTTGAAACACTCTTTTTGTAGAATCTGCAAGAGGATATTTGCATAGCTTTGAGGATTTCGTGGGAAACGGGATTGTCTTCAGGTAAAATCTAGACAGAAGCATTCTCAGAAACTTCTTTGGGATGTTTGCATTCAAGTCACAGAGTAGAACATTCCCTTTGGTAGAGCAGGTTTGAAACACTCTTTTTGTAGTATCTGGAAGTGGACATTTGGAGCGCTTTCAGGCCCATGTTGGAAAGGGAAATATCTTCCCGTAACAACTAGGCAGAAGCATTCTCAGAAACTTATTTGAGATGTGTGTACTCAACTAAGAGAATTGAACCACCGTTTTGAAGGAGCAGTTTTGAAACACTCTTTTTCTGGAATCTGCAAGAGTATATTTGCCTAGCCTTGAGGATTTCGTTGGAAACGGGATTGTCA
