# Supplementary material for: Unravelling ring chromosome structures and formation mechanisms by short-read and long-read genomic sequencing
Source: Genet Med Open. 2025 Nov 19;4:103475. doi: 10.1016/j.gimo.2025.103475 (PMC13207348; doi:10.1016/j.gimo.2025.103475)
Supplement: Supplementary File 3 [file mmc4.docx]

**Supplementary File 3. Incomplete RCs by direct breakage-fusion at two arms.**

1. **Breakage-fusion sequence for GS5-RC13.**
2. **Breakage-fusion sequence for GS16-RC21.**
3. **Breakage-fusion sequence for GS9-RC22.**
4. **Breakage-fusion sequence for GS14-RCY.**

**Supplementary File 3A. GS5-RC13**

**Breakage-fusion sequence for GS5-RC13 (microhomology)**

**13p13(-)** 5349988-TCTCTCAGGTCTTTGCC**CT**CAGCCCACAGGGACTCTTGTGT-5349948

**13q34(+)** 110078850-TTCAAGCCCTTGTTTCAGTT**CT**GTTGAAAACATTTTGCTCC-110078890

**Fusion sequence:** TTCAAGCCCTTGTTTCAGTTC**CT**CAGCCCACAGGGACTCTTGTGT

**ISCN:** seq[T2T] r(13)(p13q34) g.(pter)_5349972del::110078870_(qter)del

**a.** **CNV analysis showing a 5.35 Mb distal deletion of 13pter-p13 and a 3.49 Mb distal deletion of 13q34-qter**


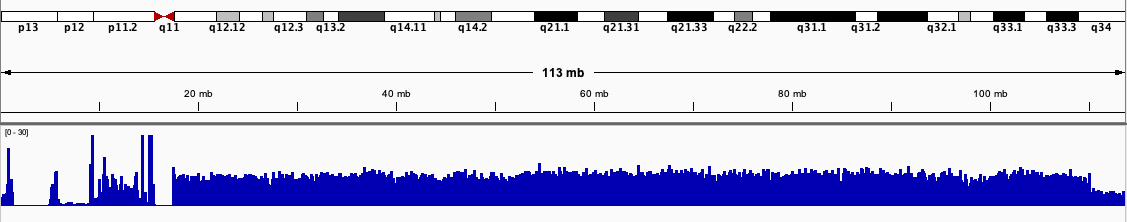


**b.** **A closer examination of the 13q34 region revealed the soft-clipped reads.**

chr13:110078869 (right-clipped sequences)


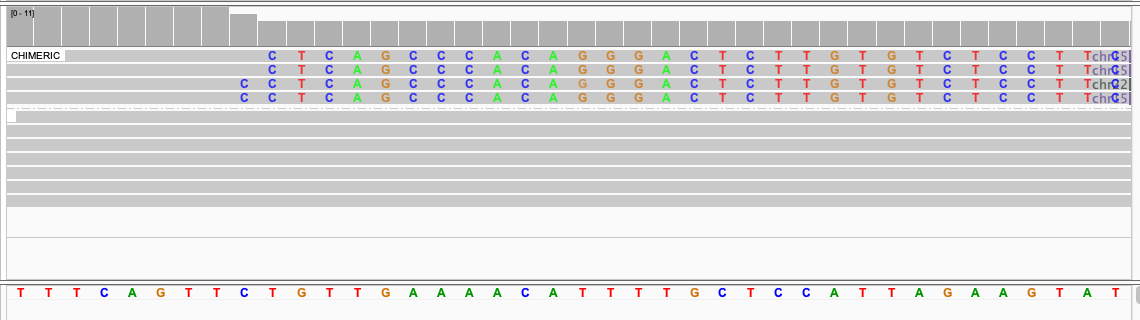


**c.** **BLAT search results in T2T**

The following sequence is the right-clipped sequences connected to IGV-chr13:110078869. Blat this 4.92 kb to T2T show a match to chr13:5344994-5349972[-] (identity 99.2%) at 13p13, which is classified as other centromeric satellites (censat_13_7). There is a fusion of 13p13(-) with 13q34(+) mediated by a microhomology sequence ‘CT’. Also there are numerous matches to 15p, 14p, 22p, and 21p as censat (identity 94.7%-99.7%).

CCTCAGCCCACAGGGACTCTTGTGTCTCCTTCAGCTACTCTCAAAACTTCTCTAGATTCCAGCTGGAGTCAGTTCCAGGCACCCACGATACACCACCGAACTCACGAATTTCACTGACTTACTCCCCCGTCTCCCTCATGTTCTCACCCCCAGCCCTCAGGGACTCTTCTGTCTCTCTCAGCTACTCTCCAACCATCTCCAGATTTCACCTGGAGTCAGCTTCCCGCACCCAGCAATCACCTACAAACTCACGGACCTTACTGCAACCCTCCCCCATTTCTTTCACCTCTTCACCCCCTGCCTTCAGGGACTCTCCTGGGTCTCCCAGCTTCTCTCCAGCCTTCCCCAGATTTCTGCCACAGTCAGCCCCAGGCACCCAGGACAACCCTAGACACTCACAGGCCTCACGAGACTATCTCCCTATGACCTGTACCTATACAGGGATGGCTCCCACGCATCCCTCAGTGACCCCAAACCCATCTCCACTTACACTCAGGCACTCCCAGGGCCTGACAGCTACTCCCCATTATCGTCGTTCAGTTCGAAGCCCTGGCCAATCTATTAGCCCACATGACGCAGTTACCTGGCCATTTCTCCACGGTTCCCGTGAGGGCCCCACACCCAGGCGCACAAGAACCCCTCCTGCATTCCGTCCTCACACGCAGGCCTGTCCATCTACTTGCTACTGTCACACTCTTGCCAGCAGAAGAGGCCCCTGTAATGGCCGATATCACCACCCAGTCTATCCTCACCCCACAGCTGTGCAGCGGGAACCTCCTGCTGGCCCACGTGGTTGCCACAGCCCATGCTGGCACGACGCTCCAGCAGGTCGGCGTCCCTGCGGGCCACACTACCGGTGACATGGCTAGCATGACCCTCCTTCCTGGCAGTGACACTGTTGATGTGAACCCCAGTTTCACATCTGTCATTTGTAAATAGGACCATTTTCCCTTTTCGCTCTCCCTTCCATTCACAGGGCTTTTCATTCTCTCTGTTTCTGCCTCCGTTTCAGATATTTACTCACCTTTTTCTCTCTCACTATGTCTGCCGTGGTCTCCATGAGACTACGCCACGTAAGATTCCCCCATTAAAGGTCATGAATTGAGTGGCTTTTAGTATACCTGTGGTTGTGCACATTCAATTTTAATTCGCAATCCATTGTAAAACGTCTTATCACCCCCGACCAGAGAAAGACCCTGTAGACATTAGTCACTCCTCATTCTGTCTCAAACCCTCTCCCTGACCCTCAGCCCTAGGTAGCAACTACCTAGTGCGATCAATCCCATATGCATAGATTTCCATATTGTGGACATTTCCTATAAACGGAATTGCACAATAGGTGAGCTGCTATGACTGACATAACACGTAGCACAATATTTTCAAGATTCATCCACATTGTAGGCTTACCCACAGGGGGAAACCATTTTTTGGGGGGTTTTAGTAACACCGGTGTTTTCTCCTTCCTTTCGTCCTTCTTTCCTCCCTTCCTTCCTTCCTTCCTTCCGTCCTAACTTCCTTCTTTCCTTTTTCCTTCCTTCCTCCTATTTCTCTCTTACTCCTTCTGCCCTCTCTCTTTCATATGCCTTAGGTGCATCCCACATTCTGCGTTTTTTGGGGGAAATCCTCGACAGGTGCAGGAAAATTGTGTTATTGTAACTATTTACCGCTATCTCTCTTTCACGGCTCTCCATCACTTGTGAACATCTATTGGTTTATCCCAAGTCACTAAGCATATTTTTATTAGGTACACCTGTTTTTCCTTATACAGCTGTTTCTGGAGTGTAGGGTCGCATACTCATAAACCCAGTGTAACTCAGAAACGAATCTAATATTCCAATAAACCCATCATAACGTTGAAAAATCATAAATCAAACCATCATAAGTCACGGTTTGTCTGTGGATATGGGCGTCATCAATTCCGTTGTATTCAGTAATGCTGTACACCATTAACAATGGCAGACTGATTGGGAGTGGATATTGATAGCATTATAAGAGTCAGTTATTAGAGGGATACTTCTTTAACCTGACTGAAGAACTGATCTGATGGCTTTAGTACAGTGCATGATTACGTGAGATGTTTTGAGACAGAGTAGTACATTTGTGAATGAAATTTTATGGCTTTTTTTTCACTTAGTAGGAACCATTGTGTGTGGAAAAGTGAGAAAATTGCTTTCTGCTGTAGGGTCTGGCATTCATTGTAGATTTAAGCTTATTTTTCTGTGAGCAAATCTTATTCAATAAAATACTACTCTTTATACTAAAAAACAAAAACAGTGGTGATGTGTGGTCATTATCGTCAGCAAACTAATCCAGGGAAAGAAAACCAAACGCCACCTTCTCACTTATAATGGGAGCTGAAAAGTGAGATCCCATGGACACAGGAAGGGGAACAACACACACTGGGGCCTTTCGGGAGGCAGAGCGTTAAGAAAAACAGCTACTGCATGCTGGGCTTAATACCTAGGTGACGGGTTGACAGGTGCAGCAAACCACCATGGCACACGTTTACCTTAGTAACAAATCTGCACATCCTGCACATATACCCCAGAACTTAGAAACGAAACGAAACAAAAGAAAACGAAAAAGCAATAGCAAAACGCTAAAGGCAAAATAAAGTTTCAAACTCAGAAAGTGACAGACCAACTTTTGGTTCAAACCCAGGTGCCATAAGGTCAGGATAAAGAATTTGATTACATATTGTAAATAAGACATGCAGCAAATGACCAGAAAGATTATTCTCCACATATGTGTGTCTTCTAATTCAATGGTGACGCTATCTACCGGGACATAGCATTAGATTCCAAAGGGCCGAGTCCCGCCAGACAGGCCTCCCACACCAATAACAATGGGAAGCCCTAAGTTGTTTTACCTGTACTTCTCAGCAACTGGCTATAAATCAGGTTGCCACCACTTCCAGATTTAGTTGCATTCATTTGCTGGAAGAGCTCACAGCACGCAGGGAAACACTTACATTTGCCGTTGTATTTTAGCGGACATTGCACAAAGTTCAGAAATAAATGTGGGGCCCGCCATGCGGGGAGGGGCGCACTACCTTCCAGGAAGTGTTATCCAGAAGCTCTCTGAACCCAGTCCTTTTGGGTTTTGATGGAGACCTCATTCTATAGGCATGATGGGTTAAACCATAGGGTATTGGTGATCAACTCCACCTGAGGCTCTCCACCCTCCCTGGAAATTGGGGTTGAGGCTTTGCCATTCTCAGTCTGACTAAAAGAATTTACCCAAACGGAATTTTAAAACAGATGAGCATAACTGGAATCTTAATTAGATGATTGGATTATCTGGAGCCACACCTTGATATTCCTAACCCGAGCACCCTCATCCAACGAATGCTCCACCCAACTGGCTCCCAAGTCTCTACGTGGTTCCAGAGCAAAAGAATGTTTATACAACGCATATCTCCACCTTTTCTTCAAAGTCTTTTCGCTTACACGGAAAGACTTCTTCAACTGCCACGCTTCAGGGTCAGGGGGAGGTCTTGTTACAACACAGATCTGCGGATCTCCGGGGTTTGATTGTGGCAAGGATGCTGCTGGTGTCAAACCACAACGTGGGAAGCACAGAACCACTAGTTGGTTTTCAGTGTTTCAGTGCATACAATTCCTAATAAATCTGGCCAAGAAAACCTGTAAGTTCTTAGATTGTCCCAAAGGTGGCGCATGAAATCAAAGCAGGAGAACAGTTTCCTACGAGGTGTAGCCTGGGAAAGTTGGGGGTGACTGATGGAAAGGAGGAGTGAAGCTCCGCCCTTTCCGCTGCTAGGCTGCGCCCGAGGCTATCTAAACCCACCCTGGCTGGCCTGTACTCAGATCTTCGCGGAGCGGATCAGCGGCCGCAGCGTTTGGCGGACTCTGCGTGGACTTGGAGCTCACAGCGTCTTGCGACTTGGAAGCGGATTCAGAGGACAGGACAGAACACTTGGGCAAGTGAATCTCTGTCTGTCTGTCTGTCTCTCTGTCTCATTGGTTGGTTGATTTCCATTTTCTTAAGGGGCACATACCTCACACCGCACACACACAAGCACACACACACACACACGCACACGCACACACGCACACACACTCCTTCCTTCTTCGAGTTAGAACATTAGTAGGGGCCCCTGGGAGCTGCAGGTTTCCTAATCATGTCTGCACCTAAGAACAGTAGGGTCTTGTCTGGCTCTTCTTATGAACGGTCCCCCAGCACGGACACCCCAAGGTCCATGCGAGCCTCACCCAGCTTCTCCCTCTCCCCTCTCAGAAACTCAGGCTTAAGGGGAAGCTCCTCACCAGGGATCCGGAACTACCATTCACCATCCCCTAGGGCTTCACCACACTCACCTCTGTCATCACCAGAATCCCCCAAGCTCCCATTTCCCTGTCCTCACCGTGATGGGCAATCAATGAAGCCATTGGGCTCTGCCGTGTCCTCCTCTGAGGACTCCTCAGAGTCCCCACGTTCATCAATAATATACCACATGTTCTTACTGCCATCACCCAGCAGCTCACCCCCAGCTCTCGGGGGGTCTCCTGTGTCTCCCAGCTACTCTCCAAACAACCCCAGATTTCAGCTGGAGTCAGCCCCCCACACCCAGGAATCACCTACAAACTCACGAGCCTCACGCTGCTCCACCCCTGTGTCTTGCATCTCTTCACCCCCAGGCCTCAGGGACCCTCCTGTGGCTCCCAGTTACTCTCCAGCCATCCCCAGGTTCCTGCGGGAGTCAGCCCCATGCACCCAGGAGTCCCCCAGAGACTCACAGGTCTCGGGAGAATATGAGCGGTCCCCCAGCCCTGACTCCTCAAGATTCATGCCTGCCTCACCCAGCTTCTCCCTCTCCCCTCCCAGAAACTCAGACCCAAGGGGCAGCTCCTCACCAGGGATGTGGAAGTCCTCTACATCATCCCGCAGGGCTTCACCACTCTCA

**Supplementary File 3B. GS16-RC21**

**Breakage-fusion sequence for GS16-RC21 (microhomology)**

**21p13(+)** 2714941-AACCCTATGTGGGGGACAATCA**C**CCAGAACACATCCGTAGT-2714981

**21q22.2(+)** 39159770-CTTCAGGCTGCTGTCATAACG**C**AGTTCTGAGCTGCAATGAT-39159810

**Fusion sequence:** CTTCAGGCTGCTGTCATAACG**C**CCAGAACACATCCGTAGT

**ISCN:** seq[T2T] r(21)(p13q22.2) g.(pter)_2714962del::39159790_(qter)del

**a. CNV analysis showing a 2.71 Mb distal deletion of 21pter-21p13 and a 5.93 Mb distal deletion of 21q22.2-qter**


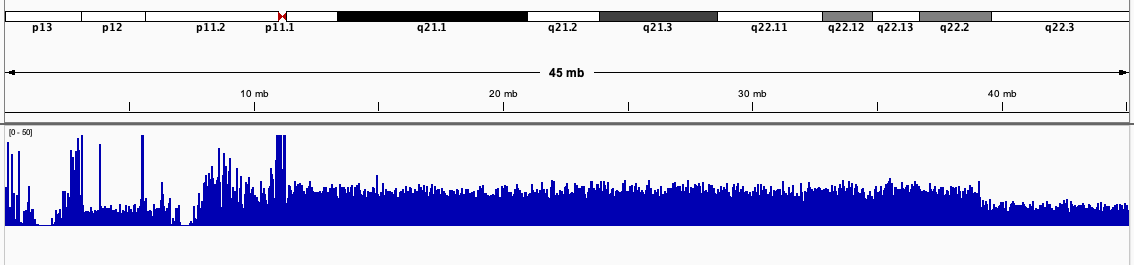


**b. A closer examination of the 21q22.2 region revealed the soft-clipped reads.**

chr21: 39159791 (right-clipped sequences)

**
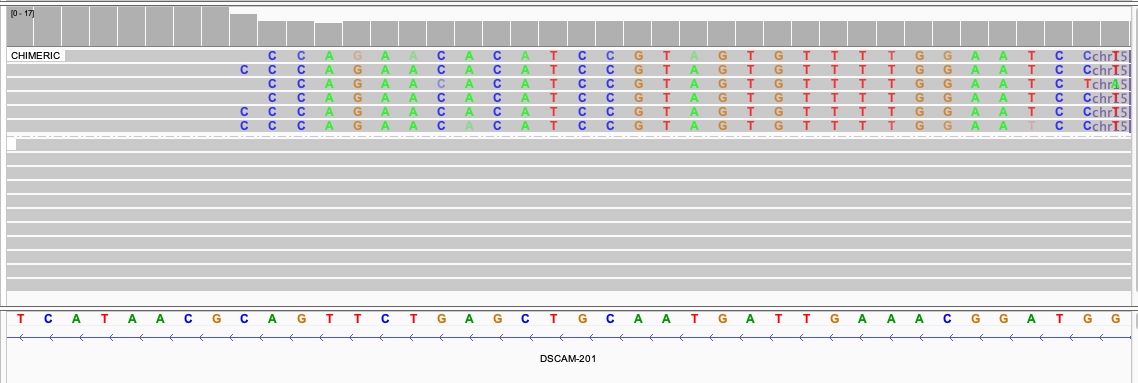
**

**c. BLAT search results in T2T**

The following sequence is the soft-clipped region directly extracted from IGV- chr21: 39159791 connect to 21p. Blat this 5.62 kb to T2T show a match to chr21:2714963-2753181 (+) (identity 93.1%) at 21p13, which is classified as other centromeric satellites (censat_21_16). There is a junction of 21p13(+) with 21q22.2(+) mediated by a microhomology sequence ‘C’. Also there are numerous matches to 15p, 14p, 22p, and 13p as censat (identity 80.2%-98.1%).

CCCAGAACACATCCGTAGTGTTTTGGAATCCTATCTGAGGGACAAATATTCAGACCCTAGTAGCACTGTTCAGGAATCCTATGTGAGGGACAAACATTCAGACAATCGTAGCATTGTTCTGGAATCCTATGTGAGGGACAAACATTCAGACCCCAGCAACAGTGTTCTGGAATCCTATGTGGGGTTCAAACATTCAAACAACAGCGGGATTGTTCTGGAATCCTATGTTACGGACAAACATTCAGACCATCGTAGCAGTGTTCTGGTATTCTATGTGAGGGAAAAACACTGAGAACACAGCAGCAGTGTTCTGGAATCCGATGTGTGGGACAAACATTCAGACCCCAGCAGCAGTGTTCTTTAATCCTATTTGAGGGACAATCATTCAGACTCTCATAGCTGTGTTCTGGAGTTCTGCATGAGGGACAATCGTTCAGACCCACGTAGCAGTGTTCCGGAATTCTGTGTGATGGAGAAACATTCATTACTTCATAGCAGTGTTATGAAAGCCTATCTGTGGGACAAACATTCAGACCACAGCAGGAGTGTTCTGGAACACTAAGTGTTCTCAAATCCTATGTGAGGAATAAACATTCAGACCCTCGCAGCAATGTTCTGGAATCCTATGTGACGGACAAACATTTGGACTGCAGCAGCAATGTTCTGGAATCCTATGTGAGGGACAAACTTTCAGAAAATCGTTGCAGTGGTCTGGTATCCTAGGTGAGGGACAAACATTCAGAACCCAGCAGCAGTGTTCTGGAATCCAATGTGAGGGACAAACATTCAAGCAACAGAGGGAGTGTTTTGCAATCCTAGGTAAAGGACAAATATTTAGACAACAGGAGCAGTGTTCTGGAATCCTGTGGGAGGGACAAACATTAGGACCCTCAGTATAGTGTTCTAGAATCTTATGTGAGAGAAAAACATTGAGACAATCATAGCAGTGTTCTAGAATCCTTTGTGACGGACAAATATTTAGACCCCAGCAGCATTGTTCTGGAATCCTATGTGAGTGACGAACATTAAAATCACAGCGGGAATGTTCTGGAATCCTTTGTTACGGGCAAACATTCAGATCCTTGTCGCAGTCTTCTTGAATTACATGTGAGGGAGAAACACTCAGAATCCACCAGCAGTGTTCTGGAATCCTGAGTGACAGACATACATGCAGACCTCAGTAGCAGTGCTTTGCGATCCTATGTGAGGGACAAACTTTCAGACGGCAGCAGCAGTGTTCTGGAATCCTGTGTGAGGGACAATCATTCAGACCCTCGTAGCTGTGTTCTGGAATTCCGTGTGATGGACAAACATTCAGACCCTCATAGCAGTGTTCTGGAAACCTATGTGAGGGACAAACATTCAGACCACAGCAGGCATGTTCTGGAATCCTCTGTGAGGAAGAACCATTCAGACCCTCGTAGCAGTGTTCTGGAATCCTATATGACAGACAAACATTTAGACCCCAGCAGCAGTGTTCTGGAATCCTGTGTGAGGGACAAACATTCACACCCTCGGAGCAGTGTTCTGGAATCCTATAAAGGGACAAACACTCAGAACCCAGCAGCCGTGTTCTGGAACTATATGTGAGGGACAAACACTGAGAACACAGCAGGATTGTTTTGGAATGCTATGTGACGGAGAAACATTCAGGCACTCGTAGCAGTGTTCTGGAATCCTAAGTGAGGGACAAACATTCAGACAATCGTAGCAGTGTTCTGGAATCCTATGCTAGGGAAAAACATTCAGACCCCAGTAGCAGTGTTCTGGAATCCTATGTGAGGGAAAAACATTCAAACAACAGCGGGAGTGTTCTGAAATAGTATGTTACTGACAAACATTCAGACCCTTGTCACAGTGCTCTGGAATTCAAAGTGAGGGGGAAACACACAGAACCCAGCAGCTGTGTTCTGGAATCCTGTGTGACGGACATACACATAGACCCCAGCAGCGGTGTTCTGGAATCCTATGTGACGGACAAACATTCAGACTCCAGCAGCAGTGTTCTGGAATCCTCTGTGAGGGACCATCATTCAGACTCTCGTAGTAGTGTTCTGGAATTCTGCGTGAGGGGCAAACATTCAGACCATCGTGGCAGTGTACTGGAATCCTGTGTGAGGGATAGTCATTCCGAACACAGCAGGAGTATTCTGGAATCCCCTGTATGGAAAAAACATTCAGACCCTCGCAGCAGTGTTCTGGAATCCTAGGTGAGGGACAAACATTTAGACCCCAGCAAAAGTGTTCTGGAATCCTGTGTGAGGGACAAACATTCAGACTTTCATAGCAGAGTTCTGGAATCCTATCTGAGGGGCAAACACTCAGAAACCAGCACCAGTGTTCTAGAACCCGATGTGAGGGACAAACATTCAGACCACAGCAGGATTGCTTTGGGATCCCATGTGTGGGAAAAACAGAAAACAGCGGGAGTGTATTGGAATCCTATGTGAGGGACAAAATATTCAGACCCTCTTTGCAGTGCTGTGGAATCCTATGTGAGGGGCAAACTTTCAGAAAATCACAGCAGTGGTCTGGAATCTTATGTGAGAGACAAACATTCAGAACTCAGCAACAATGTTCTGGAATCTTATGTGAGAGACAAACATTCAGAACTCAGCAACAATGTTCTGGAATCTTATGTGAGAGACAAACATTCAGAACTCAGAACCAATGTTCTGGAATCCTATGTGAGAGACAAACATTCATACAACAGCGAGAGTGTTCTGCAATCCTAGGTAATGGACAAACATTTAGAAACCAGCAGCAGTGTTGTGGAATCCTATGAGAGGCACAAACATTCAGACCTTAGTAGAGTTATTCTGGAATCCCATGTGAGGGACAAACATTCAGAACCTCGTCACAGTTTTCTGGAATTCTGTGTGAGGCAGAAACACTCTGAACCCAGAAGCAGTGTTCTGGAATCCTCTATGACAGACACACATGTAGACCTCAGCAGCTGTGTTCGGGAATCCTACGTGAGGGACAAGCATTCAGACCGCAACAGCATTATTGTGGAATCCCAAGTCAGGGACAATCATTCAGACCCTCGTAAGAGTGTTCTGGAATTTTGTGTGAGAGACAAACATTCAGACACTCATAGCGGTGTTCTGGAAAGCTATGTGAGGGAGAAACATTCAGACAACAGTAGGCGTGTTCTGGAATCCCCTGTGAGGAAAAACATTCATACCCTCGTAGCAGTGTTCTGGAATCCTATGTGATGGACAAACATTTAGACCCCATCAGCAGTGTTCTGGAATCCCGTGTGAGCAAAAAACATTCAGACGCTAGTAGCAGTGTTCCGGAATCCTATGTAGGAACAAACACTCAGAACCCAGCAGCACTGTTCTGGAACGCTATGTGAGGGACAAAGATTCAGACCACAGCAGGATTATACTGGAGTCCTATGTGAGGGACAAACACTCAGAACGGAGCAGGAGTGTTTTGGAATCCTATGTGTCAGACAAACATTCAGACACTCATAGCAGTGTTCTGGAATCCAATGCAAGAGACAAACTTTCAGACCACAGCAGCAGTGTTGTGGAATCCTCTGTGAGGAACAAACTTTCAGAATATCGTAGCAGTGTTCTGGAATCCTATGTGAGGGACAAACATTGAGATCCCAGCAGCAGTGTGCTGGAATCCTATGTGAAGGACAAACATTCAAACAACAGCGGGAGTGTTCTGGAATCCTAACAAGTAGACACATTTAGACACCAGCAGCAGTGTTCTGGAATCCTATGTGAGGAAAAACATATTCAGACCCTCGTAGGGTTATTCTGGAATCCTATATGAGGAACATATATTCAGACCCTCGTAGGATTATTCTGGAATCCTATGTGATTCAGACACCAGGAGCAGTGTTCTGGAATCCTATGTGTGAGACAAACATTCAAACAACAGTGTGAGTGTTCACGAATCCTATGTGACGGACAAACATTCAGACCTTCCTCTCAGTGTTTGGAATTCTATGTAAGGGAGAAACACTCAGAACCCAGCAGGAGTGCTCTGGAATCCTATGTGACGGACATACATGTAGACCCCAGCAGCAGTGTCCTCGAATCTCATGTGAGGGAGAAACATTCAGACCCCAGGAGCAGTGTTCTGGAATCCTATGTGAGGACAATGATTCAGGACCTCGTAGCAGTGTTCTGGTATTCTGTGTGAGGTACAAACTTTCAGACTCTCTTACCACTGTTCTGCAATCATGTGTGAGGGAAAAACATTCAGACCCACAGCAGGAGTGTTCTGGAATCACCTGTGAGGAACAAACATTCAGATCCTCCTAGCAGTGTTCTGGAATCCTGTGTGACGGACAAACATTTAGACGCCAGCAGCAGTGTTCTGGAATCCTAATTGCGGGACAATCATTCAGACCCTTGTAGCCGTGTTATGGAATCCTTTGTGAGGGACAGACATTTAGAACCCAGCGACAGCATTCTGGAACACTATGTGAGTGAGAAACATTCAGACCACAGCAGGATTGCTCTGGAATCCTATGTGAGGGACTAATATTCAGACCTTCGTAGCAGTGTCCTGGAATCCTAAGTGAGGGACAAACATTCAGACAATCGTAGCAGTGTTCCAGAATCCTATGTGAGGGACATTCATTCAGACGTTCGTAGCTGCGTTGTGGAATTCTGTGTAAGAGCCATATATTGAGATCCTCAAAGCCGTGTTCTGGAATCCTACGTGAGGGACAAACATTCAGACCACAGGAGGATTGTTCTGGAATCCTCTGTGAGGAAAAAACATTCAGACCCTCGTAGCAGTGTTCTGGAATCCTATGTGAGGGACGAACCTTTAGACCCCAGCAGCAGTGCTCTTGAATCCTACGTGAGGAAAAAACATTCAGACACTCGTAGCATTGTTCTCGAATCACATGTGAGGGACAAACATTCAGATAATCTTAGCCATGTTCTGGAATCCTATGTGAGGGACAAATATTCAGACCCCAGCAGCGTTCTGGAATCCTATGTGAGGAACAAACATTCAAACAACAGCGGGAGTGTTGTCGAATCCTATGTAACAGACAGACTTTCAGAACCTCGTCGCAGTGTTCTGGAATTCTGTGTTAGAGAGAAACACTCAGAAACCAACAGGAGTGTTCTGGAATCCTATGTGATGGACATACATGTAGACGTCAGCAGCAGTGTTCTTGAATCATATGTGAAGACAAACATTCAGATCTCAAAAGCAGTGTTCTGGAATCCTCTGTGAGGGTCAATCATTCAGACCCTTGTAGCAGTGTTCTGGAGATGTTTGTGAGGGAGGAAGCTTCAGACCCCCATAGCAGCGTTCTGGAAGCCTATATGAAGGACAAACATTCGGACTAGAGCAGGATTGTTCTGGAATCCAATGGGAGGAACAAATATCCGGACCCTCGTAGCATAGTTCTGGAATCCTATGTGACGGACCAACATTTAGAGCCCAGCAGCAGTGCTCTGGAACCTCAAGTGAGGGACAAACATTCAGACACTCAGTGCAGTGTTCTATAATCCTATATGAGGGACAAACATTGAAACAATCGTAGTAGTGTTCAGGAACCTATGTGACGGACAAACATACAGACCCCAGCAGCAGTGTTTTCGAATCCTATGTGGGGGACAAACATTCAAACAAC

**Supplementary File 3C. GS9-RC22**

**Breakage-fusion sequence for GS9-RC22 (microhomology)**

**22p11.2(+)** 10069210-TTTTTCACCGATGGCCCA**AAA**CCGCTCAGAAATATCCCTTT-10069250

**22q13.2(+)** 44191310-AGCCCTGGCCACCCTCAACCCCC**AAA**ACACGCATTCTAT-44191350

**Fusion sequence:**  AGCCCTGGCCACCCTCAACCCCC**AAA**CCGCTCAGAAATATCCCTTT

**ISCN:** seq[T2T] r(22)(p11.2q13.2) g.(pter)_10069227del::44191332_(qter)del

**a. CNV analysis showing a 10.07 Mb distal deletion of 22pter-p11.2 and a 7.13 Mb distal deletion of 22q13.2-qter**

**
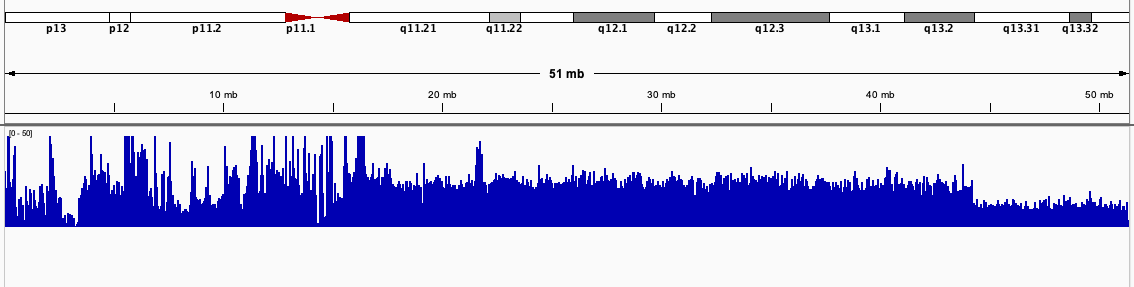
**

**b. A closer examination of the 22q13.2 region revealed the soft-clipped reads**

chr22: 44191350 (right-clipped sequences)


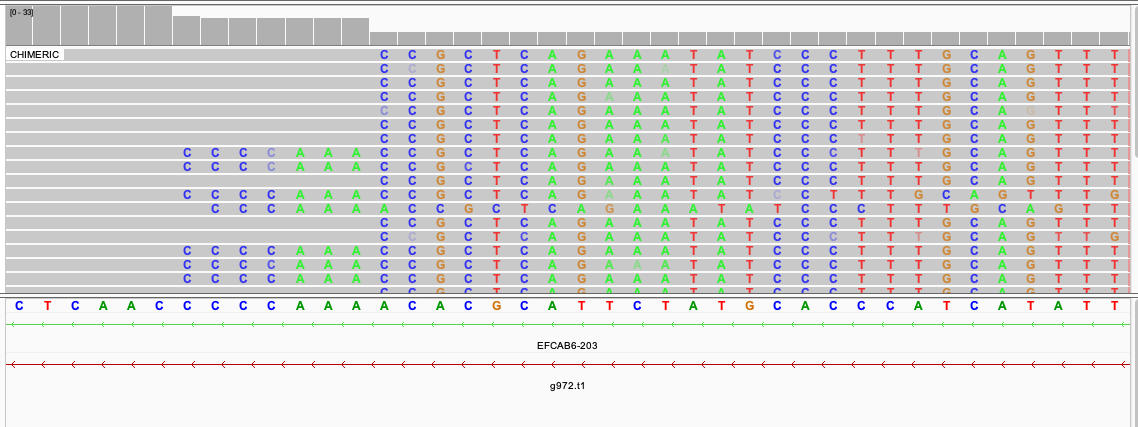


**c.** **BLAT search results in T2T**

The following sequence is the soft-clipped region directly extracted from IGV- chr22: 44191350 connect to 22p. Blat this 1.04 kb to T2T show a match to chr22:10069231-10070265 (+) (identity 98.4%) at 22p11.2, which is classified as inactive α-satellite higher order repeat (hor_22_6, S6C22H2-B). There is a junction of 21p11.2(+) with 21q13.2(+) mediated by a microhomology sequence ‘AAA’. Also there are over 30 matches to 22p (identity 97.2%-98.2%) from chr22:8697409-1012630.

CCGCTCAGAAATATCCCTTTGCAGTTTGTAGAAAAAGACTGCTTCCAAACTGCTCAATGTAAGGAAATTGCCAACTATTAGAGATGAATGGAAATGTCACTAAGAGTTTTCTCAAAAAGCTACTGTGTCGTTTTTATGTGAAGACATTGCCTCTTGCACCCTAGGCCTTAAAACTCTCTAAATACACATTCACAGATTCTACAAAAAGACTGATTCCAAACTGCTCAATCAGAAGAAGGGTTCAATTCCGTGTGACAAACGTGCACATCACCAAGAAATTTGTCAGAAAGCTTCTGTCTACTTTTTATGGGAAGATATTTCATATTTCAACAAAGGCCATAAAGGACTCACAATTATCCCTTCGCAGATTCTAAGAAAAGACGTTTTCCAAACTCCTCAATCAAAAGAAAGGTTTAACTCTGTGAGATGAATGGACACATCACGAAGAAGTTTCTCAGAAAGCTTCTGTCTAGTTTTTCTGTGAAGTTATTTCTTTTTCACCATAGGCCTCAAGCAGCTAAGAAATTTCCCTCTGCAGCTTCTACCAAAGACTGTTTCCAAACTGCTCACCTGAAAGGAAGGTTGAATTCTGTGACATGAATTCACACATCACAAAGAGGTTTTTCAGAAATCTTCTGTCTGGTTTTAAGGTGAAGATACTTCCTTTTTCACCACGGGCCTCAAATATCTCCAAATATCCATTTGCAGATTCTACAGAAAGACTTTGCAAACTGCTCAATCAAAAGAAAGGTTCAACACTGTGAGATGAAGGCACACATCACCCAAGAAGTTTCTCAGAAACCTTCTGTCTAGATTTTAGGTGAAGATACTTCATATTTCACCACAGGCCATAAAGGGCTCACAAATATCCCTCTGCAGGTTCTACAAAAAGACTGTTTCCAAACTGCCCAATCAAAGGAGAGGTTCAACTCTGTGACGTAAATGGACACATCACAAAAAACTTCTTGGAATGCTTCCGTCTAGTTCTTATGGGAAGATATTTCTCTTTCACCAGAAGCCTCAAACGGATCAGAAT

**Supplementary File 3D. GS14-RCY**

**Breakage-fusion sequence for GS14-RCY (non-template insertion)**

**Yp11.2(+)** 845760-TCCTCTCTTCTGTTTGGACCTCTCTGCTTGTGTGTGTCTGT-845800

**Yq11.221(+)** 17078520-TGAGATGAGGAAAGAAGTTTAGCCATTGGACATAAGGAC-17078560

**Fusion sequence:** AAGTTTAGCCTAAACTTCCCAAGAGTTGCTAAAGACTTCTTCTCTGCTTG

**ISCN:** seq[T2T] r(Y)(p11.2q11.221) g.(pter)_845779del::ins30bp::17078543_(qter)del

**a. CNV analysis showing** **an 8.46 kb distal deletion of Ypter-Yp11.2 and a 45.38 Mb distal deletion of Yq11.221-qter. Below, IGV view of the chimeric alignments in split screen.**


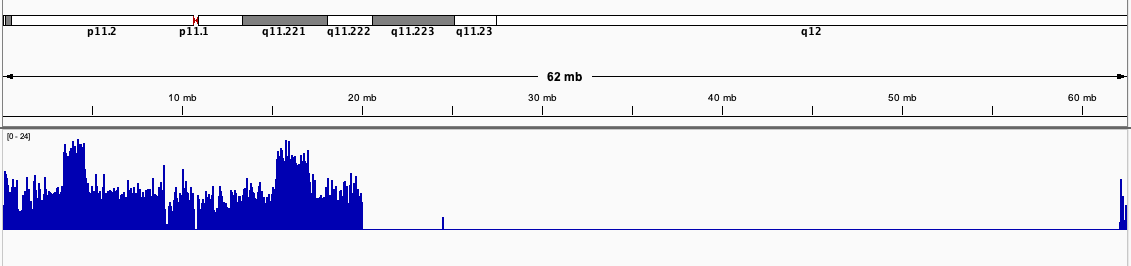


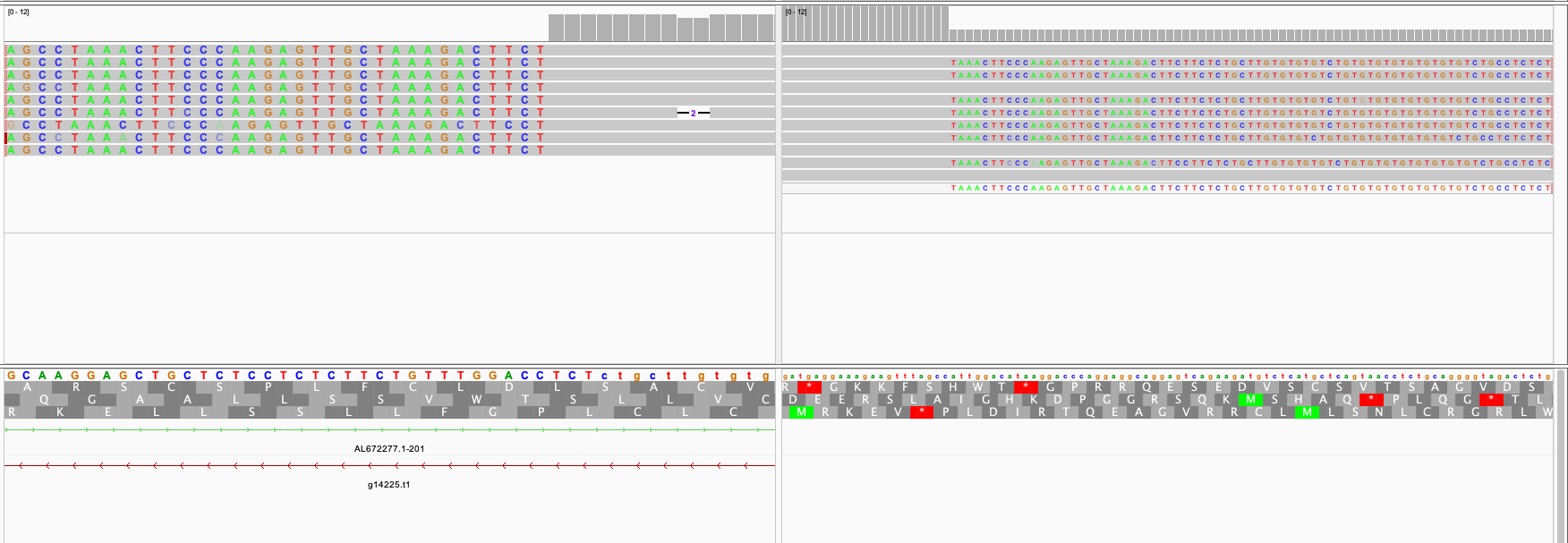


**b. BLAT search results in T2T**

The 5.066 kb sequence extracted from chrY:17078545-right-clipped sequences matches chrY:845780-850805 (identity: 99%) at Yp11.2 with a breakpoint at intron 1 of the *AL672277* gene for IncRNA. A 30 bp insert sequence (**bold**) was noted.

**TAAACTTCCCAAGAGTTGCTAAAGACTTCT**TCTCTGCTTGTGTGTGTCTGTGTGTGTGTGTGTGTCTGCCTCTCTCTCTCTCTCTCTCTCTCGCAAGGAGCTGCTGTCCTCTCTCCTTTCTTCTGTTCAGACCTCTGTGTGTGTGTGTCTCTCTCTCTCTCTGTCTCTGTGTGTGTCTGTTTCTCTCTGTGTGTGTCTCTTTGTATCTGTGTGTTTGTGTGTGTCTGTCTCTCTGTCTCTGTCTGTCTGTCTGTCTTTCTCTCTCTCTCTCTCTCTCTCTCTCGCAAGGAGCTGCTCTCTTCTCTTTTCTCCTGTTTGGACTTGTGTGTGTGTGTCTGTCTCTCTCTGTCTGACTGCCTCTCTTTGTCTCTGTCTCTCTATCTCTCTGTCTGTCTCACAAGGAGCTGCTCTCCTCTCTCCTTTCTTCCCTGTCCTCAATTATTTCTCAGAGTGAGACAAGGAACTCCAGGGTATACACCCCGCACGACGTAGCTGTTTCCAACACCAGGGGTCTTAGCTAGAAAAAGTGAGTGTATGTAATCGTCAGCATTTGCTACTGATGACAGAATAGAACAAATTCTGCCAAGAGATTTAAAGAGGTTTATTCTGAACCAATATGAGTGCTGAGCGCCTGTGCTCCAGGATTACATGGTCTCAAGAGATCTGAGAAAGTGTGTTCAGGAGGTCTGGTTACAGTTCCATTTTTATTTATTTATTTATTTTTATTTTTTTTAAATCGAGATGGAGTCTCACTCTGTCATCCAGGCTGGAGTACAATGGCGTGATCTCGGCTCACTGCAACGTCTGCCTCCAGGGTTCAAGCAATTTCCCTGCCTCAGCCTCCCGAGTAGCTGGGATTACAGGCCCCTGCCACCACACCTGGCTAATTTTTTGTATTTAGTAGAGACGAGGTTTCACCATATTGGCCAGGCTGGTCTCAAACTCCTGACCTCAGTTGATTCTCCCACCTCGGCATCCCAACATACTGGTATTACAGGTGTGAGCCACCGCGCCCGACCTACAGTTCTATTGTATATATTTCAGGGAGACAGGAATTGTAGGTAAAATCATAAATCATTACATGGAAGGTATACCTTGTTTCGGCCTTAAAGATGAAACATCTCAAAGTGGGTTCCTACGAGTCACAGGTAGGCTTTAGGGATTCTTTAGTTGGCAATTGGTTGTATAAAACATAAAAATGTAAGGCCGGGCATGGTGGCCCACGCCTGTAATCACAGCACTTTGGGAGGCTGAAGCGGGTGCATCATCTGAGGTCAAGAGTTTGAAACCAGCCTGGCCAATATGGTGAAACCCTGTCACTACTAAAAATACAAAAATTAGCTGGGCCTGTAATCCCAGCTACTTGGGAAGCTGAGGCAGGAGAAACACTTGAATCCAGGAGGCAGAGGTTGCAGTGAGCTGAGATTGTGCCACTGCACTCCAGCCTGGGTGACAGAGTGAGGAAAGAAAAGAAAGGAAGGAAGGAAGGGAAAGACAAAAAGAAAAGAAAGAGAAAGAAGGAAAGAAAGAAAAAGGAAGGAAGGAAGGAAAAATAAAAGGAAGAGAAACAAAGGAAAGAAAAAAGGGAAGAAAGGGAAGGGTAAGGAAGAGAAGGGAGAGGGGAAAGAAAGGAAGAAAGGAATGAAAGAAAGGGAAAGGGATAGAAAGGAAGAAAGGAATGAAAGAAAGGGAAAGAGGGAAGGAAAGAAAGAAAGAGAAAGAAAGAGAAGGAAGGAAAGAGGAAGGAAAAGGAAGGAAGGAAGGAAATATTTTGTAGACGCAGTGATATGGTTTTGCTGTGTCCCCACCCAAATCTCAAATTGCAGCTCCCAAAATTCCCACGTGTCATGGGACTTACCCAGTGGGAGGTAACTGAATCACAGGGCAGGTCTTTCCCTTGCTGTTCACACAATAGTGAAGACGTCTCATGAGATCAGATGGTTTTATAAAGGGGACTTTCCCCTTTTCTCTTGCTTGCCACCATGGAAGACATGACTTTGCTCCTCCTTTGCCTTCCGCCATGATTGCGAGGTCTCCTCAGCCATGTGGAACTGTGAGTCCGTTAAACCTCTTTCCTTTATCTTTTACCCAGTCTCGGGCTGGGCGCGGTGACTCATGCCTGTCATCCCAGCACTTTGAGAGGCCGAGGCAGGAGGATCACCTGAGGTCGGGAGTTCAAGACCAGCCTGCCCAACGTGGAGAAACCCTGTCTGTACTAAAAATACAAAACTAGCAGGGCGTGCTGGCACGTGCCTGTAATCCCAGCTACTCGGGAGGCTGAGGCAGGAGAATCGCTTGAACCCGGGAGGCGGAGGTTGCAGTGAGCCGAGATGGCACCATTGCACTCCAGCCTGGGTGACAAGAGCAAAACTCCATCTCAATCAATCATCAATCAATCAATAACCCAGTCTCAGGTATGTCTTTATTAGCAGCCTGAGAACAGAGTTATATACACAGAGTCTTGCTATGTTGCCCAGGCTGGTCTCGAACTTCTGGGGTAAGTGATGCTCCCACCTCAACCTCTCAAAGTGGTGGGATTACAGGCGTGAGCCACCGCGCCCGGCCTCCATGAGTCTGATTTTAACCCATGTCTTTTGTATTTCTCAGTCCTTGTCCACACATCTCATCACACACGTGCGCAGCAAACAGGCACACGCAGTGTGAACCCAGGCACAGCTCTCCGAGAGAACTCTAGTCAACACGTCGTCCGTGGGACATCGGCTGTATTTCTGTTCCTTTCCTTCTTTTCTGTCTCTTTCTCTTATTTCCCAAAACTCCGTTGTGTTTCTGATTTGTTCACATCGTTTTCGGCAAAAGGAAAACACAATGGTAAGTGGGGTTTATAAATCTATCTTCGTTATCAAGGGAAGATTGAAACGGCATGGAGACAAATGAAATTAACTGTGCACGGGACACACCGTCAACAAAACGCTAATAAGTGCAGAAAGACTGTTTAGCAGGAAACATGTCCAAAAAAAAAAAAGAAAAGAAAGGATCTTAATCATGGTTTAAGCTTATCTGATGGATTTCAACAGAGACACATCAAGTCTAATTGAATGTTTACGTGCACTTTTTATGGTTTCTGTATTCTGTGTAATGAAATAACATTACAAAAAAGCGTTGAGACGCAAGAAACTCTGGGATTCTTCTAAAGGGAGAGTTGGAGTTACTACATTCAACATGGACGGCTTCTCTTCTGTATTTCAACAGCGTTATCGTCTGCAAATTTGGAGATTCTCTGAAAGCTTCACGGCCATCCTGGGTCTCAAACTCTTTGAATATTCATACTGTAAACTATCTGACAACATGGGATTGGGTTTTGGCATTCTCTAGGTCTACTGAGAGAGGTTCTGGAATGCAATTTAACTTTAAATGCCTACCTACCTACCTGCCTATCATCTATCTATCTGTCTAACATCTATCTGTCTTTCAATCATCTATCCATTCGTCTCATCTATCTACCTACCTATCTATTCATCCATCTCATCTATCCATCCATCCATCCATTCATCCTGCCATCTATCCATCTATGTATCTATCTATCTAGTCATCCATCTCATCTGTCTTCCTATCCATGTATCATCTATCTACTTACCTATTAACTATCATCTATCAATCAAGCTATCTATATATCCATCATCTATCTACCTTATCTATTATCAATCTATCGTGTGTCCATCTATGTACTATCACCTGTCATCTGTCATCTACCATCTGTTTATCTACCCATCTCATCATCTATCAACTACCCATCCATCTTTATCTATCTATCTATGTATCTATCTATCTATCTTTTTTTTTTTTTTTTTTTTTTTGAGACAGAGTGTCACTCTGTCGCCCAGGCTGGAGTGCAATGGCGCAATCTTGGCTCACTGCAAGCACCGCCTCCCAGGTTCACGCCATTCTCCTGCCTCAGCCTCCCGAGTAGCTGGGACTACAGGCGTCCGCCACCATGCCCGGCTAATTTTTTGTATTTTTAGTAGAGATGGGGTTTCACCGTGGTCTCGATCTCCTGACCTTGTGATCCGCCCGCCTCGGCCTCCCAAAGTGCTGGGATTACAGGCGTGAGCCACCGTGCCTGGCCCTATCTATCTATCTATCTATCTATCTATCTATCTATCATCTATCATCTATCCATCCATCACATCTCTTTATCTACCCATTCATCTTATTTATTTGTCTATTTATGTATAATCGGTCTATCCATTTCATCTATCTATCTATATACCAACCTATCATCTATCTATCCATTCATCTATGTATATCTATCTATCCATCTATGTGTCTATCTACGTATCTATTATCTATCTATCCACTATCACTAATCTATCATCTATATATCCACCTATATCATCTATCATCTCTCGAGTCATGTCATGTATCATCCATCTATGTATCTATGTATCTGTGTATGTATGTATGTATGTATGTATGTATGTATGTATGTATGTATCTATCTATCTATCTATCTATCATGAACCCAAAATATCTGAGACAGGTGTCAATCAATTTAGCAGGTTTATTTTGCCAACGTTAAAGACACACCCATGAGACGGCCTCAGGAGGTCCTGACGACAGGTGTTCAAGGTGGTTGGGTCACAGCTTGGTTTTATACATTTTAGAGAGACATGAGACATCAATCAATGTGTGATACTGTAAGTTGTGCATTGGTTTGGTCCGGAAAGGTGAGACAGCTCAAAGTGAGGAGGGGGCTTATAGGTGATAGGTAGGAATTTCCTTGTGGACAGATTGTGAGGGACATATGTGATCTTTGTATCTCTGTAGCGATCTTATTTACGAATAAAATAGGAGGCAGGTTTGCCTGATGCCGTTTCCATATTGACTTTTTCCTTTGGCTTAGTGATTTGGGAGTCCTAAGATTTATTTTTCTTCCTATCAATCTATCTATCCATCCATCCATCTATTCATCCTTCCATCTATCCGTTTATGTATGTATTATGTATCTATCTATCTATCTATCTATCTGTCCATCCATCTATTCATGTATGTATGTATGTATGTATGTATCTATCCACCCATCCATCCAGCTATTCATGTATGTATGT
