# Supplementary material for: Unravelling ring chromosome structures and formation mechanisms by short-read and long-read genomic sequencing
Source: Genet Med Open. 2025 Nov 19;4:103475. doi: 10.1016/j.gimo.2025.103475 (PMC13207348; doi:10.1016/j.gimo.2025.103475)
Supplement: Supplementary File 4 [file mmc5.docx]

**Supplementary File 4. Incomplete RCs with complex rearrangements**

**A. Breakage-fusion sequences for GS3-RC13.**

**B. Breakage-fusion sequences for GS4-RC13.**

**C. Breakage-fusion sequences for GS15-RC14.**

**D. Breakage-fusion sequences for GS10-RC22.**

**E. Breakage-fusion sequences for GS17-RC22.**

**F. Breakage-fusion sequences for GS12-RC13.**

**Supplementary File 4A. GS3-RC13**

**Two-break and two-fusion for GS3-RC13 (microhomology)**

**13p11.2 (+)** 10407780-AATGTGCATCCCTTTTATT**GTTT**TTCTCTTGTCTGAATTGC-10407820

**13p11.2(-)** 10407780-TTACACGTAGGGAAAATAA**CAAA**AAGAGAACAGACTTAACG-10407820

**13q33.1 (-)** 101308860-GATTTTCTTTAAGAATCGGGGATA**TTTG**TGTAAAATGTTAG->101308900

**Fusion sequence 1 (f1):** <-GATTGTAAAATGT**GTTT**TTCTCTTGTCTGAATTGC

**13q33.1 (+)** 102776780-ATAAAGAGGATCACATGG**TGGGA**ATGCAGTGTCCTCAAAAG-102776820

**13q33.1 (-)** 102769840->TGTTACGGGGACCGGTAGGTCTT**AGGGT**ACTCAAATGGTGG-102769880

**Fusion sequence 2 (f2):** ATAAAGAGGATCACATGG**TGGGA**TCTGGATGGCCAGGGGCATTGT<-

**ISCN:**

seq[T2T] r(13)(p11.2q33.1) g.(pter)_10407798del::101308888_102769861dup::102776802 _(qter)del

**a.** **CNV analysis showing a 10.41 Mb distal deletion of 13pter-p11.2, a 1.461 Mb duplication at 13q33.1, and a 10.79 Mb distal deletion of 13q33.1-qter. Below, IGV view of the junctions.**


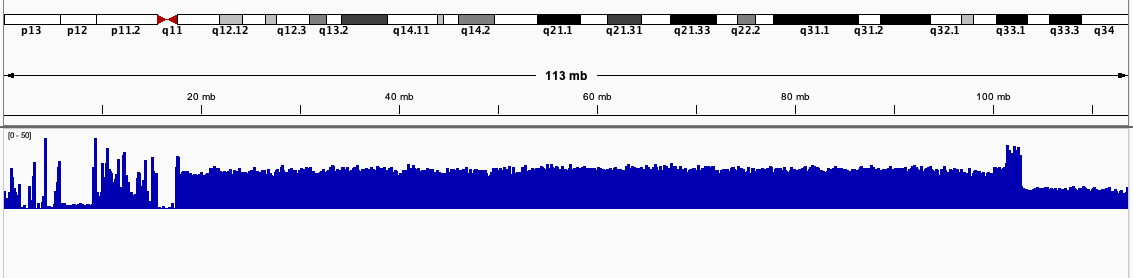


Chr13:101308886-left-clipped sequence for **f1**.


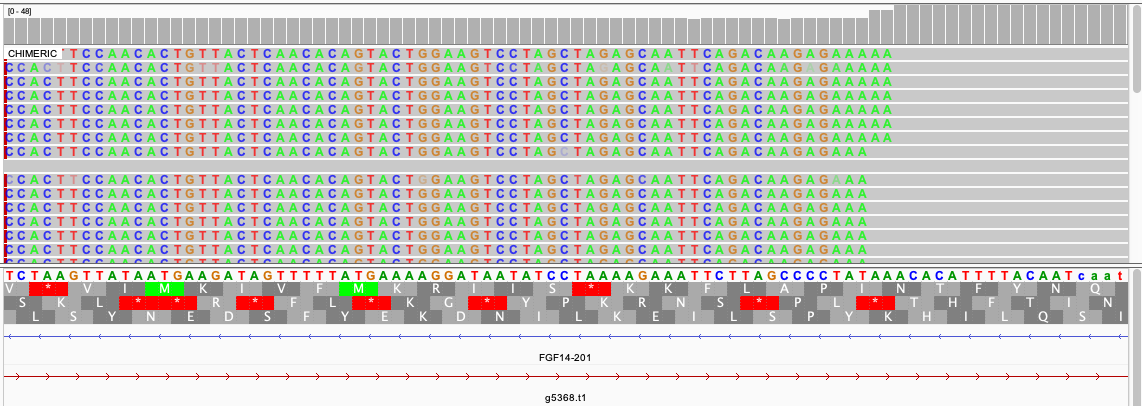


Chr13:102776802-right-clipped sequence for **f2**.


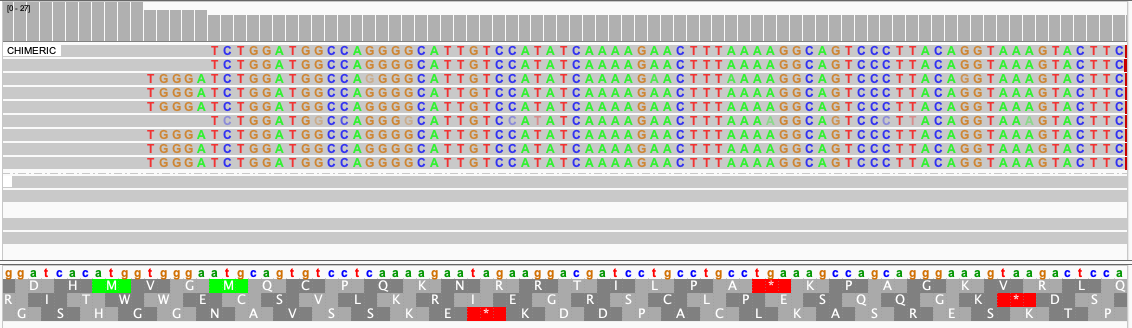


**b. BLAT search results to T2T**

Two fusions were detected by lrGS. An 8.6 kb chr13:101308886-left-clipped sequence matches to chr13:10407800-104163999(-) at 13p11.2 (identity: 100%). The fusion 1 (f1) of 13p11.2(+) and 13q33.1(-) is mediated by a microhomology sequence ‘GTTT’. A 3.275 kb chr13:102776802-right-clipped sequence matches to chr13:102766587-102769861(-) at 13q33.1 (identity: 100%). The fusion 2 (f2) of 13q33.1(+) and 13q33.1(-) is mediated by a microhomology sequence ‘TGGGA’. There is a 1.468 Mb duplication at 13q33.1 between f1 and f2 in this RC13.

**chr13:101308886-left-clipped sequence matches chr13:10407800-10416399(-) at 13p11.2:**

TAAACTCATGCAATAGGTCCAAACCAGACCAAACCAAACCAAAATGGAGTCACTCTTGCTAAATGTAACATAATCAAACTAAGACTTTAAGGAAACACATAAATCCTAGAACAAACCAGGTTTGTTTTTCTCCTGTAAACAGGATGTTCCAGCATAAGAAGATACCTTCTACTCAAGTCCTTGTTCCACCTTTTCAAATCTCACTGGTCTATTTCCCAGTGGGTTTCTAAACCAAGTAAGTACATTTGCAATGGTAATAGTGACATCAGTGACTGAAGTTTTGGCCAATCTCTCAAAATTGAGAAAATAACCAAAGGGAAGGCATTGTTAAGTGAACTAAGTATGGCCTGAGAAGGACTCCATAATTCTATATATGATTCATTGTGGATGAACTGTAACCTACCTTAATAGGTATAGAAGAATGAAAAACTAACTTAAGAGTATGCACCTTGAACAACAGCTACATCTTGGCCAATCCCAATGGCCAAACTTCAACCACTCAGGCACTGCCAAATGTTCAAAATGTGTTCAAACAAGGCAAACGCTGAGTTGTTTCTGTACCTCACTTCCGATTTCGGTATGCCATTTCCCTTTTGTCTATAAATCTTCTTCCACCACATGAATGCGCTGGAGTCTCTGTGAACCTGATGTGATTCTGGGGACTGTCTGATTCGTGAATCGTTTATTGCTCTATTAAACTCCTTTAAAGTTTTTCTTTTAACAGAACTAACACAGAAGAATTTCCAGATCATGAACAGATGTTTTGTAATACCCAACGTTGTAACATGAATAGACTCTTCCTTAGATAGCTAACCTTGTTTTTAATATGAATAGACTCTCCCTTAGCTGAGAAAACCAGACAAACTCCATTTGGCTCCTTCATTTACAAGACATCAAGGGCTCCCTACCCACCCCCTTTCCTCAAGGACTTTAACTTGTGCAAGTTGACTTTCAACATATCAAAGAGTGCAATTAACTGATAAAGTGCTTAGACAAGCGATGTCTGCAGTTCCCAGCAATTTATTCAGAGATAGTATCATAAAGCCCCACATTTGTCTGGCAGATAATGCCCAGAGCCCCCTCACCTATCATTTTGTGGTGAATTTAAAGCCCCTGCACCTGGAACAGTTTGTTTTCCTGTAACCATCAGTCTTTTTAACTTTTTTGTCTGTTTTTTTCTTCTGTAAAGTTGCTGCAGCTAGAATCCCCCCTCCCCTCTCTAAACCAAAGTATAAAAGAAAATCTAGTCCCTTCTTCGGGGCCGAGAGAATTTCGTGCATTAGCCGTCTCTCAGTCACCAGCTAATAAAGGACCCCTGAATTCGTCTCAAAGCGTGGCGTTTATCTCTAACTCACTCGGGTTCGACAGTTTCAACTATGGTAGAAGACTTGAGTAAGTCCAATACAGTCCCCCTAAATTTGACTATTAATTAGGTTAATGGTGAGTTTAGAAGAAATAAGTTAAGACTACACAGAGTGGGCTAAAGTGCAAATAAACACTGGAAATATTTCCCAGAAAATATGACTTTGAACAGGCTGCTGCACACCCTGCATGTAGAGATAAACTAAGAAAAATGTCTGGAGAGTTATTTAAGGGCCTATGGTTAACTCAGTCCTCAAGATGTTCTGGGTTTCATCCATGAATCAAGGAGGACCTCCTAAAAGCTGTTTGGGACCACACTCTTTGAGCAATGAGCACACCTTACGATGGAAGCTGTACTTTAGCGGCAGATGACCATTACCACTGCACAACACGCCGTGCTTTAGCGGAAGATGACCGTTTCCACTGCACAACACTACAAGTGGTTACTGCCAGGCCGGTGTGAAATATGTTCCAGCACATAATCTATGTCACCAATGAAGGTGGTGGTTCGGACTTGGTGCACACAATCTTTCCTGTCCCACAAGAACACAGCATGCTCTCTTCTCGGGTTCCATTCCAATCACGTAACAAATATGACTGCCTTTTTTGTCTCGGCATCAGAAAGATCAGAGGAAAATTTGCACCCAACTTAGACTACTCTAAGCTCTTATAACCTGCCTATATCTACAAGTCAGCTTTATCTTATTTATGTATATTTCCTTCAACCTGAGTTTTACTTATTTATACTTTTCCTTTTTAATTCACACACACCCATAAACTCAGAAAATACAGTGTAAAACAAAGTGAAGAACAAGTAAGCAACTCACCAGAGATTTATTCGTTTCTTGTTGCTCTTGGAAACACCCAGAGGACACTGGAAACATAGCTGGGATAGAAGGCAAATGACGTGGATTAAGGAGAGAACTGGTGTGGTGTGGTCCCAGATTCTTCTGCCCAATGCTCTAGACACATTACCCGGGAAAGCCCTCCTCCCTCCTGAAAAAGAAAAACTTCTCCAGGGGAGAAGAGTTCTTCACGCCTCATTAGGGGCAGCAGAGGCTCAAGTTAAGATAAGATACATAGACAAGTACATTAATTGGTAGACATTAGATGCACAATTTATTTTTGAATAAATATATGTATTACCTACTAATTTAGTAACAATATGATCTAAAGATATAATCTAATAATTTAATACAAAGAAACATAAGTTCACTAAAATAAATGTTATAGAAATATACTGGGCTGTATTAACTATTTTCCTATTAATATGTAGATTCCACAAATAACTTCATATGAGTGTTCCCGTGACAGTACCTCTTGCTTTTCTATACCTGAACATCATGGAAAGTGCATCTTGCAAACCAGCAATTTTGGCCTACAATTACGCTTTTTAAAATGTACATAATGCGTATTTCCTACAGTACACCATTCTACTCATGTTTCCCAATAACACCTTTCCTTCTATCCAAGCTCTCATATTATGCTCTGACAATAAATTGGGCTTTTCCATCTGACTTGTCCAGTGAATGGACAATGGAAAATGTGATGCAAATATCCATTGGTTCTTCCCTTTTTGGGAGAAATTGTGAAGAGGTCTGGAGCTACCCTGTTGGAGACACAGGGCCTAGCCAAGAGTCACCACAAACCACCAGATTGTGAAGGAAACTATCTTAAACCAACCAGGCTCAGTCAAGGCACCAGGTGACTAAGGCCTTTTTGTGATCCAGGCAACACAAATATATCAACTACCCAGGTGAATCCACCACACCAAAATGCAGATCCACAGAACTTCAAACAAATAAAATGGTGTTTGTTTTTTATAAGCCAGTAAGGTTTAATCAGTTCCTTAAACAGCAAGTATTAACTGTTACACCTAAGTGAACAGAATTCACTTCTGTGTTTTTAACAAAATTATGTAGGGGGAGAAAATCTTAAATTACAAATCAAATACAATCAATAGAACTCGCAATCTAATGCTAAATTTGGTGATGGACTAGGTTTAATATATCTCAGACGCGAAAAAACGAGCTAAGATTGAAGGAATGGGACTGTGTTTGGAGAGTATTTTAATCCTCTCAAGTATGACAGGTCACTCCTGTACCCCAGACCACACTTTCAGGCCCCTTCAAATAAGGAATATTTCCTAAGTCCTTGCCTGTTTTTCTCAGCTGAATTCACCTCAACCTTCTGAAAGTTCGTCCAAACCTTCTACTATCACCTAGTCTTTGCAAATCTTGTGCATTCTAGGGAGTAGAATTAATATTTCCTGAGCAAGGAAAACTGGGATCTTCACCTGTGACCTTTTTTCCTCCTCTGAAGCACCAGTGAGAGGTTAGACCAGACGGCTGTTCTTTCAAGTGTGCTTCTTATTCATAGGGAACCCTCCCTTTCAAACTTTGTAACACACAGTTAAGACTGAAGTACCCTTAAGGCTGACGACCATCATCTATTACGCCATCTCCCTCGCGGAATCAGTGAGTTCTTCCCTGGAAACTAGGTCTCGTATAAACTTCTGTAAATGCGACCCAGGAGGACTAGGCAGGTCACACAGTGAAGGAGGGAACCAGAAACTTCACTTGCTAAAGAGACACCAGGAAACCAAACTAATACAAACGTCAAGTTTAAGACTAGAGGCGCATGCGTTTCACACTACTCCTCTGGGAATGGGGAACGTCTCCCGAGAACTGTGTGTTAGCACTGGGACAGATGGGCAAACTGAGCATCATGCGGGTTGGTAACCGGGTCCCTCAGCGGCAGGACAGGAGCGCGGCCTGCAGACTCCGGGCCCAGGGCCACCGGCCTCTCCTACCCGCTCCTGTGCCTCTAGAACCCGCGTCACTGCTGGGACCCCACGCCTGTCCTCCCAGCCCCTGCCAGGGTCCGCAGCCCGCACCTGCTCTTCAGGCCCTGCCCTCCCGTGATGCGCCCACGCGTCTGCTCCCACAACTAGGGAACACTGGTCCGGCCCCCTGGGATCCCCTGAGGCTCACGGGTTCCTCCTGGCCCTCGCACCGACCCACAGGGACATAGAACCAAGCCCCAAGCCGGCCCAGCTACAGTACCGCCTCTGGGTGCCACACTTCCGGAGGAAAATGGCGGAGTGGGCCGGGCGGCGCATGCGCAGAGAGAAAAGCTGGTTCCCAAAGTCCTTGATGGTAACGTCATTGGAAGGTGACACTACAATTCCCATGAGGCTTTGCGGTCCCCATTTAGGAACCCACGCCGGACATTCTGTTTTGCCCAGCAGTTGAGTCCAGTTACCCAGAGACCCGGACTTAATGTATCAGGACTGGTCCCTACCCAGGTGACAGAGATGTGGCATTCTGGTTCTTTATTAAATACTGGTTTCACAGCCTGGGACATTGTGAAAATAATGGAGAAATTTCAATAGAGGCCAATTGGTCTATGCTATTAATCAGTAACTTTTTTTTTTTTTTGAGACGGAGTCTCACTCTGTCGCCCAGGCTGGAATGCAATGGTGCGATCTTGGCTCACTGCAACCTCCGCTTCCCGGGTTCAAGTGATTCTCCTGCCTCAGCCTCCTAAGTACCTGGGATTACAGGCGCGCGCCACCACACTCGCCTAATTTTTGTATTTTTAGTAGACACGGGTTTTCACCATGTTGGTCAGGCTGGTCTCCAACTCCTGACCTCGTGATCCGCCCTCCTCGGCCTCCCAAGGTGCTGGGATTACAAGCGTGAGCCACTGCACCCAGCCCTCAAGTCTATTTTTTATAGATACATTCGAAAGCATGAAAAAAATCATGTCTCTATTTTACTTTAAAATTTTAAAAACACAACTAATGAATATGGTAATTCTCTTCCAATCTGTTATCTTTTCTCTCACTAAACTAATTTGTGAGCCTTCAATTTACACAGTTAGAAAATATGCTCTAATGCATATACTAGGATAAAATAACAGGGTCATAAGACAGGTGCACTCCATAATCTTTGTGACAACTTTCACTTCCAGTGTCTGATAAATATTTGCCCGTAGACTCCCACGTTTCATCCATCCATCAATCAATCAAATCTACCTATCTTTATTTATTTATTGTGAGAAAGACCTGAAATTTGCCTTTCCTTCCCTGATTTCTGCCACAAACTAGGCAAGGAGTTCTGCATAGGGGTTTCTCAGAGCTCTGGCTACCACCGATGTTCCCAACAGGGAAACGCAGGCTTGAATGCTCAGGGTTGATGTGGGAGTGCGTGTGAAACGGGGGTGGGGTGAAAGGGCAGTGACGTTTGTAGGTGGGCAGATGGGGGTGTTGATAGGCTTTCAGGTAAGAGGCACGCAGGAAACTGGGAGAGGCAGCAAAAGCACCTCACACCTCAGATCACCAGAAGACGCTCCCTCCAGTGCCATGACAGTTTGCCAATGCCATGTCATCACGAGAAGTCCCCACCCCTTGCCATGGAAACAGATGGAAGTTACTGCCCATTTCTAGCTATTTCTGAATAACCCTCCCCTTAATTAGCATGCCATTAAAAGTGAATTATAAAAGTGACTACAAGCCACCCCTAGGCTGCTGCTCTGGGAGCACAACCCACGGAGGGCTCCCTGCCCTGCCGGAGTGGTTGCAGGGTTGTAACACCGCCAATGCCTCCGTAGAGCTGCTTTCTTCCACCATAGGCTTGCTTTTGGATTTCTTCCTGAGCGACGCCAAGAACCTGCCCTTCCTCAGTGTGACTCTTGCCTAAAACCTATCCCTGGTATTCTCTTTTCCTAAGCATGCCCTGACTTGTTCTTTCATCTCCTCTGATCTTGCAATTGGTCCTCAGTGACTCTATTCTGCAGATCCAGAAAACTCAACCTTAATCTTCCCAGAGCCCTGTTGTCTCTAATATTGGAATCTATAGCCTTGTTTTCTCAGACGCCTAGATTACAGGCCTCTCTCTTGAACACCTATTGGTATGGTATCTGGGGATCCTTTAAATACTTGATGATTGGCAGGGGTTAAATAGCGGAAATCAGTGCCTGACAATTCGCCTTCCAGGATATGGACTGTCATTCCCTCTCTTGGTGGGCCTCAGTCTCTTATCAATAAAGTAGAGATTGTAATACTCATTTGAATTGCAGATACCTCAACCCGAACCCACCTAATATAATGTAAAAGCCAAGAATGCAACCCCTTTCCTCACCCCGTGAAGGTAAAGCCCTCAGAGCCAAGGAGAGAAGGCTCAGGGATGGTATCTGGGTGTTTCCAATGCTAACCATGTATTGTAGTTTTTAGTGTTCAAGTTTAAGCTTCCCCAGCTTTAATTCTATTGTAACAAGATTTATTTTTGTAATTCCATTTTTGGATTCTTGATTTCTTGGTAAAGAAATACAGTTATTTTTGTATACCAATCTTATACAGTGTTACATTCTTAAATTTGTTCATGAGTCCTAATACTTTTTAGTAAATTTCTTACGATTTTCTAAATGCAAGATCATGTCATCTGTACATAAAGATAACTGTACTTCTTCTTTTCCAATCTAGATGCTGTTTATTTATTTACATTGCCAAATTGTCCCAGCTACCACTGTTATCAAGTAAAAGGGTCTCACTGCCCAAAGCACAAGAAGCCGGTACCATGACACTGAGTTTTCAAGAAAAGAAAAAGTTTAAAGTCAAACCAAAACCTAGGGTAGAGGCTGGGCGCAGTGGCTCATGCCTGTAATCCCAGCACTTTGGGAGGCCAAGGCGGGCGGATGATAGGGTCAGGAGATTGAGTCCATCCTGGCTAACACGGTGAAACCCCGTCTCTACTAAAAATGCAAAAAAAAAAAATTAGCCAGGCGTGAAGGCGGGCGCCTGTAGTCCCAGCTACTTGGGAGGCTGAGGCAGGAGAATGGCAGGAACCCAGGAGGCAGAGCTTGCAGTGAGCCAAGATCACGTCACTGCACTCCAGCCTGGGAAACAGAGCGAGACTCCATTTCAAAAAAAAAAAAAAAACCTATGGGATACAGTAAAAACAGTACTACAGTACTAAGAGGTAAGTTTATAGAAAAAAGCACCTACATCAAAAAAAGTAGAAAAGCTTCAAATAAACAACCTAATAATGCATCTTAAATAATTAGAAAAGCAAGAACAAACCAAAACCTAAATTAGTAGAAGGAAACATAGCAAAGGTTGGAGCAGAAATAAATGAAATTGAAATTTAGCAATATAAAATATCAATGAAATGAAAAGTTAATATTTTTAAAAGACCAACAAAATCAACAAACACTTAACGAGACTAAGAAAAAAGAGAGAAGATTCAAATACATAAAACCAGAGATTGAAAAGGAGACACTATAACTGATACTGTGGAAATTCAAAGAATCATTAGAAACTATTATGACCGACTATATTCCAATAAATTGAAAAACCTGGAAGAAATGGCTGGGCACCGTGGCTCATGCCTGTAATCCCAACACTTTGGGAAGCCAAGGCAGGTGATCACCTGAGGTCAGGAGTTCAAGACCAGCCTGGCCAACATGGTGAAACCCCATCTCTACTACAAATACAAAAATTAGCCAGGTATGGTGGCATGCACCTGTACTTCCAGCTACTCCAGAGGCTGAGGCAGGAAAATCACTTGAACCTGGGAGGCAGAGGCTGCAGTGAGCTGAGATTGTACCACGAAGCAGCCTGGGTGACAGAGCAAGATTCCATCTCAAAAAAAAAACCTAGAAGAAATGGATAAATGAAATTGAAGCCATAATAAAACATCTCCTAGCAAAGAGAAGCCTGGATTCAATGGCTTCACTGGCTTCATGGATTAATTTTACCAAACATTGAAGGCAGAATTACTTTCAATCCTACCCAAACTATTCCAAAAAACAGAGAAGGCTGTAGTATTTCCAAACTCATCCTATGAAAAAGACCATTCATCACGTCTAAGTGGGATTTATCCCGATGATGCCAACATGGTTCAACATATGCAAATCACTCAATGTGACACATCATATCAACAGAATGAAGGACAAAAACCATATGATAATTTCAATTGATAATGAAAAGCATTTAATAAAATTCAACATCCCTGTGATAAAAAGAAACCCTCAAACAAAAACTAGATGTAGAAGGAACATACCACAACACAATAAAAACCAAATGCATCAGACCCACAGCCAGAATCATCCTGAACAGGGAAAAGCTGAAAGCCTTTCTTCTAAGATCTGGAACAGGACAAGAATGTCCACTTCCAACACTGTTACTCAACACAGTACTGGAAGTCCTAGCTAGAGCAATTCAGACAAGAGAAAAA

**chr13:102776802-right-clipped sequence matches chr13:102766587-102769861(-) at 13q33.1:**

TCTGGATGGCCAGGGGCATTGTCCATATCAAAAGAACTTTAAAAGGCAGTCCCTTACAGGTAAAGTACTTCCTAGCTTCAGGCATAAAGCATCAATGGAAGCAATCCAGGAAAGGTGTTCTCATTGTCCAGGCCTTTTTGTTGTACAACTGAAAGACTGGTAGCTGGTGTTTGTCTTTACCCTTCAAGACACAGGGGTTATAAGCTCTATACATAAGGGCAGTTCTGAAGATAAATCCACTGGAATTTGCACCAAAAAGTAGAGTTAGCCTATCCCTTTCTGCCTTAAATCCTGGTGCTCACTTCTCTTCCTTACTAATAAACTTCCTTTTTGGCTTTTTCTTTTTTCCCAGAATAGTGCACTTTTGTTTGCATTAAAAAACCTGTTCACGCAAATATCCTTTCTCCTCAATGGCATCTGGGAACTCACCCACTGCCTCTTGGTCAGCAGAAGCTCCTTATCCTGTAATCTTCCCATTTTGTTTTAAGCCAAACCTCTTTCTAAAATTATCAAGCCATCTTTGAGAGCATTAACATTCTCTGAAACTCTCCAACTTTTTTAGATCCTTCACCGTCCTTTCTTCAAGTTGTCATATAATGATTTCATTTTTTTTCTGAGAACGTGTTAGAGTCTATAGGTATGCCTTTCTTGCAGCAATCTTATGCGGAAAAAAAAGCTGCATTTTCAGTATGAGATAAAATGATATTTCACCAAAAGTGCAAGACTTCCCACCTGCTGGCATAAGTACAATGACAGCTTCACAGTTTTTCTTTTTTTACAATAGTCCTTATGCTGCAATCATTTATTGGTAAATAGCAGATAAACGGACCATTAATCATCTTTTGGGGCTGGGTCCCAGACTTATGTTCACATTGTTCAAGAAGGTGATGATAGGGTAGATACATCCTACAATGCCAACTTGACTTTCAAACTCAGTACCAACCCACTTCCAGTGTATTTTTACGAATTATCTCCCATTTGTAGTAAAATAAGACAATTAAAAATAATTAAGTTTGAATTAATATAATTAACTTAGATACCTATCAGAGTAATAAGCAATATTTATAGTATTAGCTTATAATATTTTAATAAGCTTAGCTATGGCATTTACAAATCAAAAGAAATTGTTTCCTTATATTTTTTCTATCTGCCCCAATTTGTTGTTATTAGAGCCAAAATTTAGTGGGTTTTTTTTCAAATGATGTAAAGGTTTGATGTGTGAAGAAGGCTGTTTTGAAAATATTGGAGACGATATTTCCTAGCACTCCATTCCAATATTAATAAATAGGAGAACATTCTCAGTGATTTTGATAAGTGCACAAACATGAGATGTGAGATTTACAACAGAAGCAGTCTGTCTTATTCAAAGACAGACAAACATTTTCTCAGTTCTATAATTTCTAGAAGAAATGACTTACTTAGCATTTGTATAACTTCTACTCACATAAAGATTTATCTGTGTTCACCCCTATGCCTGGAGTTGCACAGATAAACATGGATTAGGAAATAGATTACACGCTATCTACTGATGCAATTTTAAAAGTTGGTGATGGAGTATCTACCCCTTTGGAAATAAAGTATGCAGATCTTTAAACACCTACCTCAACAGAAAATACCCTATTATATGAATAAAATGATATTATTTTTTATAAATCAGCCATGATTCTAATATTATTCCTGTAAGGGCTCTTATATTCAGCTTGAATGTGAAACTGTTTCTATAATTGCACATCTATAAGTTTTATTTAATTGGATGTTGCTAATTGCAGACTTTGTATGTGGTCAATAATCTTCCTAATGTTACTAGAAAGAATTTGTGCATTATTGTAAAGATTTGACTTTAAATTTAATTCCTCTAGCTAGAATATATGATGAAACAAAAAGAGTCACAAGAATATTTAGGGGTAATCTATTTTATTATTTTTATCTATCAGAAGATAAAGTCCTGAATAGGTTATCTTGACCAAAACATATATACTATTTGAACTTCATTTTAAAACCTGATTATTTCTAACGTTGTAGGATTTAGGTCAGGTGGTCCAAGTTATATGTAAAAGTTATAAGGAAAGACGTAAACCTTCTTGGAAGGCCAGGAGGTTTAGCAAAGCTTCGGGACTTTAGCCGAAGGCAGCTAAATACTCGTAAGGGCAAAGGGTAGATAACAAGGGAATGTAAAGGAACTTATCCAGATAAATTGATTTACTTATGTCTCTGGAAACCAACCTTTGATCATTCAGGAGCAGGACTGCTCTCTGCTTGGGGGGCCGACAATGTTTATTACCCATAAACTGTGTTTGCTCCAAGCCTTTGTCATTAAATCTGTGCTAAGTAAATGCTAGCATTGCTGGCTTAGGTGGGGGGCTGCACTCTCACCAGCGGCGCTAAGTGATGTGGTCCCTTTGCCGTGCTGTTAGGCAAAATACCTGCGTCAGTGTACTTCTTTCATCTATCGCTCGGCCAGAGTCAGCAGGATGGACCCGGCATAATATCACTTCAGGTCATTCAGAAATTTTATATACCAAAATTTCGGAATTGTGAAGAATTAGAAATTATTGCTGTAGTGCATTGACTATTGTCCCCCCAAAATTCATATCCACCTGGAACATCAGAATGTGCCCTTATTTGGAAACAGAACTTTGCTAATATAATTAATTAAGGATCTAGAGATGAAATCCTTCTGGATTTAGCATAAGCTCTAAACCAATGAGTTGTGTTCTTCTAAGAAAAAGAGAGACAGAGAAAAGATGTGAAGATGAAGACAGAAATTGGAGGACGCATCTACAAGCCAAGAGAAGCCAGGGATTTACTATAACCACCATTAGCTAAGAGAGAGGTATGGGCTGATTTCTCCCTCAGAACTTTCAGAAACAACCAACCCTACTGACAATTAGAGTTCAGATTTCTTGCCTCCTGAACAGTTAGAGAATAGCAATTTATGTTGTTTAAACCAATGAATTTGCAATAATTTGTCATGGCAGTCATAAGAAACTAACACAATTCCATATGCTTTGAAATTTATGACTACTTAAAATTGCTCATTTAACAATTTTATTTCTTTCATTAGCACAATCCAAGGTTGTTTTATTGCAATTTTTAAGAATCTAAGTAACATTTTGAATGTTAATTGGCCTTATTGACAATATGATCTTCATTTGTACATTTAGCCCAATGTTAGTGCTGTTTCACTGCTATTTGAAATTGAGATAGGAGTTCAGCAGGACTGGTTTTACAAAGTACAGGTCACAAAGAGCCCGCTAATAATAAAATAGGATGCT

**Supplementary File 4B. GS4-RC13**

**Two-break and two-fusion for GS4-RC13 (microhomology)**

**13p13(+)** 4748590-TTTATGTACACTA**TAG**AATATATATCTGGTGTACTTT -4748626

**13q21.33 (+)** 69863060-GTTTGTTATGGATC**TAG**TTGTGAGACAGCGTTTTCTCAGGA-69863100

**Fusion sequence 1 (f1):** TTTATGTACACTA**TAG**AGTTGTGAGACAGCGTTTTCTCAGGA

**13q34 (+)** 113145040**-**TCGGACACGCACCTGCTGACAGACACTGGGGCTGTTCCCAG-113145080

**13q34 (-)** 113150180-cccatcgggtggtctctgtccTCCCCATCGGGTGGTCCCCT<-113150140

**Fusion sequence 2 (f2):** TCGGACACGCACCTGCTGTCCCCATCGGGTGGTCCCCT<-

**ISCN:**

seq[T2T] r(13)(p13q34) g.(pter)_4748606del::69863075_113145057dup::113150159_(qter)del

**a.** **CNV analysis showing a 4.75 Mb distal deletion of 13pter-p13, a 43.28 Mb duplication of 13q21.33q34, and a 417 kb distal deletion at 13q34. Below, IGV view of the junctions.**


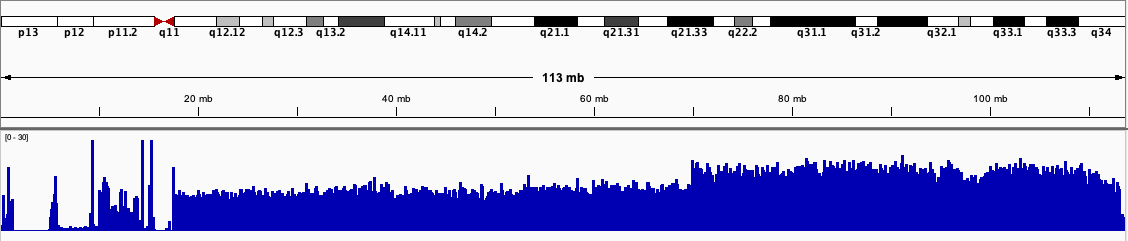


**f2**

**f1**

Chr13:69863076-left-clipped sequences for fusion 1 (**f1**)


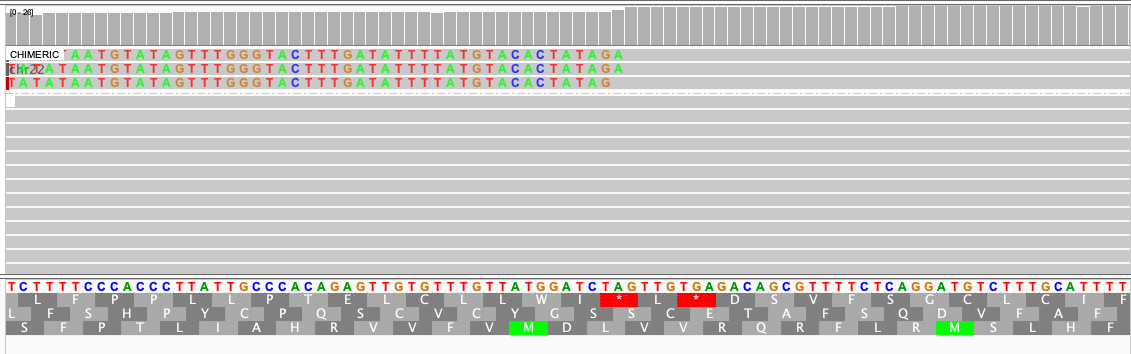


Chr13:113145057-right-clipped sequences for fusion 2 (**f2**)


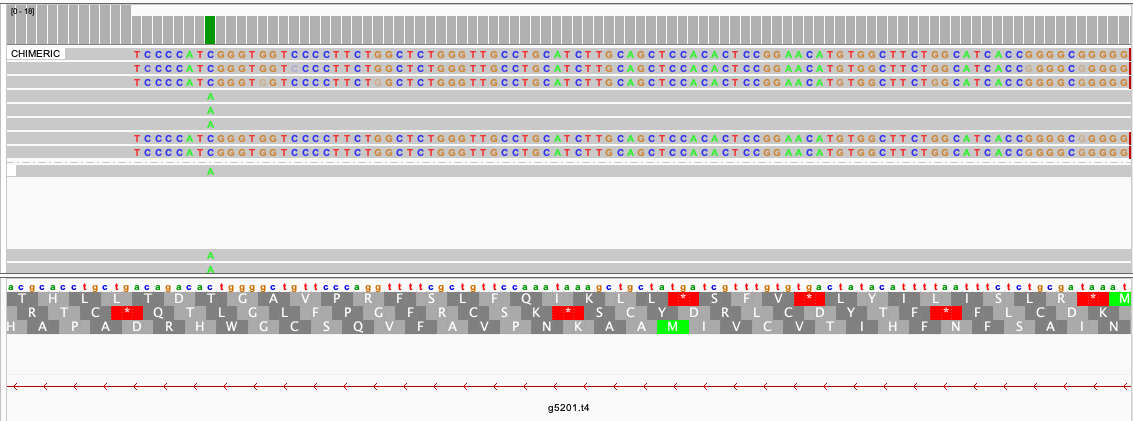


**b. BLAT search results to T2T**

The 11.698 kb chr13:69863076-left-clipped sequence matches to numerous segments at 13p and 22p (identity 82.6%-100%). The matched sequences at 13p matches classical human satellite 1A (hsat1A_13_1). The 8.587 kb chr13:113145057-right-clipped sequence match to (identity 99.8%) chr13:113141573-113150159 at 13q34.

**Chr13:69863076-left-clipped sequences match hsat1A at 13p:**

TTCATGTACAGTATATCATATATATTTGGGGTACTTTGATAGTTTATGTACAGTATATAATCTATTCTAGAAGTACTTTCATATTTTATGTACCTAATATAATATATATTTGATGTAATTTCATATTTTATGTACAGTATATAATGAATATTTTGGGTACTTTGATATTTTATGTACACTATAAAATATATATCTGGTGTACTTTGATATTTTATGTACAGAATAAAATATATATTTGATGTACTTTCATATTTTATGTACAGTATATAATACATGCTTTGGGTACTTTGATATTTTTTGTACAGTATAGAATATATACCTTGGGTACTTTGATATTTTATCCACAGTGTATAATATATAGTTTGTGAACTATGATATTTCTTGTACAGTATATAATATATATTTGGGGTACTTTGATATTTTATGTACAGTTAATAATCTATATTTGATGTACTTTCATATTTTAGGTAACTAATATAATATATATTTGATGTACTTTCATATTTTATGTACAGTATATAATGTATACTTTGGGTACTTTGATATTTTATGTACACTATAAAATATATATCTGGTGTACTTTGATGTTTTATGTACAGAATAATATATATACTTGATGTACTCTCATATTTTATGTACAGTGTATAATACATGCTATGGATACTTTGATATTTTTTATACAGTATAGAATATATACCTTGGGTACTTTGATATTTTATCTACAATATATAATATATAGTTTGCGAACTTTGATATTTCATGTACAGTATATAATATATATTTGGGCTACTTTGATATTTTATGTACAGTATATAATCTATACTAGATGTACTTTCATATTTTATGTACCTAATATAATATATATTTGATGTAATTTCATATTTTATGTACAGTATATAATGTATAGTTTGCGTACTTTGATATTTTATGTACACTATAAAATATATATCTGGTGTACTTTGATAATTTATGTAGAGAATAAAATACATATTTGATGTACTTTCATATTTTATGTACAGTATATAATACATGCTTTGAGTACTTTGATATTTTTTGTACAGTATAAAATATATACGTTGGGTACTTTGATATTTTATGTACAGTATATAATATATAGATAGTGAAGTTTGATATTTCATGTACAGTATATAATATATATTTGGGGTACTTTGATATTTTATGTACAGTATATAATCTATATTTGATGTGCTTTCATATTTTATGTACCTAATATAATATATATTTGATGTAATATCATATTTTATGTATAGCATATAATGTATAGTTTGGGTACTTTGATATTTTATGTACACTATAGAATATATATCTGGTGTACTTTGATATTTTATGTACAGAATAAAATATATATTTGATGTACTTTCATATTTTATGTACAGTATATTATACATGCTTTTGGTACTTTGTTATTTTTTGTACAGTATAGAATATATACCTTGGGTACTTTGATATTTTATGTACAGTATATAATATATAGTTTGTGAACTTTGATATTTCATGTAGACTATATAATATATATTAGGGGTACCTTGATATTTTATGTACAGTATATAATCTATATTTCATGTACTTTCGTATTTTATGTACCTAATATAATATATATTTGATGTACTTTCATATTTTAGGTACAGTATATAATACATGCTTTTTGTACTTTGTTATTTCTTGTACAGTATAGAATATATACGTTGGGTACTTTGATATTATATGTACAGTATATAATATATAGTTTGTGAACTTTGATATTTCATGTACAGTATAAAATATATATTTGGTTTACTTTGGTATTTAAAGTACAGTATATAATCTATATTTGATGTACTTTCATATTTTATGTACCTAATATAAAATATATTTGATGTAATTTCATATTTTATTTACAGTATATAATGTATAGTTTGGGTACTTTGATATTTTATGTACACTATAGAATATATATCTGGTGTACTTTGATATTTTATGTACAGAATAAAATATATATTTGATGTACTTTCATATTTTATGTAGCTAATATAAAATATATTTGATGTAATTTCATATTTTATTTACAGTATATAATGTATAGTTTGGGTACTTTGATATTTTATGTACACTATAGAATATATATCTGGTGTACTTTGATATTTTATGTACAGAATAAAATATATATTTGATGTACTTTCATATTTTACGTACAGTATATTATACATGCTTTTGGTACTTTGTTATTTTTTGTACAGTATAGAATATATACCTTGGGTACTTTGATATTTTATGTACAGTATATAATATATAGTTTCTGAACTTTGATATTTCATGTAGAGTATATAATATATATTTGGGTACCTTGATATTTTATGTACAGTATATAATCTATATTTCATGTACTTTCATATTTTATGTACCTAATATAATATATATTTGATGTAATTTCATATTGTATGTACAGTATATAATGTATAGTTTGGGTACTTTGATATTTTATGTACACTATAGAATATATATCTGGTGTACTTTGATATTTTATGTACAGAATAAAATATATATTTGATGTACTTTCATATTTTAGGTACAGTATATAATACATGCTTTTGGTACTTTGTTATTTTTTGTACAGTATAGAATATATACCTTGGGTACTTTGATATTATATGTACAGTATATAATATATAGTTTGTGAACTTTGATATTTCATATAGAGTATAAAATATATATTTGGGGTACTTTGGTATTTAATGTACAGTATATAATCTATATTTGATGTACTTTCATATTTTATGTACCTAATATAAAATATATTTGATGTAATTTCATATTTTATGTACAGTATATAATGAATAGTTTGGGTACTTTGATATTTTATGTACATTATAGAACATATATCTGGTGTACTTTGATATTTTATGTACAGAATAAAATATATATTTGATGTACTTTCATATTTTAGGTACAGTATATAATACATGCTTTTGGTACTTTGTTATTTTTTGTACAGTATTGAATATATAGCTTGGGTACTTTGATATTATATGTACAGTATATAATATATAGTTTGTGAACTTTGATGTTTCATGTACAGTATAAAATATATATTTGGGGTACTTTGGTATTTAATGTACAGTATATAATCTATATTTGATATACTTTCATATTTTATGTACCTAATATAAAATATATTTGATGTAATTTCATATTTTATGTACAGTATATAATGTAAAGTTTGGTACTTTGATATTTTATGTACACTATAGAATATATATCTGGTGTACTTTGATATTTTATGTACAGAATAAAATATATATTTGATGTACTTTCATATTTTATGTACCTAATATAAAATATATTTGACGTAATTTCATATTTTATTTACAGTATATAATGTATAGTTTGGGTACTTTGATATTTTATGTACACTATAGAATATATATCTGGTGTACTTTGATATTTTATGTACAGAATAAAATATATATTTGATGTACTTTCATATTTTATGTACAGTATATAATACGTGCTTTGGGTACTTTGATATTTTTGGTACAGTATAGAATATATACCTTGGGTACTTTGATATTTCATGTACAGTATATAATATATAGTTTGTGAACTTTGATATTTCATGTACAGTATAAAATATATATTTGGGGTACTTTGGTATTTAATGTACAGCATATAATCTATATTTGATGTACTTTCATATTTTATGTACCTAATATAATATATATTTGATGTAATTTCATATTTTATGTACAGTATATAATAAATAGTTTGGGTACGTTGATATTTTATGTACACTATAGAATATATATCTGGTGTACTTTGATATTTTATGTACAGAATAAAATATATATTTGATGTACTTTCATATTTTATGTACAGTATATAATACATGCTTTGGGTACTTTGATATTTTTTGTACAGTATAGAATATATACCTTGGGTACTTTGATATTTTATGTACAGTATATAATATATAGTTTGTGAACTTTGATATTTCATGTACAGTATAAATTATATATTTGGGGTACATTGGTATTTTATGTACAGTATTTAATCTATATTTGATGTACTTTCATATTTTATGTACCTAATATAAAAAGTATTTGATGTAATTTCATATTTTATGTACAGTATATAATGTATAGTTTGGGTACTTTGATATTTTATGTACACTATAGAATATATATCTGGTGTACTTTGATATTTTATGTACAGAATAAAATATATATTTGATGTATTTTCATATTTTATGTACAGTATATTATACATGCTTTTGGTACTTTGATATTTTTTGTACAGTATAGAATATATACCTTGGGTACTTTGATATTTTATGTACAGTATATAATATATAGTTTGTGAACTTTGATATTTCATGTACAGTATAAAATGTATATTTGGGGGTACTTTGGTTTTTAATGTACAGTATATAATCTATATTTGATGTACTTTCATATTTTATGTACCTAATATAATATATATTTGATGTAATTTCATATTTTATGTACAGTATATAATGTATAGTTTGGGTACTTTGATATTTTATGTACACTATAGAATATATATCTGGTGTACTTTGATATTTTATGTACAGAATAAAATATATATTTGATGTACTTTCATATTTTATGTACAGTATATAATACATGCTTTGGGTACTTTGATATTTTTTGTACAGTATAGAATATATACCTTGGGTACTTTGATATTTTATGTACAGTATATATAATATATAGTTTGTGAACTTTGATATTTCATGTAGTAGAGTATAAAATATATATTTGGGGTACATTGATATTTTATGTACAGTATATAATCTATATTTGATGTACTTTCATATTTTATGTAACTAATGTATATTTGATGTAATTTCATATTTTATGTACAGTATATAATTTATAGTTTGTGTACTTTGTTATTTTATGTACACTGTAGAACATATATCTGGTGTACTTTGATATTTTATGTACAGAATAAAATATATATTTGATGTACTTTCATATTTTATGTACAGTATATAATACATGCTTTGGGTACTTTGATATTTTTTGTACAGTATAGAATATATACCTTGGGTACTTTGATATTTTATGTACAGTATATAATATATAGTTTGTGAACTTTGATATTTCATATACAGTATATAATATATATTTGGGGTACTTTGATATTTTATGTACAGTATATAATCTATATTTGATGTCCTTTCATATTTTATGTACCTAGTATATATTTGATGTAATTTCATATTTTATGTACAGTATATAATGTATAGTTTGGGTACTTTGATATTTTATGTACACTATAGAATATATATCTGGTGTACTTTGATATTTTATGTACAGAATAAAATATATATTTGATGTACTTTCATATTTTAGGTACAGTATATAATACATGCTTTTAGTACTTTGTTATTTTTTGTACATTATAGAATATATACCTCGGGTACTTTGATATTATATGTACAGTATATAATATATAGTTTGTGAACTTTGATATTTCATGTACAGTATAAAATATATATTTGGGGTACTTTGGTATTTAATGTACAGTATATACTCTATATTTGATGTACTTTCATATTTTATGTACCTAATATAAAATATATTTGATGTAATTTCATATTTTATTTACAGTATATAATGTATAGTTTGGGTACTTTTATATTTTATGTACACTATAGAATATATATCTGGTGTACTTTGATATTTTATGTACAGAATAAAATATATATTTGATATACTTTCATATTTTATGTACAGTATATAATACATGCTTTGGGTACTTTGATATTTTTTGTACAGTATAGAATATATACCTTGGGTACTTTGATATTTTATGTACAGTATATAATATATGGTTTGTGAACTTTGATATTTCATGTAGAGTATAAAATATATATTTGGGGTACATTGATATCTTTTGTACAGTATATAATCTATATTTGATGTTCTTTCATATTTTATGTACCTAATATGTATTTGATGTAATTTCATATTTTATGTACAGTATATAATTTATAGTTTTTGTAATTTGTTATTTTATGTACACTATAGAATATATATCTGGTGTACTTTGATATCTTATGTACAGAATAAAATATATATTTGATGTACTTTCATATTTTATATACAGTATATAATGCATGCTTTGGGTAATTTGATATTTTTTGTACAGTATAGAATATATACCTTGGGTACTTTGATATTTTATGTACAGTATATAATATATAGTTTGTGAACTTTGATATTTCATGTAGAGTATAAAATATATATTTGGGGTACATTGATATTTAATGTACAGTATATAATCTATATTTGATGTACTTTCATATTTTATGTACCTAATATAAATTATATTTGACGTAATTTCATATTTTATTTACAGTATATAATGTATAGTTTGGGTACTTTGATATTTTATGTACACTATAGAATATATATCTGGTGTACTTTGATATTTTATGTACAGAATAAAATATATATTTGATGTACTTTCATATTTTAGGTACCTAATATATATTTGATGTAATGTCATATTTTATGTACAGTATACAATTTATGGTTTGTGTACTTTGTTATTTTATGTACACTATAGAATATATATCTGGTGTACTTTAATATTTTATGTACAGAATTAAATATATATTTGATGTACTTTCATATTTTATGTACAGTATATAATACATGCTTTGGGTACTTTGATATTTTATGTACAGTATATAATATATAGTTTGTGAACTTTGATATTTCATGTAGAGTATATAATATATATTTGGGGTACTTTGATATTTTATGTACAGTATATAATCTATATTTGATGTGCTTTCATATTTTATGTACCTAATATAATATATATTTGATGTAATATCATATTTTATGTATAGCATATAATGTATAGTTTGGGTACTTTGATATTTTATGTACACTATAGAATATATATCTGGTGTACTTTGATATTTTATGTACAGAATAAAATATATATTTGATGTACTTTCATATTTTATGTACAGTATATAATACGTGATTTGGGTACTTTGAGATTTTAGGTACAGTATAGAATATATACCTTGGGTACTTTGATATTTTATGTACAGTATATAATATATAGTTTGTGAACTTTGATATTTCATGTACAGTATAAAATATATATTTGGGGTACTTTGGTATTTAATGTACAGCATATAATCTATATTTGATGTACTTTCATATTTTATGTACCTAATATAATATATAGTTGATGTAATTTCATATTTTATGTACAGTATATAATAAATAGTTTGGGTACGTTGATATTTTATGTACACTATAGAATATATATCTGGTGTACTTTGATATTTTATGTACAGAATAAAATATATATTTGATGTACTTTCATATTTTATGTACAGTATATAATACATGCTTTGGGTACTTTGATATTTTTTGTACAGTATAGAATATATACCTTGGGTACTTTGATATTTTATGTACAGTATATAAGATATGGTTTCTGAACTTTGATATTTCATGTAGAGTATAAAATATATATTTGGGGTACATTGATATTTTATGTACAGTATATAATCTATATTTGATGTACTTTCATATTTTATGTACCTAATATAAAATATATTTGATGTAATTTCATATTTTATGTACAGTATATAATGTATAGTTTGGGTACTTTGATATTTTATGTACACTATAGAATATATATCTGGTGTACTTTGATATTTTATGTACAGAATAAAATATATATTTGATGTAATTTCATATTTTATATACAGTATATAATACACGCTTTGGTTACTTTGACATTTTTTGTACATTATAGAATATATACCTTGGGTACTTTGATATTTTATGTCCAGTATATAATATATAGTTTGTGAACTTTGAAATTTCATGTAGAGTATATAATATATATTTGGGGTACTTTGATATTTTATGTACAGTATATAATCTATATTTGATGTACTTTCATATTTTATGTACCTAATATAATATATATTTGATGTAATTTCATATTTTATGTACAGTATATAATGTATAGTTTGGGTACTTTGATATTTTATGTACACTATAGAATATATATCTGGTATACTTCGATATTTCATGTACAGAATAAAATATATATTTGATGTACTTTCATATTTTATGTACAGTATATAATACATGTTTTCGGTACTTTGATATTTCATGTACAGAATAAAATATATATTTGATGTACTTTCATATTTTATGTACAGTATATAATACATGCTTTGGGTACTTTGGTATTTTTTGTACAGTATAGAATATATACCTTGGGTACTTTGATATTTTATGTACAGTATATAATATATGGTTTGTGAACTTTGATATTTCATGTAGAGTATAAAATATATATTTGGGGTACATTGATATTTTATGTACAGTATATAATCTATATTTGATGTACTTTCATATTTTATGTACCTAATATGTATTTGATGTAATTTCATATTTTATGTACAGTATATAATTTATAGTTTTTGTACTTTGTTATTTTATGTACACTATAGAATATATATCTGGTGTACTTTGATATATTATGTACAGAATAAAAGGTATATTTGATGTACTTTCATATTTTATGTACTGTATATAATACATGCTTTGGGTACTTTGATATTTTTTGTACAGTATAGAATATATACCTTGGGTACTTTGATATTTTATGTACAGTATATAATATATAGTTTGTGAACTTTGATATTTCATGTACAGTATAAATTATATATTTGGGGTACTTTGGTATTTTATGTACAGTATTTAATCTATATTTGATGTACTTTCATATTTTATGTACCTAATATAAAATGTATTTGATGTAATTTCATATTTTATGTACAGTATATAATGTATAGTTTGGGTACTTTGATATTTTATGTACACTATAGAATATATATCTGGTGTACTTTGATATTTTATGTACAGAATAAAATATATATTTGATGTACTTTCATATTTTATGTACAGTATATAATACATGCTTTTGGTACTTTGATATTTTTTGTACAGTATAGAATATATACCTTGGGTACTTTGATATTTTATGTACAGTATATAATATATAGTTTGTGAACTTTGATATTTCATGTACAGTATAAAATATATATTTGGGGTACTTTGGTTATTAATGTACGGTATATAATCTATATTTGATGTTCTTTCATATTTTATGTACAGTATATAATGTATAGTTTGGGTACTTTGATATTTTATGTACAGTATATAATGTATAGTTTGGGTACTTTGATATTTTATGTACACTATAGAATATATATCTGGTGTACTTTGATATTTTATGTACAGAATAAAATATATATTTGATGTACTTTCATATTTTAGGTACAGTATATAATACATGCTTTTGGTACTTTGTTATTTTGTGTACAGTATAGAGTATATACCTCGGGTACTTTGATATTATATGTACAGTATATAATATATAGTTTGTGAACTTTGATATTTCATGTAGAGTATAAAATATATATTTGGGGTACATTGGTATTTTATGTACAGTATATAATCTATATTTGATGTACTTTCATATTTTATGTACCTAATATAAAATATATTTGATGTAATTTCATATTTTATGTACAGTATATAATGTATAGTTTGGGTACTTTGATATTTTATGTACACTATAGAATATATATCTGGTGTACTTTGATATTTTATGTACAGAATAAAATATATATTTGATGTACTTTCATATTTTATGTACAGTATATAATACATGATTTGGGAACTTTGATATTTTTTCTACAGTATAGAATATATACCTTGGGTACTTTGATATTTTATGTACAGTATATAAGATATGGTTTCTGAACTTTGATATTTCATGTAGAGTATAAAATATATATTTGGGGTACATTGATATTTTATGTAGAGTATATAATCTATATTTGATGTACTTTCATATTTTATGTACCTAATATATATTTGATGTAATTTCATATTTTATGTACAGTATATAATTTATAGTTTTTTACTTTGCTATTTTATGTAGACTATAGAATATATATCTGGTGTACTTTGATATTTTATATACAGAATAAAATATATATTTGATGTATTTTCATATTTTATATACAGTACATAATACATGCTTTGGGTACTTTGATATTTTTTGTACAGTATAGAATATATACCTTGGGTACTTTGATATTTTATGTACAGTATATAATATATAGTTTGTGAACTTTGATATTTCATTTAGAGTATATAATATATATTTGGGGTAGTTTGATATTTTATGTACAGTATATAATCTATATTTGATGTACTTTCATATTTTATGTACCTAATATAATATATATTTGATGTAATCTCATATTTTATGTACAGTATATAATGTATAGTTTGGGTACTTTGATATTTTATGTACACTATAGAATATATATCTGGTGTACTTTGATATTTTATGTAAAGAATAAAATATATATTTGATGTACTTTCATATTTTATGTACAGTATATAATACATGCTTTGGGTACTTTGATATTTTTTGTATAGTATAGAATATATAACTTGGGTACTTTGATATTTTATGTACAGTATATAATATATGGTTTGTGAACTTTGATATTTCATGTAGAGTATAACATACATATTTGGGGTACATTGATATTTTATGTACAGTATATAATCTATATTTGATGTACTTTCATATTTTATGTACTTAATATATATTTGATGTAATTTCATATTTTATGTACAGTATATATTTTATAGTTTTTGTACTTTGTTATTTTATGTACACTATAGAATATATATCTGGTGTACTTTGATATCTTATGTACAGAATAAAATATATATTTGATGTACTTTCATATTTTATATACAGTATATAATGCATGCTTTGGGTAATTTGATATTTTTTGTACAGTATAGAATATATACCTTGGGTACTTTGATATTTTATGTACAGTATATAATATATAGTTTGTGAACTTTGATATTTCATGTAGAGTATAAAATATATATTTGGGGTACATTGATATTTAATGTACAGTATATAATCTATATTTGATGTACTTTCATATTTTATGTACCTAATATAAATTATATTTGACGTAATTTCATATTTTATTTACAGTATATAATGTATAGTTTGGGTACTTTGATATTTTATGTACACTATAGAATATATATCTGGTGTACTTTGATATTTTATGTACAGAATAAAATATATATTTGATGTACTTTCATATTTTAGGTACCTAATATATATTTGATGTAATGTCATATTTTATGTACAGTATACAATTTATGGTTTGTGTACTTTGTTATTTTATGTACACTATAGAATATATATCTGGTGTACTTTGATATTTTATGTACAGAATTAAATATATATTTGATGTACTTTCATATTTTATGTACAGTATATAATACGTGATTTGGGTACTTTGAGATTTTAGGTACAGTATAGAATATATACCTTGGGTACTTTGATATTTTATGTACAGTATATAATATATAGTTTGTGAACTTTGATATTTCAGGTACAGTATAAAATATATATTTGGGGTACTTTGGTATTTAATGTACAGCATATAATCTATATTTGATGTACTTTCATATTTTATGTACCTAATATAAAATATATTTGGTGTAATTTCATATTTTATGTACAGTATATAATGTATAGTTTGGGTACTTTGATATTTTATGTACACTATAGATATCACATGTATTT-**chr13:69863076**

**chr13:113145057-right-clipped sequences**

TCCCCATCGGGTGGTCCCCTTCTGGCTCTGGGTTGCCTGCATCTTGCAGCTCCACACTCCGGAACATGTGGCTTCTGGCATCACCGGGGCGGGGGGAAGGGGCCAGCTGCAGCCCTTGGCTGGAACGTGTCAGGCAGCCAAGCCTATGTGCAAAGGGCCGGGACCTGAAGGAGTGAGAAGCCGGACGCTGTTGTCCCTGACCCTGAGCTGAGCAAGTTCCACTGAGGCCACAGAGATGAGTCCGACCTGAATTCTGCCCTCCCGGAGTTTACAACAGGGTGAAAGGCACCTGGCGGGTCTGGAATGCCAGCTTGGAAGGGATCGTGGGAGGAGCTGGCTGAGGAAACGTGGGCCTGGGGTGGGGGTGGCTGGGTTCCCAGGGAGGCCGCAGGTGCTGTGGTGTGCACAGCAGGGCCTGAGCCTCCTGGGGCCAGGAGGCTTCCTGGGCGTGAGGCCCAAGCTGAGTTTTCCATAGGGATTGAGAGTGAGTGAAGGCTGTTTGGAAGGACGTGCTGGGCAGTGGGAAGTTTGAGCAAAACCTGGAGACAGGAAAGAGCTGTGTGTTGAGAGAAGCTACAGCTGGTGCAGTGTTTCTGGAATGGAAAGTTCACGGCGGACGTGTGGAAGGGAGCGACTCCCAGTGGTGGGCACAGCAAGGGCCCCTCCCAAAGACGTCGGAGGCTGAACCCCCAGGACGCTTGAACAGGGCAACCTTGCAGGGGGAACTAGGTTAGGAATCCCAAGTCGGGAGATAGCCTTGGATTATTCGGTGGGTGCTCCTGGAGTTTAGGGAGGATGGAGAGGCTGCTGGACCCAGCGTAAGGTCTGGGCTCCTTAGAGGCAGGTGGGCGGGTCAAAGAGAGAGCAATTTGAAGATGCTGGGTTTGAAGATGGGCCGTGGGCCAAGGCTGCTGCCGTCTCTGGAGGCTGAGAAAAGAAAGCAGTGGCTTCTCCACCAGGCTCCTGCCAGCCCTGCCTTCAGCACCCTGAGGCCCTTTTCACACTTTCCCCTCCAGGACTGTAAGATAATACATTTGAGCTGTTGGAAGCCACTAGGTTTGTGGTAATCTGTTAAAGCAGCAGATAGGAACCTCATAGACTCACTATTGAGAAAAGAAAAGCACTTTTATCTGAGGAGCAAGTCCTTTTAATTATTGGGTCCAGAGAGGCGTTCAGTTGAGGCCGCAACCACATCCCACTCCCCCTTTGAGCTGCATGTTCATCTTGTGAAAGGAAAATCATTCTCGGGACCCCAGATCGCTGAGCCTAAGAGAAAAGTCCAGCTGGGAACTGTGCCAGGCAAACCTGCCTCCCATTCTATTCCTGAGTAAGGGAACTACAAAGATTTTAAAAAGCTACACGCCTGCCTCACGATTTGCCCACAAAGAAATTCCTTGTGGACAAAGCACAGACAGAACTCAAAGTCACCCCACTGCTCCCGTGAGACAAACGCACACAGCTGATCACTTCCTCTGCCCTCATTTCACTGAGCCAGACTCAGGCATGAGTGACCATTCCTGTAAATTGTGCATTCCGTAAAAGGCTAATCAGAAACTCAGGAGTGCAACCGTTTGTCTCTTATCTACCTACGACCTGGAAGCCTCCTCCCTGCTTCCATCTGTCCCGCCTTTCCGGACCGAACCAGCGTTCATCCGATGTATGTTGATGTCTCATGTCTCCCTAAAATATGTAAAACCAAACTCTGCGCCGACCACCTCAGGCATGTTGTCAGGACCTCCGGAGGCTGTGTCACAGGTGCGTCCTGAACTTTGGCAAAATAAACTTCCTAAATTGACTGAGCCTTATCTCAGACGCCTTTTGGTTTACAACGCTACTGAGATGTTCATCTCACCTGCAGCTGTGTATGAGAAGAGGTCGTGGCTGCATCTGGATTCGGAAAGACCCCAGTGCTAATGATTGTTACCATAACCAAGGGAAAGGACTCTAACCTGTGCGGGTTTACGGCTGCCTTCTCGGCAAGCTTGAGATTGTAGGAGCCATACGTGGCCACGATGTGGACTGCACTCTGGCTGGTGGCTGTGGCGTGACCACGGTCACTGCACAGTCACTTGGATCCCTGGGAGGAGAGTCCTGTAGCCTCGGACCGGGGATGCCACAGGCATGAACCCACCTTTGCAAAAATTATAACAGTGGAAAAATTATGGCAGTGAAAGAGACCCGAGGTAACCAACTCCCATCTTGCCTTTAGCCTTCAAACTGCCCTTAATTATTCCTGGGCTTGGGCCAAGCTAACTCTGGGAGACATTTAGTTTATAGCTTAAATGATAATAACCCTTCCGCAAAACTCAACAGCCTTTGTAAAGCTATTGAGAGACCACTAGGCTACGAGGACGAGAGGAGCCTGAATTCTGCTAAAATGGAGACACAAAGGATTACCCCCCTTTATTCTGGAGCTCAAAATATGCAACTTCCCAACTACTCCCGCAGATAACGTCACTACGGTGGAACCTAAGACTGGCCTTTGAGATGTCTTTTCAGGTTTTTTGCAAGTCTGACAACTGATTTCCCCCCCTGGACCGGTCAGCCTCCCTGGTGCCACCCAGAAGCGACTCGGGGCACAAGGACCATTTCCCACACCCCGACGACTGCACCCCCAACCAGTCAGCAGCAAGCGCCCTTGCCTACCCACCCGACTCTCTTCCCCTAAACTAGCCTTGAGAGACCCTGGTCTCTGCATTTTCAGGGAGGCTGATGTGAGTAACAATAAAACTCTAGTGTCCTGTTTAGCCGGCTCTGCGTGTGTAAAACTGTTTCTCTATTGCAGCTCTCGCCACTGGCCAGGAAACGGTTTCCCCCTCCTCGGGCTGTGCTGGGAGGAAGGGTCTGAAGTGGCTTGTAATGTCGGAAGCAAAGTGGAGGACTTGCCCGGAAGAGGGAGGAAGGCCTTGGCTCCTTTGAGCCAGTGGGAATGGAAGGGAAGGCGGCCAGGATTTCCTCAAAAATATTTCCCCATAGCCGGTCAGCTCCCCACGGGGCCAATACCGCATCCTCACTCTGGGCGCAAGGACGGCCCCTTCCCAGAGCCCATTTAGAACTTGGATGAATGTTCTATCAACACTCATGGATGTTGCCTGGATGAATGGGCTCTATCCGGGCCTTGAGTAGGAAGAAGCCGCTGGCAGTTACTTGCTCACCCAGAGACTCCAAGCAGCCACACTCACCTGGGCTGATGACTCAATTGACTCCTGAGTTTCTCCGGCTCTTCATTGAGGTCTGTCTTTTCCACCTCCATCTTTGAGCCTCTGAACTTGATGAAAATCGGTTACATGTAGATGGCTTTTCTGTCCAATGCTAGAGAACTTTGAACTTAATGGAATCCTGCCGTCTTTTCATCAACTCAGTGCTAAACAACTCTAGATTTTAAAATGATATCCAGTGTGACTGCGTCCATTTCACATGGCACAAGGATTGTCTGTGATCACGATTCATAAAGCTGCTATTTTTTAGCTGACACTAGGCCCAAGCATGGCCCCTGGGGGCACAGGGCTGTTTATTTCTCTTTTAAAGCACCCTAATCTGGGAGGTGTATTTTGCCGGTGGCTGGCGGGCTGGGAGCAGAGAAAAGTTCGGTGTAAAAATGAAAATGCATTCCCGGCAGGAAAACTCCCACCCTAAGGGCACGTATGCAGTTTACCCCAAAACAATGGTTGCCGTGGGTAACCCGAGTGGTGTTGCCAGTGGCTGCCAGTTTGGCCCTTCCAGGGAGATGTCAAGAGACCCCTTCTACCAATCCCTTTCCTCATTAAAGAGGAAGAATGTTTGCTTTCTGGGAGATATTTAAGAGGACAATTTGAAATCCCACAAAAGCCAGGGTTGGTAGAATCAGGACTTGGCTCAGCCCAGCTCAGCCCCGCAGCAGCTACCCCGGGCTCTGTTTGTCCTGGCTTCCACCAGGGCCAGCGCTTCACAGCCGGAAGCAAACAGGCCAAGCATTTCTTGGTTAATAGCACAAGGGGGCCAGGCTTGCTGGAGAAGCAGCGGTCAGGGCCAGGGGCCCCTAACCTCTGCAGTCGCCCCTCCCAGTGGCTTTCCAGAGCTGTTTTCCACCTCTCCCTGAGGAGCTGAAGGGCGCCGGGGGCCGAGTCACTGCCACAGCCGCAGGGACCAAGCCCCATCCCAGCAAGGCTGAGGCCTCAGGAAGGCGGCCAGCCCAGGCCCTCTCGCCACTGGCCAGGAAACTGTTCCCCCCCTCGGGCTGTGCTGGGAGGAAGGGTCTGAAGTGGCTTGTAACGTCGGAAGCAAAATGGAGAACTTGCCCAGAAGAGGGAGGAATGCCTTGGCTCCTTTGAGCCAGTGGGAATGGAAGGGAAGAAGGCCAGGATTTCCTCAAAAATATTTCCCTGTAGCCAGTCAGCTCCCCACAGGGCCAATACCTCGTCCTCACTCCGGGTGCAAGGACAGCCCCTTCCCAGAGCCCGTTTAGAACTTGCTTCTCTTCAGACTACATTCCCTCCTGGCTGGATTCTGGAGACCATAACTGGGGCACCTACCAAGTGGGGCCCGTTAGAGGTGCTGGCGGAGTTCCCGTGTGACCCCATCAGGGTGGCTCACAGCACATCTCCGCAGCGACACGCTCAGGTCTGGTTTTCCAGTGTGTATGCGTGGAGCCCAGCAGTGGGGCCCAGATGGCGTGGTGAGGATCACCACTGAAAGGCAGGCCTCGAGCACTTGCCCAGAGTCCACTGTTTGGAGGGCAGTTCGATTCACCGTAGCCAATTAGCCCACAGCACACACAATCTTTCTTTTCCCATTGAGTTGTCTTGGCACCTTGGTTGGAAAGTAATTTACCATGTATGTGTGGGTCTATTTCCTTTATTTGAAAGTGATTTTTAAAAGGAAAATTGCAAGACCCCTTCTTCTAATTGTGGCCCCACACCCTTGCCCATGCATCCGACTCATCTCCATCCATCCTGTGTGTCTGGTAGTTTCATAAAAAGTCAAACACATACCTGCCATTATGACCCAGCGATTGTACTCCTGGGCATTTATCGCAGAGAAATTAAAATGTATAGTCACACAAACGATCATAGCAGCTTTATTTGGAACAGCGAAAACCTGGGAACAGCCCCATTGTCTGTCAGCAGGTGCGTGTCCGACGCATCGTGGTGCCTCCACACCACACAACGTGCTCAGCAACGGAGAGAAACAGACTCTGGGAGAAAACTCCATCTCAAAGGCTGCATTCCGCACTCCGTTCCCATAACACTCTTGAAAGGATGAGTGACGCCGGCGGGTGCTGTGGGTTAGAGAAGGGCCCCGCGGGGCACACCTCACACCTGTGGGGATGGACGGTCATGCATCTCGAATGTGGTTGTGGTTACAGAAATCCACATATGCGGCCACACATCGCAGAATCTCCACACGGAGTCGTACCCAGAATAAAACAAACACACGGAAACCTAGGATGTCCAAATGAGCTCCGTCATCCAGTGACTTGCGTGGTGCCCATATCAGTTTCCTGGTTTTAAGAATTTGTTGTAGCTCTGCAAAGTGCTGCATTGTAGGAAGCTGAGGAATGGAATGTGGGACCCCTCTGTACTATTCGGCAACTTTTTGTGAAGCTATAATTAGCTCAAAATGCAAAGCTAAAATTACTGAGGACTTAGAAATAAACTTGACAAAAGATTTACAAGTCCTATATACTGAGAACTGAGAAACATCAATGAGAGGAATGAAAAAAGGCCTAAATAAATGGAGCCATAGCATGTTCATGGAAGATGCCAACTCTCCCCAGATTGATCTCCAGAATCAACACAATCCCAATCAGAATCCCAGCAGGATTTTTGTAGAAATTGGTAAGCTAATTCTAAGGTTTATACAGAAATGCACAGGACTTGGAGTAACCAAAGCAAGCATAAAAAAGAACAGAGTTGAAGAATTTATATTACTTGATTTTAATCAAGACAGTCAATATAATCATTATAACAGATAAATAGGTCAATGAGACAGAACAACACCCAGGCCTACTCATCTATGGCCACTACTCATTTATGGCCAATTGACTTTTGATAAATGTGCTGAGGCAATTCAACAGGGAGAGAATAACCTTTTCCACAAGGAATGCTGGGTCATTTGGACACCCATAAGCAACAAAATAAAACAATTACAAATCCTCAAAACCCTTACCTCACACCACATACAACAATTAACTTTGGGCTGGATGCAGTGGCTCACACCTGTAATCCCAGCACTTTGGGAAGCTGAGGCAGGTGGATCATTTGAGGTCAGGAGTTCAAGACCAGCCTGGGCAACATGGTGAAACTCTGTTTCTACTAAAAATACAAAAATTTTCTGGGCATGGTGGCATGTGCTTGTAATCCCAGCTACTCAGGAGACTGAGGCATGAAAATTGATTGAACCTGGGAAGTGGAGGTTACAGAGAGCCAAGACCACGCCACTGCACTCCAGTGTGGCTGACAGAGCGAGACTCCATCTCAAAAAAAAAAATTGTAATAGGTCATAAATCTAAATGTGTGAACTAAAACAGTAAAACTTGTAAGAAGAAGATAAGATAAAATCATGGTGACTTTTAGTTTGGGAAAGGTTTTTTAAATAGAACACAAAAAGTTCAAACTACATGAAAAAACCAATAAGCTAAATTTTATTAATTTAAAACTTTTGCCCTTTAAGAGACACTATAAAGAGGCAAGCTGCCAAGCTGGAGAAATTATTTGCAAACCATGTTCAAAAAGGACTTCTTTCTAAAACATGTCAAGAAGCCTTTCAACACAATAATGAGACAAAACAATTCAATTAAAAAGTAGGCAAAATATTTGAATGGATATTTCACCAAAGAAGCTATATTAACAGACAGAAGCACGCAAGACGATGCTGACAACCATTTAGTCCTTAGGGAAGTGCAAACGAAGACCAGTATGAGAGGTGCCTACAAACCCACCAGCATGGCTAACATGTTCAAAAGTGGCCCTGCCAAGGGCTGGCGAGGATGTGGGGCAACTGGAGCGCGCAGCATGTTGGCAGGATCATAACACAAGAACCACAGGGAACGGTTCGGCCATTTCTTAAAAAGCTACACAGAGTCACCATGTGACCCAGACATGTCTGTTCTGGGAACGTCCACAGAAAGACTGTGTCGTAAACGTTCACAACAACCTTGCCTGTAATCACCAAAATCCAAAACAACCTGAACGTCCTCCACGGGTCAGTGGATGAACCAATTATGATACACCCATGCAACTAGTGCTCCCCAGCATTGAAAGGATGAATTGTTGGATGAATCTCAAAATAATTACACTGTGAGACAATGTCAGGCAATAAGAGTATACACGCTGTGTTACCCAGGACAGAAATGAATTAATCTACAGTGACAGAAAGGTCAGTGTCATCAGAGGAGCAGGAGGGTGGAGGGACGGGGCAGGGGCCAGCCTGGGGGAGGGGATGTCCATGGTCTCGATTGTGGTCATGGCTCTGTGGGTGCAGCAGAATGTCAAAACTTATCAAACCGTACGTCTTAAATATGGGCATTTAATTGCATGTCAAAAAAATCTAAACAAAGCTGAAAATGGACAAAATACCACAACCAATGACATCTGACCGTAAAGAGATGGATTTAGGTAGAAGGAAGAATTCTTTAACACAGAATATGGGTCTCTGAGGGAAATGTTATCTCGTAATTTTCTCACTTTTGGTGTATTTAAGCTGTAGGACAAGGAGTGAACCCTAACATTTGGTGAGAGTCTGTGAAACACACTAGGAACCCCCATGCTGCTTGCGAGTTACTTCCGAGAAACAGCAGAATGTCACACCCTGTCACCCGCAGGTGCCGTCTGAGGGGCGCTCATCCCCACCAGCCCGAGGGCCTCCCCGCCAAGGGAGGGGTGTACTCCCTTCCACTTGGCGTCTGCTTGTGCATTGCCCCTCCCTGAACTCAAGACCTGAGCCTCTCTCGCCTGGACTATTTGGAAAGTCTACAAATGGAAGAAGGGGTGCTCTCACTGAGTTGTGTCCGCACAAGGGAAACTGTCCAGGAGCCACAGAAGCAACCGCAAAGTGAGCCTCTCAGACGTGGTTAAAGGGAAGCCAGGCAAAAAGACTGCACGCTGTTGGGTTTCATTTACTCAAAACCCTAAAACAGGCAGAATGAGTCATCTGTGGTAAACCAGCATTGGGGTTACCTCTGGGGGCCGGGGGGAGGGAACCCTGGGGTGCCAGTCACGTCTGGCGTCTCGGCCTGGGTGGTGGGGATGGCTCGCCAAGCCTGTTTGTTGCACACGTGGCATGTCAGCAGAAACACGGGGCAAATACACACGTGAACACAAGAACCCCGGGTCCCTCCCTCATCTACCCAGCGACCCTCAGTCACCCCAGCCGGCTCCCTCTCGCCACCCACGGGCTTTCTGTGGAGGCCCCAGCCTGGCCCTGGAGGCCGTGACCCACCCCGCCTCTTGCCTGAGGTCAGCCCAAGAGACCTTCCTGTCTTCGGCCTGGAGAAGGAGCGGCCGCCAGGACCACCCGGGCCTCAGCTCCCTGGGAGGGCCCCTGGAGGACCGGGGCTGCTAAAGTCCACGGGCATCACCGCATCTAATCCAGGCCCTTTGATGTGGGT

**Supplementary File 4C. GS15-RC14**

**Two-break and two-fusion for GS15-RC14 (non-template insertion)**

**14p11.2(-)** 5788045-TGGGTTCTCTGAAAGGTCTAAAGATGTCAGAGGATTGTCC-5788084

**14p11.2(+)** ACCCAAGAGACTTTCCAGATTTCTACAGTCTCCTAACAGG

**14q32.33(+)**  366910-ATCCTTGATCTCCATTCACCACTTTCTTGGGTTAGCTCCCA-98366950

**Fusion sequence 1 (f1):** CCTGTTAGGAGACTGTAGAACTAACTTTCTTGGGTTAGCTCCCA

**14q32.33 (+)**  100117110-GGGGAACACATCCGGAGC**CTT**GGTGGGTGCTGGAAGACAGA-100117150

**14q32.33 (-)** GTCGTCTTTGGTCCTCATCTCTA**TTC**CAATAACTCCTATAT

00119960-CAGCAGAAACCAGGAGTAGAGAT**AAG**GTTATTGAGGATATA-10012000

**Fusion sequence 2 (f2):** GGGGAACACATCCGGAGC**CTT**ATCTCTACTCCTGGTTTCT

**ISCN:**

seq[T2T] r(14)(p11.2q32.33) g.(pter)_5788064del::ins[CTA]::98366930_100117130dup::100119982-(qter)del

**a.** **CNV analysis showing a 5.79 Mb distal deletion of 14pter-p11.2, a 1.75 Mb duplication at 14q32.33, and a 1 Mb distal deletion of 14q32.33-qter. Below, IGV view of the junctions.**


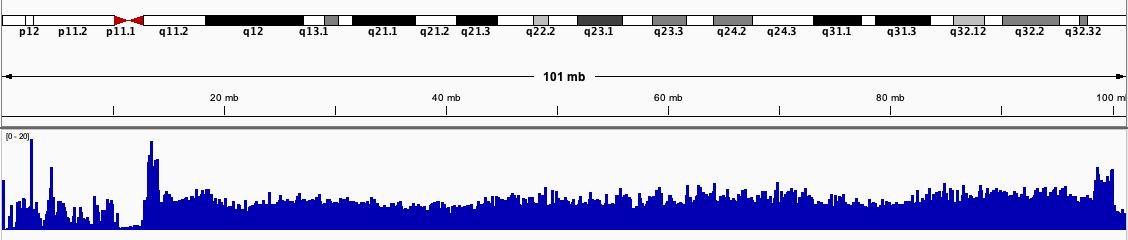


Chr14:98366930 left-clipped sequences


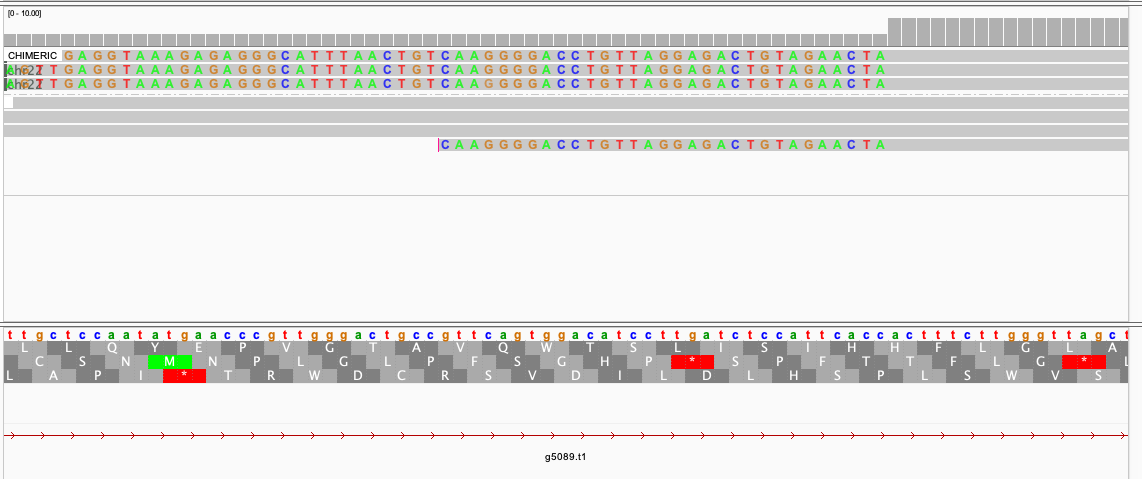


Chr14:100119982-right-clipped sequences


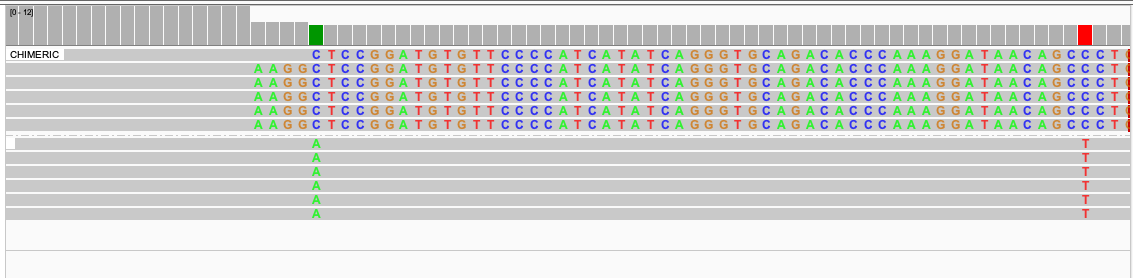


**b. BLAT search results to T2T**

**The 10.055 kb chr14:98366930 left-clipped sequences match to chr14:2903154-2913367(+) (identity: 96.2%) and chr14:5788065-5798105(-) at 14p11.2. These sequences are centromeric transition regions (ct) within other centromeric satellites (censat).**

TTAGGAACAATATCACAGAGGGGGTGTACAACTTCTGTGATATTATAACATTCTTTCTTCCCATGGATATTAGGAATGATATCCCGGGCGGCTTGTTGTACACCCCCTGTGATACGGACAGTAATATCATTGTCCTTCCCCCTACATATTAGAAACAATATCACAAGGGTGGTATACACCCCCTGGATATTAGAAACTATCACAGGGGGGCTGTACAACCTCTTTGATACTGTGAGTAATACCATTGTCTCCCCTCCTGGGTATTAATAACAATATCATAGGGTGGGTGTACACCCCCCTGCAATATTGGGAATAATATCATCCTCTCTTCCCAGGGATATTAGGAACGTTATCACAGGTGGGGTTTACACCCCCTGCAATTTTGTCAGTAATATTACTTCTGGATGTTATTGAATATATCACAGTGGGGGTGTACAACCCCTGTGATATGGGGAGTAATAGCATCCTCTTTCCCACTGGATACTACAAACAATATCGCAGATTGTGTACAACCTCCTGTGATATTGTTCACAATATTTAGGGAAGGAGAGGATGATATTACTCCACATATGGCAGGGAGTGTTACATCCCCTGTAATATTGTTCATAATATTTAGAAGACGACAGGATGATATTACTCCCAATATAGTAGGAAGTATACACTCCCCTGTGATACTGTTCATAATTTTTAGGGGATTAGAGGATGATATTACTTCCAATATCACAGGGAGTGTACACTGGTGATATTGTTTATAACTTTCAGTGGATTAGAAGATATTATCCCGAATATCACAGGGGTTGCACACCCCAAGTGATATTGTTAATATCCAGTGGGAAAGAGGATGATATTACTCCCCATATCACGGGGGATGTAAACCCGTTTGTGGTATTGTCACTTACATCCGGGGGGGAGAGGATGATATTACTCTGCATATCATAGAGGGTGCACACGGCTGTAATGTTGTCCATAATAACATCCAGAGGGGAAGAGAATATTATTCCCATGTTTCAGAAGGTGTACACACCCCTGTGATAGTCTCTGTAACATTTAGGGAAGAAGGGGATGATACTACTCCAGATATTGCAGGGGGTGTACATCCCCCTGTGATACTGTTCGTAACGTTTAGGGGGAAGAGGATGATATTACTCCCCATATCGAAGGGATTGTACATCTCCCTATATATTGTCCATAACATCCAGGGCAGGAGAGGATATTACTACTCCCCATATCACAGGGGGTGGACACCCCCCTCTAAATATGTCTAACATCCAGGCGGGGACAGGAGGATATTTTTCCCCATAACCCAGAGAAAGTAAACCTCCTGTGATATTGTCCATAACATCCAGTGGGGAGAGGATGATATCACTCCCCATATTGCAGGGGGTGCACACTCCACTCTGATATTGGCCGTAATATCCGGGGGGGGGGGTGAAGTATGAAGTCACTACACATATCGCAGGGATTATTAGTATCAGATTGTTTGAAGGGCTCACAGTAAGGGTAGTAGTAGGGCGAGTTCTAACTCAAATAGGGGAAATGTGATGTCTACTAGAAAGAATTTTATGGAGAAGGGAATGTGGGCAGAGGATAGAGGGTCAAATCTGCATTCATAAGGGCTAGATTTTTCTATATATATTTATTTTATACATATATATATTTTTTTCTCTCTTCTCTACATATATATATTAAGTTGTGGGAGCCAAAATGTAATAATTATTAGTAACAGGGCTAATAGGGTGTTGATTACTAGGGTTAATGTTAGGTGAATTACTGTTTTTCGGATGCTATCAAAACTTTGGAAATCATGGTACTATTTATACTAAAAGAGTAAGATCCTCATCAATAAATAGAAACATACAAGAATAGTCATACTATATCTACAAAGTGTCGATATCAGGCAGCGGCTTCAAAGGCAAAATGATGACTAGATGTAAAGTGGTATTTTAATTGGCGGAGAAGGCAGACTGAGGAATGTTGATCCAATAATGATGTGAATTCTGTGAAAGCCTGTAGCTATAAAAAAATGTTGAGCCATAAATACCATCAGAAATAACAAAGGGAGCTTTGAAGTATTCTGAGACTTGTAGGAAGGTGAAGTAAATATCTAATATAATTGTAACAAGTAGTGCTTGGATTGTATGTTTTTGATTATTTTTTGTTAGGCTGTGATGGGCTCAAGTAATTGAAATTCCTGATGCAAGTAATACAGATGGATTCAGGAGAGGTACTTCCAGGGGGTCAAGGGGAGAAATACCTGTTGGGGGTCAATGCCCTCCTAATTCTGGAGTAGGGGCTAGGCTAGAATGGTAGAATGCTCAAAAGAATCCAGCGAAGAGGAATATTTCTGAGATAATAAATAGGACTGTCCCATATTGGAGGCCTTTTTGAACAGTTGTTGTATGGTGACCCTGAAATGTACTTTCTCAGATACAGAACACCCTTGGTCAATTGAATACAGATCAATCACTTTAAGTAAGCTAAGTCCTTACTAAATTGATGAGACTTAAACCCATGAAAACTTAACAGCTAAACTCCCTAGTCAACTGGTTTGAATCTACTTCTCCAGCAGCTGGGGGAAAAAAGGTGAGAGAAGCAGGATTGAAGCTGCTTCTTTGAATTTACAATTCAACATGAAAATCACCTTGGGACTGGTAAAAACAGGCGTTGACCTCTGTTTTTAGATGTACAGTCTAATGCCCTACTCAGTCATTTTACCCTTTTTTCTCACTTCATTTATGTTGGCTGACAGTTGACTATTCTCAACCAACCATAAAGATATCGAGACATTATATTTATTATTTGGCACATGAGCAGGGATAGTCAGTACAGCTTTAAGCCTTATTCGAGCTGAACTCTACTAGATGATCAAATTTATGTCATTGTTATAGCCTATGCATTTGTCATAATTTTCTTTATGGTAATACTATAATTGGAGGTCTTGGCAACTGATTAGTCCCCCGATAATTGGCGCCCCCGATATAGCATTTCTCTGCATAAATAATATGAGCTTCTGACTCCTCCCACCCTCCTTCCTTTTATTACTTGCATCCACTATAGTAGAAGCCGGCACTGGAACCGGCTGAACAGTCTCTCCTCCCTTAGCAGTAAACCTAACACATGCAGGCGCCTCTGTAGATTTCACTATCTTTTCACTCCACTTGACAGGTGTTTCTTCTACTTCAGGGGCTATTAACTTTATTACCACAATTGTTAATATAAAACCCCCAGCCATGTCCCAATATCACACACCCCTCTTCATCTGATTAGTCCTAATTACAGCAGTTCTTCTACTCCTTTGTCTCCGAGTCCTAGCCGCCGGCATCACTATATTGTTAACTGACTGCAATCTTAATACTACTTTTTTCTATCTGGCTGGCGGAGGTGATCCTATCTTATTTCAGCATTTATTCAGATTCTTTGGTCACCCTGAAGTCTACATCCTCATCCCACTGGGCTTTGGGATAATTTCCCACGTCGTAACATACTATTCTGGAAGAAAAAAGAACCATTCAGGTATATGGGCCTAGTGTGAGCTATAGGATCAGTTGGGTCCTTACGGTTTATTGTATGGGCCCACCGTATATTTACGGTAGGGATAGATGTGGATACATGAGCCTGCTTCACCTCTGCTATTATAATTATTGCTATTCCTACTAGCGTCAAAGTTTTTAGCTGACTAGCTAGCTACACTTCACGGCGGTAATATCAAATGATCCCCCGCAATGCTGTGAGCCCGGGGATTTATTTTCCTTTTTACAGTAGGAGGCCTAACCAGCATTGTATTGGCCGAGGCTTATATTATGGTTCATTCATATATTTAGAAACCTGAAAGTTTCTAAATAAGTTGTAAAAAAGTTGTAAAAAAACCCCAGCTGAAATAACTACGAAGGTGCCTTTAATATTCTGAAGACAAAATAGCTAAGATCCAAACTGGGAGTAGATACCCCGCTATGCTTAACTCTAAACTCGAATAGTTAGATCAACAAAACTGTTCGCCAGAACACTACAAGCAACAGCTTAAAACTCAAAGGACTTGGCGGTGCTTTATATCCCTCTAAAGGAGGCTGTTCTATAATCGATAAACCCCAATTTACCTCACCACCTCTTGCCCAGCCTAAATACCTCCATCTTCAGCAAACCCTGGAAAGGCCGCAGAGTAAGCACAAGTATCTACATAAAAACTTTAGTTCAAGGTGTAGCCCATGAGGTGGCAAGAAATAGGAACGTTTTCTACATCCAGAAAAATGTCGTGACAACCGTTATGAAATCTAAGGGCTCAAGGAGGATTTAGCAATAAATTGAGAGCAGAGTGTTTAATTGAATAAGGCCATGAAGCATGCACACACCGCCCGTCACCCTCCTCAAATACATTCTAGAAACTCATTGTACACTCCCCTGTGATATTGTCCATAATATCCAGGGAGGGAGAGAATGATTTGATTTTTTTTTTTTGAGACAGAGCCTTGCTCTGTCGCCCGGGTTGGAGTGCAGTGGCCTGATCTTGGCACACTGCAAGCACCGCCTCCCAGGTTCACACCATTCTCCTGCCTCAGCCTCCCTAGTAGCTGGGACTACAGGCGCCCGCCACCACGCCCGGCTAATTTTTTGTATTTTTAGTAGAGACGGGGTTTCACCATGTTAGCCAGGATGGTCTCGATCTCCTGACCTCGTGATCCGCCCGCCTCGGCTTCCCAAAGTGCTGGGATTACAGGTGCGAGCCACCGCTCCTGGCCAGAGAGAATGATTTTACTCCCCATATCGCAGGGGATTTACATTCCCCTGCATTATTTTTCGTAATATCCAGGGGGAAGATGAAGATGTTACTCCCCATATAGCATGGGAGAACAATTCCCTGCGATATTGTTCATAATATCTCTGGGGGAAAGAATTATATTTCTCCTTTTATCGCAGGAAGTGTACACCCCCTTGTGATATTGTTTATAATATCTAGTGGGGGAGAGGATGATGCTACTCCCCATATTGCAGGGGGTGTACAACCCCCTAGAATATTGTTCATAATATCCACGCGGGGAGGAGATGATGTTACTACCCATATCGCCAGGGTGTACTGCCCCCTGCCATATTGTTTGTAATATCCAGGCTGGGAAAGGATGATATTACTCCCTGTATCACAGGGGATGTACACACCCCTGTGATATTATTAGTAATATCCATGGGGGAGATAATACTACTTCCAATACCATAAACACCCTGTGTGTACACCCTCTGTGATATTGTTTGTAATATCCAGGGTGGGAGAGGAGTATATTACTCCCTATAAGGCAGAGTGTGTATACACCCCTCTGTGATATTGTTCATAATATCCACTGGGGGATATGATATTACTCCCAATATCATAAACACCTCACATGTACACCGTCTGTGATATTATTTGTAATATCCAGTGGGGGAGAAGATGATTTTACTTTCCACATTGCAGGGGTTTACACCTCTCTGTGATACAGTTTGTAATATCTAGAGGGGGAGAGGGTTATATTACTCCTCATATCTCAGGACATGTACACCCCCTGTGATATTGTTTGTAATATTGTTCCCAATATCCTTTTCCCCCATGGATATAGGAACAGTATCCCATAGGACGTGTACACCCCCTGCCATATTGGAAGTAGTAGTGTTTTCTCCCTTGCTGGACATTAGGAACAATACCATGGGGGGGTGCACACCCCCTGTGATATTGACAGTAATGTAATCCACTATCCCCTAAATATAGGAACAATATCACAATGGTGATGTACACACTTGGTGATATTGAAAGTGATATGATCCTCTCCCCACCTGAATATTGGGAACAATATCACAGAAGGGGTGTACACCCCCTGCGATATTGACAGTAATATCCTCTCCCACCCCCGGATATTAGGAGCAATGTCACAGAAGGGTTGTACACTCCCTGCGATATTGACAGTAATATCCTCTCCTCCCCGGATATTAGGAACAATATCACAGAAGAGGTGTACACCCACTGTGATATTGACAGTAATTTCCTCTTCCCCCCCGGATATTAGGAACAATACCACGGGGGGTGTACACCCCCTGCGATATTGACAGTAATATCATCCTCTCCCCGCCAGATATTAGTAACAATATCACTGAAGGGGTCTACACCCCCTGTGATAGTGACAGTAATATCCTCTACCCCCCAGATATTAGGAACAATACCACGGGAGGATGTACACCCCCTGTGACATTGACAGTAATATAAACCTCTCCCCCCACCCTGGATATTAGGAAGAATACCACGGGGGGTGCACACCCCCTCTGATATTGGGAGTAATATCACCCACTATCCCCTAAATATTAGGAACAATATCACAGGGCGGGGAGTACACCCTCTGCAATATTGGGAGTAATGTCATTTCCCTGCCCCTGCATACTAGGAATAATATCACAAGGGATGTACACCCCCATATGCTCTTGGGAGTAACATCACTCTTTCTTCCCATGGATATTAGGAACAATATCACAGAAGTGGTGTACATACGCTGCACTGTATAGAATAAAGCAGGCGGAGGAAGATGGGATAACCTTGCTAGCTGAAGCTTCTGGCTCTCTTTTTTTTCTTCTTCCCGTGCAGGACACTTGCTTCCCTTCTTCCTGCCCTCGGACATGAGACTCCAGGTTCTTATGCCTTTGGACTCTGGGACTTGCACCAGCGGCTTCCCCGAGGCTCTCAGGCCCTCGGCCTCATACTGAAGACTGCACTGCGGGCTTTCCTGGTTTTGAGGCTTTTGGACTTGGACTGAGCCACTACTAGCTTCTCTCTTTCCCTACCTGGCAGACAGCCTATTGTGGGACTGCCTTCTAACCGTGTGAACCAATTCTCTCTCGTAAACTCCCTTATACATATACCTGTATCTTGTTGGTTCTGTCCCTCTGGAGAACCCTGACTCATACATTTTGTTTATTTTTTCTCCATTGTCCTTTCCTCTGCTTCTAGGCTTACCTAGACCACCACCATTCTTTCCCCCTTTCTAAAGTAAAAGTTGTCTTTTTCTCACTAAATGCATGGCATTCTGCCCGTTTTCCATGGCTTTCCTCAGCCCTGCTCTGTTTATTCTTGCTATCTTAAGAGGAAATCCCTGCCTCTTCCGTGGCTTTTCCCACTTGGTCTACATACTGGTTTCTGTTGTTCTCAGAGACACACTGGGACCTTTCACATCTCACTGTCACTTCTTGGAAGGGCTCTCTACCTCGTCTGCCTGCTGAGCAACCTCTTGGGGAGACGCGGGCCCTCTTGAGTCACTGAACTTGAGCTATTTGGTGTTGGTATGTTAATTTATCTTCTTAGACCACTTACCACTTCTTTAACTTCAAAAGAGAAGAATAAGTATTATTTTCCATGGTTGATATGAAGAGTAGAAATAACTTATACAAAATGCATTGCAGTTAAGTGATAATAAATGGCCGTGAATGCCCTTATTAATGTTATTCTATCAGTCTCGGCTCAGATACCATCTGCTCTGTAAGCTCTGCTCTGAATCATATCTGCTTCTTCTCTGGGTTCCTTGTGCTCTGTTCTTAACTACTTTAAAGCAGTAATTGTTCTGATTCTAATTAGTGATCCTTCCCAATAAAATTTTAAATTTGTAGATCTCTTCCCCCTACCCCTGCCCCAGCCTAACCAGTGTTTTCTTACTTTTTTGTGTACAGGCTACATCACTGGTCTTTCATTTGTGGCTAAATAAATGTTGTGTTAGAAGAGTAAAGAGTTCCCAGTTACATGGGATCTATAGTTCTACAAAATGAATGTATACATAATCCATGTAAATATTCCACTTATTTTAAAACAATTTTTTAATTTTTAAATTAAATTTAATTTGTGCATGTGTGTGAGACCAGAGTGAGACCAGAGATGGCGGCGGTGAGGGGCGGTGGTCTCACCATGTTGCCCAGGCTGGTCTTGAACTCCCCTTGAAGTGCCCCCCTCCTCACCTGGCCCCCCTCACCTTGCCTCCTCCCCCTCACTCCTATGCCAGTCCCTGCCATTCCCTACCCCCTCTGCTGACCGCAAGACTCAACAAGTGACTTGCTGAGCAAACCCTGCTGAGAAGAGGTCTGTTTAGGGACACAGGAGGCCAAGTACACAAAAAAGCAAAAGAACTAGCATGACTTTTTCAATGGATGTCTATTTTACAGGGCTGGCTTCAGATTATTGTTATAGCTTTAAATAAAAGGACCGTTTTGTCATCTCGGCCCATGGCCTACATTATTTCTTTACTGTCCATTGCCCTGGGCGCTTGACTAATAATTTAACAGCAATTTTTTTTTTAAATTTTAAATCATGATTCATCGCATTGCTGTAAGAGTAATTAGAGGTAAATTAGGGCTTGAAACTGCCTGTAGTGGGTTACTTTCTGAGATCTTAGCATAATTATCAGGTGAGAGGGTGAAGTTTTAATTAGCGCTAAGTGGGATAGAAATTCAATGCACTGAAACTGCAGTGTCCAATTCAGTAGGCGCTAGCCACCTGTAACTATTGAGCACTTGAAATCCTGGCTAGTCCTAATTGAGATGTGTTGTGTTAAATATACTGGATTTTGTCATTGGAGTGGGAAGAACAGCGTAGAATATCTGCTTGATAATTTTTATATTGATTACATGTTAAAATTATTACTATGATTACTATTTGGGACATACTGAGTTAAGTATATTTAAAATTAATTTCACCTTTTAATGGGGCTTACTAGTACATTTAAAATTACATATGTGGCTCACATATTTATTGGACAGCACGGCTCTAGAAGTTTAGGAAGAAATAAGAAAAATATTGAGAATAGGTAGCAACAGTAGGAAGTTTGACCTCCTGTAAGACTGATTCCAATAAAACTAATAGGTGATAGTTTTAATTGGCTTTTTCCTACTAGAAAAAGTAAGTGTACTTTACATGTCTCTTTGTTCTCCCTCTTTCCCCTTCAATTTAGTGGTTAGCGTGTATTTACTATATCAGGCTTAATATTCACTAAGCAGTGTTAAGAAGACTTAGGTAAATGATTCCCTGATGAACACACTTGATTTTCAAAGCACTTTCCTAACCCATTTTTAATTGGAATAGAGTCAAAGGTAGATGGCTTATTATTAGTCATCTCATTTAAACCTCATGGAATTTTCCTGCTAAATCTCAAAGTAAACAATTTGTTATAGGCTGTTTTGTCAAGTGCATGGAAGGGACAGCATAAGACATGTGGTACTTCATTTCAAAATTGCTTGAGATGGTTTTCATTATAATCATACATTATTTGCTTCTGGTTTTCCAGAAAAGCCAGCTAAACCTGTGGTTCATTTAGATATAAATGAAATATCCTCAAAGGATTGCTCAAAGTGACTTATGCAACTTGAATATATATTTTTTTCTGTAGGGTGTTTATTCCAAATTATCTGGCCATTTGTGGCAATTTGCAGTTTTTTAGAAAACACCAAATATTTTTCAAGTTAGAGATGTTTTAATAAAAACAGTCATATTGAACTGGAAGCAGCCAAATAAAATGGTCCTTTATTTCACAGTTGAGAATTTGAAATTGGAAGGTAATTAACATATGTAAGGATGATGTATTTGCTGCCTGGCTTATAGGAATGAGCCAAGGTGTTTTTTGAGGGATGTTGTTGCTGTGTATGTCCCACCTATTTTGCTTCCTTAATGAACAATGCAAGTTTGAGACAGAAATATTTGAGAACATTTTTATCAGTTGCGACATTTTGATAGTGAACATTTTATATCTTCTGTAAACTTAAAATGTTTGCATCTTCTGTCTGCTTAAAATGTTTAATTTGTTATACCCAAATAATTTGGCTATAATTGATAATTTAATTTATCAAATTAAATTGTTTTTTTTCCCTTTAGACTTTCTTCAGTCACATCTGAATAAATCACTTAGAAGTAAGCTAGATGTAATATAATGAAATGCTTAAAAGGGCTGTGTATCTTCATATTACACTTACAGTGACTTTCTGCTGTACCCATTATATTCTACCTGCAGCTAGTTGAGGTAAAGAGAGGGCATTTAACTGTCAAGGGGACCTGTTAGGAGACTGTAGAACTA- chr14:98366930

**This 2.609 kb Chr14:100119982-right-clipped sequences match chr14:100114516-100117130 at 14q32.33 (identity: 99.9%). The breakpoint is at exon 1 of the *IGHD* gene.**

AAGGCTCCGGATGTGTTCCCCATCATATCAGGGTGCAGACACCCAAAGGATAACAGCCCTGTGGTCCTGGCATGCTTGATAACTGGGTACCACCCAACGTCCGTGACTGTCACCTGGTACATGGGGACACAGAGCCAGCCCCAGAGAACCTTCCCTGAGATACAAAGACGGGACAGCTACTACATGACAAGCAGCCAGCTCTCCACCCCCCTCCAGCAGTGGCGCCAAGGCGAGTACAAATGCGTGGTCCAGCACACCGCCAGCAAGAGTAAGAAGGAGATCTTCCGCTGGCCAGGTAGGTCGCACCGGAGATCACCCAGAAGGGCCCCCCAGGACCCCCAGCACCTTCCACTCAGGGCCTGACCACAAAGACAGAAGCAAGGGCTGGGCTGTGAGGCAACCCCCACCTCCCCCTCAGAGCACGTTCCTCCCCCTTCACCCTGTATCCACCCCTCCGGACCCTCCCCATCTCAGTCCCTCCGCTCCCTCTCTCTGAGGCCCATCTCCCAATACCCAGATCACTTTCCTTCCAGACCCTTCCCTCAGTGTGCACGGAGGCAGCTTGCCCAGCAAAGGTGACTGTCTAGTGGGCTTCCCACAGCCAAGCTCCCACCCCATGCTGCGGCCCCTCCCTTCTTCCTGCTTGGCTGCCTGTGCCCCCCACCTGCCTGTCCACAACCCAGCCTCTGGTACATCCATGCCCTCTGCCCTCAGCCTCACCTGCACTTTTCCTTGGATTTCAGAGTCTCCAAAGGCACAGGCCTCCTCAGTGCCCACTGCACAACCCCAAGCAGAGGGCAGCCTCGCCAAGGCAACCACAGCCCCAGCCACCACCCGTAACACAGGTGAGAAGCCCCTTCCCTGCACACTCCACCCCCACCCACCTGCTCATTCCTCAGCCGCCTCCTCCAGGCAGCCCTTCATAACTCCTTGTCTGAGTCTCCAAGTCACACTTTGGTAAGGAGAGGGACACTGAACGGACCTCTAACAAACACCTACTGCCAGCCAGCCCCAGTCTGGGGGCCAGCAGATGCCAAACAACCAGCAGACTCCCAGAGCAGACCTGGGCCGGCTCCCTGGCCCATGGACCCAGCTCTGCCTCGCTGGGCATGGGCTCTCAGCGCAGCCTCACATAGAGCCACCCTGCCGAGGCAGTCCGGCTTGCAGACTCACAGGTCACTTGGGCCGCAGCAGCCCCTCCCCGTGACCCTCGCCTCCCGCCCGCCCCAGCCTGGCTCTCTCCAAGTGTTGGATCTTGGTGGCCAGCCTGCTTCTCACCCTCACCCTGCCTGCCACCTCAGAATGGCAGGGGAAAGAGGGCCCTCACCAAGAACTTTATCTGAGAAGTCTGAGGCTTGTGACTCTGACCTGCCTGAGATGTCCATGTGGCCGGGGGGACGGGTTCAGTGTTCGGGAGAACTCGGGTACGTGCCTGACTTTCTCTGAGTAGGGCAGGAAGCTGTTAGGAGAAGCAGCAGTGAGGTGGGCTGGACCAACAGGCAGAATGACTGTCCCTCAGCCACCCTCTGGGATGTGGGTCAAGCTCTGACAAAGGCATGGCACAGCCATGGTGGCCCCTGCTTGGATGAGTGGCCACGGTGCCCTCACCCTGGGCCAGAATCTGCCTCCACTCTGCAGGTGCAGAAACACGACATTCCCGTCTCTAAACACACCTAGCTCCTAGGCTTGGGGTGGGCCTATCAAATGCAGGGAGATGGACACAGCACAAGGGCCAGAGCTTCCCATGAGAAAGGTGAGGGCAGCTGCTCCCTGACCCGGGCATCTGCACTTGTCCCTCTCCACCCTCCTCATGGGCAGTGGAGACTCAGCAACAAAACAAGTTGAGTGCATTAGCAGCCAGCTCTGGAGCCAAGTCACTCACCCCACGGCCTTGGCTGCTGGTGGAGGGGCCTTCCCCTGGGCAGCCTCCAAGAAGACAGCCAAGTGCTCTTACTCAGACCACGGCGCTGCTTCCTGGCACCTCGATTTCCCACAACAACATGGGGTGCAGACAGGCTAGGGCCCCCTGCCCTGGGGCCTGGACGGCATCCAGTTAAAGATGACCCTTCACGGGCGGTGCCTGAGGTGTGCTGACCTCAGCAGCTAAGCCCTCAGGTCTGGTCTGCACTGCCCCACCTGGAGGACCCAACTGACCCAGACACAGCCAGGGTTATGGCATGACCCCGTGGACGGTGACCCACAGGCCAGATGCAGCCGGGGGCTGTTTTGTGTGGCCTAGAAATGTCTTTACAGTTGTAGTGGGATGGAGGAGGAAGAGGAAGAGAGGAGGGGAGAGGAAAGCAGGGAAGGGGAAAAAGAGGAGTTCAATGCAACCCCAAAAGCCAGAACAGTTTTGAGCTGAAAGAACAAGGCAGGAAACATCCCAGTACCTGACTTCAAAACATACTATAAAGCAGTTGTAATCAAAACAGGATCATAAAAACAGACACACAGACCCATGGAACAGAAAAGCGAGCCCAGAAATAAATCTACATGCTTGCAGTCCATTGATTTTCAACAAAGGCACCAGGAAAACACAATGGGGAGAGGACAGTTTCCTCAATAAATAGTGCTGGGGAAACTGGATATCCATGTGCAGACTAATGAAA

**Supplementary File 4D. GS10-RC22**

**Breakage-fusion sequence for GS10-RC22 (non-templated insertion)**

**22p12(+)** 5625232-CCTCCTCCGCTTCCCCCTCGACGGGGTTGGGGGGGAGAAG-5625271

**22q13.2(+)** 43478280-GATTTGGCCAGGCACAGTGGCTCACGCCTGTTATCCCAGGA-43478320

**Fusion sequence:** GATTTGGCCAGGCACAGTGGC**CACTGGC**ACGGGGTTGGGGGGGAGAAG

**ISCN:**

seq[T2T] r(22)(p12q13.2) g.(pter)_5625251del::ins[CACTGGC]::43478300_(qter)del, del(22)(q11.2) g.19265357_20705710del

**a.** **CNV analysis showing a 1.44 Mb interstitial deletion at 22q11.2, a 5.63 Mb deletion at 22pter-p12, and a 7.85 Mb distal deletion at 22q13.2-qter.**


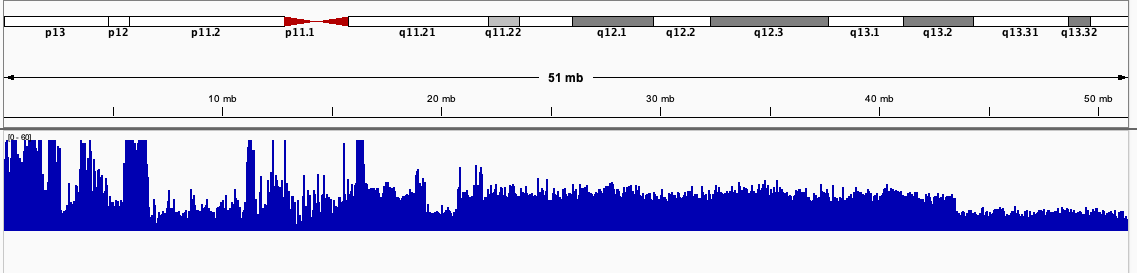


**b. A closer examination of the 22q13.2 region revealed the soft-clipped reads.**
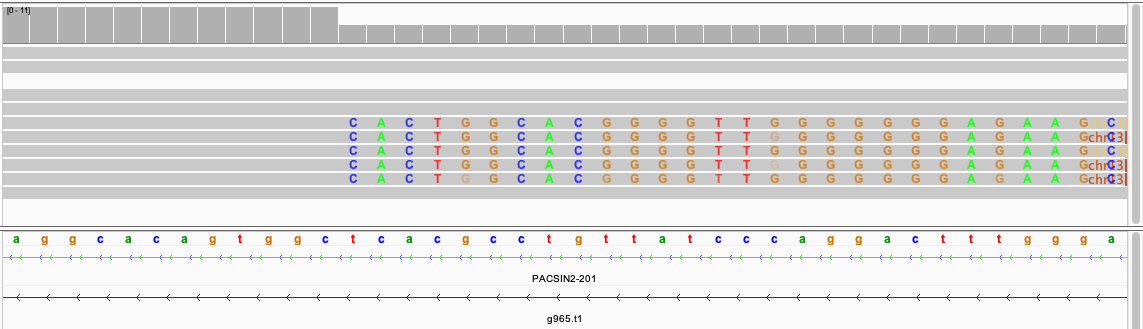


**c. BLAT search results to T2T**

The following 7.041 kb sequence is the soft-clipped region directly extracted from IGV.

Sequence 1-5361 (highlight blue) matches to chr22:43472941-43478300 (identity: 100%) at 22q13.2; sequence 6362-5773 matches to 16 segments within chr22:4799183-5625657 (identity: 100%), sequence 5774-7041 matches to two segments of chr22:5670960-5672220 and chr22:5716295-5717558 (identity: 99.4%). Consider the closeness of the matched sequence, assign chr22:5625252-5625657 (rDNA_22_1) and chr22:5670960-5672220 (rDNA_22_1) at 22p12 (highlighted yellow) for the latter two sequences. There is a 7 bp insertion ‘CACTGGC’ in the junction of 22p12 and 22q. Furthermore, there is a 1.44 Mb deletion at 22q11.21.

[chr22:43472941-43478300]

CATTTGTGACCTGGGATTAGGCAAAGATTTTAGCTGTGACACCAACCAAAAAAGCACAAGCCGTAAAAGAACAAATTGACTTCAGCAAATGTTTAAAACTTCTGTTCTTCAAAAGATACTGTTAAAAGAATGAAAAGACAGCCTCAGACAGGGAGTCAATATTTTCAAATTATGTAATTGATCAAGGAATTGTATCCAAAATATATTTTAAAAATTTCAAAACTAAGTTAATAAAACAACTCAGCTTTTTCAACAGGCAAGTGATTTAAAGAGATAATCACTAGGGAAATGCAAATTAAACCTACCAGAATGGCAAAAATTAAAAAGACTACATGTACCAAGTGTTGGTGAGGATGTGGGGGAACTAGAACCCAGTGCTGGTGATGCGCGCAAAGCACTTTGGAAAAGTTTGGCAGTGTCTGGACATGTTAAACGTACCCTAGCCACCAAACCTAGCCTAACCACCTAAACCACCAAAGCACCTAGCCATTCCACTCTAGGTATTTACCCCAGGGAAGTGGAAGCAGATGTTGGCCAAGTGCAGTGGTTCACACCTGTAATCCCAACACTTTAGAAGACTGAGGCAGGAGGATCATTTGAGCCCAGGAGTTCAAGATCAGTCTTGGCAACATAGGGAGACTCTGTCTCCATTAAATTTTTGATAAAAAGAAAGCAGATGTCCCCACAGAGACTGGCACACAAATGTTAATAGCACCTTATTTTTCTGTAACATCTTTTCACGACATAGCATCTTCATTTGTGATTACACCAAGTGGAAACAGCCCAAACGTCTGTCAATAGGTGAATGAACAAATTGTAGCATGCCCATGCATTGGATTTTTTAAAAAATGAAACTAACTGATAAATGCTACAACAATCTCAAAGTAATTTTACTGAGTGAAAGAAGCCAGACTAAAAATAACACATACTGTAAAAAGAATATATACTGGCCGGAAGTGGTGGCTCACACCTGTAATCCTAGCACTCTGGGAGGCTGAGGCGGGCAGATTGCCCGAGCTCAGGAGTTTGAGACCAGCCTGGGCAACACAGTGAAGCCCCGTCTCTACTAAAAATACAAAAAAATTGGCCGGGCATGGCAGCATGCACTTATAGTCCCAGCTACTCGGGAGCTGGGAGCTGGGAAGCAGGAGAATCACTTCAACCCGGGAGGCGGAGGTTGCAGTGAGTCGAGATTGCACCACTGCACTCCAGCCTAGCAACAGAACAAGACTCCATCTCAAAAAAAAAAAAGGAAGAAGAAGAATACATAGAATACATACTGTATGATTCCATTTACATAAAATTTTAGAAAATGCAAATGATTCTACAGTGGCAAAGAGTAGAGCAGTGGTGGCCTGGAAGAAGTGGGGGCAGAGGGACCCCAAGTGGCAAGAGGAACCCTCAGAGGGTGGACTTCTCACTATCTTCTTTGCAATGATGGCTTAACAGTGTATTCATATGCCAAAACTTATCGCCTGTTCATTATAACTCAATAAAACTGTTTCAAATTTGAAGGCAGAATAACATAAGACTGCTTACTTCTTATTTTGCCAAGTAAGAATATCCTTTAGAAACTACCAGCTCTTTTCATCAACATGGAAAGATAGGACATTTTAAAATGTAAAAGATACAATGGTCAAAATTTTATACAGAAAGAGCCCTTCCCAAGAAAGGGCAGCTGCTAACCTTACACACATGAAAACTGCATTGCCCTTATTTTTTCCACGTTGATGATAACACGATTGCAGACTGTCATCTTCTGAGACTGGCAGGAATGCCCCTGAAGGCCGACCAGAGCCGAAGGACACTCTCCGGGTTCATCTCCACATCCGACCTGGCAGTTCCTCCTGGAAGGGCGGCACCAGGGACAGCCATGTGCGCTGTGGGACCCAGACATCACTTCCCTGCCCTCCCACAAGTCAAGAAATCACAAGAAGAATGTGGCCCAAAAGGTTCCTGAAGCACTCCTATGCAGCTGAAGAAGGGAAGTGCCTTGTTTCCACAAGAGCTGCTCACCCCCAAGCCACAGTATCTGTGGTTTGCTGCTGGGACAAGTTCCATAAGTCAACATTAAAAACTCTTCAGTGGGATTCCTCCAGGCTAAAGTCCAGTGAGCTAAAGCCGAAATCAAATATGAGCTCTCAAGAAGACCACGATGTAGACTGGAATCCTTCTGATATTGCGCCAGAATGCTAACAAAAGCCAAGAGATGTCATCAGCAAAGCCCTAGAGGGAAACTTTGGGTAAAATGACAGTGCAGTGCGCTCCTGCTCAAGTGTGGCTGGTTTGGTGGTAAGAAGGGCTGAGCAGGGGGACAGGCCTGCTCTTAACCCCACCTCCCCATGCAACTGGGGCTCCAGGCATGGTCCCAAGCCCCATGTGTCAGATAGGGAGGGACAACCCCAAACACACCCAGAAAATAAGCATGAACTTTACCATTCAAGAAACCAGGCACACACATGAATCCAGAGCCGTGTCCTGCGCCACAAAGACCCTCGGGCGTGCTTATGGGGACACCCCAGCCTGCACACCCAAGCTCTGTCCACACATACCCTGCCCATGGCCACCATTCAGCCACCTTTGGCTGGAGGCACGTGCACACAGTGCACAGCCTACCCTCAGCAGGACACGCCAGGGAGGCGGCCCATGCAGGCCATGTAAGGGGTCTGGGGGTTACTGGGGCAGGGAATTCCAGGGTCTCTGTCCCTGTGCATGGCCTAAAGGGAGAGGCACAAGTTTTAGGGAGATACCACTTCTTGATACCTCAGATTCCTCACCCTATAGGGACAGTCACACTGGAAAAAGGGCCCCGGGCAGGGGTCCCACATGCCCCACCTACGGGAATGCTGACATATGAAAGGTCTGGGGGCTGCGGGGAGCACCCTAGCATATCCACACCTAGAGCCACAGCCTCGCAGCCAGCGAATGCAGCTGCCTCTTCCCTGGCCCAGAGATCCTTCTCTCTTGTCAGCAATCCCCATCCCACCCCAAGCCCCAGTCTCTGTCTCACTGGACTTTACACCAGGACTCCCGCAACTGTGCCTTGCCCGTTTAGACTGGAAATTCCATTTAGGCTGCCTCCTGGACTCCCTCTCAGGACCCTGCTTCCAACAATCACATAGTCAAAGGGGACAATGTCCCCCCTCCCCGCCTCGGGGCTGCTCCGGGCCCCAACTGCCAGGAAGCAAGACTGGACACTGCAACGTTTAACTCACATTGAGCTGAAGCAGCCGAGTTCTTCCTCTATAAAGTGAGCTGACTGCAGAGAAACTGCACCCTGGCATTCAGTCATGTTTCACAATAACTCCAGAGGAGGAGCTCTGCTTCTGACATGAAGTGTGGCAAGCCTTAGAAGGAAGCAGAGCTACAAAGTTATTCAATGCAAATATTTAAAGAACAGGCTGCTTTTGGTGCCAGGAGGACGGCAGCTTGCAAACCTCCAGTCACAATAAAGGTTCTGCTTGGTTGGGACTGAGTCAGGATGACAAATGTTGTTTCATTGCACCCTCCGTCCACTGAGGAGACAAGGTTTTTAAAGCAACTTTTCAGGAAGTTACTCTGTCCATTGGGCAGGACCTTAAAAGTCACAGCAAGAGAGCAAGTGTCTGGTGTGGAAAAACCAGAGTGTGTATACAACTACTCAGTCAAGAACTAAATGTGCATTTCCAATGTCATTCACGCGGCAATTATTTGGAAATAAGGATGGGATCATGCAATTTTGCCAACAGTAAATATACAGATTTGGTAACTTCAAGTGCCTATAATGTCCACCAGACAGAGGCCTTAAAGCACATTCCACCCATGTTTTCCTCCTTGAGAAAGGCAATTTGATTGTTAAAAGAAACAGTGGCCAGGCACAGTGACCCACACCTGTAATCCCAGCGCTCTGGGAAGCCGGGGACAGGAGGATCACTTGAAGCCAGGAGTTCAAGACCAGCCTGGGCAACACAGTGAGACCTGCCCCCTTCCCCAGTCTCCAGAAAAATTTAAAAATTAGCCAGGTATTGGCTGGGCGTGGTGGCTCACGTCTGTAATCCCAGCACTTTGGGAGGCCGAAGTGGGCGGATCACAAGGTCAGGAGATCAAGACCATCCTGGCTAACACGGTGAAACCTCATTTCTACTAAAAATACAAAAAATTAGCTGGGCGTGGTGGTGGGCGCCTGTAGTCCCAGCTACTTGGGAGGCTGAGGTAGGAGAATGGCTTGAACCTGGGAAGCGGAGCTTGCAATGAGCCGAGATTGCCACCACTGCACTCCAGGCTGGGCGACAGAGCGAGACTCCGTCTCAAAAAAAAAATAATAAAATTAGCCAGGTATGACACTGGACGCGGTGGCTCACGCCTGTAATCCTAGCACTTTGGGAGGCCAAGATGGGTGGATCACAAGGTCAGGAATTCAAGACCAGCCTGGCCAAGATGCTGAAACCCCATCACTACTAAAAATACAAAAATTAGCTGGGCATGGTGGCACACGCCCGTAATCCCAGCTACTGGGAAGGCTGAGGCAGGAGAATCGCCTGAACCCAGGCAGCAAAGGTTGCAGTGAGCCAAGATCGAGCCACTGCACTCCAGCCTGGGTGACAGAGCAAGACTCTGCCGCAAAAAAAAAAAAAAAAAAAAATTGGCCAGGTATGATGGTACACACTTGTAATTTGGGAGGGTGAGGTGAGAGGATCATTAAGCCCTGGAGTTGGAGGCTGCAGTGGGCCACAATCACACCACTACACTCCAGCCTGGGTGACAGAGTGAGGCCCTGTCTCAATCAGGGGGAAAAAAAGCAGAGAATAGAGACGGTCCAGGCCAAAATGGACACACTGAATTTTTTGTTGGTATTTCTGATGGTGTCCTCAGGCCTGGCTTACTGAGTTCTACAGCCTCAAGCTGCATGCTGATCAAAACCACACATCACATTTCCTAGGTATGAGGCCTAGGCCCAGCTTTCTCACTTGCAAAACAAAGTTGTCATGAGAATAAGTGTAATCACGCAAGGAAAAATAGGACACCTGGCACAAAATGAGCCTCAATAAACATTAAATTTTTTAAAAATAATTCTGATAACGATTGTGAAGTCAGAAACCAGTTCAGGGAAGTGAAGGCTAGAAGGGGGCTGGGAGGTTTGGCAACAAGCAGAGCCCTGGCAGGAGGGGATGAGGCTTGGACCCTCCCGCCCACTGAGGCTGGCCTTCCCTCCACACCAGGCTGGGGGTGTTTAATCACGCTCTGCCTCCTCTGACCAGAATGTAAGATCATGGACTACGTTGTTCCTGTTGTAACTCCAGTGCCTAGAACCGTGCCTAGCACATGGCAGGCGTTCAAAAATTTGCTGAATACATAAATGGGGATTTGGCCAGGCACAGTGGCCACTGGCACGGGGTTGGGGGGGAGAAGCGAGGGTTCCGCCGGCCACCGCGGTGGTGGCCGAGTGCGGCTCGTCGCCTACTGTGGCCCGCGCCTCCCCCCTTCCGAGTCGGGGGAGGATCCCGCCGGGCCGGGCCCGGCGTTCCCAGCGGGTTGGGACGCGGCGGCCGGCGGGCGGTGGGTGTGCGCGCCCGGCGCTCTGTCCGGCGCGTGACCCCCTCCGCCGCGAGTCGGCTCTCCGCCCGCTCCCGTGCCGAGTCGTGACCGGTGCCGACGACCGCGTTTGCGTGGCACGGGGTCGGGCCCGCCTGGCCCTGGGAAAGCGTCCCACGGTGGGGGCGCGCCGGTCTCCCGGAGCGGGACCGGGTCGGAGGATGGACGAGAATCACGAGCGACGGTGGTGCGGGCGTGTCGGGTTCGTGGCTGCGGTCGCTCCGGGGCCCCCGGTGGCGGGGCCCCGGGGCTCGCGAGGCGGTTCTCGGTGGGGGCCGAGGGCCGTCCGGCGTCCCAGGCGGGGCGCCGCGGGACCGCCCTCGTGTCTGTGGCGGTGGGATCCCGCGGCCGTGTTTTCCTGGTGGCCCGGCCGTGCCTGAGGTTTCTCCCCGAGCCGCCGCCTCTGCGGGCTCCCGGGTGCCCTTGCCCTCGCGGTCCCCGGCCCTCGCCCGCCCGTCTGTGCCCTCTTCCCCGCCCGCCGCCCGCCGATCCTCTTCTTCCCCCCGAGCGGCTCACCGGCTTCACGTCCGTTGGTGGCCCCGCCTGGGACCGAACCCGGCACCGCCTCGTGGGGCGCCGCCGCCGGCCACTGATCGGCCCGGCGTCCGCGTCCCCCGGCGCGCGCCTTGGGGACCGGGTCGGTGGCGCCCCGCGTGGGGCCCGGTGGGCTTCCCGGAGGGTTCCGGGGGTCGGCCTGCGGCGCGTGCGGGGGAGGAGACGGTTCCGGGGGACCGGCCGCGACTGCGGCGGCGGCGGTGGTGGGGGGAGCCGCGGGGATCGCCGAGGGCCGGTCGGCCGCCCCGGGTGCCGCGCGGTGCCGCCGGCGGCGGTGAGGCCCCGCGCGTGTGTCCCGGCCGCGGTCGGCCGCGCTCGAGGGGTCCCCGTGGCGTCCCCTTCCCCGCCGGCCGCCTTTCTCGCGCCTTCCCCGTCGCCCCGGCCTCGCCCGTGGTCTCTCGTCTTCTCCCGGCCCGCTCTTCCGAACCGGGTCGGCGCGTCCCCCGGGTGCGCCTCGCTTCCCGGGCCTGCCGCGGCCCTTCCCCGAGGCGTCCGTCCCGGGCGTCGGCGTCGGGGAGAGCCCGTCCTCCCCGCGTGGCGTCGCCCCGTTCGGCGCGCGCGTGCGCCCGAGCGCGGCCCGGTGGTCCCTCCCGGACAGGCGTTCGTGCGACGTGTGGCGTGGGTCGACCTCCGCCTTGCCGGTCGCTCGCCCTCTCCCCGGGTCGGGGGGTGGGGCCCGGGCCGGGGCCTCGGCCCCGGTCGCGGTCCCCCGTCCCGGGCGGGGGCGGGCGCGCCGGCCGGCCTCGGTCGGCCCTCCCTTGGCCGTCGTGTGGCGTGTGCCACCCCTGCGCCCGCGCCCGCCGGCGGGGCTCGGAGCCGGGCTTCGGCCGGGCCCCGGGCCCTCGACCGGACCGGTGCGCGGGCGCTGCGGCCGCACGGCGCGACTGTCCCCGGGCCGGGCACCGCGGTCCGCCT

**Supplementary File 4E. GS17-RC22**

**Pericentric inversion and breakage-fusion for GS17-RC22 (microhomology)**

**22p13:** 53000-GCTGGGATTACAGGCGCCCGCCACCATGCCTGGCTAATTTT-53040

**22q11.21:** 20358540-GCTGTGACAAGGGCCAGGGCTCTGGGTTCTGGTTACCCAGA-20358580

**Inversion inv(22)(p13q11.21)**  GCTGGGATTACAGGCGTTCTGGTTACCCAGA

**22q11.21(-)** reverse by inv: ACATCCTGTGCCCCA**GG**CTGAAGTTCAAGCGGGAGGCGGGC

20340760-GCCCGCCTCCCGCTTGAACTTCAG**CC**TGGGGCACAGGATGT-20340800

**22q13.32(+)** 50522360-CCAGGGCCTGATGGGACCCACCGG**GG**TGCCCAGAAAGTGTG-50522400

**Fusion sequence:** CCAGGGCCTGATGGGACCCACCGG**GG**CTGAAGTTCAAGCGGGAGGCGGGC

**ISCN:** seq[T2T] r(22)(q11.21q13.32)inv(22)(p13q11.21)

g.(pter)_53013del::[53014_20358565inv]::20340785::50522383_(qter)del

**a. CNV analysis showing 802 kb distal deletion of 22q13.32-qter.**
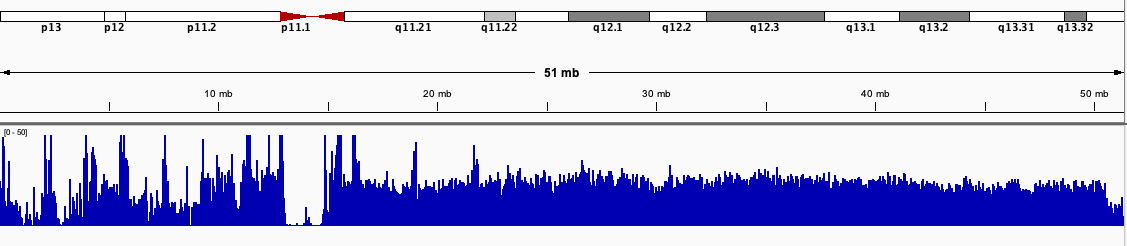


**b. IGV view of the junctions.**

**The 10.912 kb Chr22:20340783-right-clipped sequences match chr22:50511740-50522385(-)**


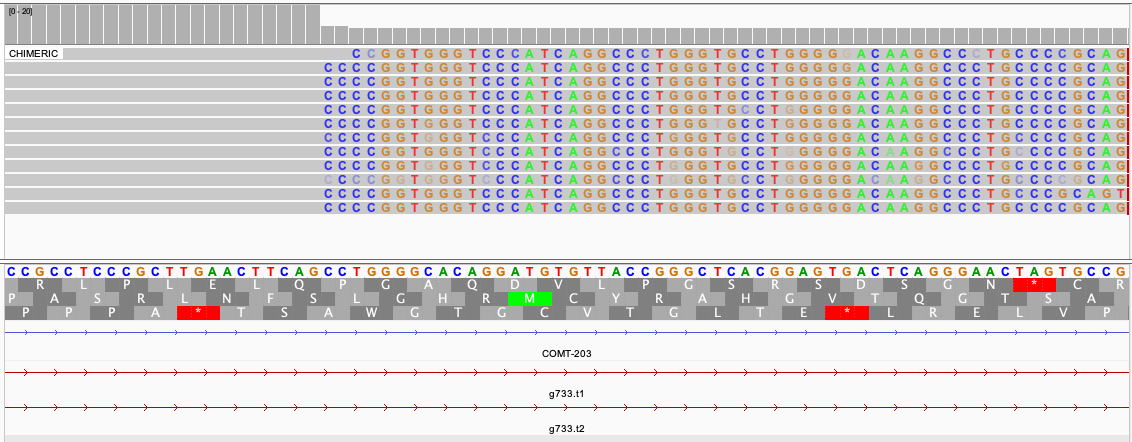


Chr22:20340783 CCCCGGTGGGTCCCATCAGGCCCTGGGTGCCTGGGGGACAAGGCCCTGCCCCGCAGTGCTACCTGGCCTGCTGCGCTGGGCCACCCTGGCAGATTTGACCTCCCAGTCCTGTGCCCGGCCTCCGTGCGTCTCTCCGCTGGGATGAGAGCCCTGGGGAGTGGGAGCTGTTCCCACCTCGTTCTTCCTGCTGCTCATGCCCTGCTTGTGCCACGTGGGCCCTGGCCTGTCCTATGGGCCAAGCTGGCCCAGCCCTTCCGAGGTTCCCGGAAGCCAAGGACTGCTTTGCATAGGCACTGGGGGCTCTCCCCACCCAGCCCCCCAGGAAGAGTGACTCAGCCTGGGGCTCGGAGTTTGCCGTGGGCGGTGGGTGCCCCAGCAGGGTGGTGCCCGCACTTCTGGGCTGGGCTGTGGCTGCTTGGGAACCTACAGCCCTGGCGGCTAGGAAAACCAGGGAGTCCCCTCTTCACCCCACATCCCTACCCCACCCTGGGGCTCCTTCTGTCCCTCCCCCACCAGCTATAGCTGCAGCAAGTGTGCAGGCTGGCCTTCCCCTAGTCCTTCCTGGCCTGGGCAACCTGAAGTTTGACTCACCAGCCCCAGTACATGGGGGACGGGGCTTGGCACAGGCTCACCCCACTGAGAAGGGCTGGCCAGGGACCCGGGACAGCCCCAGGGCAAGGACAGCGGTTGGAGGCCAGTGAGAGCCAGGCCAAGGCCCCAGCCTCTCGGATGAAAGGGACACATGGTGGTCACACCTGCCAGCCTTGCCTATCCTGTGACGCTCCGAGGAACTGCGTGAAAGTTCTCAAAACGCAGCAGCTCCCATGCAGGGGGTGGCCTTGCTGAGTGGCCAGCCCTGCGGTGGACGGCAGCTACCTGCTCTCACCCATCACGGCTGCCACCTTTGAGTCCATGCAGGAGAGGGCACGGTCTGCAGCCGCCTGCCACACCACGGGCACCACGTGGCCGCCTGCCAGACCACGGGCACCACGTGGGATGGTGGGCCTGGGGTGTGCTTCCTCCGGCAGCTCCTGGCATCAGAACTGCCCATGGGCCCTGGTGAGGTCTGACCACAGGTTGTGGGCAGCAGGTCTTCTAGCTCCACCAGGAGCCCCAGCCCAGGCCCACTTCGAAGGTGCCAGTGTGTCCAGATGGCCTGCACTGGGTGCAGCCTCTTGTGGGGACTCGGTGGCTCCTGGAGCACCCCCAGTCTTAACACTCCCACCTCCCCTCCTGACCCCTCCAGCTGCTGACCCATCCTGGGACTCTGTGGCTGGCCCAGCATGCTGCTGGCCCTCGCCTGCAGGCACCGTCCTGAACCCACCACCCACTTGGTCCTGGCCCAGTGGGGATGGGTCCTCAGCACCAGGCATCTGAACCTGAGCCCAGGCACAGCAGGGCAGGACGAGTTGGGGTGGGGGCTGCTCTTCTCCAAATCTCCTGGCCGGTTGGGTCCTTCTAGGCAGGACTCACCCGGAGGAGGGCTGAGAGAGGCTGACAAGCTAGAGGCAGAGCTGGACTGCCCCTCTGGGGTGGGGGGCCAGGTGGAGGGGCGTTAGGGAGGGTGAGGGGGGTGGGGAAGGGGACCTCCCATCCGCAGAGCCTTCATCCCAGAGGGACGACACCCTACCCCCGCCCCCCTGCGTTTGAGTGGAGGTCTTGGTTTTCCGAAAGCTGGGGGAAGCAGGTGGGCTCCATGCAGGAGCGAGTTGGGAGCCACCTGGGGCTGGTCTTGCAGGTGATAAGGGTGACCCTGGGGGGCCTGCCTGGGGGCGTGGCCTTGAGGGGCGCGGGCGTCGCCTGTTGGTGGGTGTGGATTGAGGGACGGGTGGGTGCGGTCCGGGGACCTGGGGTCGAGGTCAAGGGGCGCTAGGCACAGTCAGGGGGCCTGGGGCGCGGTCGGGGGTGGGCTGGGGTAGGAGAGGACAGGGGCGAGGTTTCAGGGATCTGTGGGCTCAGGGTCTGTGGACGCAGTCCAGGGTTCCCGGGGGGTGTGGGAAGGTCAGTGGGCTGGAGGAGCGGTGGCGGGGGGTGGGGTGGGCGGGGCCCGGGGACGCGACTGCAGGTGGAGGAAGGCGCTGTCGGGGGTGGGGAGTCCCGGGGCGCGCCGGGGCGGGCGAGTGCTGTCGGGGTGGAGGGAGGCGCGGCCGGGGAATGTGGGGCCGGCGCACGCGGTCTCAGCGCCCCTCCCTCCCCAGGGCTGCCGAGGGACCCCCGAGCGCCGCCCTGCCTCGTGTGCCGCGGGCTGCTGCCACCCTCCGGGCCCTGCAAGCGCTGCCGCTCGTTCTGCGCCGCGGTCCTGCAGGGCGCGTCCTTCGTGCGGCTCGGCGGGCGGAGCTGCAGCCCGCGGACCCCGTGAGTCGGGACCGGCGCGGGGACCAGGACGACGACCCGGAGCCACGCGCGTCTCCGTGGAAACCTGCGCGGAGCTGAGGCCGAGAACACAGCGCTGTTGTGGTGAACCCGCGTCCTCGCCGTGGTTTTCTTGGGAACCTCGTGCCCAGCACCCCCGCACCCGCTCCGGACCCTCCGAGCCGCTCTGGGGAAGGCTCCAGCCCGGGTCCCGGGCGGACGCTGGTTCGTTCCTTCCCGCGTTCGTCCGACGAGTCTCGGCGCCGCCCAGGGTCTCCGCACCCCGTTTCCTCCCATCTGGTGCCCAGCGCGGGCGCGGAGGGAGCCCCCGGGGTTGGACCCTCAGATCCCTTCACCGTTGGGCTTCTGGGCGACCGATGGTCACTGGGCTGTGTGGCCGTGCTGCCCTGGAGACCCAGACTTGGAAAGACCCCACCCCCACCCCAGGCCTCGCCCAGCTGCGGTGGGTGATCTCGGTGGCCGGGGCCCGCCCAGGAGCCCGTGGCCTGGCTCTTCATGGATACTGAGCAGGGCCAATCCCCCAGCCCTCAGAGACAGGGCTGGAGCCCTCTGACCTCAGGCTGGAGAGGGTGGCCGGGGGCCTGGGGCAGAGGGCAGAGGCCGGCCCCACTGTCCTTCCCTGGGCCTTGGAACCAGCACCAGCCACACCCCGTGGGCCGTCCCTACACGAGATCACCGACTACTGGCCACCTGCAGCCCCTGCCTGCGTCACGGCACGCTGGGATGGCGCCTTCAGGCAACGGGTGTGTCGTGGTGGGGGCCTGTCCCAGAGAGGCCTGGAGAGAGGTTTGGGTGTGAGTGGGCCCATGGGGGAGGGACATTTGTCCTTTCCCAATGATCCTGCTGGCCGTGCCCATGGACATCGTCTGGGACACCCCTGCCACAGTACAGCAGCCCCCATCGTCCCCTGGTATAAGGAGGGCTGGAGGCCAGGCTGAGGGGCGAGGCCCAGCTGGAGTATCTGTCTTACAGCTCAGCCAGCCTGGGATAGGGGATGACAGTGGCCCGTCCTGAGCCACCCCCAAATGTGCCTGCCTGCTGCTGACCATGCCGTGGCATGGAGGCTGGCACTAGCCACTCTCTCCAGGGCATTGTGGCCACAGGCACGCTGCTGCAGGGGGCGGGCTCAGCCCCATCTACTCTGCCCAGCAGTGAGTGCATAAGTTCCAGCCCATGGAGGGCTCTAGGCCAGGCGGGGGCAGGGTCGAGGTGCTGGCCCAGGAGGCAGGGAGGTGGGCAGTGCGGGGTGGTGGGAGGGAGGGAGGACTTCACGGTCACAGCAGGCCCCTGCCGCCTTCCCTCCCCTGGGACCGAACCCCAGAGCTTGTTTCTCAAGCTCCCTCCTGACTACCCGAGCCCACAGTGTGAGGCCCACAGCCTCTCCCAGCCTGGGGGTGGTGCCGCCCTCCACACTTGGGGTGTCCTGTGGGTATTCCAGGCAGAACACCCCAGCTAGACACTCCCAGGCTCCCCAGCTATTAACTCCCGCCATCCCCCCCCCCCACACACACAGACACACACACACACACACACACACACACACACACACACACACACACGTGTGGGGCAGTGCCCTCCCAGAACCTGAGGCAGGTGCAGAAGCCTCTGTGCCTGTCCAGGAAGGGAGGAGCCAGGAGGACCCAAGAACTGCACCCTGGTTCTCTCCAACCCCCTGCCCTGGCTGGAGGCAAATGGGAAGCAGTAAGCTTCTGCCCAGAAACTTTCCTATGCATTTGATGCTCCAAATAAGAAGATGACACAGAACAGCTCCCTGAGCAGAGGCTGGGCCCCAGAGTTCCCAGCCTCGGGGGGTGCCCTCCGGAGTGGGGGGGGGGGCTCATAGGGAGGACCGCACAGGGGAGGTGGCCTTTTGCATCTTGAGGGGAGGTGGAGGCCAGGGACTTGGGTGGAATCTCAGGTCTTAGGAGAGGGCTCCCCTTAGGGGCTGGGGCTGAAGCCAGGGGCTCTGGGGTATGAGTGCCCAGGACAGATTCCACCCCTCTGCCAGCCCAGCCAGCTTTCCAGGGCCCTTGTCTACCCTCCTGGCCTCATGCATGCCTGAGGGAGGTGCACGCCGGCGGCAGCCTATCCCGCCGGCCTGGAGCCATCTCTGCACCAGTGGCCGCCCTGTGGGGAACCCCATTAGGGTGTCCTCGGACCCCAAAGGCCCCAAGGTTTCTGGATGCTTCCTTTGGAAACCTAGAAACTCCACAAGTGTCTGGCCACAGTCTTCTGGCCCCAGGCGGTGGAAAGTCTCCGGGCTGCTGTCTGTGAAGCGAGCCCTGTTTTGCCGGGAGGGGGAGTGTCTCGCTCGCTCCAGCTGTGAAGAGCAGCCTCCACCCTCTGTTTGTCTTTGGGGCTGGAGCTGCAGGGTCAGCCTGGAGCCGCTGCCACAGGGCATCCTGCAAACTTGCCCCACTCACATCCACGCTTACCAGTGGGAGCTCTCACTGCACAGCCAGATGCTGGCTGGGCAAGCACTCGCGTTCTTGGGCCTGACCTGGGGCACTTTCCAGAGCCTGGCCATCCCCCGGATTACAGAATGTGGCCTCTCCTGTTCTCAGGTAAGCTCCTGCCTCCTCCTAGGCCCCTACACTGTGGCAGATAAGGCCCTGGTTTCTCCTCAGGTGGGTCTGGCCTGACGCTCTCAGCACATCAGCAGGTGAGGGGTTGCAGGGCCCTGGGGGTCCGAGGGCAGCGCTGGTGAAAGAAGGATGGGAGACCTGGCCTGACCCTGCCGAGCTGCTCCAGGAAGTGTTTGCTGGAAGCTCAAGGCAGGGTGAGCCCACCTCGGACCCACCTGTGACCTCCAAGGAGGCTGTGTTAGAATCTAGGGCAGCCCGCAAGCCCATCCGGGGCTCCCACCCCACCCCGCTCGGCTGCTCTGAGCCATCGGGAGCCCGCAACACCTGGGCTTGTGTAGACACCTGCCAAGGAAACACTCGGAGAACGTGCTGAGAATGAGATGTTGGCAGTGGAGGTGGCTGGGTTGGGGTGGAGGGCTCTCACTGCACAGCCCACTCACTCAGCCTTACAGGAGAGGGCGCTTCATCAGCACCTGCAGCAGGAAGTGTACCCAGCGTCCCAGGAAGGGGCCCCGGTGCCCAAGGCTGGCCGGGCACAGTGGGGACACAGGCATGGAAAGGGCACAGAAGGTGTCCCGGAAAGGGAGGACTCACTGAGGACCTGCTGGGTGCACCGTTCCTGGCATGGGGACAGCAGTTAGCAAGATGTGCCTTTAGGGTGGTGGCCTGGTCACCTCAGGGGACACTTGCGGGGACATCCAGCAGCTCCACAGGGCCTGAGACGGGTCCAGCAGAGGGCTCAGCTCAGGGAGGGGAGCAGCAAGGGCTGGAGTGCTGGGTGCCATGGGCAGGCTCAGATCCACAGGGCATTCGTGGAACAAGGAAGAAAGCAGGCTGGGATGCGTGGGAACCGGTGGCAGCAGGGGAGATGGGCGGGTTCAGGTGGGAGGGGTGTCCACCGGATGCCACCCTGCAGTGGAGATGGAAAACAGGGAGGGGGCAGGCCTGGGGATCCTGGCTAGGAGGGGGAAGGAGAAGCGAGAGATGAGACCTCAGAATCGACTTTTGCCTCTGGGAGGAAATGGTCCCTGAGTTGGGAGACATGGGGGGAGGCGGCCTTGAAGTGCAAGCTGGAAAGCTTTGCTGAGGCGGCTGCGGGGTATGCAGGCAGCCTCCCGGTCCGAGGTGTGACCGCGCTGTCCACGCTGGGACGGCCAGGTGATCCTGGGGAATGGGTGGGGACCAAGATGGAACCCTCAGTACTCCAAGGAAAGGGGCAGGGGCAAGGGCAGGGGCTGAGGCGGGTGGAAGAGGCCAGGAAGGAGGGGAAGTGCTGGAGGGGAGAGGAGAGGAGGGGTTTGGCGGGGCTGAGGGAGGGTCTCATGGAAGATGGGGTGGGGAGGGTTGAGCAGGAGGGGTCAAGGGGTGACAAGAGGAGCGGGAAGCTGGAGGTGGGTGTAGGGGCGCTGCCTGGGCAGCCTTGGACAGGGCGGTGGTTGCGAGGGGGCGCGGGAGCCCCTGCCCGCGGGGGGAAGCCAGGGAGGGGAGGCTGGGGCTACAGCCGCGCCCTGGGGGGGACCCGCGCGGGTGACGGCGGGAGGCTGGGCCGCATGCAGGGGAGCTTGGGCGGGGTGGGGCCGCGAGCAGCCGCCCTGGGGGTTCCCTGCACACCCCCCGACGCCGGCCGCTGGGAAGCCCCGAGAAGCACGACGGAAAACCCCGTGCGGGGGAGGCGCCCAGCGCCCGGGCTCAGCCCCCCCGGCCCCCGCTCCCCCCACCTTCCCCCCGTGGGGCAGGGGCGGGGCAGAACCGCGCGACGTTCCGCGCCCCCGACAGCGGCTTCTCCTGCAGCCCCAGACACTCGGGCCACGGGGCCAGCCCTGGGTGGGCTTCAGAGTCCAGGAAGTCAGGAGAGAGAGCAGACGCCCTTCGCCTCTCCAGCCCACCCCGGCCTCCGAGCCCCCAGCGGGGATTCTTTCTCTCCTGAAGGTGGATGCGGGGAGTCCAGGCCACAGTGGGCGCCCAGAGGGGCTTCAGGGCACCTGGCGGGAGGGAGGCCCGGGGACCCACGCCACTGCCCCCCTGCAGTTCCACACAGGCGGCCTCACTGGGCCTCCCCACGCTGCCTCCGCCGCTCCCTCTGTGCCCGGGCTGGTCTGGGCTGCCCTCGCCTGCCCACCTGAGGCCTGCTCGGATCCCGTTCCCCAGAGGCTGCCCCGGGTGAGCCCGGCCCCTTTTGCCTGGGCGGAGCCCTCAGCAGCCTGGGGTCAGCTCCGCCGGCCCCTCGTTTGAGGCCCTGCTCCCCACCAGGGGCCGAGGCCGGGGCTTCCATCAGGACGCTGGGTCCAGCCTGTATACTCCGCCCACGGGCGGCGGGTTTCGTGGCCATGGCAGGCGGAGGAGGGTCACTCACGGCAGTCTCTGGGGAGTTATTGGGGTGGTCGGCAAATGGGTAGAGACGGTGGGGTGAGAACGAAGACTCAGAGGCCATCTGCAGGGTGGGGTCCTGGGCTGGACCCTGGAACAGAAAACAGTTCACTAGTAGCACGTGATTCCACACCAGACTGAGGGTCACTGACTGCCGCCGATTCCACACCAGACTGAGGGTCACTGACTGCCGCCGATTCCACACCAGACTGAGGGTCACTGACTGCCGCCGATTCCACACCAGACTGAGGGTCACTGACTGCCGCCGATTCCACACCAGACTGAGGGTCACTGACTGCCGCCGATTCCACACCAGACTGAGGGTCACTGACTGCCGCCGATTCCACACCAGACTGAGGGTCACTGACTGCCGCCGATTCCACACCAGACTGAGGGTCACTGACTGCCGCCGATTCCACACCAGACTGAGGGTCACTGACTGCCGCCGATTCCACACCAGACTGAGGGTCACTGACTGCCGCCGATTCCACACCAGACTGAGGGTCACTGACTGCCGCCGATTCCACACCAGACTGAGGGTCACTGACTGCCGCCGATTCCACACCAGACTGAGGGTCACTGACTGCCGCCGATTCCACACCAGACTGAGGGTCACTGACTGCCGCCGATTCCACACCAGACTGAGGGTCACTGACTGCCGCCGATTCCACACCAGACTGAGGGTCACTGACTGCCGCCGATTCCACACCAGACTGAGGGTCACTGACTGCCGCCGATTCCACACCAGACTGAGGGTCACTGACTGCCGCCACCCATGTGGCTCCCCAGGTTTTGCAGATGCACGGGAAACTGTTGAGATGAGGGGATCCTGGGTAAACTCTCTGTAAATCTAAACATATTCCAAAATAAAAAATATTTAGAAGGAGGTGTAGCCACCGGTAATGCCCGCTCCACAATTCAAGGAACAGAAGTGGGCCCGACTTTTATTTTTCCGTGTTCCCACCTGGGCTAGGAGCAGAAGTCCAGGTCTCCATCTTGAGCTCCCCCCACCCCCACCCCCACCCCGACAGCCTGGGCTTCCCCGTCCGGTCTGTAGAGGGTCAGCTCTTTTCAAGAATGGAGCGGCTGTGTTGACCGCGGGCTGCACAGTCCAACACCCCAGGCTCAGTGGCCGCCCCCAGGGGGCCTGGACTCTGGGGGTCCTGCCCTGCTCTGAGGGCGCCAGTGCCACCCTGTGCTCTCGGACCTCAGCTCCCACGCCGTTGGTGCCGGGGTGCCGGGCGCCTTCTGCCTCACCCACAGGCTGTCTGCGAGCAGCCTGTCCCACAGGACCCTTCTGCCCCGTGGGAGCCTGGCCACAAGTCCTCATGCCCCTCTTTGAGTGCCCTTGGTCAGGTTGGGCTGGGGATGATCATAAATCAGGAGTGCAGCTTGGACCTGGACCATCCAGGACCTGTTCACTGCACGGCATGCCCCCGCCCCGCACCACCAACTCAGCCTCACTTGCTTCCAGGCCCATGAAATTGACCTCTATGAGGCCCCCCAAGCACCCCTTAGATTTATAACCTGCACCCCCCACACTATCCGGACCCCTCTTGCTCCTCCTGACCCCTCTGTTATTTTCCCACCCCTTTATTTTAGTTACACAGAATATAAGTTGCTGATTTCCCAGGTTTCGGATCGTTCTGTCTCCCCTTCTCGGAATGTGAGCTCCTGGAGAGGTGGGGATGGTTCAGTTTTCCTCTGTTCATGAATCCCAGCACCTAAGCGAGCCTGGCGCGTAGTGGGTGCTCTGCTAGTGTCCACTTAAACGCACAAGTGGGTCGGGGAGCTGGGGCCTGAGGGTCCAGAGGGATTCAGACCACACTGTTTACTAAGTGACCGCCGCATGCCAGCCTTGGGCCAGGCCCCTGACACTGAGGAGAAGCCAGGATTCAGGGCAGCCCAGGACCCCCAGGAGTGAGGGACAGGGCAGGAGCAGGATGGGCTGGGGAGGGGGTGCCCTCAGCCGCTCACCCCTCTCACTGTTCTGTCCCCAGGGCTTTGCCTGCAGGAGCCACAGGAACCGTGAGTACATCTCAGCCCCGGGGGATGCTCAGGGCCTGGACTCCCCACCCCAGGCTACCAGGGCCGCTTTGTCCACCATGGGAGGCAGGTCTGTCCTGCAGTGTCCCCAGCAGCTGGGGTTCCCTTCTGGGCAGGGCAAACCTCTATCACTCACCGATGCGTGTGGGACCCTGGCCACACCCAGGCAAGGCCACCCTGGCTCCTGGCCAGAGGTGGGCCTGCCAGAGAGTCCCACGTCTCCACTTAACGCACAATGCCTCGTTGCGTTCCAAACCGGCCGCCCAGAAGCAGCAGGTGGAAAACCCCGTCTCCTCTAGCAAAGCTTTTTCCCTGTGTAAAGTGATCCCTACTGGGCCTTTAACAACTACCTTGTCTTTGTTAAACAGCGTATCTATTTATAAAAGGACTCTGAGTGATGCAGACGCCACAAGGAAGAAAGTTAAGGCTCACTGGAAAGCCTCCATGTAGAAATAAACAGTGTTTACCTAGGCAAACATTGCTCTAGGCATTTTGGGTTTTTTTTTCTTATTTTTGATTTTCATAGGAATAGACCTGAAGTCTTCCTATCTCTGGTTGGTCAACCAAACCCAGGCTGTTCTCAAACTCCTGGGCTCAAGCGATCCTCCTGCCTCGGCCTCCCAAAGTGCTGGGATTGTGAACTTGAGCCACCATGCCTGGCCTGTTCTAGACATTTGTGTAGCCAAATATACAGATATTTGCAGGATAAACAGATAAAAATTATTTTATAGAAATGAAATTACCTTATGTCATTTAAACTATTTTGTTTATTTTTACTTGAATTTAACAGAAAAATAGATGGCTAAACTTTACTTAAATGTTTCTTCACCTCAAGGACCTCTAAGCTTAAGGGAGGAGATTAGATCAGCTTGGCCCCTGCGCAAGGATGACATGCAAATTCACAAAGCGGACTATAGTAACAAACAAACAACAAATGCAAAGCATGGCTGATGCGGTGGCTCACACCTGTAATCCCAGCACTTTGGGAGGCCGAGGCAGGTGGATCACCTGACGTCAGGAGTTCGAGACCAGCCTGGCCAACATGGTGAAACCTGTCTGTACTAAGAATCCCCCCCGCAAAAAAAATTAACTGAGTGTGGTGGCACTTGCCTGTAATCCCAGCTACCTGGAAGGCTGAGGCAAGAGAATCACTCGAACCCAGGAGGCAGAGGTTGTAGTGAGCCAAGATTGCACCATTGCACTCCAGCCTGGGCAATGGAGTGAGACTCTGCCTCATAAAAACATAAATAAACAAATAAAAGATAAAGCTCTTTAGTATTTCTAATAGATTTCCTATGGAGTCTCCTTTTTTAATGATGATCACATATTCTGCAGCTAATGACAGCTCGTAAACCTACGCATTGGGTGGAGGGGGCGGATCTTGTGGAATTGGCTCAGCTCTCCAGGGCAATGGTGAATGAGGGAATTCCTGTTTGAGTCCTGATATTAATGGTAATGATTCCAAAGGTTTACTGTTCAGTATTAAGTTTATTGGATGTTTGCACTAGATGTCCCTGATCTAGACAAAGAGGTGCCTTTCT

**This 7.255 kb Chr22:20358564-left-clipped sequences match best to chr22:53,015-60,265(-) (ct_22_1) at 22p13 (identity: 99.6%).**


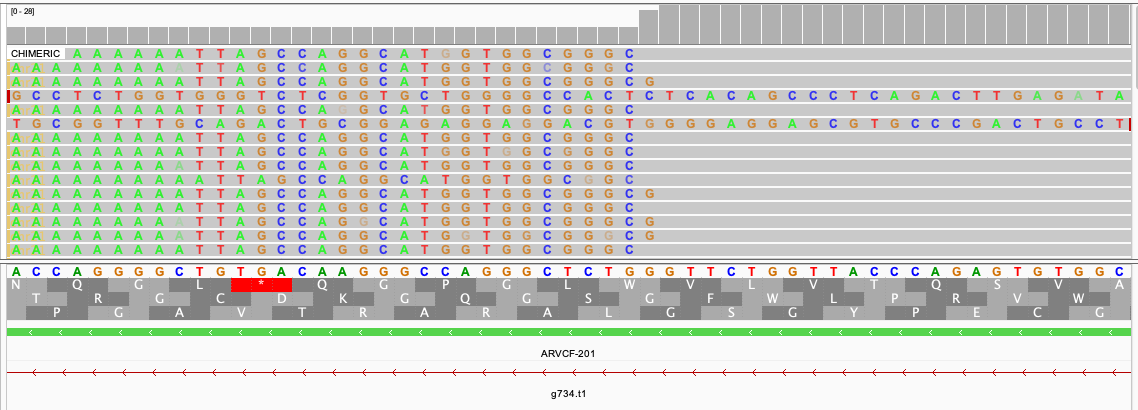


ACTGCATGTTCTCAGTTAGATGAGGAATCTAAAAATGTAAACTCATAGGAACAGAGAGTAGAATGGTGGTTGTTAGTGCCTGAGGGTGGGAATATGAGGAGATGCTGTTCAAAGAGTACAAATGATTAGTTGTAAGTTGAGTAAGTCTGAGAAAGCTAATGTACATTAGCATTAGATTAGCAAAATAGCATTGTCTTTATAGTAATACTGTAAAGTATGCTTACAATTTGCTACAACAGTAGACCTCAAGTGTTCTTGTGACAAAAAAAAAATGGTTATCTATGTGAGGTAATAAATGTGTTCATCAACTTGACATTTTGTTTCAAAATGTATATGTATATCAAATCATTACATTGTACAAATTAAATATAGTTATATAATTTTATCAGAAAAATCCGTATCACACAAACACAGATGTATGTTAAGTCCTCATTTAATGTTCAAATAGATTCTTGAAAACTGCATCTTTAAATGAAACAATGTTGTTACATGCCATTCAAACATAACTTGTTTTTATTTATTAAACTCTGGTAAAATTAGTTTTCTTAAACAATATGTTGCTTTACTTACAGTTTCCAAGAACCTATCAATGATGTTAAGCGAGAATTTCCTATACACATATATGACTCATATAGGTATGTGTATGTGTGTGTGACACACAGTTCTACATATAGATGGATTATAGCTCAATGACAGAATCCCAGGAGGCTTCCTACTAAATAAGACAAAATTATAAGATAATAGTTTATCAAGAAATAAGGTAACAATTGAGAGCTTAATATACACTTAGGAATGTTCTAAGCCGGTTAATGACATAGTGCATTTAAGAAATCTAGAAGGCGGGCCAGGCACGGTGGCTCAGGCCTGTAATTCTAGCACTTTGGAAGGCAGAGGTGGGCGGATCACTTGAGGTCAGGAGTTCAAAACCTGCCTGGCCAACATGGTGAAACCCCGTGGCGGGCACCTGTAATCCCAGCTACTCAGGAGGCTGAGGCAGAAGAACCATTTGAACCCGGGAGGTGGAGGCTGCAGGGCAGCAGAGGTTGCAGTGAGCCGAGGTCACGCCACTGCACTCCAGCCTTGGTGACAGAGCAAGACTCCGTCTCAAAAAAAAAAAAAAAAGAAAGAAAGAAAGAAATCTAGAAGGTGAGTAGATACTTTAAACCAGTGGTATCCAATCCTTTGAGTGTGAGGACGTTTTTGATTATCTGTGGTGATGGGTATCAAAAAAATTATTCACAAACCTATTTCTTGTTTCTTAGCTCATCGGCTATCATTACTGTTTGTGTATTTTATAATTTTAAGTGTGTCCCAAGACAAATCTTCTTCCAATGTGGCCCAGGGAAGCCAAGAGTTTGAAAACCTGTGCTGTAAACTATATTGTAATTTGAAAATTTGAATATATATAGAGTTCAAATTACAAATTTCAGTTTATACAAAATAACATTTTGAAGTAAAATAACATATTTTTTCATATTTGGAAAAACGCTTATTGTTCTGATAAAATTGCTGAGTAAGAGTTTTTTGTAGAATTACATTTGCAACCTCTACAGGATTAAAAATCACTAAGCAGGGTGATGCACACCAGCATGGCACATGTATATGTATGTCACTAACCTGCACATTGTGCACATGTACCCTAAAACTTAAAGTATAATTAAAAAAAAAAATCACTAAGCAGAAAAGATGCAACATCTGTCCTCGGTGTTCCTAAGATTTCTAAACCAAATCCCATTATGTCCACTACTCACCTTACGTATTTTAGGCATGATGTGAAAATAATATTTGAAAAAATACTTTAACATAGAGGTCCTCAATAATACAAATACTCCTATGTCACTAAAAGCAATGGAACAGCAGTCAGATTATGCAGCCCATTATAAGCGATAAAGTGGACATTGGCTCTCCTTGTAAACTTGGAGATCACTGAAGAAAAAAAGGAAATTCTTAGAAGATTTAAGAGCATGGGACAGAAGATGACCCTATTTGTGAATAAGAGAAAAAATGCAGCCTTTTCGGAAATTATTTTTATTGGCGCAGTTTCCCAGACTACATTTGAAGGCCTGGCTTCCTTCCTGACCTCTGGCCTCCTAGCCTGTGTCCTCTCCTTCATTCAGTCTCACCTACCTGGGGGTTTGGCTGCTGTCTCATGTATCTTCAAATTGCAGGGCTCTTTTATTTGCTCCAGACAGGTGACCAGGTCTGGGTTAGAGATCACAAAACCTGTTTTATTAAAAAAAAATTAACATGACTATTGCTGGGGATACTCCAATGACCAATCTATTACTATGCTAAGTAGAATAAAGAAATTATGGAAGACTCCAGAAAATTAATCCAAAAATTGTTTCCTGACAGAATCTTTAGAATACTGAAATATTTTAAATCTGTGGGTTCTAAGTTCCACTACCCAGTACTACTGAATCAAAAATAAGTGGTGCAAATTAGATTTTAGATGTGGAAAACAATATTTTACATCACTGAATTTCTAGAATTACTACTATCCTAGAGTGAAGGACACAAATTAGCTCAAGAAAAGAGATGGTTTATGCAATGATGAAACATCCTAAAGATTTTCTTTTTACACCAGCAAATCCCAATTTTTTTCTTGTAGAAAGGCACTGAAATTAATTAATGCAAAGCAGAAGCACCCAAGAGACATTCTGCAAAGAAAGAAAATGAAACTCTGAGGAAGTATTAGGAATTATGTATTGAAGTTATCCTCACCCAGGGAGACCAGGTTCCTGTAGTTCTCCAACATCACATCTCTATACAAATTCTGCTGGGCAGGGTCCAGACATTTCCACTCTTCAGGGGAGAATTCTATGGCCACATCCCTGAATGTTACGAGTTCCTGAAAACACATATTTATCATGTGACAGAGTTCTTAATTTGACTATAAGTGAAATCAGAGAACTAGTTGTGACTTATGGGGCTGACTGGAATTATCTGATAAAATAACTTTTAACACAGTAATGTTATCTAAAGTATTGTATAATTCTGAAGAAAAAGGAGGGCATGCCAGCAATTTTTGTTGCTGCAATGGAAATATGGGCTACACTGATCTGTCCCTACCAAAACCAAGCAAAGCAGGTCCTGTGACCTCCTTGAACAAAGGGTGAACTCACGTCTCATTAAAGTAACTGGAAGCCCTCATGCTTGACCCTGGCCTCACTGTAATGTCACACAAGAAACTTAACAGGATCTGTGGGGAGGGAACAGGTGACTGTTTCTCTTCAAACTGCCATGTGATCCTACTGGAAGACTGGGCTGAGAGTCACTTAGCTAAACATTGCCTCTCAAGCTTCAATGTGCACATAAATTTGGTATTCTAGGCCTCACTGTAAGTAACAAAATTCTGCAGGTTTGAAAAGGGTCCATTAATCAGCTTTTTATAGCCAAGTCTCCCATTAATGCTCATGCTCCTCCTAGACCCGTTATAGTACCACTCAGCTAGAGAAAGCAGACACAGCACGCAGAGTCCCTTATCCCAACACCCTTATCACAACACAAATACTTTTCATCCGAAGACAAGACCAGCAATCAACATCCTGAAACGTGGCATTATCTGCAAGCCCTTTAAAGGTTACAGAGGCTGGAGATGGTGGCAATGTCTGAGTAAGTCTGCATTTGAAAAACAGCACATCCACATGCATTAATGTGATATTTGTGGAGCATGTACTATGTGCTCAGGAGTATGTCACAGAGCACTGTGCTGGGAAGCTCACATTATGTGTGTTAATTCTCACAGCATCCTGGGAGGTGGGTACAAAGTGTGTGATACTTCCCAGGATTTAAATGCAGGGCCCAGCATTTCTATTTTTTCTTCTGTTTTCCTATCATTGATTTTTTAAAAAATATATAGAATAATAGCTCAATGTAGATAAATGGGAGAGACACAGAAGAAAGTTTTAAGTACAATTCGGGAATTTTTTATTTTTGTGTTTATAGTTACTTTGAGACTTGTGAAAAATCCACTGAAACTGCAAAGATGGAGAACAGGTTGCTGGATGGGATGTCTCTAGAAATACTGGTTTTAATTTTACATAAAATAATTTAAGTACATTAAATTCAAAGTACATTGCCTTTCTCCATTTATCAGCTTTTGTGTTTCAGGAAATTGGGAGCACCAGCTCTGGAGAGGCAGTAGGAATAGCTACTCCAAACTCTGATCTTCTCTAAGCAGTTTCTGTGAGGTATTTCAGTGTGGGGTCAGACCTGGACAAGGTTCAGTAGAGGGGGGATCTGGGCAAAGTTGGGACAGAAAATGGGCCCTAGGCTTCTGCTCTCTAGGCCACTAAGTAGTTTCAGTTTTGTCTTTTCTAAGCCTGCCCAAAAGAAATTTGATTCCCAGAGTTTGTGTAATTTTTATCTATTTTGCCACTTCCCCATCTATAACATACAATAACAAGGAATTTAACCAAAACCCTTTGTTTTTCCAGACCAATTATATTTGAAGTTAAATATTTATTCTTAGCAAGGCAAAAACAATACAAATAATGACTTCTCTTCTGTCCATAAACAGCCATGCAGGTGGTGGTATCAAACTCACAAAACAATAAAAGGGAAGTAACCCAAACGAAGCTTAAGTCTCCTGTACAGTTTTCATCTTTGTACTGAATACATGACGCTGAATTCAATCATTTATTAATGTGCTCTAGAATGCAAGTTCCTTGATGGTAGGGACCACGACTATTTCATCTCTTTTTCTAATGTCCATATGAAACACTGACCCAGTCTGCACAGGACATCCTCAAATGTCTCAAATACTCACTGATGCTGCTGAGAGTGTCCCCAGTGACCCTGGGCTGATGCCCCCATAGTGATCCAGGCAGGAGACTCAGGCTTTCTGGGGTGCAAACAGAAAATGGAACTGCCCTGATGGAGCTGCAGATCCTGGATCTGGATGTGATATCCCCTGTCCCTGATCAGCTAACTCTGAGGTAGGCGAAGGAAAAAAGTACTCTACTCCAGTAACACAGGGAGGCATAGTTGGCATTATGGCTCTGGATATTTAGTGGCCTTGACTTCCCACTGCTAAGGTGCTTGTTTACACTTACAGATTCTGCCACAGTATTGTGTTCCCACCAGAAGCCTCTTACTCAGCTGTAGCAGGTCACTGGACAAGATCTGGAAAACTCAAAGGGCTCCACTCTGAAAGTGGGGCTTATGTCTATGCTGACCTCTCATGATGCAGAAAACACCTTCTGTGACTTTTCTGTATCTCTTCAACCCAAAGTCTGGCCCTGTCTTGTGAATCCTGGGCAGAAGCCAGCTTTTATGTGCAGATTCTAGGTGAGATCAATGTGTGCTGCATTCTTGAGTTACAGGAAACAGAGTAAAATCAGAGGAAAGACCTTCTCATGAAGCCTCCTTCAACATATTCTAAAGAATATTTTGAGCTAAAGGGAAAAAGCTGGGGTAAACATAAGTAGAGAGTTTATTTGAGCCAAAGCTTGAAGACTGCAATCCTGGAGTATAAAGTTGCCCTGAATATACACTCCAATTAGAAGCAGTTACAAGCATATTTTTACAGGCAAAAAGGAAGTCAGAGAGGAGATTGATAGAAAGGTGTCAGAAATTCTTATTGGTTTATAGAAGTAACATTGGTTAGTGATTGGCCATATACATAGTTAAGCTATAGGGTATGGGTCATAGTGTCCAGTGTGGCATTATTAGGTTAATTTACAGCCACTTGTGGCAATAGCAAGCAATTTCAAAAGACAAATAGTTCAAGGAGGAGAACAGTATGTGAGTGTGGTCTCATTTTAACACATCTCTGGGTCTGATAAATCTAAAAACTTGTATTTCTCAAATAAAAGTTCTTTTATCAAATCTCAGGACATAAATTCAGAATTTGGAACTGCACATTTAGGTCTTGGAGGGCTGGTGAGCTATGTTTGTGGGCCACAATGTGGCCCATGCCAAATGTTTGTGGGCATGTGGGCAAGGGGAGGTGGGAGGGAGCAGAATTTCTCAGGTTTAATCGATGCGTATGTGTGAATCTGGTTAGGTTTATGGGCCCCATATCTCTGAAATCAGTGTCAGAACGGAAGATGCCAGGAGCACTGAAGGCGGTGAAATAACTGACTGCTGTCCTGAAAAGTTATTTTTGTAGAAATTTAACTGCTCTAGAAAGGACTGTAGATGCCAAGAGAGAGACCATTCTGTTTGCAGATTTAGGAAATACTTTGCTGCACTTTGCCACACAATTGCAGGTTGTGACTGGAATCCTGAGAAAGAATGTTTTCTCAAGTGAAGTCTGCTCGACATTTTGTGTATAACATCTGGTAATTCTGGACAGTGTGTGGAAAATATAACTAAAAGCAAAATCATCTGCAATCCTAAAAAAGTCTCCACAGTAACAGAACTGCAAGAAAATGTTTTGTTGTATAATTAAACCAAAATATGATGTGCATCACAAGCAATCTAAGAAAATGCAAACACAGAAAGTCACCATAATTAGTTCTCAAGTAGAAGCCTTGAAAGCACCATTTGTCATACACAATTTATTATAATTTCATCATGTAATTTGAGAGGGTCATCTGGGTTTTCTAATTTTTTCTAATCATTAAAGAAAAAATAAACATCCACATCTTCGTGACAGGATGTAGATTTGCAATGTGGAGCTAGGTACCTGCTGAAGGCAGCCTCCTAGTCCCCTACAGAAACTGTGAAATAGGGTGTATATTCTTGCTATTTACATTTCAAAGCAATAGTTCCCAGGTCCCAGATGAAGACAAATTTTGAGCCAAAAAAGACAAATGACCTATTTAACTGATAAAAATGATTTACATATATTTCAAAGAAGCAGAGAAAATATTTAAATATAAAAGTTCTCAAACTAAATGCTTTAAGAAAAAGGAGAAGAGCAAAAATTCTTCCCTCGTTCTAAAGAGAAAGCATTAAGCCTCTTCTAATTTGTGTTTGCTCCTACAACAGCCAGGCCAAAAGGCCCTGGTTTCGAACTCACTAACGTCTGAATTCTCATAGGCACCTGAGGGATGGACTTGGGCACACCGTGTACACAGAAAAGAGAATTTGTGGGGGAGGAAAAAGCAGAAGAGAAAGGAGCTATCAACAGCCATGGGTGGGAGGCCGGGCGCGGTGGCTCACGCTTGTAATCCCAGCACTTTGGCAGGCCGAGGCGGGAGGGCTCAAGGTCAGGAGATCCAGACCATCCTGGCTAACACGGTGAAACCCCGTCTCTACTAAAAATACAAAAAAAAAAAAAAATTAGCCAGGCATGGTGGCGGGC-**chr22:20358564**

**This 3.046 kb Chr22:50522385 right-clipped sequences blat to chr22:20337716-20340783 (identity 99.8%)**


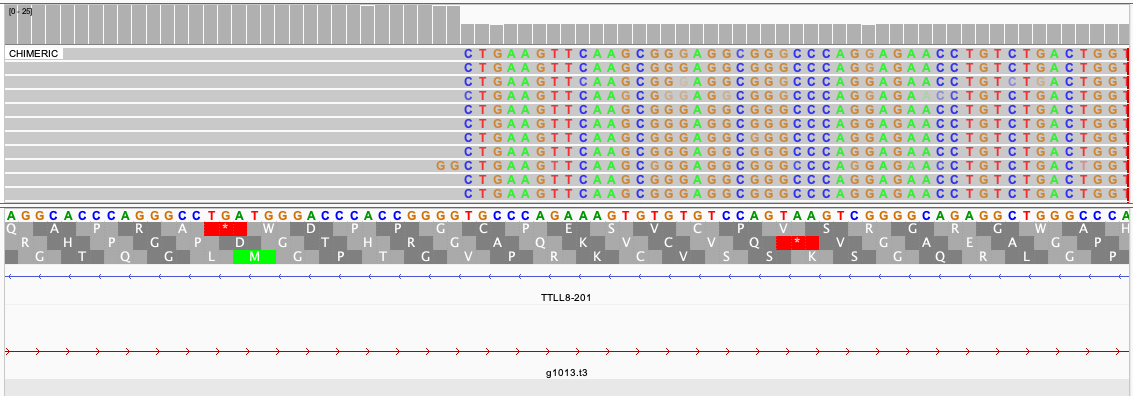


CTGAAGTTCAAGCGGGAGGCGGGCCCAGGAGAACCTGTCTGACTGGTCCCCAGGCAGGTCCCATGGTGGCCAGAACAGAGTTCAAGGGGAACTGCACCACCGTCAGACCCTGTGAGTGTGGGGGCCTGTTGTCCTGGGGCAGCAGGCAGCAGGGCCTGCAGGGGTCATCAGGGACCCTGGCACCCTGGGACTCCAGAGGAGCCCCCCAGCCCTATCTGGGCATATCCCAGGGTTCCCAGCCCCCTGGAAATGGGGTGATAACAGCTTCTCCTGTAAGGGCTTTGATGCCTGGTCCTGGGTCCCTGTCCCAGAGCTGAGCACCTGCTGGCCCGGACCCCACCTTTCTTGTCGCCCACGTTCATGGCCCACTCCTTCTGCTCGCAGTAGGTGTCAATGGCCTCCAGCACGCTCTGTGCGTTCCCGGGCTCCGCATGCTGCAGCACATGGTTCAGGATGCGCTGCTCCTTGGTGTCACCCATGAGCAGGTTGTGGATGGGCTGCAGGATGAACTCGTTCCAGCCGATAAGGCACAGGCCCCAGCCCCAGTGCCTCAGAAGCAGCAGCAGCACCACCAGCAGCACCAGGCCCAGCAACACAGCTGCCAACAGCAGAGGCGGGGCCTCCGGCATCTGCAGGGCGGGAGGGGAGGGGCGCCAGTGCTCTGTGCTCCTCCTGCAGCACCCACACGCCCCTTTGCTTGGAGTGCCACCATCGCCCCCTTGTGTTTGCAGAGGGGCAAGGTTCAGAAATGCCAGCCTTGTGCCTCAGTTGCCTGACGTGTCAGCGCCAGGGAGCCAGCCTTCTCGTGCCCTCTGGTGCCCTCGGGTAGACAGAGCAGGTGTGTGCCTCTGGCAGTCCTGTGTGGAGCCTGCAGGGTCACACTCCCTGGCCCTGGGTGCTTCCCCAGGCAGCCCAGGGATCCTCTAAAACCACACAAAATACAGAAGCAGACACGGACTCAGGCTGTTGTCTCCCAGCTGGCTTAGAGGACACCCAGGGGTAGGGAAGCCCCATGGCCCGGAGTTGGGGGTAGTCACGGGTGGCCAAAGGAGAGGCTGTATGGTGCAGCCCCTTGGCCTCATGTGGTGAGCCCGTGCTGGTCACCCATGCTCTGGACGCCCCAGGGATTCATGTGGCCTTTTCATTGAGTGTCCAAGTAAGGACACTGGCAGTCCCCAGGCCAGGCACACCAGGACAGACCCACTCACCACTCCCCTTGGGAACAGCAACGTGGCATGTCCCAGATGAGAAAGGCCAGCCCTGCCGCCCACAACAGTGTACCTCGTGGGGCACAGTGATGGCAAGTGACCCCACTTGAAACCTGTTTGAAATCCTCCTGGTGTGACCCTCCAGGCAGGTAGCCTGGGAACCAGACAGAAGCCTGGTGAGGGGACCCAGCAGGCAGAGAGCAGAGCACAGGAGGCCTGAGGCAGAAGCCCAGGGGTTCAGGTCACAGACCCTAAAACGGGAAGGACACAAGGAAACTTCTTCTAGGGCCCTGTGCTCACTCAGAGGTCTTTCTGGGATGGGGCCCTGGGCTCCCAGTGGCATAGGAGATCCCACTCTCTGCTGGACGCTCCATTTTAGAAGGCACACCCAGGCAAGGAGGCAGGCCTAGGGCCACAGAGGGGACACCCCTGGAGGCCAGCCTCAGGGCACCAGAAGACAGAGCAGAAGAGAACAGGGAAGCAGAGGCTCCGCCCTAGGGGGAGGTCATGCGGACACCAGGCTTCTAGGGGATTTCAGGCCATGTGGCCTATTTACACAGGCAGAGTGGACATGTGCTCAGCAGACCTGTGCCCATGCACCGGGCGGGAATCTGTACCCACCCTGGCACTGCCCCGGCTTCCGTTGGCCAACTCACCTTCAAAGCACCTCTGAGCAGGCTCCAGCCCTCTGGCTGCGGGAGGGGTCTGGGGTCTCCTCTGAGCTCGGCAGCAAAGCAGATGTTATTTCTGTGGAAATCAATTTTGCCTTTGGATAATCATCAACATAAACCCTAACCTGGCGATGACCAAGGTCCAGATACCTGAGCCCCTTCCCCGGATAGGGCCAGCTGGCCTGAGCCTTCCCTAGCAGGGCGAGGGCAGTGCTTCGCCTTTCCGGCCTCAGAAGAGACAGCAGAAGGCCTCAGAGAGGCCAAGGGACATCCCTACTAGTGTCCCCCAGCCAGGGACCCCAGGGGCAGTCTCCTGGTCCCTGTCCACACTGCCCTGTGGGCTATGTGTGTGTGCACCTGCACCTTGTGAACCACAAGAGGAGGGAACCACTCTGCTCAAGTTGTATTTCCCCTGAAGAGTCCTTAGTACCTTGGAAATGATGTGTAAGACCAAGGGCTAGTTTACCAAGAACATACACAAACCAGTAAGAAAAAGATGATGAGACAGATAGGTCACAGTGGCCAACATGCAGCTCACAGGAAAAGGCCAAAGGCCAGAGCCCCGAGCAGGTGATGGAACCCGGTGCAGGGCTCAGTGACAGTGGGGGTGCCCCTGTCCACAGCTGCAGGAGTGGGAATCGTGCTGCCTTCTGCTGCTACCCTTGGGAAGGGTCCCAGGCCTGCAGGGAAACCCGTGGCCCACTGTGTTTCCCAGCGGGGCAGGACGTGCCATGACCCACAGGGCTCGTGGGGAAAAAACAAGAGATGGTGCTTCCCTAAGGACAGTGTCCTTATTCTGGCAGGAAGATGACAGGGGGAACCATGGTGGAGAGGGACCATGCAGACCTGAAGCAGCCAGAACTTCCACCTTGCTCCTTTCTTCATGGTGGGGATTTCCTATGACACAGCCTGTTGCTTTGTTTACTATTATTTATCTGCTATGGTTTATCTATAATGACTACTAGTATTTGTGTGGTCAGATAATGGGCCATTTTGTTTACTCTCCATATTTAAAAAACAGTGTTAGAAATATATACCCCAAATTAAAAGGGCTATTCTGAAACAGCAAATTACTTGGTAGACCTTGGAGTCCCAAATTATAAATCCAAATCTGCGTTAATACTTGAATTGTGTAAGAATTTGGCTTTTGAATCTCTAATCTCTAGGTCTGTCTGTTCATAAATC

**Supplementary File 4F. GS12-RC13**

**Complex rearrangement for GS12-RC13 (two-break five-fusion with fork-stall and template-switch, FoSTeS)**, **BLAT search results for breakage-fusion sequences:**

**Fusion sequence (f1):** chr13(+): 15562601::[chr1:890898_890424ins]::41706593

**Fusion sequence (f2**): chr13(-): 70420369::[ins10bp]::103443256

**Fusion sequence (f3, TeS):** chr13(+): 60933721::70420072

**Fusion sequence (f4, TeS):** chr13(-/+): 63326518::60956427

**Fusion sequence (f5, TeS):** chr13(+/-): 70269448:: 60857975

ISCN: seq[T2T] r(13)(p11q33.2) g.(pter)_1556260del::[chr1:890898_890424ins]::41706593_[60933721::70420072tes_ 63326518::60956427tes_ 70269448::60857975tes]_70420369 ::103443256_(qter)del

**1p36.33 (-)**  AGCACATCCGAGTCCCACGACCACACCCGTCGTCGCGGAGGGTAGAAGGTCC

**13q14.11 (+)** 41706561-GTTGGACGTTTGAGTTGATTTTCATTTTTTGTCACCCTGAACAATCTTGTAATGA-41606615

**1p insertion at f1:** GAAGATGGGAGGCGCTGCTGCCCACACCAGCACCCTGAACAATCTTGTAATGA

**13q21.33 (+)** 70420336**-**AAAGATCCAGCAGTAGGAACTTGATAGGATTTGAGAAGTGCTAC-70420379

**13q33.1 (-)** AGTTCATGGGGAATTACTAGTCAGTCTCTTTCTCCATATT

**Distal fusion at f2:** AAAGATCCAGCAGTAGGAACTTGATAGGATTTGATCAGTAAATT

Sequence at 13q14.11 belongs to L1ME4a (LINE/L1), and sequence at 13q21.22 belongs to MLT1G1 (LTR/ERVL-MalR)


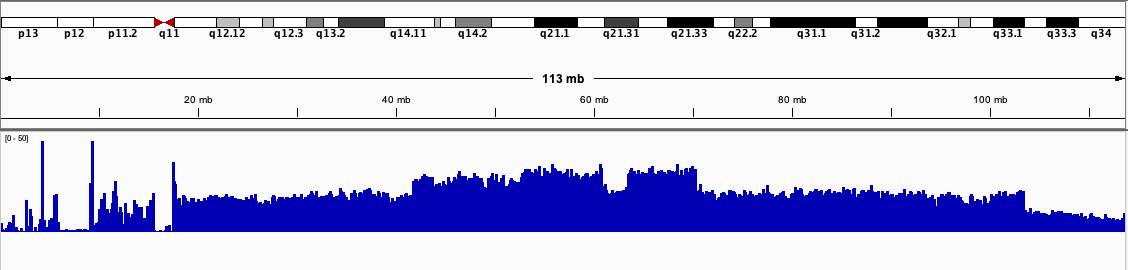


1. **Chr13:41706593 left-clipped sequences**


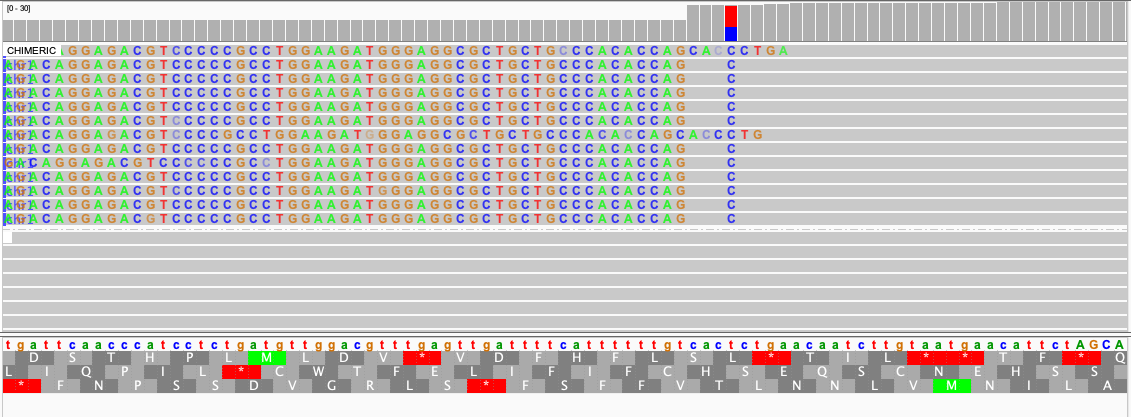


TAAGCGGGATTCAAATAAAAGGTAGACAGCAGCATTCTCAGAAATTTCTTTCTGATCTCTGCATTCAACTCATAGAGTTGAACATTCCCCTTTCATAGGGCAGGGTTTGAAATACTCTTTCTGTAGTATCTGGATGTGGACATTTGGAGCGCTTTGATGCCTACGGTGAAAAAGTAAATATCTTCCCATAAAAACGAGACAGAAGGATTCTGAGAAACAAGTTTGTGATGTGTGTACTCAGCTAACAGAGTGGAACCTCTCTTTTGATGCAGCAGTTGGAAACACTCTTTTTGTAGAAACTGTAAGTGGATATTTGGATAGCTCTAATGATTTCGTTGGAAAAGGGAATATCATCATCTAAAATCTAGACAGAAGCCCTCTCAGAAACTACTTTGTGATATCTGCATTCAAGTCACAGAGTTGAACATTCGCTTTCTTAGAGCACGTTTGAAACACTCTTTTTGTAGTGTCTGGAAGTGGACATTTGGAGCGCTTTGATGCCTTTGGTGAAAAAGGGAATGTCTTCCCATAAAAACTAGACAGAAGCATTCTCAGAAACTTGTTTGTGATGTGTGTACCCAGCTAAAGGAGTTGAACATTTCCATTGATAGAGCAGTTTTGAAACACTCTTTTTTGTGGAAAATGCAAGTGGATATTTGGATAGCTTGGAGGATTTCGTTGGAAGCGGGAATTCAAATAAAAGGTAGACAGGAGCATTCTCAGAAATTTCTTTCTGATGTCTGCATTCAACTCATAGAGTTGAAGATTCCTTCATAGAGCAGGTTTGAAACACTCTTTCTGGAGTATCTGGATGTGGACATTTGGAGCGCTTTGATGCCTACGGTGAAAAAGTAGATATCTTCCCATAAAAACGAGACAGAAGGATTCTGAGAGACAAGTTTGTGATGTGTGTACTCAGCTAACAGAGTGGAACCTTTCTTTTTACAGAGCAGCTTTGAAACTCTATTTTTGTGGATTCTGCAAATGGATATTTAGATTGCTTTAATGATATCGTTGGAAAAGGGAATATCGTCATACAAAATCTGGACAGAAGCATTCTCACAAACTTCTTTGTGATGTGTGTCCTCAACTAACAGAGTTGAACCTTTCTTTTTGATGCAGCAGTTTGGAAACACTCTTTTTGTAGAAACTGTAAGTGGATATTTGGATAGCTCTAACGATTTCGCTGGAAACGGGAATATCGTCATCTAAAATCTAGACAGAAGCACTATTAGAAACTACTTGGTGATATCTGCATTCAAGTCAAAGAGTTGAACATTCCCTTACTTTGAGCACGTTTGAAACACTCTTTTGGAAGAATCTGGAAGTGGACATTTGGAGCGCTTTGATGCCTTTGGTGAAAAGGAAACGTCTTCCAATAAAAGCCAGACAGAAGCATTCTCAGAAACTTGTTTGTGATGTGTGTACTCAACTAAAAGAGTTGAACCTTTCTATTGATAGAGCAGTTTTGAAACACTCTTTTTGTGGATTCTGCAAGTGGATATTTGGATTGCTTTGAGGATTTCGTTGGAAGCGGGAATTCGTATAACAACTAGACAGCAGCATTCCCCAGAAATTTCTTTCGGATATTTCCATTCAACTCATAGAGATGAACATGGCCTTTCATAGAGCAGGTTTGAAACACTCTTTTTGTAGTTTGTGGAAGTGGACATTTCGATCCCCTTGACGCCTACGGTGAAAAAGGAAGTATCTTCCCATAAAAAATAGACAGAAGCATTCTCAGAAACTTGTTGGTGATATGTGTCCTCAACTAACAGAGTTGAACTTTGCCATTGATAGAGAGCAGTTTTGAAACACTCTTTTTGTGGAATCTGCAAGTGGATATTTGGATAGCTTGGAGATTTCGTTGGAAGCGGGAATTCAAATAAAAGGTAGACAGCAGCATTCTCAGAAATTTCTTTCTGATGTCTGCATTCAACTCATAGAGTTGAAGATTCCTTTCATAGAGCAGGTTTGAAACACTCTTTGTGGAGTATCTGGATGTGACATTTGGAGCGCTTTGATGCCTACGGTGAAAAAGTAAATATCTTCCCATAAAAACGAGACAGAAGGATGCTGAGAAACAAGTTTGTGATGTGTGTACTCAGCTAACAGAGTGGAACCTCTCTTTTGATGCAGCAGTTTGGAAACACTCTTTTTGTAGAAACTGTAAGTGGATATTTGGATAGCTCTAATGATTTCTTTGGAAACGGGAATATCATCATCTAAAATCTAGACAGAAGCACTATTAGAAACTACTTTGTGATATCTGCATTCAAGTCACAGAGTTGAACATTCGCTTTCTTAGAGCACGTTGGAAACACTCTTTTTGTAGTGTCTGGAAGTGGACATTTGGAGCGCTTTGATGCCTTTGGTGAAAAAGGGAATGTCTTCCGATAAAAACTAGACAGAAGCATTCTCAGAAACTTGTTTGTGATGTGTGTACCCAGCTAAAGGAGTTGAACATTTCTATTGATAGAGCAGTTTTGAAACACTCTTTTTGTGGAAAATGCAAGTGGATATTTGGATAGCTTGGAGGATTTCGTTGGAAGCGGGAATTCAAATAAAAGGTAGACAGCAGCATTCTCAGAAATTTCTTTCTGATGTCTGCATTCAACTCATAGAGTTGAAGATTCCCTTTCATAGAGCAGGTTTGAAACACTCTTTCTGGAGTATCTGGATGTGGACATTTGGAGCGCTTTGATGCCTACGGTGAAAAAGTAAATATCTTCCCATAAAAACGAGACAGAAGGATTCTCAGAAACAAGTTTGTGATGTGTGTACTCAGCTAACAGAGTGGAACCTTTCTTTTTACAGAGCAGCTTTGAAACTCTATTTTTGTGGATTCTGCAAATGGATATTTAGATTGCTTTAACGATATCGTTGGAAAAGGGAATATCGTCATACAAAATCTGGACAGAAGCATTCTCACAAACTTCTTTGTGATGTGTGTCCTCAACTAACAGAGTTGAACCTTTCTTTTGATGCAGCAGTTTGGAAACACCCTTTGGTAGAAACTGTAAGTGGATATTTTGATAGCTCTAACGATTTCGTTGGAAACGGGAATAATCATCATCTAAAATCTAGACAGAAGCACTATTAGAAACTACTTGGTGATATCTGCATTCAAGTCACAGAGTTGAACATTCCTTACTTTGAGCACGTTTGAAACACTCTTTTGGAAGAATCTGGAAGTGGACATTTGTAGCGCTTTGATGATGCCTTTGGTGAAAAGAAAACGTCTTCCAATAAAAGCCAGACAGAAGCATTCTCAGAAACTTGTTTGTGATGTGTGTACTCAACTAAAAGAGTTGAACCTTTCTATTGATAGAGCAGTTTTGAAACACTCTTTTTGTGGATTCTGCAAGTGGATATTTGGATTGCTTTGAGGATTTCGTTGGAAGCGGGAATTCGTATAACAACTAGACAGCAGCATTCCCAGAAATTTCTTTCGGATATTTCCATTCAACTCATAGAGATGAACATGGCCTTTCATAGAGCAGGTTTGAAACACTCTTTTTGTAGTTTGTGGAAGTGGACATTTCGATCGCCTTGACGCCTACGGTGAAAAAGGAAATATCTTCCCATAAAAAATAGACAGAAGCATTCTCAGAAACTTGTTGGTGATATGTGTCCTCAACTAACAGAGTTGAACTTTGCCATTGATAGAGAGCAGTTTTGAAACACTCTTTTTCCTGAATCTGCAAGTGGATATTTGGATAGTTTGGAGGATTTCGTTGGAAGCGGGAATTCAAATAAAAGGTAGACAGCAGCATTCTCAGAAATTTCTTTCTGATCTCTGCATTCAACTCATAGAGTTGAACATTCCCTTTCATAGGGCAGGTTTGAAATACTCTTTCTGTAGTATCTGGATGTGGACATTTGGAGCGCTTTGATGCCTATGGTGAAAAAGTAAATATCTTCCCATAAAAACGAGACAGAAGGATTCTGAGAAACAAGTTTGTGATGTGTGTACTCAGCTAACAGAGTGGAACCTCTCTTTTGATGCAGCAGTTTGGAAACACTCTTTTTGTAGAAACTGTAAGTGGATATTTGGATAGCTCTAATGATTCGTTGGAAACGGGAATATCATCATCTAAAATCTAGACAGAAGCCCTCTCAGAAACTACTTTGTGATATCTGCATTCAAGTCACAGAGTTGAACATTCGCTTTCTTAGAGCACGTTGGAAACACTCTTTTTGTAGTGTCTGGAAGTGGACATTTGGAGCGCTTTGATGCCTTTGGTGAAAAAGGGAATGTCTTCCCATAAAAAACTAGACAGAAGCATTCTCAGAAACTTGTTTGTGATGTGTGCACCCAGCTAAAGGAGTTGAACATTTCTATTGATAGAGCAGTTTTCAAACACTCTTTTTGTGGAAAATGCAAGTGGATATTTGGATAGCTTGGAGGATTTCGTTGGAAGCGGGAGTTCAAATAAAAGGTAGACAGCAGCATTCTCAGAAATTTCTTTCTGATGTCTGCATTCAACTCATAGAGTTGAAGATTCCCTTTCATAGAGCAGGTTTGAAACACTCTTTCTGGAGTATCTGAATGTGGACATTTGGAGCGCTTTGATGCCTACGGTGAAAAAGTAAATATCTTCCCATAAAAACGAGACAGAAGGATTCTCAGAAACAAGTTTGTGATGTGTGTACTCAGCTAACAGAGTGGAACCTGTCTTTTGATGCAGCAGTTTGGAAACACTCTTTTTGTAGAAACTGTAAGTGGATATTTGGATAGCTCTAATGATTTCGTTGGAAACGGGAATATCATCATCTAAAATCTAGAGAGAAGCCCTCTCAGAAACTACTTTGTGATATCTGCATTCAAGTCACAGAGTTGAACATTCGCTTTCTTAGAGCACGTTTGAAACACTCTTTTTGTAGTGTCTGGAAGTGGACATTTGGAGCGCTTTGATGCCTTTGGTGAAAAAGGGAATGTCTTCCCATAAAAACTAGACAGAAGCATTCTCAGAAACTTGTTTGTGATGTGTGTACCCAGCCAAAGGAGTTGAACATTTCTATTGATAGAGCAGTTTTGAAACACTCTTTTTGTGGAAAATGCAAGTGTATATTTGGATAGCTTGGAGGATTTCGTTGGAAGTGGAAATTCAAATAAAAGGTAGACAGCAGCATTCTCAGAAAATTTCTTTCTGATGTCTGCATTCAACTCATAGAGTTGAAGATTCCCTTTCATAGAGCAGGTTTGAAAACACTCTTTCTGGAGTATCTGGATGTGGACATTTGGAGCGCTTTGATGCCTACGGTGAAAAAGTAAATATCTTCCCATAAAAACGAGACAGAAGTATTCTCAGAAACAAGTTTGTGATGTGTGTACTCAGCTAACAGAGTGGAACCTTTCTTTTTACAGAGCAGCTTTGAAACTCTATTTTTGTGGATTCTGCAAATGGAATATTTAGATTGCTTTAACGATATCGTTGGAAAAGGAATATCGTCATACAAACTCTAGACAGAAGCATTCTCACAAACTTCTTTGTGATGTGTGTCCTCAACTAACAGAGTTGAACCTTTCTTTTGATGCAGCAGTTTGGAAACACTCTTTTTGTAGAAACTGTAAGTGGATATTTGGATAGCTCTAACAGTTTCGTTGGAAACGGGAATATCATCATCTAAAATCTAGACAGAAGCACTATTAGAAACTACTTTTGTGATATCTGCATTCAAGTCACAGAGTTGAACATTCGCTTTCTTAGAGCACGTTGGAAACACTCTTTTTGTAGTGTCTGGAAGTGGACATTTGGAGCGCTTTGATGCCTTTGGTGAAAAAGGGAATGTCTTCCCATAAAAACTAGACAGAAGCATTCTCAGAAACTTGTTTGTGATGTGTGTACCCAGCTAATGGAGTTGAACATTTCTATTGATAGAGCAGTTTTGAAACACTCTTTTTGTGGAAAATGCAAGTGGATATTTGGATAGCTTGGAGGATTTCGTTGGAAGCGGGAATTCAAATAAAAGGTAGACAGCAGCATTCTCAGAAATTTCTTTCTGATGTCTGCATTCAACTCATAGAGTTGAAGATTCCCTTTCATAGAGCAGGTTTGAAACACTCTTTCTGGAGTATCTGGATGTGGACATTTGGAGCGCTTTGATGCCTACGGTGAAAAAGTAAATATCTTCCCATAAAAACGAGACAGAAGGATTCTGAGAGACAAGTTTGTGATGTGTGTACTCAGCTAACAGAGTGGAACCTTTCTTTTTACAGAGCAGCTTTGAAACTCTATTTTTGTGGATTCTGCAAATTGATATTTAGATTGCTTTAACGATATCGTTGGAAAAGGGAATATCGTCATACAAAATCTAGACAGAAGCATTCTCACAAACTTCTTTGTGATGTGTGTCCTCAACTTACAGAGTTGAACCTTTCTTTTGATGCAGCAGTTTGGAAACACTATTTTTGTAGAAACTGTAAGTGGATATTTGGATAGCTCTAACGATTTCGTTGGAAACGGGAATATCATCATCTAAAATCTAGACAGAAGCACTATTAGAAACTACTTGGTGATATCTGCATTCAAGTCAAAGAGTTGAACATTCCCTTACTTTGAGCACGTTTGAAACACTCTTTTGGAAGAATCTGGAAGTGGACATTTGGAGCGCTTTGATGCCTTTGGTGAAAAGGAAACGTCTTCCAATAAAAGCCAGACAGAAGCATTCTCAGAAACTTGTTGTGATGTGTGTACTCAACTAAAAGAGTTGAACCTTTCTATTGATAGAGCAGTTTTTGAAACACTCTTTTTGTGGATTCTGCAAGTGGATATTTGGATTGCTTTGAGGATTTCGTTGGAAGCGGGAATTCGTATAAAAACTAGACAGCAGCATTCCCAGAAATTTCTTTCGGATATTTCCATTCAACTCATAGAGATGAACATGGCCTTTCATAGAGCAGGTTTGAAACACTCTTTTTGTAGTTTGTGGAAGTGGACATTTCGATCGCCTTGACGCCTACGGTGAAAAAGGAAATATCTTCCCATAAAAAATAGACAGAAGCATTCTCAGAAACTTGTTGGTGATATGTGTCCTCAACTAACAGAGTTGAACTTTGCCATTGATAGAGAGCAGTTTTGAAACACTCTTTTTGTGGAATCTGCAAGTGGATATTTGGATAGCTTGGAGGATTTCGTTGTAAGCGGGAATTCAAATAAAAGGTAGACAGCAGCATTCTCAGAAATTTCTTTCTGATGTCTGCATTCAACTCATAGAGTTGAAGATTCCCTTTCATAGAGCAGGTTTGAAACACTCTTTCTGGAGTATCTGGATGTGGACATTTGGAGCGCTTTGATGCCTACGGTGAGAAAGTAAATATCTTCCCATAAAAACGAGACAGAAGGATTCTGAGAAACAAGTTTGTGATGTGTGTACTCAGCTAACAGAGTGGAACCTCTCTTTTGATGCAGCAGTTTGGAAACACTCTTTTTGTAGAAACTGTAAGTGGATATTTGGATAGCTCTAATGATTTCGTTGGAAACGGGAATATCATCATCTAAAATCTAGACAGAAGCCCTCTCAGAAACTACTTTGTTATATCTGCATTCAAGTCACAGAGTTGAACATTCGCTTTCTTAGAGCACGTTGGAAACACTCTTTTTGTAGTGTCTGGAAGTGGACATTTGGAGCGCTTTGATGCCTTTGGTGAAAAAGGGAATGTCTTCCCATAAAAACTAGACAGAAGCATTCTCAGAAACTTGTTTGTGATGTGTGCACCCAGCTAAAGGAGTTGAACATTTCTATTGATAGAGCAGTTTTCAAACACTCTTTTTGTGGAAAATGCAAGTGGATATTTGGATAGCTTGGAGGATTTCGTTGGAAGCGGGAGTTCAAATAAAAGGTAGACAGCAGCATTCTCAGAAATTTCTTTCTGATGTCTGCATTCAACTCATAGAGTTGAAGATTCCCTTTCATAGAGCAGGTTTGAAACACTCTTTCTGGAGTATCTGAATGTGGACATTTGGAGCGCTTTGATGCCTACGGTGAAAAAGTAAATATCTTCCCATAAAAACGAGACAGAAGGATTCTCAGAAACAAGTTTGTGATGTGTGTACTCAGCTAACAGAGTGGAACCTGTCTTTTGATGCAGCAGTTTGGAAACACTCTTTTTGTAGAAACTGTAAGTGGATATTTGGATAGCTCTAATGATTTCGTTGGAAATGGGAATATCATCATCTAAAATCTAGAGAGAAGCCCTCTCAGAAACTACTTTGTGATATCTGCATTCAAGTCACAGAGTTGAACATTCGCTTTCTTAGAGCACGTTTGAAACACTCTTTTTGTAGTGTCTGGAAGTGGACATTTGGAGCGCTTTGATGCCTTTGGTGAAAAAGGGAATGTCTTCCCATAAAAACTAGACAGAAGCATTCTCAGAAACTTGTTTGTGATGTGTGTACCCAGCCAAAGGAGTTGAACATTTCTATTGATAGAGCAGTTTTGAAACGCTCTTTTTGTGGAAATGCAGGTGGATATTTGGATAGCTTGGAGGATTTCGTTGGAAGCGGGAATTCAAATAAAAGGTAGACAGCAGCATTCTCAGAAATTTCTTTCTGATGTCTGCATTCAACTCATAGAGTTGAAGATTCCCTTTCATAGAGCAGGTTTGAAACACTCTTTCTGGAGTATCTGGATGTGGACATTTGGAGCGCTTTGATGCCTACGGTGAAAAAGTAAATATCTTCCCATAAAAACGAGACAGAAGGATTCTGAGAAACAAGTTTGTGATGTGTGTACTCAGCTAACAGAGTGGAACCTTTCTTTTTACAGAGCAGCTTTGAAACTCTATTTTTGTGGATTCTGCAAATGGATATTTAGATTGCTTTAACGATATCGTTGGAAAAGGGAATATCGTCATACAAAATCTAGACAGAAGCATTCTCACAAACTTCTTTGTGATGTGTGTCCTCAACTAACAGAGTTGAACCTTTCTTTTGATGCAGCAGTTTGGAAACACTCTTTTTGTAGAAACTGTAAGTGGATATTTGGATAGCTCTAATGATTTCGTTGGAATCGGGAATATCATCACCTAAAATCTAGACAGAAGCCCTCTCAGAAACTACTTTGTGATATCTGCATTCAAGTCACAGAGTTGAACATTCGCTTTCTTAGAGCACGTTGGAAACACTCTTTTTGTAGTGTCTGGAAGTGGACATTTGGAGCGGCTTTGATGCCTTTGGTGAAAAAGGGAATGTCTTCCCATAAAAACTAGACAGAAGCATTCTCAGAAACTTGTTTGTGATGTGTGTACCCAGCCAAAGGAGTTGAACATTTCTATTGATAGAGCAGTTTTGAAACGCTCTTTTTGTGGAAAATGCAGGTGGATATTTGGATAGCTTGGAGGATTTCGTTGGAAGCGGGAATTCAAATAAAAGGTAGACAGCAGCATTCTCAGAAATTTCTTTCTGATGTCTGCATTCAACTCATAGAGTTGAAGATTCCCTTTCATAGAGCAGGTTTGAAACACTCTTTCTGGTGTGTCTGGATGTGGACATTTGGAGCGCTTTAATGCCTACGGTGAAAAAGTAAATATCTTCCCATAAAAACGAGACAGAAGGATTCTGAGAAACAAGTTTGTGATGTGTGTACTCAGCTAACAGAGTGGAACCTTTCTTTTTACAGAGCAGCTTTGAAACACTATTTTTGTGGATTCTGCAAATGGATATTTAGATTGCTTTAACGATATCATTGGAAAAGGGAATATCGTCATACAAAATCTGGACAGAAGCATTCTCACAAACTTCTTTGTGATGTGTGTCCTCAACTAACAGAGTTGAACCTTTCTTTTGATGCAGCAGTTTGGAAACACCCTTTTGGTAGAAACTGTAAGTGGATATTTGGATAGCTCTAACGATTTCGTTGGAAACTGGAATATCATCATCTAAAATCTAGACAGAAGCACTATTAGAAACTACTTGGTGATATCTGCATTTCAAGTCACAGAGTTGAACATTCCCTTACTTTGAGCACGTTTGAAACACTCTTTTGGAAGAATCTGGAAGTGGACATTTTGGAGCGCTTTGATGCCTTTGGTGAAAAGGAAACGTCTTCCAATAAAAGCCAGACAGAAGCATTCTCAGAAACTTGTTTGAGATGTGTGTACTCAACTAAAAGAGTTGAAACCTTTCTATTGATAGAGCAGTTTTGAAACACTCTTTTTGTGGATTCTGCAAGTGGATATTTGGATTGCTTTGAGGATTTCGTTGGAAGCGGAAATTCGTATAACAACTAGACAGCAGCATTCCCAGAAATTTCTTTCGGATATTTCCATTCAACTCATAGAGATGAACATGGCCTTTCATAGAGCAGGTTTGAAACACTCTTTTTGTAGTTTGTGGAAGTGGACATTTCGATCGCCTTGACGCCTACGGTGAAAAAGGAAATATCTTCCCATAAAAAATAGACAGAAGCATTCTCAGAAACTTGTTGGTGATATGTGTCCTCAACTAACAGAGTTGAACTTTGCCATTGATAGAGAGCAGTTTTGAAACACTCTTTTTCCTGAATCTGCAAGTGGATATTTGGATAGTTTGGAGGATTTCGTTGGAAGCGGGAATTCAAATAAAAGGTAGACAGCAGCATTCTCAGAAATTTCTTTCTGATCTCTGCATTCAACTCATAGAGTTGAACATTCCCTTTCATAGGGCAGGTTTGAAATACTCTTTCTGTAGTATCTGGATGTGGACATTTGGAGCGCTTTGATGCCTACGGTGAAAAGTAAATATCTTCCCATAAAAACGAGACAGAAGGATCTGAGAAACAAGTTTGTGATGTGTGTACTCAGCTAACAGAGTGGAACCTCTCTTTTGATGCAGCAGTTTGGAAACACTCTTTTTGTAGAAACTGTAAGTGGATATTTGGATAGCTCTAATGATTTCGTTGGAAACGGGAATATCATCATCTAAAATCTAGACAGAAGCCCTCTCAGAAACTACTTTGTGATATCTGCATTCAAGTCACAGAGTTGAACATTCGCTTTCTTAGAGCACGTTGGAAACACTCTTTTTGTAGTGTCTGGAAGTGGACATTTGGAGCGCTTTGATGCCTTTGGTGAAAAAGGGAATGTCTTCCCATAAAAACTAGACAGAAGCATTCTCAGAAACTTGTTTGTGATGTGTGCACCCAGCTAAAGGAGTTGAACATTTCTATTGATAGAGCAGTTTTCAAACACTCTTTTGGTGGAAAATGCAAGTGGATATTTGGATAGCTTGGAGGATTTTCGTTGGAAGCGGGAGTTCAAATAAAAGGTAGACAGCAGCATTCTCAGAAATTTCTTTCTGATGTCTGCATTCAACTCATAGAGTTGAAGATTCCCTTTCATAGAGCAGGTTTGAAACACTCTTTCTGGAGTATCTGAATGTGGACATTTGGAGCGCTTTGATGCTACGTGAAAAAGTAAATATCTTCCCATAAAAACGAGACAGAAGGATTCTCAGAAACAAGTTTGTGATGTGTGTACTCAGCTAACAGAGTGGAACCTGTCTTTTGATGCAGCAGTTTGGAAACACTCTTTTTGTAGAAACTGTAAGTGGATATTTGGATAGCTCTAATGATTTCGTTGGAAACGGGAATATCATCATCTAAAATCTAGAGAGAAGCCCTCTCAGAAACTACTTTGTGATATCTGCATTCAAGTCACAGAGTTGAACATTCGCTTTCTTAGAGCACGTTTGAAACACTCTTTTTGTAGTGTCTGGAAGTGGACATTTGGAGCGCTTTGATGCCTTTGGTGAAAAAGGGAATGTCTTCCCATAAAAACTAGACAGAAGCATTCTCAGAAACTTGTTTGTGATGTGTGTACCCAGCCAAAGGAGTTGAACATTTCTATTGATAGAGCAGTTTTGAAACACTCTTTTTGTGGAAAATGCAAGTGTATATTTGGATAGCTTGGAGGATTTCGTTGGAAGTGGAAATTCAAATAAAAGGTAGACAGCAGCATTCTCAGAAATTTCTTTCTGATGTCTGCATTCAACTCATAGAGTTGAAGATTCCCTTTCATAGAGCAGGTTTGAAACACTCTTTCTGGAGTATCTGGATGTGGACATTTGGAGCGCTTTGATGCCTACGGTGAAAAAGTAAATATCTTCCCATAAAAACGAGACAGAAGTATTCTCAGAAACAAGTTTGTGATGTGTGTACTCAGCTAACAGAGTGGAACCTTTCTTTTTACAGAGCAGCTTTGAAACTCTATTTTTGTGGATTCTGCAAATGGATATTTAGATTGCTTTAACGATATCGTTGGAAAAGGGAATATCGTCATACAAAATCTAGACAGAAGCATTCTCACAAACTTCTTTGTGATGTGTGTCCTCAACTAACAGAGTTGAACCTTTCTTTGATGCAGCAGTTTGGAAACACTCTTTTTGTAGAAACTGTAAGTGGATATTTGGATAGCTCTAACGATTTCGTTGGAAACGGGAATATCATCATCTAAAATCTAGACATAAGCACTATTAGAAACTACTTTGTGATATCTGCATTCAAGTCACAGAGTTGAACATTCGCTTTCTTAGAGCACGTTGGAAACACTCTTTTTGTAGTGTCTGGAAGTGGACATTTGGAGCGCTTTGATGCCTTTGGTGAAAAAGGGAATGTCTTCCCATAAAAACTAGACAGAAGCATTCTCAGAAACTTGTTTGTGATGTGTGTACCCAGCTAATGGAGTTGAACATTTCTATTGATAGAGCAGTTTTGAAACACTCTTTTTGTGGAAAATGCAAGTGGATATTTGGATAGCTTGGAGGATTTCGTTGGAAGCGGGAATTCAAATAAAAGGTAGACAGCAGCATTCTCAGAAATTTCTTTCTGATGTCTGCATTCAACTCATAGAGTTGAAGATTCCCTTTCATAGAGCAGGTTTGAAACACTCTTTCTGGAGTATCTGGATGTGGACATTTGGAGCGCTTTGATGCCTACGGTGAAAAAGTAAATATCTTCCCATAAAAACGAGACAGAAGGATTCTCAGAAACAAGTTTGTGATGTGTGTACTCAGCTAACAGAGTGGAACCTTTCTTTTTACAGAGCAGCTTTGAAACTCTATTTTTGTGGATTCTGCAAATTGATATTTAGATTGCTTTAACGATATCGTTGGAAAAGGGAATATCGTCATACAAAATCTAGACAGAAGCATTCTCACAAACTTCTTTGTGATGTGTGTCCTCAACTTACAGAGTTGAACCTTTCTTTTGATGCAGCAGTTTGGAAACACTATTTTTGTAGAAACTGTAAGTGGATATTTGGATAGCTCTAACGATTTCGTTGGAAACGGGAATATCATCATCTAAAATCTAGACAGAAGCACTATTAGAAACTACTTGGTGATATCTGCATTCAAGTCAAAGAGTTGAACATTCCCTTACTTTGAGCACGTTTGAAACACTCTTTTGGAAGAATCTGGAAGTGGACATTTGGAGCGCTTTGATGCCTTTGGTGAAAAGGAAACGTCTTCCAATAAAAGCCAGACAGAAGCATTCTCAGAAAACTTGTTTGTGATGTGTGTACTCAACTAAAAGAGTTGAACCTTTCTATTGATAGAGCAGTTTTGAAACACTCTTTTTGTGGATTCTGCAAGTGGATATTTGGATTGCTTTGAGGATTTCGTTGGAAGCGGGAATTCGTATAAAAACTAGACAGCAGCATTCCCAGAAATTTCTTTCGGATATTTCCATTCAACTCATAGAGATGAACATGGCCTTTCATAGAGCAGGTTTGAAACACTCTTTTTGTAGTTTGTGGAAGTGGACATTTCGATCGCCTTGACGCCTACGGTGAAAAAGGAAATATCTTCCCATAAAAAATAGACAGAAGCATTCTCAGAAACTTGTTGGTGATATGTGTCCTCAACTAACAGAGTTGAACTTTGCCATTGATAGAGAGCAGTTTTGAAACACTCTTTTTGTGGAATCTGCAAGTGGATATTTGGATAGCTTGGAGGATTTCGTTGTAAGCGGGAATTCAAATAAAAGGTAGACAGCAGCATTCTCAGAAATTTCTTTCTGATGTCTGCATTCAACTCATAGAGTTGAAGATTCCCTTTCATAGAGCAGGTTTGAAACACTCTTTCTGGAGTATCTGGATGTGGACATTTGGAGCGCTTTGATGCCTACGGTGAGAAAGTAAATATCTTCCCATAAAAACGAGACAGAAGGATTCTGAGAAACAAGTTTGTGATGTGTGTACTCAGCTAACAGAGTGGAACCTCTCTTTTGATGCAGCAGTTTGGAAACACTCTTTTTGTAGAAACTGTAAGTGGATATTTGGATAGCTCTAATGATTTCGTTGGAAACGGGAATATCATCATCTAAAATCTAGACAGAAGCCCTCTCAGAAACTACTTTGTTATATCTGCATTCAAGTCACAGAGTTGAACATTCGCTTTCTTAGAGCACGTTGGAAACACTCTTTTTGTAGTGTCTGGAAGTGGACATTTGGAGCGCTTTGATGCCTTTGGTGAAAAAGGGAATGTCTTCCCATAAAAACTAGACAGAAGCATTCTCAGAAACTTGTTTGTGATGTGTGCACCCAGCTAAAGGAGTTGAACATTTCTATTGATAGAGCAGTTTTCAAACACTCTTTTTGTGGAAAATGCAAGTGGATATTTGGATAGCTTGGAGGATTTCGTTGGAAGCGGGAGTTCAAATAAAAGGTAGACAGCAGCATTCTCAGAAATTTCTTTCTGATGTCTGCATTCAACTCATAGAGTTGAAGATTCCCTTTCATAGAGCAGGTTTGAAACACTCTTTCTGGAGTATCTGAATGTGGACATTTGGAGCGCTTTGATGCCTACGGTGAAAAAGTAAATATCTTCCCATAAAAACGAGACAGAAGGATTCTCAGAAACAAGTTTGTGATGTGTGTACTCAGCTAACAGAGTGGAACCTGTCTTTTGATGCAGCAGTTTGGAAACACTCTTTTTGTAGAAACTGTAAGTGGATATTTGGATAGCTCTAATGATTTCGTTGGAAATGGGAATATCATCATCTAAAATCTAGAGAGAAGCCCTCTCAGAAACTACTTTGTGATATCTGCATTCAAGTCACAGAGTTGAACATTCGCTTTCTTAGAGCACGTTTGAAACACTCTTTTTGTAGTGTCTGGAAGTGGACATTTGGAGCGCTTTGATGCCTTTGGTGAAAAAGGGAATGTCTTCCCATAAAAACTAGACAGAAGCATTCTCAGAAACTTGTTTGTGATGTGTGTACCCAGCCAAAGGAGTTGAACATTTCTATTGATAGAGCAGTTTTGAAACGCTCTTTTTGTGGAAAATGCAGGTGGATATTTGGATAGCTTGGAGGATTTCGTTGGAAGCGGGAATTCAAATAAAAGGTAGACAGCAGCATTCTCAGAAATTTCTTTCTGATGTCTGCATTCAACTCATAGAGTTGAAGATTCCCTTTCATAGAGCAGGTTTGAAACACTCTTTCTGGAGTATCTGGATGTGGACATTTGGAGCGCTTTGATGCCTACGGTGAAAAAGTAAATATCTTCCCATAAAAACGAGACAGAAGGATTCTGAGAAACAAGTTTGTGATGTGTGTACTCAGCTAACAGAGTGGAACCTTTCTTTTTACAGAGCAGCTTTGAAACTCTATTTTTGTGGATTCTGCAAATGGATATTTAGATTGCTTTAACGATATCGTTGGAAAAGGGAATATCGTCATACAAAATCTAGACAGAAGCATTCTCACAAACTTCTTTGTGATGTGTGTCCTCAACTAACAGAGTTGAACCTTTCTTTTGATGCAGCAGTTTGGAAACACTCTTTTTGTAGAAACTGTAAGTGGATATTTGGATAGCTCTAATGATTTCGTTGGAATCGGGAATATCATCACCTAAAATCTAGACAGAAGCCCTCTCAGAAACTACTTTGTGATATCTGCATTCAAGTCACAGAGTTGAACATTCGCTTTCTTAGAGCACGTTGGAAACACTCTTTTTGTAGTGTCTGGAAGTGGACATTTGGAGCGCTTTGATGCCTTTGGTGAAAAAGGGAATGTCTTCCCATAAAAACTAGACAGAAGCATTCTCAGAAACTTGTTTGTGATGTGTGTACCCAGCCAAAGGAGTTGAACATTTCTATTGATAGAGCAGTTTTGAAACGCTCTTTTTGTGGAAATGCAGGTGGATATTTGGATAGCTTGGAGGATTTCGTTGGAAGCGGGAATTCAAATAAAAGGTAGACAGCAGCATTCTCAGAAATTTCTTTCTGATGTCTGCATTCAACTCATAGAGTTGAAGATTCCCTTTCATAGAGCAGGTTTGAAACACTCTTTCTGGTGTGTCTGGATGTGGACATTTGGAGCGCTTTAATGCCTACGGTGAAAAGTAAATATCTTCCCATAAAAACGAGACAGAAGGATTCTGAGAAACAAGTTTGTGATGTGTGTACTCAGCTAACAGAGTGGAACCTTTCTTTTTACAGAGCAGCTTTGAAACACTATTTTTGTGGATTCTGGGTCCACACTCAGGGGACTTTCCCTCCGAGGGACCCTCCACTGGGCCTCCTGCGTGGCCACAAGGACAGAGCTGCAGAAAGACAGGGTCCCCAAGAGACACCTGGACCCCCTTCCCAGAAGGCAGCCTGCTGGGCCGCAGCCAAGGGCCTGGTGCATGCTGTGGGGACGTCTCACCCGCGCCGGCTGGTATTGGCCCAGTCAAAGAGCTTGTGCATGGCGGTCACGCCTTCCCGCCCCATGGGGGCCACGTCCCCGCCTGTCATGATGGCGTAGTCCATGCCTGAGTGCAGGGCGAGTTTCTGGACAAGAAGAGTGAGGGGAGACAGAACAGATGTTCCCTCCGCCTCTGCAAAGCCTGCACACTCCACCCGGAGTGTGGAGGCTCTGCCCAGCGTCCCTGCCTGGAAGCCACAGCCTGCCAGACAGGAGACGTCCCCCGCCTGGAAGATGGGAGGCGCTGCTGCCCACACCAG-

**BLAT search results for chr13:41706593 left-clipped sequences**

The first 16931-bp match to 16 fragments in chr13(-) from chr13:15562154-17496540 at 13p11-q11 (97.4%-98.5 identity, active αSat HOR, hor_13_3, S2C13/21H1L); BLAT the last 543-bp sequence (highlight yellow) matches to 16 fragments in chr13(-) from chr13:15562601-15843915 (identity 99.3%), the most proximal fragment chr13(-):15563145-15562601 at 13p11.1 was assumed for break-fusion. BLAT the next 475-bp (underlined) matches to chr1(-):890898-890424 at 1p36.33 (100% identity, ATAD3201/202)

**The fusion sequence (f1): chr13:15562601::[chr1:890898_890424ins]::41706593**

1. **Chr13:70420369 right-clipped sequences**


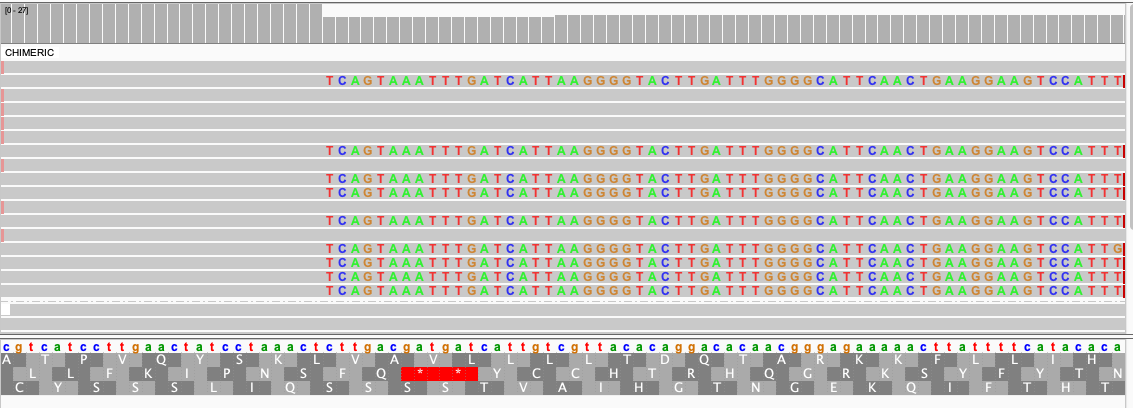


Chr13:70420369---

TCAGTAAATTTGATCATTAAGGGGTACTTGATTTGGGGCATTCAACTGAAGGAAGTCCATTTGGGAATTCTTTTTCATATCGGTATGGTAGCATGGATTTATTTTACGCTTTGCCATCACTTCCATAATAAGATTTTGTGGACTGCCCCATGGTACAAGAAAGGCTTTCAGGCAAACGACTGTCCTCTGTGATGACTTGTCCAGTCTAGAGACTCTGATACAGGGCCAAAAGTCCTATCATGGTGGGAATGGGGTACTAGCAGCCTGACGACAGAGGGTATGTGAGGAGTCTAGCAGCCACAAAAGACAGAAAATGTGTGAGGAAAATTCTTCCAATTGCTGAAAATGTGAAATCCTGTGAAGACTCAGTTTTCATTGAAAGTTAAAATAGAAATTCAAATAGCAGGATTTGAATTTCTATTTTATAGTTAATATATAGATACAACATACACCAGCTGTGCATGTGTGTGTGTGTATATATGTGTATGTCACATACAATGGTAAATGGATCACATAATTTCTTTTTTGAAATGGAATTGATCAGAATCCCTGTGTTTATGGGGCACAATCTCATAGCATCACTGCAAAGCACAGGAGTGTCTGTGTCTGCCGCTGCATGTTTCATACATCCTAACTTACCAAATCATGTCTTAGCATAGCTGATGCATTTTGTCCTGATGAATTTTGACATGTCCCAGCAAAACAGCACACTAAATTGCTACCAACCCAGCAAATCACAGCAAAGAGTCAAGTACTACACAGAGGCACCTACACGTATCAGTAAATACATCAAAGGGAGGTAAGAGTAATCTGATTACATAATGTAGTTCAACATACATACAGCAGTTTAAAAGTGTTTGAATTGTTCTTATGTTGCCTTATTTTTATATTACTTTAAGGTATTTTTATTAGAAATATTTATCAAACCAGTAATTTAAAAGATATTATAGAGTTGCTGTAGGAAACTAATTAATCAAGGCAGTCTGCTTTTTGTCTTTCTAAATTTTATGTCTGTGATGTTTGCCAAATTTCTTATTTTAAATATTCAATTGTATCTAATTTCAGATTTATAATATTTATTCATTTTTATAAGAAATTCTAATTATTAAAAAATAACATTATAAAAGCTTCAGATTTCACACAATCTGAATCTGTCCCTTCTATATGTCAGCATTGCTAGTGTAGCTTTAGTGACTGAAACCCATTTGTCTGAATGGCCTGTCTTATCTAACATACTAGAATTCTTTATAACTAAAATGTTATTATACTTCCATTTGATATTTGCCCAATCACTTTACAAGGTTAGGTTGGGTCCATGCAAGGCTTTTTGTTTTGTTTTGTTTTGTTTCTTCAATCTCAAGGCAGGAGGATTTACCCTGGTTGAAGTATTAAATGTTTTAAGATATTTACAAAATCTGTGAGCATTTATTATTAGGCAAGTTCGCCTGTTCATTTCATATTCATCATAAAGAAAGCAGATGGAAGATCTTGACCAACCGTAACCATTTTTTGGCAATATTTAACACTAAAATTTTAGCTGGTAGCACCTGTTTTCTAAGGGTAAAGACTATAATTCCCAAGCACTTCACTGTAAGACTTTTGACGATTCTTCTGTTATTGTTCTATTAGCAGTACTTACTCTTGGAAAACATTATTTTATTATTCTGTGAAATAATTTTGGTTAACATACATTGGCATTACTATTACAGATTAAAAGTTTTATAAAATAGTGTATTTTCAGTTAAATGAAACAGAAACTCTGTGACTCTAGGTGTTTTCTGGCATGGATAGTTAAAATTTCAGAAGAGAGGTGGCCTTCAGTTATGCAATCACATCAGGCTTTGTTTCTTGAATTCTTTTAGACTCATCAGTTGTATCAGTTCTCCTTCAAACTTGTTTGCCTTACCAGTAGCAGCTTGCGCAAAAATAGAGAAACGATCAAATCTTATATTCTCCATATGAAAATATGAGAGGATATGATATGCCAATTGTTTAAATTAACCAAAGTTAGAAAAACAAAATAAATCGAATCTCTAACATGTGCAAGTATGGCAGGCACCTGTACAAGTTTTCTCACTGAATTTTACAACCTTTCTTAGATTGATAGTATTTTCTTCATTGTTCAAATTTCATACCTAAAGATCAGTGAAGTTAAATCATTTACCTATGAAACTGAAAAATGATAAAACATTTTAAGAGGTCCTCCTACCAAGCTGATGTGGTTTGGCTGTGTCCACACCAAAAACATCCTCTTGAATTGTAATCCGCATAATCCCCATAAAAGGCAGGACCAGGTGGAGATAATCTTATCATGGGTGCGGTTTCCCCCGTGCTATTCTCGTGATAGTGAGTCTCACGAGATCTGATGGTTTTCTAAGCGTCTGGCATTTCCCCTGCTTGCACTCACTCCATCCTTCACCCTGTGAAGGAGCCTGCTTCTCCTTTGCCTTCCATCATGACTGTAAGTTCCCTGAGGTCTCCCCAGCAATGCAGAACTGTGAGTCAATTAAACCTCTTTCCTTTATAAATGACCCAGTCTTGGGTATTGCTTCATAGCAGTGTGAGAATGAACTAATATACAAGCCCATACTGTTTTTAAACTCATGCCATGCAGATTTAGAATACCTTCTCAAACACAGACTTTTCAGTCCTATTTATACATGGATAGCTATGCCCTTAGTGTTTATGCTACAGGAATTCTTGAAATTTTCCAGTAGAAGTTTTTTCAAATGTGATATTTCTTCTATCTGTTGAGGCTCATGCTGTGTGATAAACTTACTTCTCAAATAGATCCTAAATAATCTATCTGAAACTTTCTCTTCTACACACAAGCAAATAGCCAATAACTAACATAGGACTTGAGCAATTTCTAAATATGCACTCATCTTGGGTTATGATTATATTTAGATGCTATCAAAATAGAAGACAAATTTACCATCTAAATGTTTGATGTGCTCAATAGCAAACAATCCAGATTTACACCTGATTTTCTGTTATGATAATGTATAAACATAGAGCAAGTCTATTTTCATGTTTAAAGGCAGGTGACAGAAATCCGGGCATGTAGGGGTTGAGGCATTCAAGAAAATAGGTGAGAAAGAGCTGCACGTCTGGTACACAGTAGTACTGCTCACAGGCAAGTCAATTGCCTTCCTTGTGGTTTGAGATATCAGTTAATATTTTATACATTTCCAATTATGAGAGAAGAAATACCAGTAACATTCCTAAGCCTGCAAGCTGGCTGGGTATTTGAGAAGTTACCTAAATTATATCTCTGAGTGTATTTTAAAATGATTTTAATTACTATAAATTTTCAGTTTCCATAATAAAATATACTTAACTATTTACATATTGCCCAAGATTGGGCTGTAAGCTCTTTTCTCAGCAGAGGTCACCAAATAGGCCTCAGCCAATGTAAAGTCTACCCACACATTCTGTGTGGCTGTTTCCTGAGGCCCACTGTGTTTTAAAAACAAATGTAACTACACGCATTTGATCTCTGGTTCCTGTTCAGATGTTAAGAGTGTGCATATTTTTTCTTGGCTTCTCCAGGAATTTATAATATCTACAATCATTTCAGTGTGCCTCCCTATTCTATGGTCCTTTCATCCACATGCAACCACAGAGATGCTTCTAGTTCCTTCTATGTGCCCAGGATGATGAAATGTAAATTTCAACCCGAGCTCACCTCTGAGATTTAGACCCGTATACCAAAGGCACTTTAAGGGTTTCTATTTCAATAAATGCCATTGCTATCCAGTTAATGACGCAAACCAGAAAGTTACAAGTCAGTTATGATGCCTCCTCCCCTTCAACTGCCATAGCTAGTCCAACACAAAAACCTTTTAATTTTTTCTTCCCAAATGTTCCCAGAATGGGATCATTTCTGTTCATTTCTTATTACCCTCTTGAATATACTCTCAAAGTGCCCTGTACTTTTCTTTACATGTGATTTGTAAATATATAGCCATGGGTGGAATCACCAATATCGATTTTCACCATTAAGTTCTAAACTTCAAGTGAATAGTGACAATGTATATTCTGGTCCCTGTTCATCTCCAGAAAATACCAAAAATTTTAGGAAAAATAAATGTGGAACTTTTAGCATGTTTCCATTTCTTTCTTTTTATGTATATATATACACACACACACACACATATATATATATAATACTTTAAGTTCTAGGGTACATGTGCACAACGTGCAGGTTTGTTACATATGTATACATTTGCCATGTTGGTGTGCTGCACCCATTAACTCGTCATTTGCATTAGGTGTATCTCCTAATGCTATCCCTCCCCCCTCCCCCCAGCCTACACAGACGCTGGTGTGTGATGTTCCCCTTCCTGTGTCCAAGTATTCTCATTATTCAATTCCCACCTATGAGTGAGAATACGTGGTGTTTGGTTTTTTGTCCTTGCGATAGTTTGCTGAGAATGATGGTTTCCAGCTTCTCATCCATGTCCCTACAAAGGACAAGAACTCATCATTTTTTATGGATGCATAGTATTCCATGGTGTGTGTGTATATATATATGTATGTATGTATGTATATGTGTGTATGTGTGTATGTATGTATATGTGTGTGTGTATATATATATATGTGTGTGTGTGTGTGTGTGTGTGTGTGTGTGTTGCTCTGTCGTCCAGGCTGGAGTGCAGTGGCACGATCTTTGCTCACTGCAAGCTCCACCTCCCGGGTTCATGCCATTCTCCTGCCTCAGCCTCCGGAGTAGCTGGGACTACAGGCGTCCGCCACCACGCCTGGCTAATTTTTTTGTATGTTTAGTAGAGACAGGGTTTCACCATGTTATCCAGGATGGTCTCGATCTCCTGATCTTGTGATCTCCCCACCTCGGCCTCCCAAAGTGCTGGGATTACAGGTGTGAGCCACCACGCCCAGCCTCCATTTCCTGTTTTTTAAATGATAGCTTTATTATTATCATTGTATAGAAATGTTTACTGTATTTTTTATTTTTATGAAGTTTCCTGCGAGTAAAAATTGTATCAAATGTTAGAAAATACCAGAAAGTTTCAGTCAAGAAAGGTTTTATATTTTACATGCCCAAAAATAGAGTATAAAGTTACTTATCTACTTTTTAAGCATTGATTTTTATATGGTTTTTATGTTTGTCAATTTAGTTTGATATTTTAATTTTTCCAATTTTATTATATTTGATCTCTTGTGTGGCCCAGTGATATTTTCTGGCTCTGCGTCCCCACCTAAATCTCATCTCAAATTGTCATTCCCATAAACATCCCCACATGTCTAGGAAGGGACCAGGTAGGAGGTGATTTGATCATTGGGGCAGGTCACCCATGCTGTTCTCCTGATAGTGAGTGAGTTCTCACAAGGTCTGATGGTTTTATAAGTGCTTGACAGTTCCTCCTTCACATTCTCTCTCTCGCCTTCTGCCATGTAAGAAGTGCCTGCTTCCCCCTCCCCCACGATTACAAGTTTCCGGAGGCCACGTCTGCCATGTGGAACTGTGAGTCAATTAAACTTTTCTTTTTTTTCTTTATATATATATATATATATATATATATATATATATATATTTTTTTTTTTTTTTTTTTTTTTTTTGAGATAGAGTCTCACTCTGTTGCTAGGCTGGAGTGCAATGGTGTGACCTTGGCTCACTGCAACCTCTGCCTCCCAAATCTAAGCAATTCTCCTGCCTCAGCCTCCAGAACAGCCTGGATCACAGGTGCACACCACCACGCCTGGCTAATGTTCTGTACTTTTAGTAGAAATGGGGTTTCACCATGTTAGCCAGGCTGGTCTTGAACTCCCAACCTCAGGTGATCCACCCGCCTCGGCCTCCCAAAGTGCTGGGGTCACAGGCATAAGCCACTGTGCCCTGGGCTTAAACTTCTTTCTTAATAAATTACCCAGTCTCAGGCAGTTCTTTATAGCAATGTGAAAACAAACTAATACCATTTGACAGAAATTTATATTATTTCTATAAATTGTCGTTTAGAGCCTTTTCTCAATTCTATTTGGGTATTAATGTTTCCTAAACCAATAATAAGAGCTATTTGTATATTAAAATTATTTGATAATATTATTTATATATTAATATTCATAACATTAACATTTATAATACATATAAATAGAAATAGTAATATTATTTACTTGCTAAATATTAGTGCTTTTGTTATGAGATGGGGTCTCACTATACTGTCCAGGCTGCTCTTGAACTCCTGGGCTTAAGTGATTCTCACTCTATTGTCCAGGCTGCTCTTGAACTCCTGGGCTTAAGTGATTCTCCTGCCTCAGTCTGTGGAGTAGCTGAGACTTAGTGCTTTTTTAATAGTAACTATTTTCATTTTAATTCTATTTGTGACATTTTGATGTGCCAAGTATTTTACAGTATATTTAACCAAAGCTGAACTTTATTTTTGCCTGCCCATTCTGTGATAGGAATGCTTTTGCCCACTTAAAATAGAGATGAACACAAACTGTTGTCTATTTGTTCTGAATTTCAGGTTTTCAGATACAGTTTTTAAATTCATGAAGTTTTTATAATTTAAATAACCTTACAAATATAATGCATGAAATAGTTTGTATTATGAAATGATTATTACAGATAAAGCAAAAGTCCCCTTTGACCCCCAATCCTAAATCCATCTAGTAGATATTGAGCCAGGACAACAATTTATCCCGTTGTACATGATGAGGGAAGAAGTGTAGGGATAGTTTATAGAGGGAGGTAATTCCATTTACTGACCAGAGTGTAAAGATCTCTGATACCTTATCTAATGATAACAGAACAGGGAATCATTCAGCAGAACTGAAGAGTGGTTTACATTCACAGGGCCACAGCGTTGAGAAATGTCAGAAACACAGTAGGAGTTTAGCCAAGCAGAGCCACACTCCTGAGAGTGGCAACACCACCCACTCCAGCAACAGAACACACACCTTATGGAGATGGAAATGGCTCCATAATGAGAAAAGCTAGCCAAGGCCCCTCCAAATAGTATTTAGAGGTGGTGTTAAATCATAACAGGTATTTGGGGAGACATTTTAAAAGATGTTTTGTGTGGTTGTTTTGGTTTTTGTTCTTAACCATAGGAAAAGAATCAAGTCATAAGTATCATTTACAGATGGAGTGGCCAAGAGGATTTAGATATGGTTTTGGATACACTGGATGTGCAGAGTACTAACATATTGTTCCTCCTACTAAATTTCAACAAAGTCAGAACAGATATTATTATTTAGTTGCCCTTCAACTCTGCCATCTTTTCTACTGTTTTGACATCACTGCCAGGAGAAACATATGGTCTAGGAAGAAGCAAAAGCCCTAAGCCCTTCCTTTGGTCCCTGTGTAATTTCGCTGAGACTTGTAGCATCAGAGAGGCCTGACACTGAACCTTCTTACACTTTCAAACTTACGTTTCACTGTGTCCTCCTTTATGCCCCTGATGCCTTCCCTGGCTGTTTCTGTATTATGTTTTGATATTTACTAATTTATTAAACTTTTTAACGAAATTATTTAGCTTCATTAACCAGTGCTAATTGATGATGGATTTATATTTTGTAATTTCTATAGTATTTCTTATTACTTTGTTTTTAGTTTTGATTAATCTCAGTTTTCAGATTTATTTAGTTTTTTTTGGTCTAGTTTTCTAAGTTGAACACTTCATTTATTTAAAACATTTGTATTTACAGTAGATGTATTTAAAGTTCTTAACCCCTTTAAGATATTGCTTTATCTACATTTACATATGTCATGTTTTTGTTGCCATTCAATTCAAATTATTTTCAAAATTTTAGCTCGATTTTTCCATATCCCCCTCAATTGTATAGAAATATATTTTCTTAATTGCAGTATGTTACTTATTTTCTATTTTTTCTAATTGTATTGAACTGTGGTAAATAATTATGGTTTTAATTGTGAGAAATTTGCTGAGATTTCTCATGTAGTTTAATGCATGGTTATTTTAATAATAACTCACTGCCATATATTCATTGTTTTAAAAATGTATCTATACATGTCTATCATTTCGAAAAAGATTGTTGTGTTATTCAATTGCTCCTTATAGCCATTTATTTTCTGTCTGTGGAGTGTTCCACTTCTGGCATAGCATATTAAAAATTTCTAAACATTTCTAGGGATCTGCCAATTCTCCTGGTAGAACTTTACTCTTGAGTTCTCTCTTTTACCCTTTAAACAAAACTATTTTTAAAAATTTTTCCAGACTTATGATATCCTGTCTTTTGGACCTCAAAAATTGGGCTTTGTCATTTAAGATTTCAAGACAAGTACTCTTAGTAAGGTTACCAATGAAATTCCTGTTGCTAAATGCAATACAAAATATTATTTTGTATTTTATTAAGTACAAATAAAATTATTTATTTTATTATTTTGTATATTATTAAGAAAAGAAGTTTAAGCCCAGGCTTCATCTTTGATAAAGGTAAGCACACCTCTGTCCTGAAAATATTTATTTTTTGGACTTCCTTAGCAGAAACCACAATTCACTAATTTTCATTCTAATTACCTGGCCATTCCTCTAAGTCAACTTTAAGAACAGTTTCTTTGTTCTCTGAATTCTTGCTCTTTCAGTGGCTCAAGCTCCAATCCTTGGCTTTTTATTTAAATTAAGTCCCATGATCCTTTAGTCAGACTCAGAGCTCAATTATAAATCAGCAGGTCTAGTATGTTTTGGCTATAAATAAATCATCAGGAAATATTATTTGTCTACCCATTGAAACCTAAATGTAATATTTCCATCTGTTGCATTAGTGGCATAGCCAGAAAATCCAGAAAATTCTCCTGGAATGATAATGACAAGGATACTCCATGATAAATATATAAACTATTCTAGGTAAAGTCTTTACCCTTTTGATAAAAATAGATTAAATTCACTATAAACACATAAAGATATATAATACGAAAGAATGTCATGAAGAAATTACTCAGTATAACAATATTTAACATTATATAGACATCATTGTAGGTATCTTTTCATGCATAAAGTGTGATCAAAAGTCATGACGCTTTCCCTTTCTGCATGAGAATCCAGCTTATGAGTAACATCTTCATTCTTTCCCCTTCTGAGCTAATAACATGAGAAATGTTAATTATGATACTAGGCAACAGTACCTACTGGAAGGATTCAGAGAAATTACAAATAAACTATAAATATCTGATGGTGAGGTTATAGCTTCCTTGAGTGTGGTGACATGTAATAATTGGGCTTAATTTTTGAGGGAACATTCACACCTATTCTTATGGGATTGTTATAAAAAAGATTGAATCCTGTGAGGCATGAAGATGATATGCCAGGGCATACCTTGCATTCACCTAATGCAGAGTTGATTTCCTTGAACCTCAGCTTACTCCTACATGGCTATTTGAAGTGATATACACATGGCTGCTATGACTCTCAGTAAGAGTATCCTGACACTGCACTGCTGGAAGTTGTCCTGAAGCTTTCTTTGCAGGCTGGTGCCAAGGGAGGCCTATGAGGATCTTGTGAATCTGAATCTTTTGTAAATCTAAAGACAATTACCATGGCAGATGTTATAGTTTGAGAGCAGAGTAGACACAATTTTATTTAATATATCTTTGAATTGAATACTAAAGAATACAGTACAGTGAAACTCAAGTAATGGTAAACTTTAAACATTTCTTCAACATAAATGTGTTCTTATTTAAAAAGATTCCTATTTGTAAAAAAAAAATGAAAATATATTTAGAATAAGGATGTGTATGTATATGTGTGTATTTAAATGTGCATTATACCTTACTATTTACTGACTCTCTGCTCTGAAAGGTGAGAAAATTATTCCTCTTAAGCTATGTTTAGTTTCCCATCCTCCAAATTTTAAATTTATTTTGTAATATATCATTAATGCTAATACCATGTATTCTAGAGCTATAATTCCAGTGTTGTTTTAACTATATATAAATATAATACTCATCACCAATGTTTTTACCATTTTTTGTATATTTATCTTGACTCATCTGATCACCAGATTCTAATTCAAAACCATTTATTCTAACTTCTGTAAGGTGCTAATTCCTGAGTTCTTAGAAAATTCTTGCAAATGCTAATTCCTTGAATTCTAGAGAGTTTGCTTACATGCCTTTGCAGGAGGGGTGGCCCAAAATACTGCAAACTCCTGATTCATGGTCTCTCTTATTCTCCTATGCCTACACTGTGGTGACTCCATGTTTTTCAAAGCCTCCAGCCCATGTCTTTTTCTGGTCTTTCCACTTATTATATATATTAAACAAATCTAGCCCTTGTATCTAATTTAAATCATGCATGATCATGCATTTCAGCACATTTAACAAAAAAACCTCTTTATTCTCAACATATCAGGAGTGTCTCAAGGGACAGAAACCAGGAACAGAAGCATTTCTAGAGAAATTTTCATAAGATGAGGCTCTATATTTTTCCCAAAGGAGAGAAAGTAAACTGTCAGATCAAGCATGGGCCCCAAATGTGGCAATGCACACTGTCACAGCCTCAATCAGTGTCTGAAGCAGGGTCATACCTCAGTTGGTATGTGGGCCAAAAGGCATTTGCAACTCTAAATAAAAAAAACTAAAAAATAAAAAAAAAAACAAAAACACGATGAAATGATTCTCCATTTGGGGGAAAAATATGCTCAATAAAACTGTCAATGGGTTCATTGAGTGAGTAAGAACCTTGACTATGGCAGTGACCTAAGGATAGTCTCATGATTCTGCCTGCTATACCTGTTATAGGCATTGGAGCTGGAGCACACAGTGCTAGGAATTGTCAGGCTTCTGAGTCCAAGCTAAGCCATCCTATCCCCTGTGACCTGCATGTATACATCCAGATGGCCAGTTCCTGCCTTAACTGATGACATTCCACCACAAAAGAAGTGAAAATGGCCTGTTCCTGTCTTAACTGATGACATTATCTTGTGAAATTCCTTCTCCTGGCTCATCCTGGCTCAAAAGCTCCCCTACTGAGCACCTTGTGACCCCACCTTCCCGCCAGAGAACAACACCCCTTTGACTGTAATTTTCCTTTACCTACCCAAATCCTATAAAATGGCCCCACCCATATCTCCCTTCACTCTCTTTTCGGACTCGGCCTAC

BLAT this 11,083-bp matches to chr13(-):103443256-103432189 (99.8% identity, in two blocks) except for a 10-bp unmatched sequence at the beginning ‘TCAGTAAATT’

**Fusion sequence (f2): chr13(-):70420369::[ins10bp]::103443256.**

1. **Intra-strand template switch (f3)**

Left clip sequence:


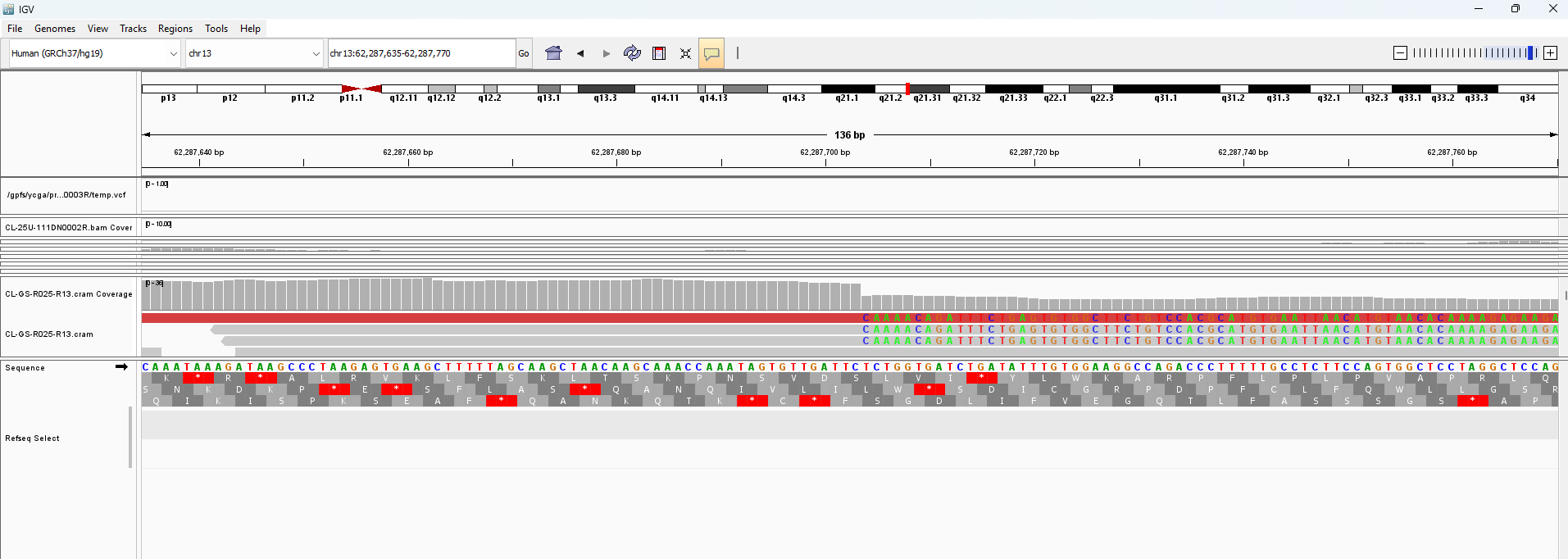

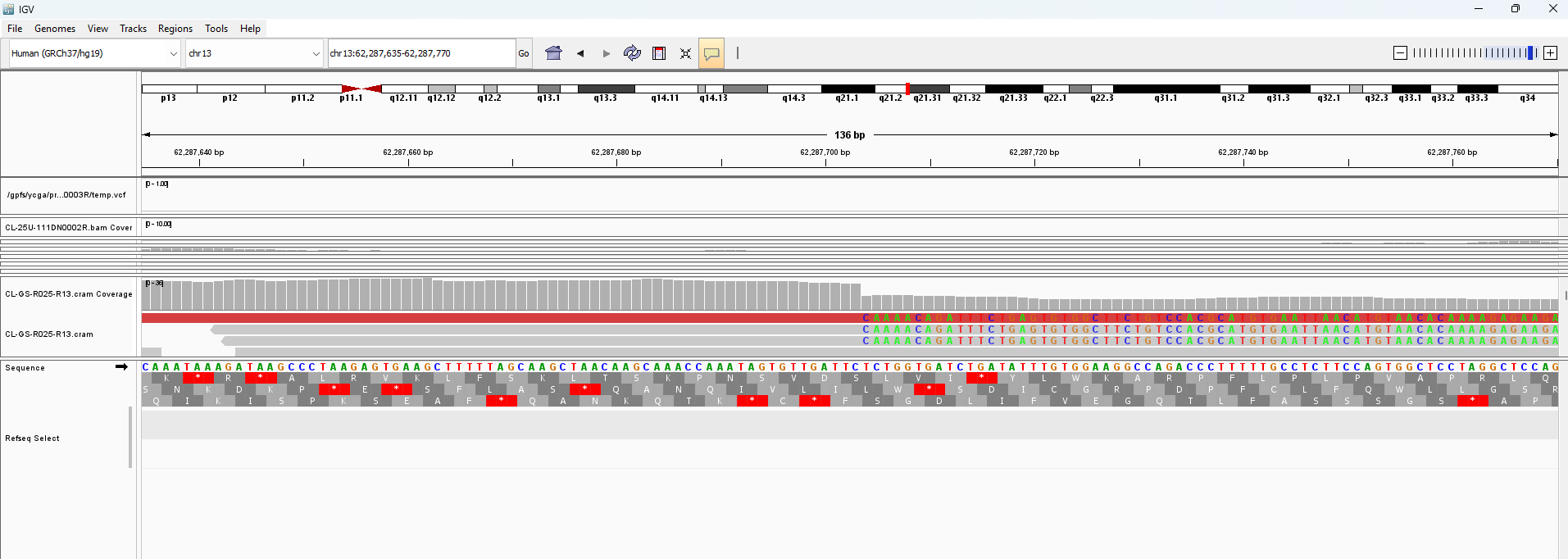


Right clip sequence:


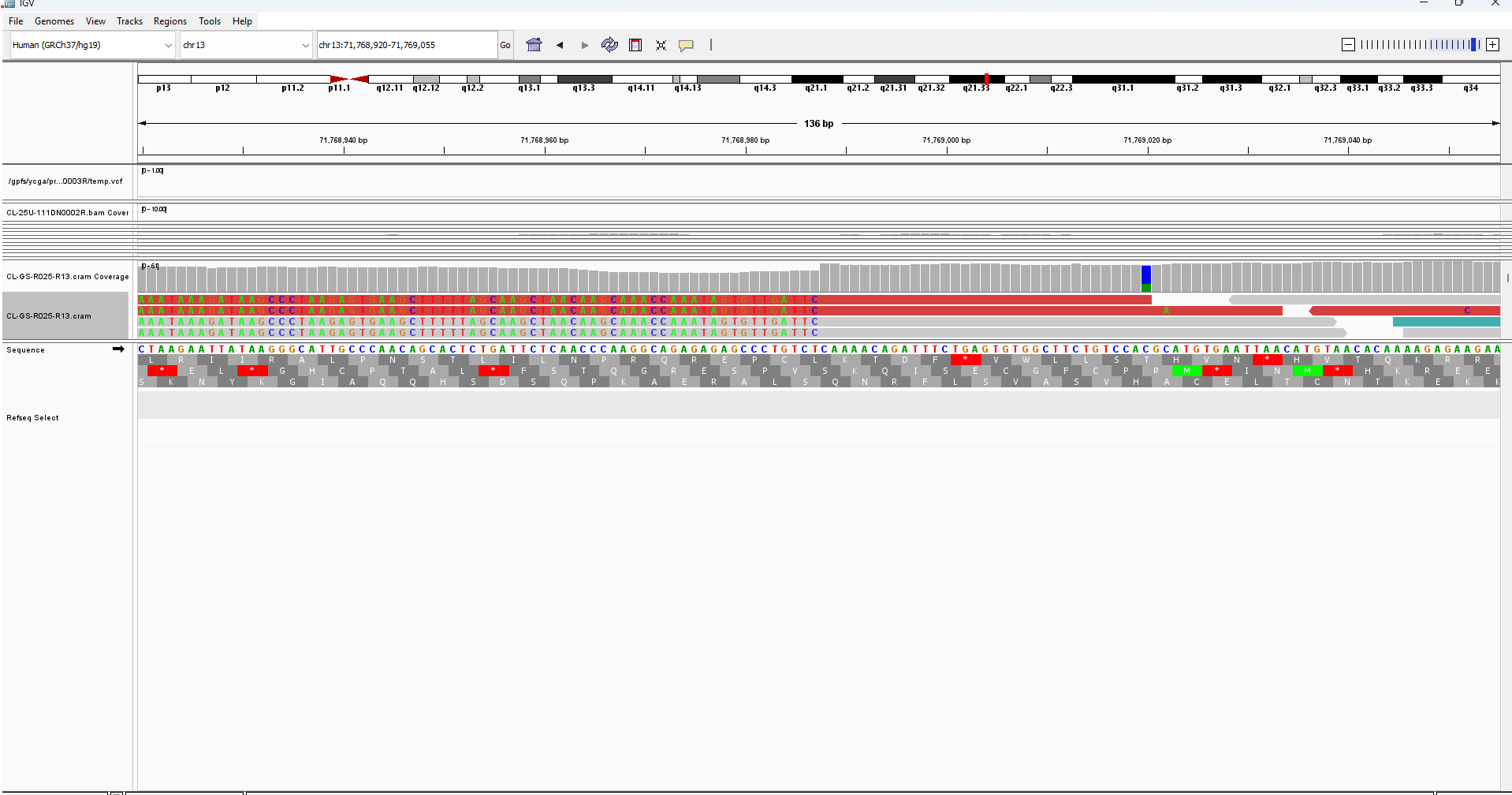


**Chr 13(+):** 60933707-AAATAGTGTTGAT**TCT**CTGGTGATCTGATATTT-60933739

**Chr 13(+):**  70420057-GAGAGAGCCCTG**TCT**CAAAACAGATTTCTGAGTG-70420090

**Fusion sequence (f3):** AAATAGTGTTGATTCCAAACAGATTTCTGAGTG

**ISCN:** 60933721::70420072 (mediated by microhology sequence ‘TCT’)

1. **Intra-strand template switch (f4)**

Left-clip sequence:


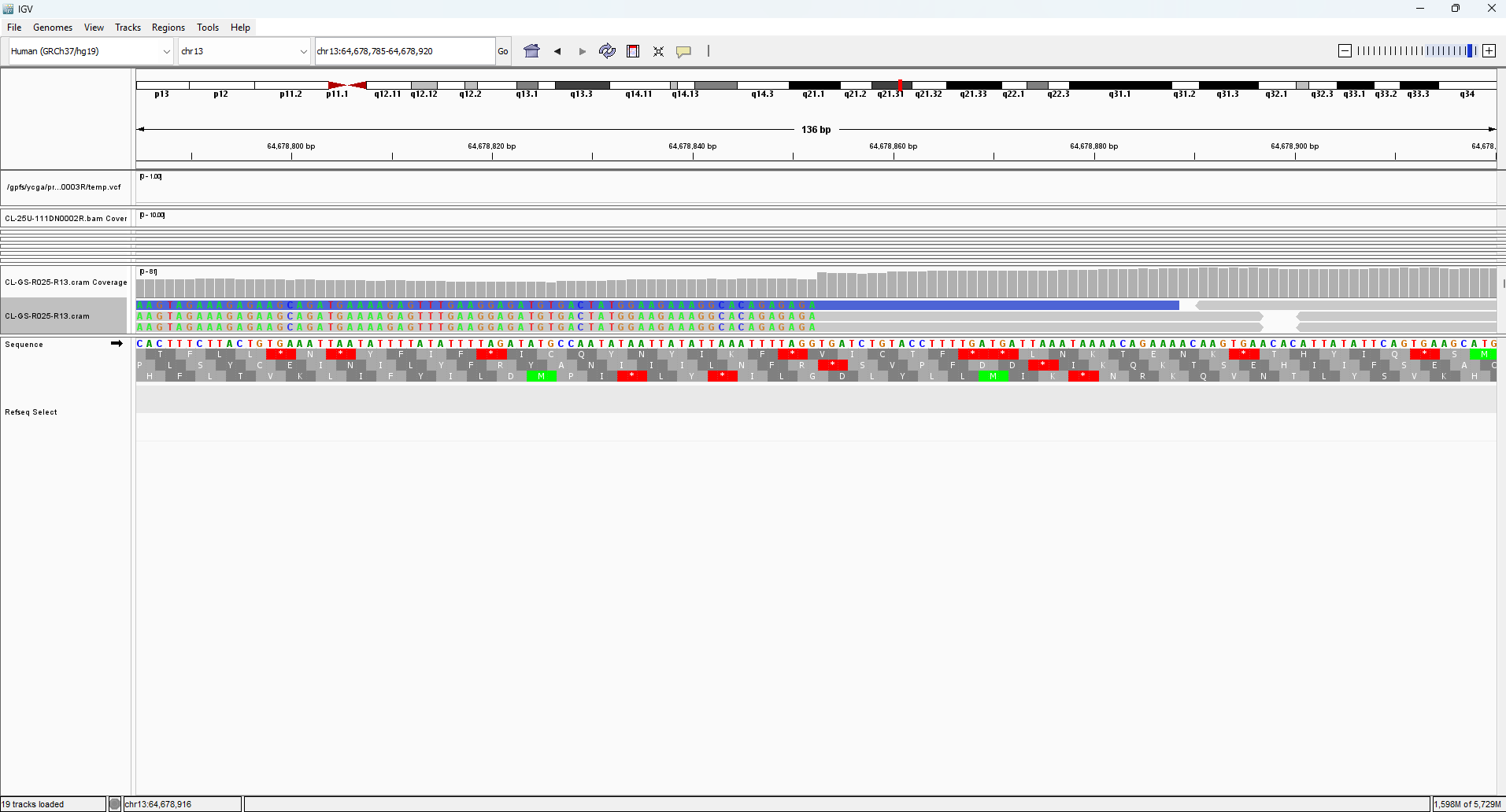

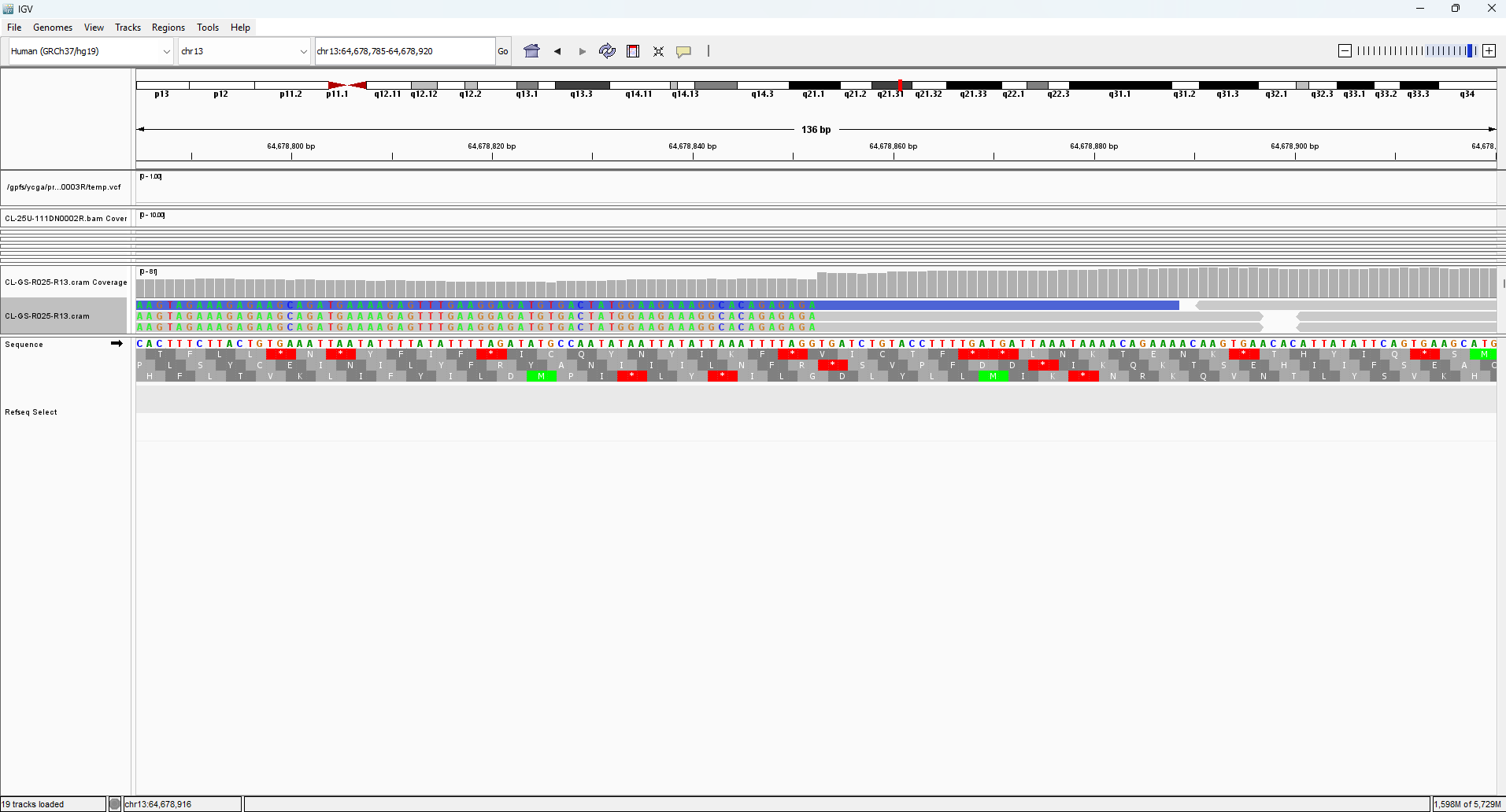


Right-clip sequence:


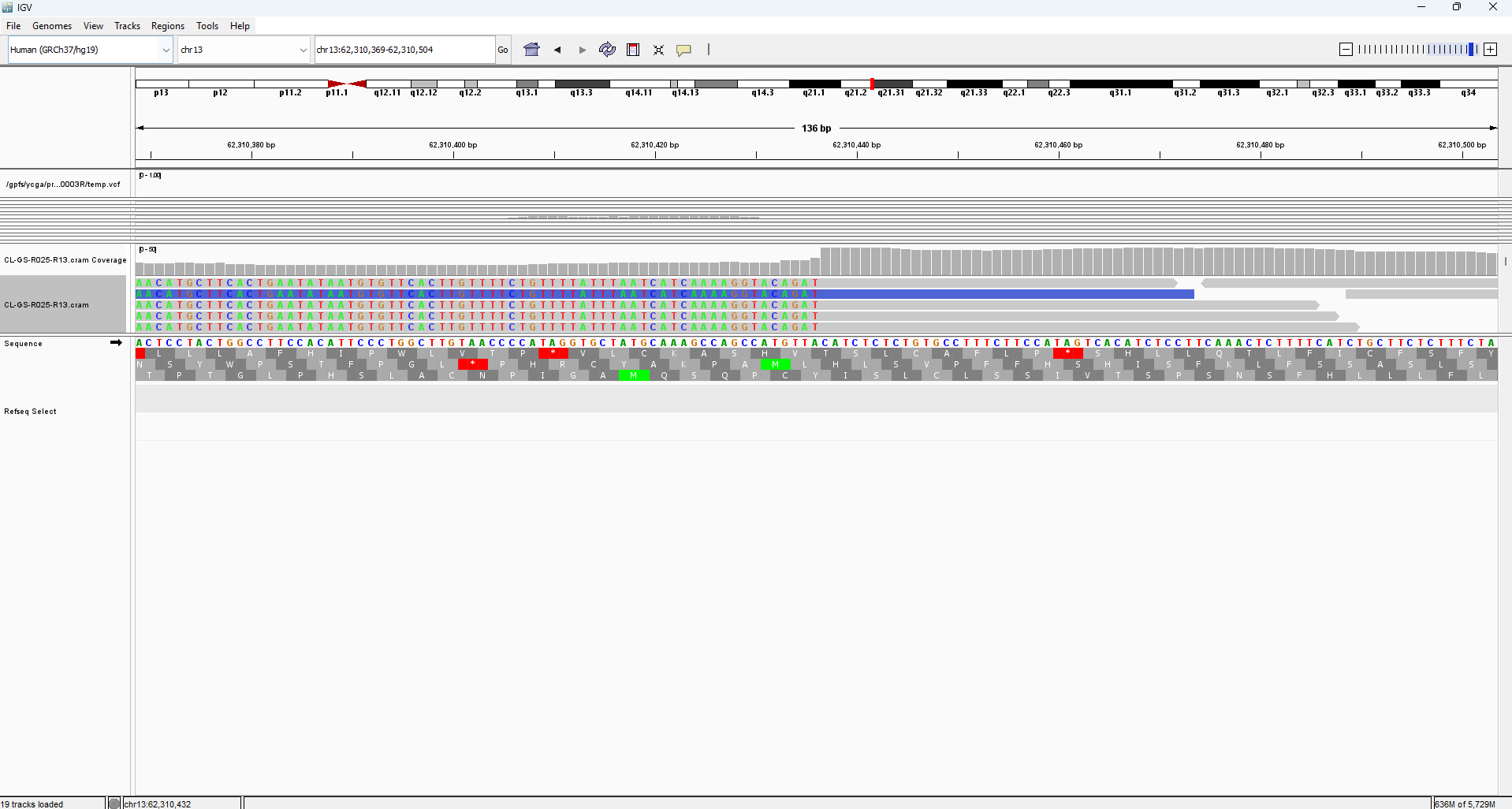


**Chr13(+):** 63326501-TTATATTAAATTGTAGGTGATCTGTACCTTTTGAT-63325535

**Chr13(-):** 63326501-AATATAATTTAACATCC**AC**TAGACATGGAAAACTA-63325535

**Chr13(+):** 60956407-GCAAAGCCAGCCATGTTA**CA**TCTCTCTGTGCCTTTC-60956442

**Fusion sequence (f4, -/+):** ATCAAAAGGTACAGAT**CA**TCTCTCTGTGCCTTTC

**ISCN:** 63326518::60956427 (mediated by microhomology sequence ‘CA’)

1. **Intra-strand template switch (f5)**

Left-clip sequence:


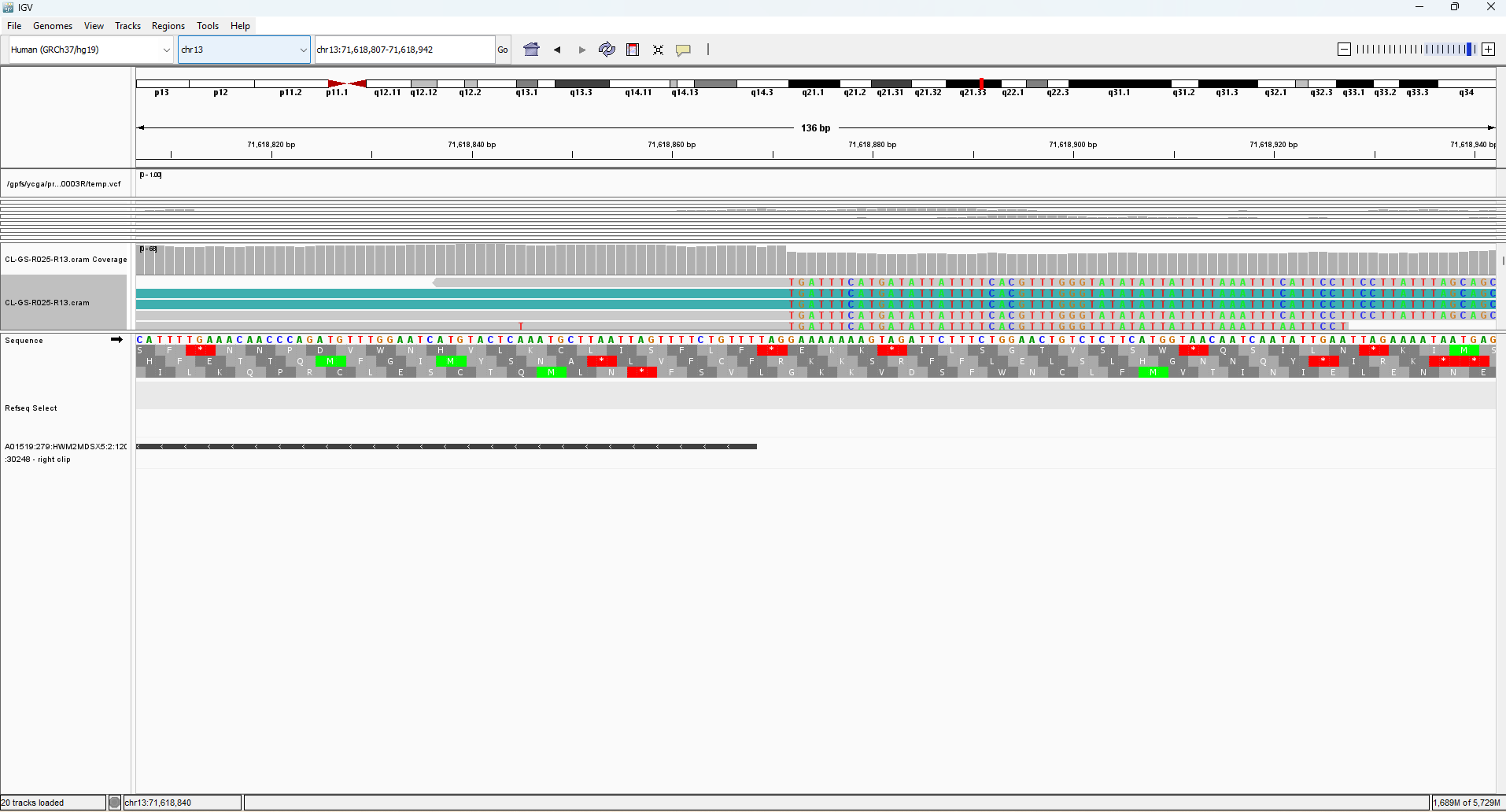

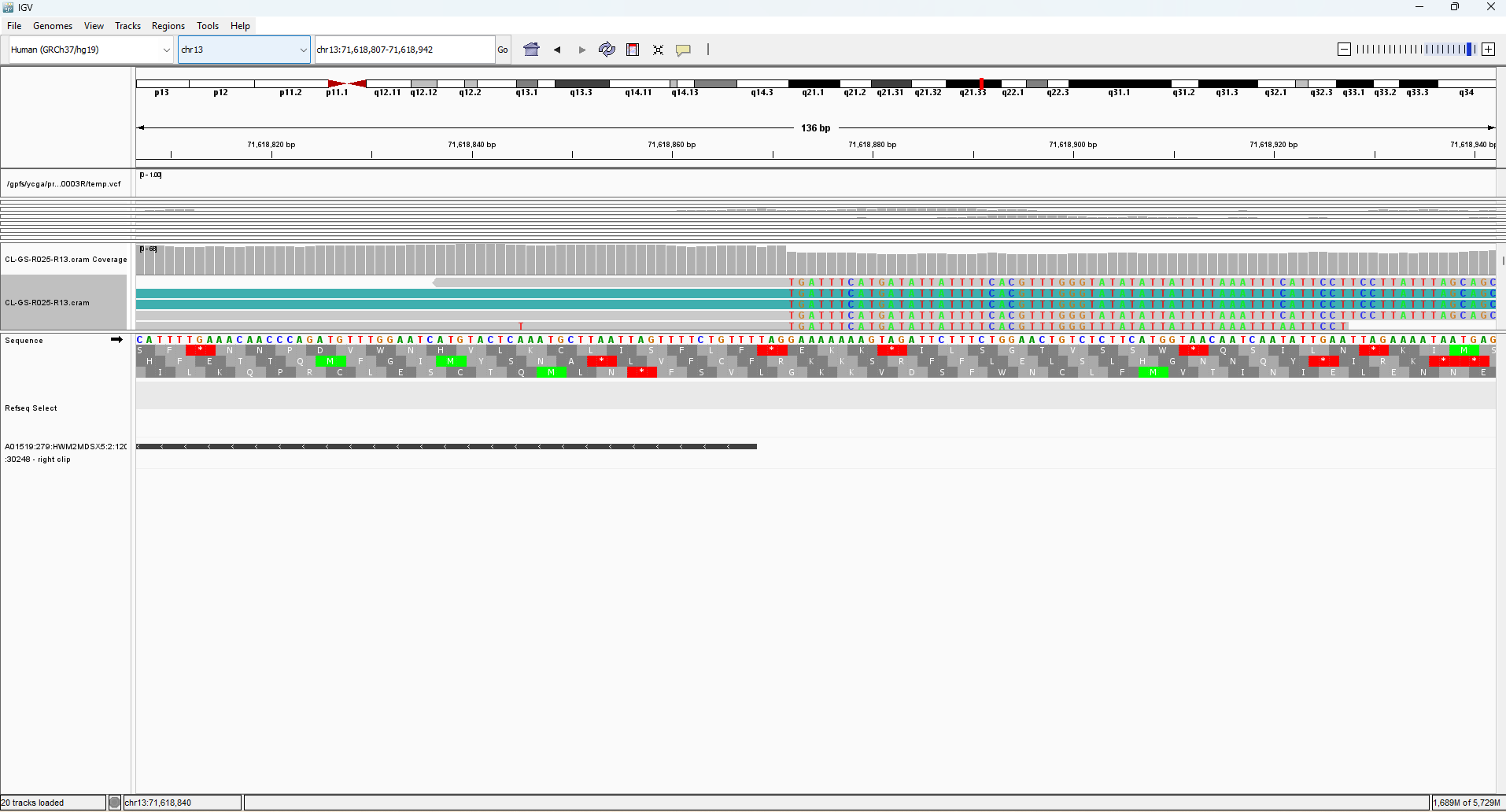


Right-clip sequence:


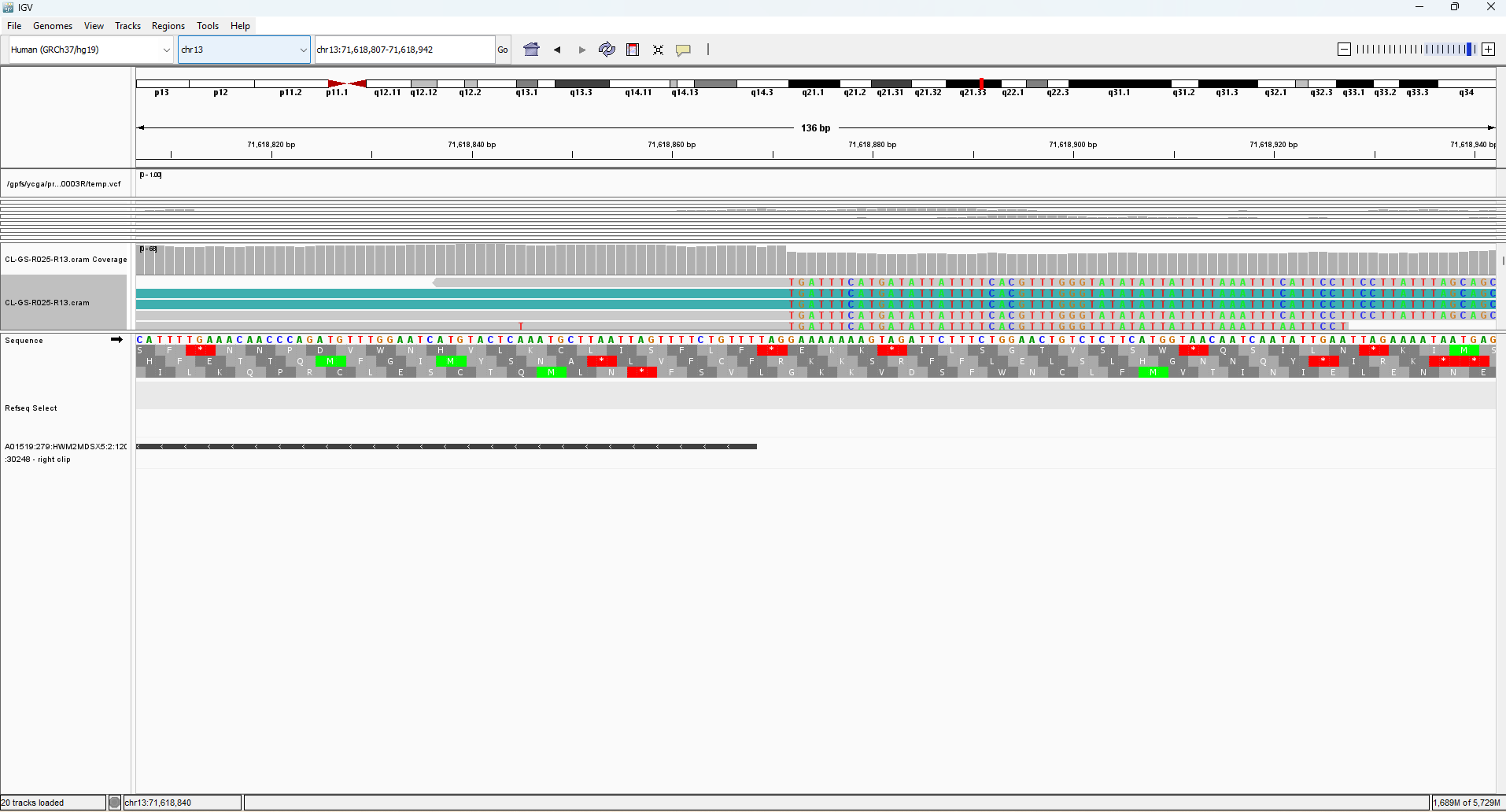

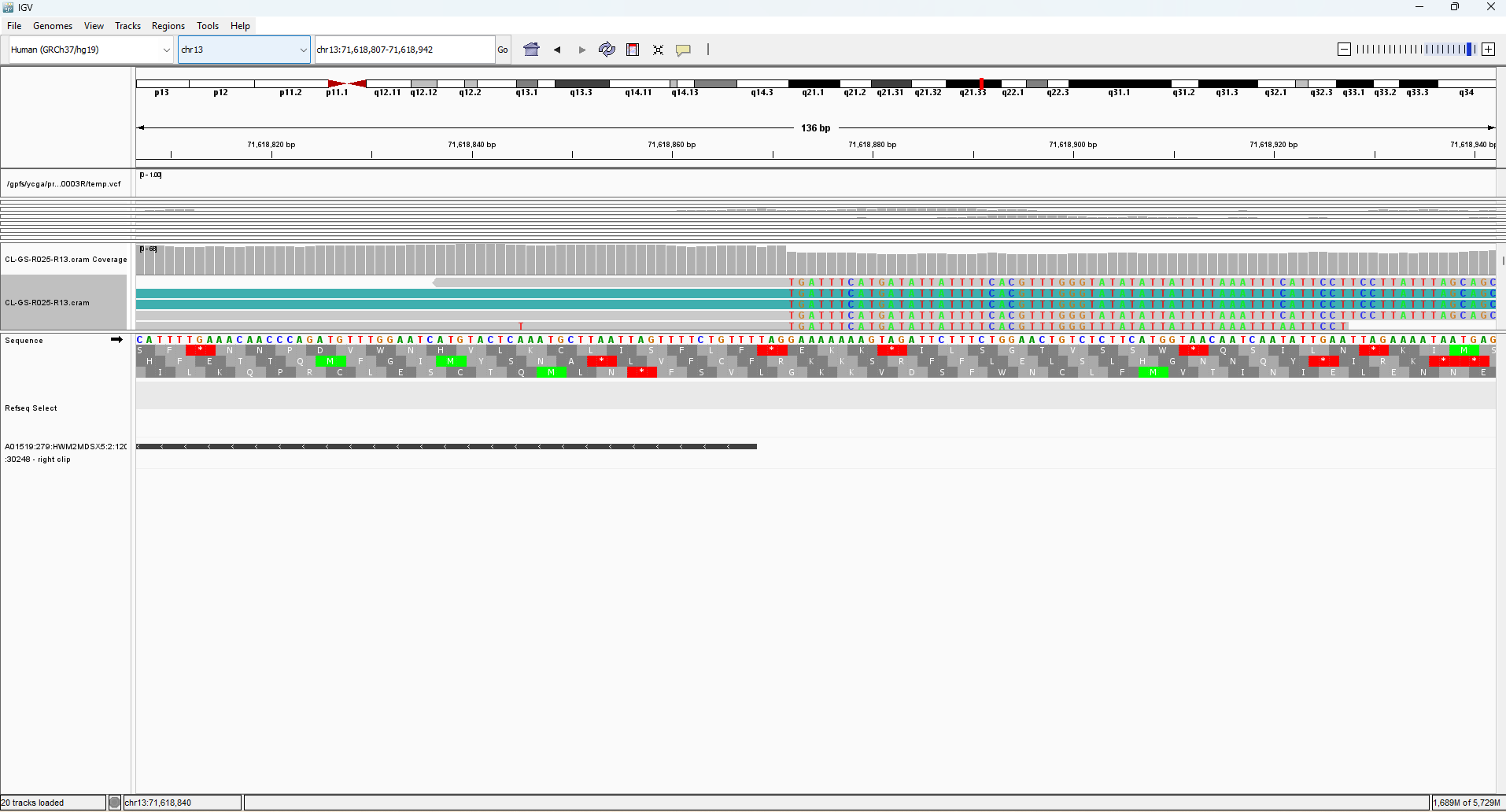


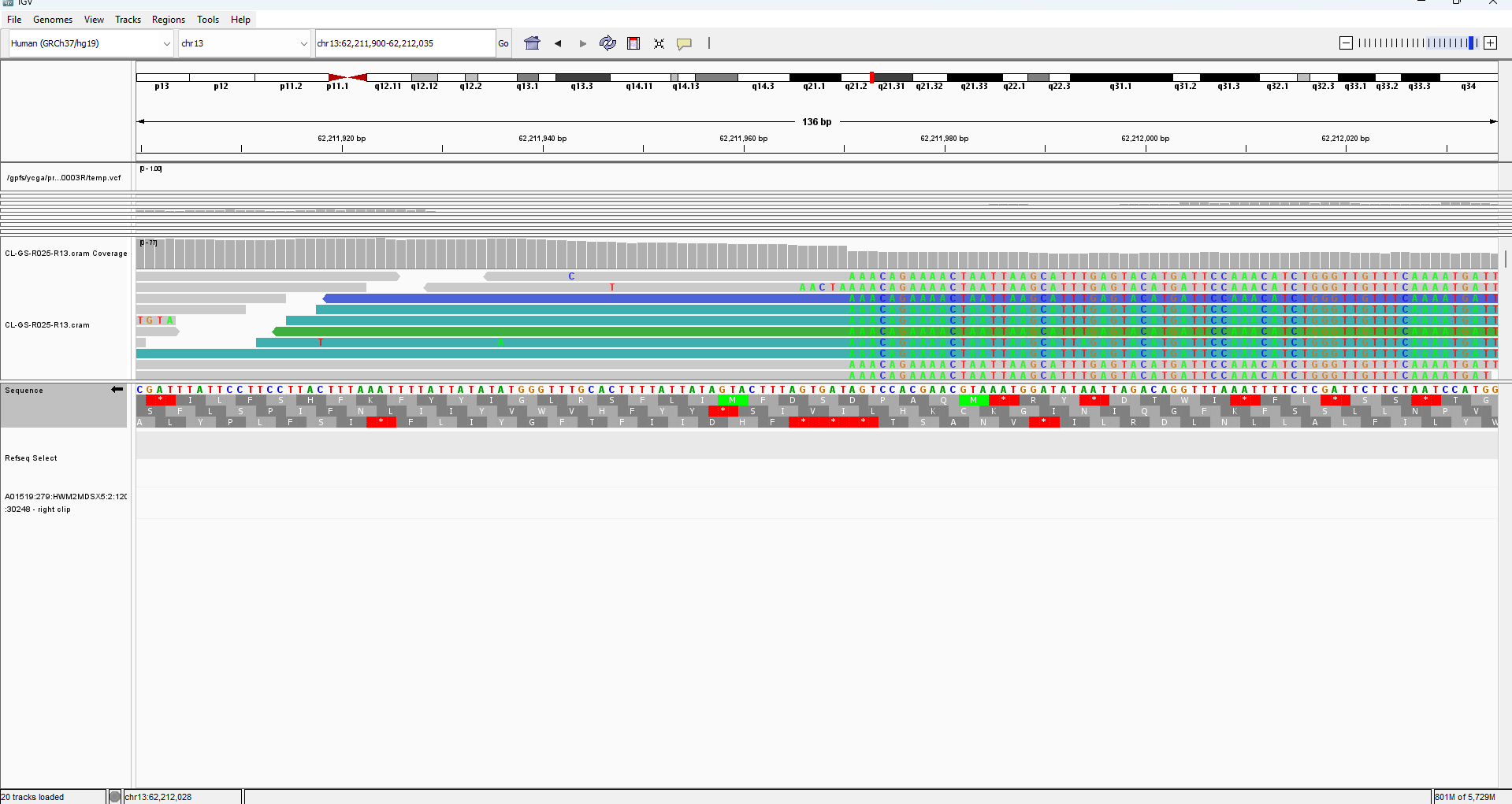


**Chr13(+):** 70269431-TTAATTAGTTTTCTGTTT**TAG**GAAAAAAAGTAGATT-70269466

**Chr13(-):** 70269431-AATTAATCAAAAGACAAA**ATC**CTTTTTTTCATCTAA-70269466

**Chr13(-):** 60857958-TTATAGTACTTTAGT**GAT**AGTCCACGAACGTAAATG-60857993

**Fusion sequence (f5, +/-):** TTATAGTACTTTAGT**GAT**AAACAGAAAACTAATTAA

**ISCN:**  70269448:: 60857975 (mediated by microhomology sequence ‘GAT’)
